# Predicted HLA Class I and Class II Epitopes From Licensed Vaccines Are Largely Conserved in New SARS-CoV-2 Omicron Variant of Concern

**DOI:** 10.3389/fimmu.2022.832889

**Published:** 2022-01-28

**Authors:** Daniel López

**Affiliations:** Presentation and Immune Regulation Unit, Centro Nacional de Microbiología, Instituto de Salud Carlos III, Majadahonda, Spain

**Keywords:** SARS-CoV-2, escape mutant, vaccine, HLA class 1, HLA class 2

## Abstract

The potential effect of emerging SARS-CoV-2 variants on vaccine efficacy is an issue of critical importance. In this study, the possible impact of mutations that facilitate virus escape from the cytotoxic and the helper cellular immune responses in the new SARS-CoV-2 Omicron variant of concern was analyzed for the 551 and 41 most abundant HLA class I and II alleles, respectively. Computational prediction showed that almost all of these 592 alleles, which cover **>**90% of the human population, contain enough epitopes without escape mutations in the emerging SARS-CoV-2 Omicron variant of concern. These data suggest that both cytotoxic and helper cellular immune protection elicited by currently licensed vaccines are virtually unaffected by the highly contagious SARS-CoV-2 Omicron variant of concern.

## Introduction

The current vaccine prophylaxis deployed globally is perhaps the most important factor in social protection and economic recovery against the COVID-19 pandemic. Vaccine effectiveness against the new emerging SARS-CoV-2 variants is a challenging issue in the control of this new epidemic (1). All licensed formulations are based on the original D614 spike protein sequence of the Wuhan-1 wild-type strain. However, in these almost 2 years of epidemic, where SARS-CoV-2 has gone from being a local pathogen to a global pandemic, the evolutionary pressure is generating an increased number of novel variants and sub-variants (2). Changes in the amino acid sequence of the SARS-CoV-2 spike protein present in these new viruses may affect several stages of the replicative cycle of the virus and/or efficacy of the humoral and/or cellular immune responses. Strong activation of the three arms of adaptive immunity—neutralizing antibodies, helper CD4^+^ T lymphocytes, and cytotoxic CD8^+^ T lymphocytes—are relevant after both SARS-CoV-2 natural infection and vaccination (3). Changes in the spike protein between new SARS-CoV-2 variants compared to Wuhan-1 wild-type strain included in current formulations in use can lead to the selection of escape mutant, which reduce or even eliminate one, several, or even all three arms of the adaptive immune response against this pandemic virus. In the last weeks, the heavily mutated SARS-CoV-2 B1.1.529 variant of concern (Omicron) seems to be spreading quickly across first South Africa and now to the rest of the world, raising doubts about vaccine efficacy.

The exquisite interaction of the receptor of the CD8^+^ or CD4^+^ T helper lymphocytes with pathogen peptides bound to HLA class I or II molecules not only triggers the activity of these T cells, but also initiates, regulates, or suppresses the other components of the adaptive immune responses. In absence of appropriate HLA class I- and II-restricted T-cell recognition, both cellular and humoral immune responses cannot be efficiently activated, and thus, the infective virus could spread within the whole organism with fatal results for the host. This whole highly complex set of immune events can be altered, or even suppressed by single changes in the virus epitope sequences that lead to a complete loss of antigen recognition. This extremely low tolerance to amino acid changes in the antigen recognition can favor epitope escape at the T-cell level, rendering ineffective the lymphocytes previously activated by the administration of vaccines. Thereby, confirmed cases of re-infection associated with different viral genotypes were detected at the beginning of the pandemic (4), and re-infection of immunized individuals by mutational evasion is common in other RNA viruses such as influenza (5).

Altogether, the analysis of the influence of mismatches between the immune response elicited by currently licensed vaccines and emerging SARS-CoV-2 variants is of primary importance. In this study, we have approached this aspect focused on the cytotoxic and helper immune responses against the last new SARS-CoV-2 B1.1.529 variant of concern (Omicron). Although some HLA supertype-dependent differences in the predicted quality of cytotoxic, but not of helper, protection against this strain of concern was observed, this effect is still very minor compared to the global cytotoxic and helper responses elicited with current licensed vaccines.

## Methods

### HLA Class I Epitope Prediction and Analysis

Non-redundant HLA class I and II epitopes between 8 and 12 residues in the spike protein of the SARS-CoV-2 reference proteome (Wuhan-1; RefSeq: NC_045512.2) including the modifications added to Moderna mRNA-1273, Pfizer BNT162b2, and Janssen Ad26.COV2.S vaccines and the SARS-CoV-2 B1.1.529 variant of concern (Omicron), which accumulate the following changes versus SARS-CoV-2 reference proteome: A67V, Δ69-70, T95I, G142D, Δ143-145, Δ211, L212I, ins214EPE, G339D, S371L, S373P, S375F, K417N, N440K, G446S, S477N, T478K, E484A, Q493R, G496S, Q498R, N501Y, Y505H, T547K, D614G, H655Y, N679K, P681H, N764K, D796Y, N856K, Q954H, N969K, and L981F, were predicted using two predictors. First, the peptides considered “Strong Binders” by NetMHCIpan EL 4.1 (6) for HLA class I ligands or NetMHCIIpan EL 4.0 (6) for HLA class II ligands were selected. For redundant epitopes, those sharing the same binding core for the same allele, only the one with the highest score was considered per allele. Non-redundant epitopes were further verified through the NetMHCIpan BA 4.1 (6) or NetMHCIIpan BA 4.0 (6) algorithms. These verified non-redundant epitopes did not match any of those predicted for a random sequence with the same length and residue composition than the reference SARS-CoV-2 spike protein generated with the EXPASY RandSeq tool (https://web.expasy.org/randseq/). Predictions were restricted to alleles that share anchor residues of the 551 alleles including in the twelve HLA class I supertypes (7) and 41 alleles including in the ten HLA class II supertypes (8, 9). To further test the specificity and sensitivity of NetMHCIpan EL 4.1 algorithm, substitution of all Pro and Arg/Gln by Ala yielded no epitopes for HLA-B*07:02 and HLA-B*27:05 alleles, respectively, as these amino acids are their respective anchor motif residues.

## Results and Discussion

In contrast to the study of humoral immune response when the escape mutants affecting the antibody response can be carried out experimentally, the impressive degree of HLA class I and II polymorphism distributed in the human population, with at least 24,000 and 8,000 alleles identified to date respectively, can only be approached bioinformatically. However, many HLA class I and II molecules identified have been grouped first in families, later in superfamilies, and finally in twelve and ten canonical HLA class I and II types, respectively, sharing strong similarities at the peptide–ligand specificity level, termed supertypes, that cover >90% of the world population regardless of ethnicity (7–9). These 551 or 41 HLA class I or II alleles experimentally shared the same motifs within their respective supertype showing exact matches for residues at the specific pockets of the HLA molecule. While thousands and thousands of HLA class I and class II alleles have been reported (10), the utilization of supertypes significantly reduces data complexity and facilitates herd immunity analysis. Thus, the first computational prediction to the theoretical epitopes from the SARS-CoV-2 spike protein, the only protein included in internationally licensed vaccines, was carried out. These epitopes could be presented by each of the 551 HLA class I or 41 HLA class II alleles associated to the twelve or ten canonical HLA class I and II supertypes, respectively. The latest versions of the universal and neural network-based netMHCpan EL and BA algorithms were utilized, which outperforms any other method so far (6) and are recommended by the central Immune Epitope Database and Analysis Resource (11).

**Figure 1:**
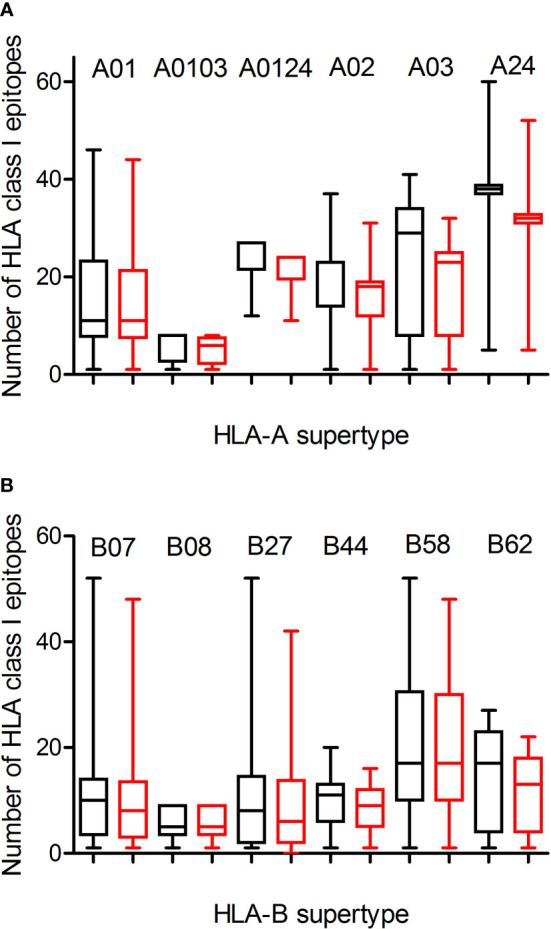
Average number of epitopes in the Wuhan-1 and Omicron spike protein sequences predicted for HLA class I alleles including in the 12 HLA class I supertypes. The median value is indicated. Box limits indicate the interquartile range. Whiskers are adjusted to maximal and minimal values. The number of epitopes in the Wuhan-1 (black) and Omicron (red) strains predicted for HLA class I alleles including in the 6 HLA-A and 6 HLA-B supertypes are depicted in panels **(A, B)**, respectively.

Predicted ligands for most HLA-A class I supertype molecules ranged by a few tens per allele except in the A0103 supertype with less than 9 epitopes per allele (**Figure 1A**; **Table 1**, and **Supplemental Table 1**). This supertype besides some individual alleles of other HLA-A supertypes that also showed low predicted epitope binding (**Figure 1**) would be candidates to be affected by escape mutants within SARS-CoV-2 variants. The influence of SARS-CoV-2 B1.1.529 variant of concern (Omicron), which is spreading rapidly against a backdrop of ongoing Delta-variant transmission and shows multiple mutations in the spike protein, in the vaccine-derived immunity is currently cause for much speculation and even alarm worldwide. Thus, a predictive analysis of the impact of mutations described in the Omicron variant of concern over the HLA-A class I supertypes was carried out.

**Table 1:** Summary of average number of epitopes in the Wuhan-1 and Omicron spike protein sequences predicted for HLA class I and II alleles including in the 12 and 10 HLA class I or class II supertypes, resepctively.

HLA superfamily	Number of epitopes (mean ± SEM)		Vaccines/Omicron *p*-value
	Vaccines	Omicron	
A01	14.9 ± 1.4	13.7 ± 1.3	n.s.
A0103	6.3 ± 1.8	5.3 ± 1.5	n.s.
A0124	24.1 ± 1.9	21.6 ± 1.7	n.s.
A02	18.7 ± 1.0	15.3 ± 0.9	<0.5
A03	22.6 ± 1.6	17.5 ± 1.1	<0.01
A24	38.7 ± 2.1	32.7 ± 1.8	<0.5
B07	11.2 ± 1.0	10.5 ± 1.0	n.s.
B08	5.8 ± 0.9	5.8 ± 0.9	n.s.
B27	11.3 ± 1.7	9.2 ± 1.4	n.s.
B44	9.9 ± 0.6	8.8 ± 0.5	n.s.
B58	19.4 ± 3.2	18.9 ± 3.1	n.s.
B62	14.7 ± 1.6	11.8 ± 1.2	n.s.
DR1	12.5 ± 0.9	12.1 ± 0.8	n.s.
DR52	21.0 ± 1.0	19.5 ± 0.5	n.s.
DR53	11.0 ± 3.1	10.8 ± 3.2	n.s.
DP1	16.0 ± 0.9	15.8 ± 0.8	n.s.
DP3	13.0 ± 1.0	12.5 ± 0.5	n.s.
DQ2	9.0 ± 1.0	9.0 ± 1.0	n.s.
DQ4	14.0 ± 1.4	13.8 ± 1.6	n.s.
DQ5	11.7 ± 2.3	11.7 ± 2.3	n.s.
DQ7	11.4 ± 1.0	11.2 ± 1.1	n.s.
DQ8	13.5 ± 0.5	13.5 ± 0.5	n.s.

ns, not significant.

The changes in the Omicron variant of concern generated little loss of HLA-A-restricted epitopes derived from SARS-CoV-2 vaccines in the protein spike. In the A01, A0103, and A0124 supertypes the average escape epitope rate was not statistically significant with a loss of only 1-2 epitopes by allele on average (**Figure 1A** and **Table 1**). In contrast, for the A02, A03, and A24 supertypes the differences were statistically significant with between 3 and 5 epitopes by allele on average mutated on Omicron variant of concern, although most epitopes presented by these HLA-A alleles remained conserved between both virus strains (**Figure 1A**; **Table 1**, and **Supplementary Table 1**). In addition, none of HLA-A class I alleles from all supertypes with low number of predicted epitopes in the vaccine sequence was affected by the Omicron sequence. For example, for 8 alleles from HLA-A*03 family (A03 supertype) the predicted epitopes in the Wuhan-1 wild-type strain ranging 1-4 peptides were all conserved in the Omicron sequence.

Similarly to HLA-A locus, a predictive analysis of the impact of mutations described in the Omicron variant of concern over the HLA-B class I supertypes was also carried out. As **Figure 1B** and **Table 1** show, the mutations selected in the Omicron variant of concern versus vaccine sequence practically not altered (0-3 epitopes on average) the number of conserved epitopes between both strains for the six HLA-B supertypes analyzed. This is true also for HLA-B class I molecules with low number of predicted cytotoxic epitopes in the vaccine strain. The 1-5 predicted epitopes for 21 alleles including in the HLA-B*51 family, for 3 alleles including in the HLA-B*78 family (both from B07 supertype), and for 7 alleles including in the HLA-B*18 family (B44 supertype) remained conserved in the Omicron variant of concern (**Supplementary Table 1**). Also, the one predicted epitope for 4 alleles including in the HLA-B*38 family (B27 supertype) and 6 alleles including in the HLA-B*52 family (B62 supertype) were not modified in the Omicron sequence (**Supplementary Table 1**). In contrast, the HLA-B*48 family (B27 supertype) was the most affected by the Omicron variant of concern. Two of the four HLA-B*48:09, -B*48:10, -B*48:12, and -B*48:13 predicted epitopes in vaccine strain were mutated in the Omicron variant of concern (**Supplementary Table 1**). Also, one of three HLA-B*48:03 predicted epitopes in Wuhan-1 strain were mutated in the Omicron strain (**Supplementary Table 1**). Finally, the two predicted epitopes for the HLA-B*48:05 subtype, and the one predicted peptide for HLA-B*48:01, and -B*48:04 subtypes in the vaccine sequence were modified in the Omicron variant of concern (**Supplementary Table 1**). These three HLA-B*48 subtypes are the only ones among the 551 HLA class I alleles analyzed in which Omicron mutations would completely eliminate the cytotoxic cellular immune response generated by the vaccines. HLA-B*48:01 allele frequency is relevant in diverse human populations. This HLA-B allele is expressed in the 26% of the Amis, an indigenous Austronesian ethnic group native of Taiwan. Also, the 22% of Gila River Amerindian from Arizona are positive for this HLA-B allele. In addition, 19% and 17% of Atayal and Taroko Taiwanese indigenous peoples, respectively, express HLA-B*48:01. Finally the HLA-B*48:01 allele frequency is greater than 10% in other diverse Amerindian populations from Peru and US, Maori from New Zealand, and American Samoa. Lastly, the HLA-B*48:04 allele frequency is very minor (<1%) in some populations of China and India just like HLA-B*48:05 in other populations of Israel, Jordan, and Saudi Arabia. As all these HLA-B*48^+^ populations represent less than a million inhabitants worldwide, the impact of Omicron variant of concern on the cytotoxic T-cell immune response derived from SARS-CoV-2 vaccines would be very marginal.

**Figure 2:**
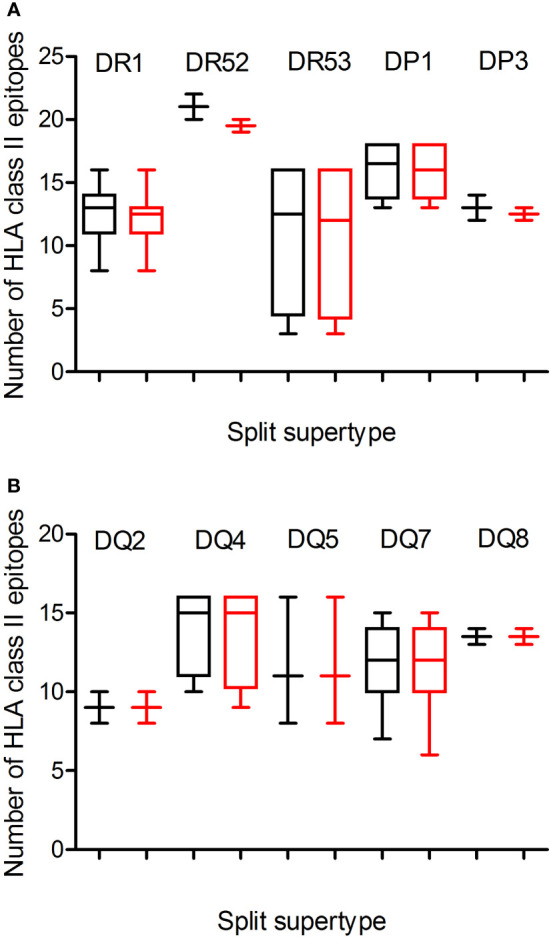
Average number of epitopes in the Wuhan-1 and Omicron spike protein sequences predicted for HLA class II alleles including in the 10 HLA class II supertypes. The median value is indicated. Box limits indicate the interquartile range. Whiskers are adjusted to maximal and minimal values. The number of epitopes in the Wuhan-1 (black) and Omicron (red) strains predicted for HLA class I alleles including in the 3 HLA-DR and -DP or 5-DQ supertypes are depicted in panels **(A, B)**, respectively.

Additionally, a predictive analysis of the impact of mutations described in the Omicron variant of concern over the HLA-DR, -DP, and -DQ class II supertypes was also carried out. As **Figure 2**; **Table 1**, and **Supplemental Table 2** show, the mutations selected in the Omicron variant of concern versus vaccine sequence practically did not alter the number of conserved epitopes between both strains for the three DR, two DP, and five DQ supertypes analyzed. Only 12 of the 530 (the 2%) predicted epitopes for the 44 HLA class II analyzed were not conserved in the Omicron variant of concern versus vaccine strain. In addition, none of HLA class II alleles from all supertypes with low number of predicted epitopes in the vaccine sequence was affected by the Omicron strain. Thus, the impact of Omicron variant of concern in the helper T-cell immune response would be even less relevant than in cytotoxic T cell immune response.

In our previous study about the effect of emerging SARS-CoV-2 variants identified to May 2021 on cytotoxic T-cell response generated by the licensed vaccines, a prediction of the number of intact epitopes for some representative alleles of the twelve HLA class I supertypes was calculated after random progressive position sets ranging from the length of the full spike protein (12). In this hypothetical scenario, 39 random mutations in the spike protein sequence did not prevent the fact that the 75% of predicted cytotoxic epitopes derived from SARS-CoV-2 vaccines remained unaltered (12). In the real scenario analyzed in the current study, the 30 changes, 3 deletions, and 1 insertion generated by the Omicron variant of concern versus current SARS-CoV-2 vaccines, only with the A03 supertype was a similar (26%) escape mutation rate found. A02 was the second supertype affected (21%), while in the other 10 HLA class I supertypes, this rate was less than 20%, resulting in a 12% escape mutation rate on average for the 12 HLA class I supertypes analyzed, less than half of the theoretical rate calculated by random mutation (12). B58, with only 2% altered epitopes, and B08 without predicted epitopes modified in Omicron strain versus SARS-CoV-2 vaccines were the HLA class I supertypes least impacted by the Omicron variant of concern. The changes in this new SARS-CoV-2 variant are not randomly distributed, but many of them are usually very close to each other, i.e., A67V and Δ69-70; G142D and Δ143-145; L212I and ins214EPE; S371L, S373P, and S375F; S477N, T478K, and E484A; and especially Q493R, G496S, Q498R, N501Y, and Y505H changes. Thus, the destructive effect of these mutations was concentrated on a small number of epitopes, leaving very large regions of the spike protein unchanged. In this context, currently 1,312 experimentally detected epitopes restricted by HLA class I and II have been identified [Immune Epitope Database and Analysis Resource (11)]. Of these, only 125 (9%) were modified by the Omicron variant of concern (**Supplemental Table 3**). Therefore, most HLA class I- and II-restricted peptides remain preserved. In addition, in the hypothetical scenario using random progressive mutation on SARS-CoV-2 spike protein previously described (12), an average of 81 mutations were necessary to destroy half of the predicted CD8^+^ T-cell epitopes generated by vaccination and restricted by all HLA class I supertypes. Thus, taking into account the above, it is probable that the impact of 80 mutations in the spike protein screened by natural selection was far less disruptive than the hypothetical scenario, and still, most HLA class I- and II-restricted peptides of multiple HLA supertypes remain preserved. This implies that for current vaccines to lose efficacy with respect to cellular immunity against new SARS-CoV-2 variants, they should accumulate many more mutations in the spike protein, and that these changes should also be distributed throughout the protein sequence derived from licensed vaccines. Therefore, since generally SARS-CoV-2 mutations (as in many other viruses) are associated with selection by factors such as interaction with cell receptor or transmissibility, as suggested by epidemiological data for the principal strains of concern (13–15), it appears unlikely that the simple accumulation of other new several dozen more mutations in the spike protein *per se* will achieve critical mass in a reasonable time to allow either cytotoxic or helper immune evasion for most HLA class I and/or class II alleles, making current licensed vaccines ineffective. In addition, the HLA polymorphism is widely distributed in the human population with few inbred groups, generally aborigines living in remote regions with little contact with the outside world. Thus, the immune pressure so that omicron or any other new variant that may appear in the future could develop a sufficient number of escape mutants to evade the cellular immune response generated by current vaccines is very low.

In conclusion, HLA class I and class II molecules of most common human alleles can present enough unmodified ligands from SARS-CoV-2 Omicron variant to activate vaccine-generated CD4^+^ and CD8^+^ T lymphocytes.

## Data Availability Statement

The original contributions presented in the study are included in the article/**Supplementary Material**. Further inquiries can be directed to the corresponding author.

## Author Contributions

All contributions have been made by DL.

## Funding

This research was supported by grant MPY 388/18 of “Acción Estratégica en Salud” from the ISCIII.

## Conflict of Interest

The author declares that the research was conducted in the absence of any commercial or financial relationships that could be construed as a potential conflict of interest.

## Publisher’s Note

All claims expressed in this article are solely those of the authors and do not necessarily represent those of their affiliated organizations, or those of the publisher, the editors and the reviewers. Any product that may be evaluated in this article, or claim that may be made by its manufacturer, is not guaranteed or endorsed by the publisher.
