# Supplementary material for: Predicted HLA Class I and Class II Epitopes From Licensed Vaccines Are Largely Conserved in New SARS-CoV-2 Omicron Variant of Concern
Source: Front Immunol. 2022 Jan 28;13:832889. doi: 10.3389/fimmu.2022.832889 (PMC8831693; doi:10.3389/fimmu.2022.832889)
Supplement: Supplementary file 1 [file DataSheet_1.pdf]

Supertype	Allele	Begin position	Sequence	Score (EL)
A01	HLA-A*01:01	28	YTNSFTRGVY	0,8696
A01	HLA-A*01:01	28	YTNSFTRGVYY	0,9035
A01	HLA-A*01:01	135	FCNDPFLGVY	0,6253
A01	HLA-A*01:01	136	CNDPFLGVY	0,6408
A01	HLA-A*01:01	136	CNDPFLGVYY	0,6059
A01	HLA-A*01:01	160	YSSANNCTFEY	0,7273
A01	HLA-A*01:01	161	SSANNCTFEY	0,7703
A01	HLA-A*01:01	162	SANNCTFEY	0,6674
A01	HLA-A*01:01	256	SGWTAGAAAYY	0,7466
A01	HLA-A*01:01	257	GWTAGAAAYY	0,7407
A01	HLA-A*01:01	258	WTAGAAAYY	0,9622
A01	HLA-A*01:01	358	ISNCVADYSVLY	0,7628
A01	HLA-A*01:01	361	CVADYSVLY	0,8233
A01	HLA-A*01:01	362	VADYSVLY	0,8361
A01	HLA-A*01:01	370	NSASFSTFKCY	0,5177
A01	HLA-A*01:01	440	NLDSKVGGNY	0,893
A01	HLA-A*01:01	603	NTSNQVAVLY	0,8721
A01	HLA-A*01:01	604	TSNQVAVLY	0,9585
A01	HLA-A*01:01	746	STECSNLLLQY	0,8875
A01	HLA-A*01:01	828	LADAGFIKQY	0,928
A01	HLA-A*01:01	863	PLLTDEMIAQY	0,8726
A01	HLA-A*01:01	865	LTDEMIAQY	0,9987
A01	HLA-A*01:01	866	TDEMIAQY	0,8997
A01	HLA-A*01:01	1197	LIDLQELGKY	0,9031
A01	HLA-A*01:03	28	YTNSFTRGVY	0,8142
A01	HLA-A*01:03	28	YTNSFTRGVYY	0,8662
A01	HLA-A*01:03	160	YSSANNCTFEY	0,6327
A01	HLA-A*01:03	161	SSANNCTFEY	0,6876
A01	HLA-A*01:03	258	WTAGAAAYY	0,9493
A01	HLA-A*01:03	358	ISNCVADYSVLY	0,6647
A01	HLA-A*01:03	361	CVADYSVLY	0,7732
A01	HLA-A*01:03	603	NTSNQVAVLY	0,8133
A01	HLA-A*01:03	604	TSNQVAVLY	0,9385
A01	HLA-A*01:03	746	STECSNLLLQY	0,8277
A01	HLA-A*01:03	828	LADAGFIKQY	0,8857
A01	HLA-A*01:03	863	PLLTDEMIAQY	0,7915
A01	HLA-A*01:03	865	LTDEMIAQY	0,9982
A01	HLA-A*01:03	1197	LIDLQELGKY	0,8476
A01	HLA-A*01:06	28	YTNSFTRGVY	0,7602
A01	HLA-A*01:06	28	YTNSFTRGVYY	0,7892
A01	HLA-A*01:06	29	TNSFTRGVYY	0,5721
A01	HLA-A*01:06	30	NSFTRGVYY	0,8159
A01	HLA-A*01:06	160	YSSANNCTFEY	0,5052
A01	HLA-A*01:06	161	SSANNCTFEY	0,7022
A01	HLA-A*01:06	162	SANNCTFEY	0,6641
A01	HLA-A*01:06	256	SGWTAGAAAYY	0,6894
A01	HLA-A*01:06	257	GWTAGAAAYY	0,7487
A01	HLA-A*01:06	258	WTAGAAAYY	0,9282
A01	HLA-A*01:06	358	ISNCVADYSVLY	0,607

A01	HLA-A*01:06	359	SNCVADYSVLY	0,5742
A01	HLA-A*01:06	360	NCVADYSVLY	0,6328
A01	HLA-A*01:06	361	CVADYSVLY	0,8242
A01	HLA-A*01:06	601	GTNTSNQVAVLY	0,5577
A01	HLA-A*01:06	603	NTSNQVAVLY	0,7856
A01	HLA-A*01:06	604	TSNQVAVLY	0,9241
A01	HLA-A*01:06	733	KTSVDCTMY	0,6247
A01	HLA-A*01:06	746	STECSNLLLQY	0,5958
A01	HLA-A*01:06	828	LADAGFIKQY	0,7349
A01	HLA-A*01:06	865	LTDEMIAQY	0,9873
A01	HLA-A*01:06	1197	LIDLQELGKY	0,6364
A01	HLA-A*01:07	28	YTNSFTRGVY	0,7994
A01	HLA-A*01:07	28	YTNSFTRGVYY	0,7935
A01	HLA-A*01:07	135	FCNDPFLGVY	0,5269
A01	HLA-A*01:07	136	CNDPFLGVY	0,5497
A01	HLA-A*01:07	136	CNDPFLGVYY	0,5021
A01	HLA-A*01:07	160	YSSANNCTFEY	0,6031
A01	HLA-A*01:07	161	SSANNCTFEY	0,7204
A01	HLA-A*01:07	162	SANNCTFEY	0,6395
A01	HLA-A*01:07	256	SGWTAGAAAYY	0,6935
A01	HLA-A*01:07	257	GWTAGAAAYY	0,7199
A01	HLA-A*01:07	258	WTAGAAAYY	0,9322
A01	HLA-A*01:07	358	ISNCVADYSVLY	0,6325
A01	HLA-A*01:07	361	CVADYSVLY	0,7815
A01	HLA-A*01:07	440	NLDSKVGGNY	0,8818
A01	HLA-A*01:07	603	NTSNQVAVLY	0,8153
A01	HLA-A*01:07	604	TSNQVAVLY	0,9147
A01	HLA-A*01:07	746	STECSNLLLQY	0,728
A01	HLA-A*01:07	828	LADAGFIKQY	0,8525
A01	HLA-A*01:07	863	PLLTDEMIAQY	0,7608
A01	HLA-A*01:07	865	LTDEMIAQY	0,9944
A01	HLA-A*01:07	1197	LIDLQELGKY	0,8505
A01	HLA-A*01:08	28	YTNSFTRGVY	0,8357
A01	HLA-A*01:08	28	YTNSFTRGVYY	0,8794
A01	HLA-A*01:08	135	FCNDPFLGVY	0,5792
A01	HLA-A*01:08	136	CNDPFLGVY	0,5738
A01	HLA-A*01:08	136	CNDPFLGVYY	0,5287
A01	HLA-A*01:08	160	YSSANNCTFEY	0,71
A01	HLA-A*01:08	161	SSANNCTFEY	0,7846
A01	HLA-A*01:08	162	SANNCTFEY	0,6944
A01	HLA-A*01:08	254	SSSGWTAGAAAY	0,5093
A01	HLA-A*01:08	256	SGWTAGAAAYY	0,748
A01	HLA-A*01:08	257	GWTAGAAAYY	0,7602
A01	HLA-A*01:08	258	WTAGAAAYY	0,9628
A01	HLA-A*01:08	358	ISNCVADYSVLY	0,7216
A01	HLA-A*01:08	361	CVADYSVLY	0,8161
A01	HLA-A*01:08	362	VADYSVLY	0,7907
A01	HLA-A*01:08	603	NTSNQVAVLY	0,8442
A01	HLA-A*01:08	604	TSNQVAVLY	0,9539
A01	HLA-A*01:08	697	MSLGAENSVAY	0,5924

A01	HLA-A*01:08	733	KTSVDCTMY	0,6742
A01	HLA-A*01:08	746	STECSNLLLQY	0,8198
A01	HLA-A*01:08	828	LADAGFIKQY	0,9
A01	HLA-A*01:08	863	PLLTDEMIAQY	0,7815
A01	HLA-A*01:08	865	LTDEMIAQY	0,9976
A01	HLA-A*01:08	866	TDEMIAQY	0,8381
A01	HLA-A*01:08	1197	LIDLQELGKY	0,8632
A01	HLA-A*01:09	28	YTNSFTRGVY	0,8696
A01	HLA-A*01:09	28	YTNSFTRGVYY	0,9035
A01	HLA-A*01:09	135	FCNDPFLGVY	0,6253
A01	HLA-A*01:09	136	CNDPFLGVY	0,6408
A01	HLA-A*01:09	136	CNDPFLGVYY	0,6059
A01	HLA-A*01:09	160	YSSANNCTFEY	0,7273
A01	HLA-A*01:09	161	SSANNCTFEY	0,7703
A01	HLA-A*01:09	162	SANNCTFEY	0,6674
A01	HLA-A*01:09	256	SGWTAGAAAYY	0,7466
A01	HLA-A*01:09	257	GWTAGAAAYY	0,7407
A01	HLA-A*01:09	258	WTAGAAAYY	0,9622
A01	HLA-A*01:09	358	ISNCVADYSVLY	0,7628
A01	HLA-A*01:09	361	CVADYSVLY	0,8233
A01	HLA-A*01:09	362	VADYSVLY	0,8361
A01	HLA-A*01:09	370	NSASFSTFKCY	0,5177
A01	HLA-A*01:09	440	NLDSKVGGNY	0,893
A01	HLA-A*01:09	603	NTSNQVAVLY	0,8721
A01	HLA-A*01:09	604	TSNQVAVLY	0,9585
A01	HLA-A*01:09	746	STECSNLLLQY	0,8875
A01	HLA-A*01:09	828	LADAGFIKQY	0,928
A01	HLA-A*01:09	863	PLLTDEMIAQY	0,8726
A01	HLA-A*01:09	865	LTDEMIAQY	0,9987
A01	HLA-A*01:09	866	TDEMIAQY	0,8997
A01	HLA-A*01:09	1197	LIDLQELGKY	0,9031
A01	HLA-A*01:10	28	YTNSFTRGVY	0,8782
A01	HLA-A*01:10	28	YTNSFTRGVYY	0,8568
A01	HLA-A*01:10	135	FCNDPFLGVY	0,5464
A01	HLA-A*01:10	136	CNDPFLGVY	0,55
A01	HLA-A*01:10	160	YSSANNCTFEY	0,617
A01	HLA-A*01:10	161	SSANNCTFEY	0,7739
A01	HLA-A*01:10	162	SANNCTFEY	0,7209
A01	HLA-A*01:10	254	SSSGWTAGAAAY	0,5306
A01	HLA-A*01:10	256	SGWTAGAAAYY	0,7805
A01	HLA-A*01:10	257	GWTAGAAAYY	0,801
A01	HLA-A*01:10	258	WTAGAAAYY	0,9558
A01	HLA-A*01:10	269	YLQPRTFLLKY	0,5653
A01	HLA-A*01:10	358	ISNCVADYSVLY	0,7038
A01	HLA-A*01:10	361	CVADYSVLY	0,8467
A01	HLA-A*01:10	362	VADYSVLY	0,7818
A01	HLA-A*01:10	414	QTGKIADYNY	0,7033
A01	HLA-A*01:10	440	NLDSKVGGNY	0,8551
A01	HLA-A*01:10	601	GTNTSNQVAVLY	0,6737
A01	HLA-A*01:10	603	NTSNQVAVLY	0,8545

A01	HLA-A*01:10	604	TSNQVAVLY	0,9462
A01	HLA-A*01:10	733	KTSVDCTMY	0,7195
A01	HLA-A*01:10	746	STECSNLLLQY	0,8093
A01	HLA-A*01:10	828	LADAGFIKQY	0,8825
A01	HLA-A*01:10	863	PLLTDEMIAQY	0,6841
A01	HLA-A*01:10	865	LTDEMIAQY	0,996
A01	HLA-A*01:10	1039	RVDFCGKGY	0,8273
A01	HLA-A*01:10	1197	LIDLQELGKY	0,875
A01	HLA-A*01:12	28	YTNSFTRGVY	0,5329
A01	HLA-A*01:12	28	YTNSFTRGVYY	0,528
A01	HLA-A*01:12	161	SSANNCTFEY	0,5055
A01	HLA-A*01:12	162	SANNCTFEY	0,5654
A01	HLA-A*01:12	258	WTAGAAAYY	0,8102
A01	HLA-A*01:12	361	CVADYSVLY	0,7278
A01	HLA-A*01:12	444	KVGGNYNYLY	0,594
A01	HLA-A*01:12	603	NTSNQVAVLY	0,5927
A01	HLA-A*01:12	604	TSNQVAVLY	0,7812
A01	HLA-A*01:12	865	LTDEMIAQY	0,9207
A01	HLA-A*01:14	28	YTNSFTRGVY	0,8208
A01	HLA-A*01:14	28	YTNSFTRGVYY	0,8729
A01	HLA-A*01:14	135	FCNDPFLGVY	0,6119
A01	HLA-A*01:14	136	CNDPFLGVY	0,6715
A01	HLA-A*01:14	136	CNDPFLGVYY	0,6088
A01	HLA-A*01:14	160	YSSANNCTFEY	0,663
A01	HLA-A*01:14	161	SSANNCTFEY	0,7519
A01	HLA-A*01:14	162	SANNCTFEY	0,6665
A01	HLA-A*01:14	256	SGWTAGAAAYY	0,6944
A01	HLA-A*01:14	257	GWTAGAAAYY	0,7187
A01	HLA-A*01:14	258	WTAGAAAYY	0,9506
A01	HLA-A*01:14	269	YLQPRTFLLKY	0,5565
A01	HLA-A*01:14	358	ISNCVADYSVLY	0,7087
A01	HLA-A*01:14	361	CVADYSVLY	0,8057
A01	HLA-A*01:14	362	VADYSVLY	0,7998
A01	HLA-A*01:14	601	GTNTSNQVAVLY	0,6797
A01	HLA-A*01:14	603	NTSNQVAVLY	0,8237
A01	HLA-A*01:14	604	TSNQVAVLY	0,9452
A01	HLA-A*01:14	733	KTSVDCTMY	0,7034
A01	HLA-A*01:14	746	STECSNLLLQY	0,8081
A01	HLA-A*01:14	828	LADAGFIKQY	0,8737
A01	HLA-A*01:14	863	PLLTDEMIAQY	0,7423
A01	HLA-A*01:14	865	LTDEMIAQY	0,9971
A01	HLA-A*01:14	1039	RVDFCGKGY	0,8252
A01	HLA-A*01:14	1197	LIDLQELGKY	0,8561
A01	HLA-A*26:01	28	YTNSFTRGVY	0,5472
A01	HLA-A*26:01	191	EFVFKNIDGY	0,8157
A01	HLA-A*26:01	192	FVFKNIDGY	0,9119
A01	HLA-A*26:01	258	WTAGAAAYY	0,9338
A01	HLA-A*26:01	340	EVFNATRFASVY	0,6393
A01	HLA-A*26:01	360	NCVADYSVLY	0,7886
A01	HLA-A*26:01	361	CVADYSVLY	0,8578

A01	HLA-A*26:01	603	NTSNQVAVLY	0,5793
A01	HLA-A*26:01	686	SVASQSIIAY	0,6831
A01	HLA-A*26:01	780	EVFAQVKQIY	0,7706
A01	HLA-A*26:01	1095	FVSNGTHWF	0,5675
A01	HLA-A*26:02	28	YTNSFTRGVY	0,7649
A01	HLA-A*26:02	30	NSFTRGVYY	0,7196
A01	HLA-A*26:02	50	STQDLFLPF	0,7114
A01	HLA-A*26:02	125	NVVIKVCEF	0,6379
A01	HLA-A*26:02	191	EFVFKNIDGY	0,9206
A01	HLA-A*26:02	192	FVFKNIDGY	0,9721
A01	HLA-A*26:02	192	FVFKNIDGYF	0,7022
A01	HLA-A*26:02	215	DLPQGFSAL	0,5481
A01	HLA-A*26:02	256	SGWTAGAAAYY	0,5903
A01	HLA-A*26:02	257	GWTAGAAAYY	0,6462
A01	HLA-A*26:02	258	WTAGAAAY	0,5731
A01	HLA-A*26:02	258	WTAGAAAYY	0,9678
A01	HLA-A*26:02	261	GAAAYYVGY	0,6857
A01	HLA-A*26:02	298	ETKCTLKSF	0,7757
A01	HLA-A*26:02	340	EVFNATRF	0,5818
A01	HLA-A*26:02	340	EVFNATRFASVY	0,834
A01	HLA-A*26:02	343	NATRFASVY	0,526
A01	HLA-A*26:02	359	SNCVADYSVLY	0,6372
A01	HLA-A*26:02	360	NCVADYSVLY	0,9187
A01	HLA-A*26:02	361	CVADYSVLY	0,9515
A01	HLA-A*26:02	366	SVLYNSASF	0,6928
A01	HLA-A*26:02	392	FTNVYADSF	0,6269
A01	HLA-A*26:02	442	DSKVGGNYNY	0,6561
A01	HLA-A*26:02	554	ESNKKFLPF	0,6456
A01	HLA-A*26:02	568	DIADTTDAV	0,5453
A01	HLA-A*26:02	583	EILDITPCSF	0,8274
A01	HLA-A*26:02	603	NTSNQVAVL	0,5594
A01	HLA-A*26:02	603	NTSNQVAVLY	0,7784
A01	HLA-A*26:02	686	SVASQSIIAY	0,8793
A01	HLA-A*26:02	691	SIIAYTMSL	0,7185
A01	HLA-A*26:02	710	NSIAIPTNF	0,7355
A01	HLA-A*26:02	718	FTISVTTEI	0,7944
A01	HLA-A*26:02	780	EVFAQVKQI	0,672
A01	HLA-A*26:02	780	EVFAQVKQIY	0,8897
A01	HLA-A*26:02	865	LTDEMIAQY	0,7697
A01	HLA-A*26:02	869	MIAQYTSAL	0,7428
A01	HLA-A*26:02	880	GTITSGWTF	0,5972
A01	HLA-A*26:02	886	WTFGAGAAL	0,7617
A01	HLA-A*26:02	898	FAMQMAYRF	0,5082
A01	HLA-A*26:02	940	STASALGKL	0,6677
A01	HLA-A*26:02	962	LVKQLSSNF	0,536
A01	HLA-A*26:02	1054	QSAPHGVVF	0,6027
A01	HLA-A*26:02	1095	FVSNGTHWF	0,8644
A01	HLA-A*26:02	1113	QIITTDNTF	0,6231
A01	HLA-A*26:02	1168	DISGINASV	0,5184
A01	HLA-A*26:02	1188	EVAKNLNESL	0,5001

A01	HLA-A*26:03	192	FVFKNIDGY	0,7319
A01	HLA-A*26:03	258	WTAGAAAYY	0,746
A01	HLA-A*26:04	28	YTNSFTRGVY	0,5132
A01	HLA-A*26:04	191	EFVFKNIDGY	0,6376
A01	HLA-A*26:04	192	FVFKNIDGY	0,853
A01	HLA-A*26:04	258	WTAGAAAYY	0,8788
A01	HLA-A*26:04	360	NCVADYSVLY	0,6391
A01	HLA-A*26:04	361	CVADYSVLY	0,75
A01	HLA-A*26:04	686	SVASQSIIAY	0,5849
A01	HLA-A*26:04	780	EVFAQVKQIY	0,6193
A01	HLA-A*26:05	28	YTNSFTRGVY	0,5354
A01	HLA-A*26:05	191	EFVFKNIDGY	0,7735
A01	HLA-A*26:05	192	FVFKNIDGY	0,8748
A01	HLA-A*26:05	258	WTAGAAAYY	0,9178
A01	HLA-A*26:05	340	EVFNATRFASVY	0,5592
A01	HLA-A*26:05	360	NCVADYSVLY	0,7308
A01	HLA-A*26:05	361	CVADYSVLY	0,7925
A01	HLA-A*26:05	603	NTSNQVAVLY	0,5637
A01	HLA-A*26:05	686	SVASQSIIAY	0,6565
A01	HLA-A*26:05	780	EVFAQVKQIY	0,7142
A01	HLA-A*26:06	192	FVFKNIDGY	0,7319
A01	HLA-A*26:06	258	WTAGAAAYY	0,746
A01	HLA-A*26:07	28	YTNSFTRGVY	0,5921
A01	HLA-A*26:07	50	STQDLFLPF	0,531
A01	HLA-A*26:07	192	FVFKNIDGY	0,929
A01	HLA-A*26:07	257	GWTAGAAAYY	0,6545
A01	HLA-A*26:07	258	WTAGAAAYY	0,8842
A01	HLA-A*26:07	261	GAAAYYVGY	0,7223
A01	HLA-A*26:07	360	NCVADYSVLY	0,557
A01	HLA-A*26:07	361	CVADYSVLY	0,7863
A01	HLA-A*26:07	366	SVLYNSASF	0,546
A01	HLA-A*26:07	686	SVASQSIIAY	0,7678
A01	HLA-A*26:07	691	SIIAYTMSL	0,5743
A01	HLA-A*26:07	698	SLGAENSVAY	0,5027
A01	HLA-A*26:07	718	FTISVTTEI	0,5245
A01	HLA-A*26:07	864	LLTDEMIAQY	0,554
A01	HLA-A*26:07	865	LTDEMIAQY	0,7389
A01	HLA-A*26:07	880	GTITSGWTF	0,6723
A01	HLA-A*26:07	976	VLNDILSRL	0,5945
A01	HLA-A*26:07	1095	FVSNGTHWF	0,7015
A01	HLA-A*26:07	1196	SLIDLQELGKY	0,516
A01	HLA-A*26:08	28	YTNSFTRGVY	0,5682
A01	HLA-A*26:08	50	STQDLFLPF	0,5601
A01	HLA-A*26:08	191	EFVFKNIDGY	0,6318
A01	HLA-A*26:08	192	FVFKNIDGY	0,8385
A01	HLA-A*26:08	258	WTAGAAAYY	0,9025
A01	HLA-A*26:08	340	EVFNATRFASVY	0,5328
A01	HLA-A*26:08	360	NCVADYSVLY	0,766
A01	HLA-A*26:08	361	CVADYSVLY	0,819
A01	HLA-A*26:08	603	NTSNQVAVLY	0,5863

A01	HLA-A*26:08	686	SVASQSIIAY	0,7121
A01	HLA-A*26:08	780	EVFAQVKQIY	0,596
A01	HLA-A*26:08	865	LTDEMIAQY	0,7329
A01	HLA-A*26:08	1095	FVSNGTHWF	0,5998
A01	HLA-A*26:09	28	YTNSFTRGVY	0,6479
A01	HLA-A*26:09	191	EFVFKNIDGY	0,751
A01	HLA-A*26:09	192	FVFKNIDGY	0,9239
A01	HLA-A*26:09	257	GWTAGAAAYY	0,5288
A01	HLA-A*26:09	258	WTAGAAAYY	0,9326
A01	HLA-A*26:09	298	ETKCTLKSF	0,5067
A01	HLA-A*26:09	340	EVFNATRFASVY	0,6007
A01	HLA-A*26:09	360	NCVADYSVLY	0,7668
A01	HLA-A*26:09	361	CVADYSVLY	0,8649
A01	HLA-A*26:09	603	NTSNQVAVLY	0,611
A01	HLA-A*26:09	686	SVASQSIIAY	0,7378
A01	HLA-A*26:09	780	EVFAQVKQIY	0,7512
A01	HLA-A*26:09	1095	FVSNGTHWF	0,6488
A01	HLA-A*26:10	28	YTNSFTRGVY	0,5472
A01	HLA-A*26:10	191	EFVFKNIDGY	0,8157
A01	HLA-A*26:10	192	FVFKNIDGY	0,9119
A01	HLA-A*26:10	258	WTAGAAAYY	0,9338
A01	HLA-A*26:10	340	EVFNATRFASVY	0,6393
A01	HLA-A*26:10	360	NCVADYSVLY	0,7886
A01	HLA-A*26:10	361	CVADYSVLY	0,8578
A01	HLA-A*26:10	603	NTSNQVAVLY	0,5793
A01	HLA-A*26:10	686	SVASQSIIAY	0,6831
A01	HLA-A*26:10	780	EVFAQVKQIY	0,7706
A01	HLA-A*26:10	1095	FVSNGTHWF	0,5675
A01	HLA-A*26:12	191	EFVFKNIDGY	0,6509
A01	HLA-A*26:12	192	FVFKNIDGY	0,8495
A01	HLA-A*26:12	258	WTAGAAAYY	0,8859
A01	HLA-A*26:12	360	NCVADYSVLY	0,7498
A01	HLA-A*26:12	361	CVADYSVLY	0,7826
A01	HLA-A*26:12	603	NTSNQVAVLY	0,5023
A01	HLA-A*26:12	686	SVASQSIIAY	0,5107
A01	HLA-A*26:12	780	EVFAQVKQIY	0,5545
A01	HLA-A*26:12	1095	FVSNGTHWF	0,5181
A01	HLA-A*26:13	28	YTNSFTRGVY	0,5142
A01	HLA-A*26:13	191	EFVFKNIDGY	0,7343
A01	HLA-A*26:13	192	FVFKNIDGY	0,8696
A01	HLA-A*26:13	258	WTAGAAAYY	0,9172
A01	HLA-A*26:13	340	EVFNATRFASVY	0,5729
A01	HLA-A*26:13	360	NCVADYSVLY	0,7292
A01	HLA-A*26:13	361	CVADYSVLY	0,794
A01	HLA-A*26:13	603	NTSNQVAVLY	0,561
A01	HLA-A*26:13	686	SVASQSIIAY	0,6258
A01	HLA-A*26:13	780	EVFAQVKQIY	0,7104
A01	HLA-A*26:13	886	WTFGAGAAL	0,5071
A01	HLA-A*26:13	1095	FVSNGTHWF	0,5038
A01	HLA-A*26:14	28	YTNSFTRGVY	0,5472

A01	HLA-A*26:14	191	EFVFKNIDGY	0,8157
A01	HLA-A*26:14	192	FVFKNIDGY	0,9119
A01	HLA-A*26:14	258	WTAGAAAYY	0,9338
A01	HLA-A*26:14	340	EVFNATRFASVY	0,6393
A01	HLA-A*26:14	360	NCVADYSVLY	0,7886
A01	HLA-A*26:14	361	CVADYSVLY	0,8578
A01	HLA-A*26:14	603	NTSNQVAVLY	0,5793
A01	HLA-A*26:14	686	SVASQSIIAY	0,6831
A01	HLA-A*26:14	780	EVFAQVKQIY	0,7706
A01	HLA-A*26:14	1095	FVSNGTHWF	0,5675
A01	HLA-A*26:15	28	YTNSFTRGVY	0,5472
A01	HLA-A*26:15	191	EFVFKNIDGY	0,8157
A01	HLA-A*26:15	192	FVFKNIDGY	0,9119
A01	HLA-A*26:15	258	WTAGAAAYY	0,9338
A01	HLA-A*26:15	340	EVFNATRFASVY	0,6393
A01	HLA-A*26:15	360	NCVADYSVLY	0,7886
A01	HLA-A*26:15	361	CVADYSVLY	0,8578
A01	HLA-A*26:15	603	NTSNQVAVLY	0,5793
A01	HLA-A*26:15	686	SVASQSIIAY	0,6831
A01	HLA-A*26:15	780	EVFAQVKQIY	0,7706
A01	HLA-A*26:15	1095	FVSNGTHWF	0,5675
A01	HLA-A*26:17	28	YTNSFTRGVY	0,5472
A01	HLA-A*26:17	191	EFVFKNIDGY	0,8157
A01	HLA-A*26:17	192	FVFKNIDGY	0,9119
A01	HLA-A*26:17	258	WTAGAAAYY	0,9338
A01	HLA-A*26:17	340	EVFNATRFASVY	0,6393
A01	HLA-A*26:17	360	NCVADYSVLY	0,7886
A01	HLA-A*26:17	361	CVADYSVLY	0,8578
A01	HLA-A*26:17	603	NTSNQVAVLY	0,5793
A01	HLA-A*26:17	686	SVASQSIIAY	0,6831
A01	HLA-A*26:17	780	EVFAQVKQIY	0,7706
A01	HLA-A*26:17	1095	FVSNGTHWF	0,5675
A01	HLA-A*26:18	191	EFVFKNIDGY	0,6509
A01	HLA-A*26:18	192	FVFKNIDGY	0,8495
A01	HLA-A*26:18	258	WTAGAAAYY	0,8859
A01	HLA-A*26:18	360	NCVADYSVLY	0,7498
A01	HLA-A*26:18	361	CVADYSVLY	0,7826
A01	HLA-A*26:18	603	NTSNQVAVLY	0,5023
A01	HLA-A*26:18	686	SVASQSIIAY	0,5107
A01	HLA-A*26:18	780	EVFAQVKQIY	0,5545
A01	HLA-A*26:18	1095	FVSNGTHWF	0,5181
A01	HLA-A*26:19	28	YTNSFTRGVY	0,7228
A01	HLA-A*26:19	28	YTNSFTRGVYY	0,5399
A01	HLA-A*26:19	30	NSFTRGVYY	0,7407
A01	HLA-A*26:19	50	STQDLFLPF	0,6401
A01	HLA-A*26:19	161	SSANNCTFEY	0,5341
A01	HLA-A*26:19	162	SANNCTFEY	0,5571
A01	HLA-A*26:19	192	FVFKNIDGY	0,8978
A01	HLA-A*26:19	256	SGWTAGAAAYY	0,6687
A01	HLA-A*26:19	257	GWTAGAAAYY	0,8485

A01	HLA-A*26:19	258	WTAGAAAYY	0,9258
A01	HLA-A*26:19	261	GAAAYYVGY	0,8482
A01	HLA-A*26:19	359	SNCVADYSVLY	0,5535
A01	HLA-A*26:19	360	NCVADYSVLY	0,613
A01	HLA-A*26:19	361	CVADYSVLY	0,8311
A01	HLA-A*26:19	366	SVLYNSASF	0,5818
A01	HLA-A*26:19	372	ASFSTFKCY	0,5748
A01	HLA-A*26:19	603	NTSNQVAVLY	0,5876
A01	HLA-A*26:19	604	TSNQVAVLY	0,8069
A01	HLA-A*26:19	634	RVYSTGSNVF	0,5845
A01	HLA-A*26:19	686	SVASQSIIAY	0,8445
A01	HLA-A*26:19	687	VASQSIIAY	0,7283
A01	HLA-A*26:19	691	SIIAYTMSL	0,5489
A01	HLA-A*26:19	733	KTSVDCTMY	0,5057
A01	HLA-A*26:19	865	LTDEMIAQY	0,7578
A01	HLA-A*26:19	880	GTITSGWTF	0,7351
A01	HLA-A*26:19	940	STASALGKL	0,5468
A01	HLA-A*26:19	1054	QSAPHGVVF	0,5969
A01	HLA-A*26:19	1059	GVVFLHVTY	0,6507
A01	HLA-A*26:19	1095	FVSNGTHWF	0,5261
A01	HLA-A*26:19	1128	VVIGIVNNTVY	0,5268
A01	HLA-A*26:21	192	FVFKNIDGY	0,7319
A01	HLA-A*26:21	258	WTAGAAAYY	0,746
A01	HLA-A*26:23	28	YTNSFTRGVY	0,5472
A01	HLA-A*26:23	191	EFVFKNIDGY	0,8157
A01	HLA-A*26:23	192	FVFKNIDGY	0,9119
A01	HLA-A*26:23	258	WTAGAAAYY	0,9338
A01	HLA-A*26:23	340	EVFNATRFASVY	0,6393
A01	HLA-A*26:23	360	NCVADYSVLY	0,7886
A01	HLA-A*26:23	361	CVADYSVLY	0,8578
A01	HLA-A*26:23	603	NTSNQVAVLY	0,5793
A01	HLA-A*26:23	686	SVASQSIIAY	0,6831
A01	HLA-A*26:23	780	EVFAQVKQIY	0,7706
A01	HLA-A*26:23	1095	FVSNGTHWF	0,5675
A01	HLA-A*26:24	28	YTNSFTRGVY	0,5472
A01	HLA-A*26:24	191	EFVFKNIDGY	0,8157
A01	HLA-A*26:24	192	FVFKNIDGY	0,9119
A01	HLA-A*26:24	258	WTAGAAAYY	0,9338
A01	HLA-A*26:24	340	EVFNATRFASVY	0,6393
A01	HLA-A*26:24	360	NCVADYSVLY	0,7886
A01	HLA-A*26:24	361	CVADYSVLY	0,8578
A01	HLA-A*26:24	603	NTSNQVAVLY	0,5793
A01	HLA-A*26:24	686	SVASQSIIAY	0,6831
A01	HLA-A*26:24	780	EVFAQVKQIY	0,7706
A01	HLA-A*26:24	1095	FVSNGTHWF	0,5675
A01	HLA-A*26:26	28	YTNSFTRGVY	0,5472
A01	HLA-A*26:26	191	EFVFKNIDGY	0,8157
A01	HLA-A*26:26	192	FVFKNIDGY	0,9119
A01	HLA-A*26:26	258	WTAGAAAYY	0,9338
A01	HLA-A*26:26	340	EVFNATRFASVY	0,6393

A01	HLA-A*26:26	360	NCVADYSVLY	0,7886
A01	HLA-A*26:26	361	CVADYSVLY	0,8578
A01	HLA-A*26:26	603	NTSNQVAVLY	0,5793
A01	HLA-A*26:26	686	SVASQSIIAY	0,6831
A01	HLA-A*26:26	780	EVFAQVKQIY	0,7706
A01	HLA-A*26:26	1095	FVSNGTHWF	0,5675
A01	HLA-A*30:02	28	YTNSFTRGVY	0,5478
A01	HLA-A*30:02	30	NSFTRGVYY	0,6841
A01	HLA-A*30:02	161	SSANNCTFEY	0,5282
A01	HLA-A*30:02	162	SANNCTFEY	0,5717
A01	HLA-A*30:02	192	FVFKNIDGY	0,5825
A01	HLA-A*30:02	195	KNIDGYFKIY	0,5462
A01	HLA-A*30:02	240	TLLALHRSY	0,5237
A01	HLA-A*30:02	257	GWTAGAAAYY	0,6876
A01	HLA-A*30:02	258	WTAGAAAYY	0,7568
A01	HLA-A*30:02	261	GAAAYYVGY	0,6441
A01	HLA-A*30:02	304	KSFTVEKGIY	0,5482
A01	HLA-A*30:02	357	RISNCVADY	0,5878
A01	HLA-A*30:02	361	CVADYSVLY	0,5824
A01	HLA-A*30:02	372	ASFSTFKCY	0,5884
A01	HLA-A*30:02	413	GQTGKIADY	0,581
A01	HLA-A*30:02	444	KVGGNYNYLY	0,7271
A01	HLA-A*30:02	445	VGGNYNYLY	0,5772
A01	HLA-A*30:02	496	GFQPTNGVGY	0,5657
A01	HLA-A*30:02	604	TSNQVAVLY	0,8094
A01	HLA-A*30:02	628	QLTPTWRVY	0,5588
A01	HLA-A*30:02	666	IGAGICASY	0,6063
A01	HLA-A*30:02	686	SVASQSIIAY	0,5074
A01	HLA-A*30:02	687	VASQSIIAY	0,6482
A01	HLA-A*30:02	733	KTSVDCTMY	0,6807
A01	HLA-A*30:02	781	VFAQVKQIY	0,6982
A01	HLA-A*30:02	865	LTDEMIAQY	0,69
A01	HLA-A*30:02	1039	RVDFCGKGY	0,6059
A01	HLA-A*30:02	1264	VLKGVKLHY	0,8117
A01	HLA-A*30:03	28	YTNSFTRGVY	0,5478
A01	HLA-A*30:03	30	NSFTRGVYY	0,6841
A01	HLA-A*30:03	161	SSANNCTFEY	0,5282
A01	HLA-A*30:03	162	SANNCTFEY	0,5717
A01	HLA-A*30:03	192	FVFKNIDGY	0,5825
A01	HLA-A*30:03	195	KNIDGYFKIY	0,5462
A01	HLA-A*30:03	240	TLLALHRSY	0,5237
A01	HLA-A*30:03	257	GWTAGAAAYY	0,6876
A01	HLA-A*30:03	258	WTAGAAAYY	0,7568
A01	HLA-A*30:03	261	GAAAYYVGY	0,6441
A01	HLA-A*30:03	304	KSFTVEKGIY	0,5482
A01	HLA-A*30:03	357	RISNCVADY	0,5878
A01	HLA-A*30:03	361	CVADYSVLY	0,5824
A01	HLA-A*30:03	372	ASFSTFKCY	0,5884
A01	HLA-A*30:03	413	GQTGKIADY	0,581
A01	HLA-A*30:03	444	KVGGNYNYLY	0,7271

A01	HLA-A*30:03	445	VGGNYNYLY	0,5772
A01	HLA-A*30:03	496	GFQPTNGVGY	0,5657
A01	HLA-A*30:03	604	TSNQVAVLY	0,8094
A01	HLA-A*30:03	628	QLTPTWRVY	0,5588
A01	HLA-A*30:03	666	IGAGICASY	0,6063
A01	HLA-A*30:03	686	SVASQSIIAY	0,5074
A01	HLA-A*30:03	687	VASQSIIAY	0,6482
A01	HLA-A*30:03	733	KTSVDCTMY	0,6807
A01	HLA-A*30:03	781	VFAQVKQIY	0,6982
A01	HLA-A*30:03	865	LTDEMIAQY	0,69
A01	HLA-A*30:03	1039	RVDFCGKGY	0,6059
A01	HLA-A*30:03	1264	VLKGVKLHY	0,8117
A01	HLA-A*30:04	257	GWTAGAAAYY	0,5598
A01	HLA-A*30:04	258	WTAGAAAYY	0,6721
A01	HLA-A*30:04	261	GAAAYYVGY	0,5257
A01	HLA-A*30:04	444	KVGGNYNYLY	0,5264
A01	HLA-A*30:04	445	VGGNYNYLY	0,512
A01	HLA-A*30:04	604	TSNQVAVLY	0,6629
A01	HLA-A*30:06	257	GWTAGAAAYY	0,5598
A01	HLA-A*30:06	258	WTAGAAAYY	0,6721
A01	HLA-A*30:06	261	GAAAYYVGY	0,5257
A01	HLA-A*30:06	444	KVGGNYNYLY	0,5264
A01	HLA-A*30:06	445	VGGNYNYLY	0,512
A01	HLA-A*30:06	604	TSNQVAVLY	0,6629
A01	HLA-A*30:09	30	NSFTRGVYY	0,5993
A01	HLA-A*30:09	162	SANNCTFEY	0,5586
A01	HLA-A*30:09	195	KNIDGYFKIY	0,5437
A01	HLA-A*30:09	257	GWTAGAAAYY	0,5637
A01	HLA-A*30:09	258	WTAGAAAYY	0,667
A01	HLA-A*30:09	261	GAAAYYVGY	0,6001
A01	HLA-A*30:09	361	CVADYSVLY	0,5285
A01	HLA-A*30:09	444	KVGGNYNYLY	0,6407
A01	HLA-A*30:09	445	VGGNYNYLY	0,6713
A01	HLA-A*30:09	604	TSNQVAVLY	0,7365
A01	HLA-A*30:09	666	IGAGICASY	0,5459
A01	HLA-A*30:09	733	KTSVDCTMY	0,5356
A01	HLA-A*30:09	781	VFAQVKQIY	0,6655
A01	HLA-A*30:09	865	LTDEMIAQY	0,6628
A01	HLA-A*30:12	28	YTNSFTRGVY	0,5478
A01	HLA-A*30:12	30	NSFTRGVYY	0,6841
A01	HLA-A*30:12	161	SSANNCTFEY	0,5282
A01	HLA-A*30:12	162	SANNCTFEY	0,5717
A01	HLA-A*30:12	192	FVFKNIDGY	0,5825
A01	HLA-A*30:12	195	KNIDGYFKIY	0,5462
A01	HLA-A*30:12	240	TLLALHRSY	0,5237
A01	HLA-A*30:12	257	GWTAGAAAYY	0,6876
A01	HLA-A*30:12	258	WTAGAAAYY	0,7568
A01	HLA-A*30:12	261	GAAAYYVGY	0,6441
A01	HLA-A*30:12	304	KSFTVEKGIY	0,5482
A01	HLA-A*30:12	357	RISNCVADY	0,5878

A01	HLA-A*30:12	361	CVADYSVLY	0,5824
A01	HLA-A*30:12	372	ASFSTFKCY	0,5884
A01	HLA-A*30:12	413	GQTGKIADY	0,581
A01	HLA-A*30:12	444	KVGGNYNYLY	0,7271
A01	HLA-A*30:12	445	VGGNYNYLY	0,5772
A01	HLA-A*30:12	496	GFQPTNGVGY	0,5657
A01	HLA-A*30:12	604	TSNQVAVLY	0,8094
A01	HLA-A*30:12	628	QLTPTWRVY	0,5588
A01	HLA-A*30:12	666	IGAGICASY	0,6063
A01	HLA-A*30:12	686	SVASQSIIAY	0,5074
A01	HLA-A*30:12	687	VASQSIIAY	0,6482
A01	HLA-A*30:12	733	KTSVDCTMY	0,6807
A01	HLA-A*30:12	781	VFAQVKQIY	0,6982
A01	HLA-A*30:12	865	LTDEMIAQY	0,69
A01	HLA-A*30:12	1039	RVDFCGKGY	0,6059
A01	HLA-A*30:12	1264	VLKGVKLHY	0,8117
A01	HLA-A*32:01	366	SVLYNSASF	0,6299
A01	HLA-A*32:01	417	KIADYNYKL	0,8445
A01	HLA-A*32:01	634	RVYSTGSNVF	0,6705
A01	HLA-A*32:01	691	SIIAYTMSL	0,7021
A01	HLA-A*32:01	815	RSFIEDLLF	0,5863
A01	HLA-A*32:01	880	GTITSGWTF	0,7522
A01	HLA-A*32:01	1185	RLNEVAKNL	0,7116
A01	HLA-A*32:02	366	SVLYNSASF	0,5561
A01	HLA-A*32:02	417	KIADYNYKL	0,7698
A01	HLA-A*32:02	634	RVYSTGSNVF	0,5403
A01	HLA-A*32:02	691	SIIAYTMSL	0,62
A01	HLA-A*32:02	880	GTITSGWTF	0,6422
A01	HLA-A*32:02	1185	RLNEVAKNL	0,6529
A01	HLA-A*32:05	366	SVLYNSASF	0,6066
A01	HLA-A*32:05	417	KIADYNYKL	0,7528
A01	HLA-A*32:05	634	RVYSTGSNVF	0,5466
A01	HLA-A*32:05	691	SIIAYTMSL	0,6692
A01	HLA-A*32:05	815	RSFIEDLLF	0,5277
A01	HLA-A*32:05	880	GTITSGWTF	0,7114
A01	HLA-A*32:05	1185	RLNEVAKNL	0,5942
A01	HLA-A*32:06	366	SVLYNSASF	0,6299
A01	HLA-A*32:06	417	KIADYNYKL	0,8445
A01	HLA-A*32:06	634	RVYSTGSNVF	0,6705
A01	HLA-A*32:06	691	SIIAYTMSL	0,7021
A01	HLA-A*32:06	815	RSFIEDLLF	0,5863
A01	HLA-A*32:06	880	GTITSGWTF	0,7522
A01	HLA-A*32:06	1185	RLNEVAKNL	0,7116
A01	HLA-A*32:07	50	STQDLFLPF	0,5832
A01	HLA-A*32:07	77	KRFDNPVLPF	0,6519
A01	HLA-A*32:07	78	RFDNPVLPF	0,7048
A01	HLA-A*32:07	144	YYHKNNKSW	0,6197
A01	HLA-A*32:07	193	VFKNIDGYF	0,6354
A01	HLA-A*32:07	195	KNIDGYFKI	0,501
A01	HLA-A*32:07	261	GAAAYYVGY	0,5142

A01	HLA-A*32:07	267	VGYLQPRTF	0,5924
A01	HLA-A*32:07	268	GYLQPRTFL	0,5759
A01	HLA-A*32:07	310	KGIYQTSNF	0,5492
A01	HLA-A*32:07	345	TRFASVYAW	0,7472
A01	HLA-A*32:07	366	SVLYNSASF	0,7088
A01	HLA-A*32:07	417	KIADYNYKL	0,6173
A01	HLA-A*32:07	557	KKFLPFQQF	0,6339
A01	HLA-A*32:07	634	RVYSTGSNVF	0,6399
A01	HLA-A*32:07	635	VYSTGSNVF	0,566
A01	HLA-A*32:07	689	SQSIIAYTM	0,5018
A01	HLA-A*32:07	710	NSIAIPTNF	0,6477
A01	HLA-A*32:07	781	VFAQVKQIY	0,715
A01	HLA-A*32:07	815	RSFIEDLLF	0,684
A01	HLA-A*32:07	880	GTITSGWTF	0,8141
A01	HLA-A*32:07	1054	QSAPHGVVF	0,6406
A01	HLA-A*32:07	1101	HWFVTQRNF	0,7546
A01	HLA-A*32:07	1208	QYIKWPWYI	0,6932
A01	HLA-A*32:09	50	STQDLFLPF	0,5147
A01	HLA-A*32:09	366	SVLYNSASF	0,6078
A01	HLA-A*32:09	417	KIADYNYKL	0,6951
A01	HLA-A*32:09	691	SIIAYTMSL	0,552
A01	HLA-A*32:09	815	RSFIEDLLF	0,5187
A01	HLA-A*32:09	880	GTITSGWTF	0,7219
A01	HLA-A*32:10	634	RVYSTGSNVF	0,5972
A01	HLA-A*36:01	28	YTNSFTRGVY	0,7119
A01	HLA-A*36:01	28	YTNSFTRGVYY	0,7867
A01	HLA-A*36:01	160	YSSANNCTFEY	0,5391
A01	HLA-A*36:01	161	SSANNCTFEY	0,7073
A01	HLA-A*36:01	162	SANNCTFEY	0,6682
A01	HLA-A*36:01	256	SGWTAGAAAYY	0,667
A01	HLA-A*36:01	257	GWTAGAAAYY	0,7231
A01	HLA-A*36:01	258	WTAGAAAYY	0,8964
A01	HLA-A*36:01	358	ISNCVADYSVLY	0,6026
A01	HLA-A*36:01	361	CVADYSVLY	0,7473
A01	HLA-A*36:01	362	VADYSVLY	0,65
A01	HLA-A*36:01	414	QTGKIADYNY	0,5523
A01	HLA-A*36:01	601	GTNTSNQVAVLY	0,629
A01	HLA-A*36:01	603	NTSNQVAVLY	0,7051
A01	HLA-A*36:01	604	TSNQVAVLY	0,8841
A01	HLA-A*36:01	733	KTSVDCTMY	0,7042
A01	HLA-A*36:01	746	STECSNLLLQY	0,6614
A01	HLA-A*36:01	828	LADAGFIKQY	0,7384
A01	HLA-A*36:01	863	PLLTDEMIAQY	0,5018
A01	HLA-A*36:01	865	LTDEMIAQY	0,9845
A01	HLA-A*36:01	1039	RVDFCGKGY	0,8092
A01	HLA-A*36:01	1197	LIDLQELGKY	0,7101
A01	HLA-A*36:02	28	YTNSFTRGVY	0,5893
A01	HLA-A*36:02	28	YTNSFTRGVYY	0,6541
A01	HLA-A*36:02	161	SSANNCTFEY	0,6078
A01	HLA-A*36:02	162	SANNCTFEY	0,605

A01	HLA-A*36:02	256	SGWTAGAAAYY	0,583
A01	HLA-A*36:02	257	GWTAGAAAYY	0,6743
A01	HLA-A*36:02	258	WTAGAAAYY	0,8378
A01	HLA-A*36:02	361	CVADYSVLY	0,6943
A01	HLA-A*36:02	444	KVGGNYNYLY	0,5293
A01	HLA-A*36:02	601	GTNTSNQVAVLY	0,5171
A01	HLA-A*36:02	603	NTSNQVAVLY	0,5926
A01	HLA-A*36:02	604	TSNQVAVLY	0,8154
A01	HLA-A*36:02	733	KTSVDCTMY	0,6327
A01	HLA-A*36:02	865	LTDEMIAQY	0,95
A01	HLA-A*36:02	1039	RVDFCGKGY	0,7404
A01	HLA-A*36:02	1197	LIDLQELGKY	0,5242
A01	HLA-A*36:04	28	YTNSFTRGVY	0,7271
A01	HLA-A*36:04	28	YTNSFTRGVYY	0,8143
A01	HLA-A*36:04	30	NSFTRGVYY	0,6993
A01	HLA-A*36:04	160	YSSANNCTFEY	0,6118
A01	HLA-A*36:04	161	SSANNCTFEY	0,727
A01	HLA-A*36:04	162	SANNCTFEY	0,6738
A01	HLA-A*36:04	256	SGWTAGAAAYY	0,709
A01	HLA-A*36:04	257	GWTAGAAAYY	0,7396
A01	HLA-A*36:04	258	WTAGAAAYY	0,9128
A01	HLA-A*36:04	358	ISNCVADYSVLY	0,652
A01	HLA-A*36:04	361	CVADYSVLY	0,7656
A01	HLA-A*36:04	362	VADYSVLY	0,6977
A01	HLA-A*36:04	414	QTGKIADYNY	0,5768
A01	HLA-A*36:04	601	GTNTSNQVAVLY	0,6316
A01	HLA-A*36:04	603	NTSNQVAVLY	0,7383
A01	HLA-A*36:04	604	TSNQVAVLY	0,9007
A01	HLA-A*36:04	733	KTSVDCTMY	0,64
A01	HLA-A*36:04	746	STECSNLLLQY	0,7554
A01	HLA-A*36:04	828	LADAGFIKQY	0,7998
A01	HLA-A*36:04	863	PLLTDEMIAQY	0,6402
A01	HLA-A*36:04	865	LTDEMIAQY	0,9936
A01	HLA-A*36:04	1039	RVDFCGKGY	0,7674
A01	HLA-A*36:04	1197	LIDLQELGKY	0,7682
A01 A03	HLA-A*30:01	89	GVYFASTEK	0,524
A01 A03	HLA-A*30:01	142	GVYYHKNNK	0,5185
A01 A03	HLA-A*30:01	302	TLKSFTVEK	0,5401
A01 A03	HLA-A*30:01	344	ATRFASVYA	0,8043
A01 A03	HLA-A*30:01	378	KCYGVSPTK	0,5092
A01 A03	HLA-A*30:01	454	RLFRKSNLK	0,5644
A01 A03	HLA-A*30:01	634	RVYSTGSNV	0,632
A01 A03	HLA-A*30:01	1065	VTYVPAQEK	0,5221
A01 A03	HLA-A*30:08	344	ATRFASVYA	0,6186
A01 A03	HLA-A*30:11	89	GVYFASTEK	0,524
A01 A03	HLA-A*30:11	142	GVYYHKNNK	0,5185
A01 A03	HLA-A*30:11	302	TLKSFTVEK	0,5401
A01 A03	HLA-A*30:11	344	ATRFASVYA	0,8043
A01 A03	HLA-A*30:11	378	KCYGVSPTK	0,5092
A01 A03	HLA-A*30:11	454	RLFRKSNLK	0,5644

A01 A03	HLA-A*30:11	634	RVYSTGSNV	0,632
A01 A03	HLA-A*30:11	1065	VTYVPAQEK	0,5221
A01 A03	HLA-A*30:15	89	GVYFASTEK	0,524
A01 A03	HLA-A*30:15	142	GVYYHKNNK	0,5185
A01 A03	HLA-A*30:15	302	TLKSFTVEK	0,5401
A01 A03	HLA-A*30:15	344	ATRFASVYA	0,8043
A01 A03	HLA-A*30:15	378	KCYGVSPTK	0,5092
A01 A03	HLA-A*30:15	454	RLFRKSNLK	0,5644
A01 A03	HLA-A*30:15	634	RVYSTGSNV	0,632
A01 A03	HLA-A*30:15	1065	VTYVPAQEK	0,5221
A01 A24	HLA-A*29:01	30	NSFTRGVYY	0,825
A01 A24	HLA-A*29:01	151	SWMESEFRVY	0,7114
A01 A24	HLA-A*29:01	162	SANNCTFEY	0,7814
A01 A24	HLA-A*29:01	192	FVFKNIDGY	0,9181
A01 A24	HLA-A*29:01	240	TLLALHRSY	0,6806
A01 A24	HLA-A*29:01	257	GWTAGAAAY	0,5826
A01 A24	HLA-A*29:01	257	GWTAGAAAYY	0,6803
A01 A24	HLA-A*29:01	258	WTAGAAAYY	0,9403
A01 A24	HLA-A*29:01	261	GAAAYYVGY	0,7714
A01 A24	HLA-A*29:01	360	NCVADYSVLY	0,6347
A01 A24	HLA-A*29:01	361	CVADYSVLY	0,9086
A01 A24	HLA-A*29:01	444	KVGGNYNYLY	0,5931
A01 A24	HLA-A*29:01	445	VGGNYNYLY	0,6436
A01 A24	HLA-A*29:01	496	GFQPTNGVGY	0,6858
A01 A24	HLA-A*29:01	604	TSNQVAVLY	0,8854
A01 A24	HLA-A*29:01	628	QLTPTWRVY	0,51
A01 A24	HLA-A*29:01	686	SVASQSIIAY	0,6064
A01 A24	HLA-A*29:01	687	VASQSIIAY	0,6791
A01 A24	HLA-A*29:01	781	VFAQVKQIY	0,9197
A01 A24	HLA-A*29:01	827	TLADAGFIKQY	0,5983
A01 A24	HLA-A*29:01	865	LTDEMIAQY	0,819
A01 A24	HLA-A*29:01	896	IPFAMQMAY	0,5343
A01 A24	HLA-A*29:01	909	IGVTQNVLY	0,5165
A01 A24	HLA-A*29:01	1059	GVVFLHVTY	0,7218
A01 A24	HLA-A*29:01	1102	WFVTQRNFY	0,7439
A01 A24	HLA-A*29:01	1147	SFKEELDKY	0,6923
A01 A24	HLA-A*29:01	1264	VLKGVKLHY	0,8598
A01 A24	HLA-A*29:02	30	NSFTRGVYY	0,825
A01 A24	HLA-A*29:02	151	SWMESEFRVY	0,7114
A01 A24	HLA-A*29:02	162	SANNCTFEY	0,7814
A01 A24	HLA-A*29:02	192	FVFKNIDGY	0,9181
A01 A24	HLA-A*29:02	240	TLLALHRSY	0,6806
A01 A24	HLA-A*29:02	257	GWTAGAAAY	0,5826
A01 A24	HLA-A*29:02	257	GWTAGAAAYY	0,6803
A01 A24	HLA-A*29:02	258	WTAGAAAYY	0,9403
A01 A24	HLA-A*29:02	261	GAAAYYVGY	0,7714
A01 A24	HLA-A*29:02	360	NCVADYSVLY	0,6347
A01 A24	HLA-A*29:02	361	CVADYSVLY	0,9086
A01 A24	HLA-A*29:02	444	KVGGNYNYLY	0,5931
A01 A24	HLA-A*29:02	445	VGGNYNYLY	0,6436

A01 A24	HLA-A*29:02	496	GFQPTNGVGY	0,6858
A01 A24	HLA-A*29:02	604	TSNQVAVLY	0,8854
A01 A24	HLA-A*29:02	628	QLTPTWRVY	0,51
A01 A24	HLA-A*29:02	686	SVASQSIIAY	0,6064
A01 A24	HLA-A*29:02	687	VASQSIIAY	0,6791
A01 A24	HLA-A*29:02	781	VFAQVKQIY	0,9197
A01 A24	HLA-A*29:02	827	TLADAGFIKQY	0,5983
A01 A24	HLA-A*29:02	865	LTDEMIAQY	0,819
A01 A24	HLA-A*29:02	896	IPFAMQMAY	0,5343
A01 A24	HLA-A*29:02	909	IGVTQNVLY	0,5165
A01 A24	HLA-A*29:02	1059	GVVFLHVTY	0,7218
A01 A24	HLA-A*29:02	1102	WFVTQRNFY	0,7439
A01 A24	HLA-A*29:02	1147	SFKEELDKY	0,6923
A01 A24	HLA-A*29:02	1264	VLKGVKLHY	0,8598
A01 A24	HLA-A*29:03	30	NSFTRGVYY	0,7608
A01 A24	HLA-A*29:03	151	SWMESEFRVY	0,5544
A01 A24	HLA-A*29:03	152	WMESEFRVY	0,5108
A01 A24	HLA-A*29:03	162	SANNCTFEY	0,6647
A01 A24	HLA-A*29:03	192	FVFKNIDGY	0,814
A01 A24	HLA-A*29:03	240	TLLALHRSY	0,5915
A01 A24	HLA-A*29:03	258	WTAGAAAYY	0,863
A01 A24	HLA-A*29:03	261	GAAAYYVGY	0,648
A01 A24	HLA-A*29:03	360	NCVADYSVLY	0,6374
A01 A24	HLA-A*29:03	361	CVADYSVLY	0,8638
A01 A24	HLA-A*29:03	445	VGGNYNYLY	0,5077
A01 A24	HLA-A*29:03	603	NTSNQVAVLY	0,5268
A01 A24	HLA-A*29:03	604	TSNQVAVLY	0,8418
A01 A24	HLA-A*29:03	781	VFAQVKQIY	0,8384
A01 A24	HLA-A*29:03	865	LTDEMIAQY	0,7807
A01 A24	HLA-A*29:03	1102	WFVTQRNFY	0,612
A01 A24	HLA-A*29:05	30	NSFTRGVYY	0,6683
A01 A24	HLA-A*29:05	192	FVFKNIDGY	0,6624
A01 A24	HLA-A*29:05	240	TLLALHRSY	0,5807
A01 A24	HLA-A*29:05	257	GWTAGAAAYY	0,595
A01 A24	HLA-A*29:05	258	WTAGAAAYY	0,7752
A01 A24	HLA-A*29:05	361	CVADYSVLY	0,6495
A01 A24	HLA-A*29:05	496	GFQPTNGVGY	0,6148
A01 A24	HLA-A*29:05	604	TSNQVAVLY	0,6551
A01 A24	HLA-A*29:05	686	SVASQSIIAY	0,547
A01 A24	HLA-A*29:05	781	VFAQVKQIY	0,7974
A01 A24	HLA-A*29:05	1102	WFVTQRNFY	0,5904
A01 A24	HLA-A*29:05	1264	VLKGVKLHY	0,8571
A01 A24	HLA-A*29:06	30	NSFTRGVYY	0,825
A01 A24	HLA-A*29:06	151	SWMESEFRVY	0,7114
A01 A24	HLA-A*29:06	162	SANNCTFEY	0,7814
A01 A24	HLA-A*29:06	192	FVFKNIDGY	0,9181
A01 A24	HLA-A*29:06	240	TLLALHRSY	0,6806
A01 A24	HLA-A*29:06	257	GWTAGAAAY	0,5826
A01 A24	HLA-A*29:06	257	GWTAGAAAYY	0,6803
A01 A24	HLA-A*29:06	258	WTAGAAAYY	0,9403

A01 A24	HLA-A*29:06	261	GAAAYYVGY	0,7714
A01 A24	HLA-A*29:06	360	NCVADYSVLY	0,6347
A01 A24	HLA-A*29:06	361	CVADYSVLY	0,9086
A01 A24	HLA-A*29:06	444	KVGGNYNYLY	0,5931
A01 A24	HLA-A*29:06	445	VGGNYNYLY	0,6436
A01 A24	HLA-A*29:06	496	GFQPTNGVGY	0,6858
A01 A24	HLA-A*29:06	604	TSNQVAVLY	0,8854
A01 A24	HLA-A*29:06	628	QLTPTWRVY	0,51
A01 A24	HLA-A*29:06	686	SVASQSIIAY	0,6064
A01 A24	HLA-A*29:06	687	VASQSIIAY	0,6791
A01 A24	HLA-A*29:06	781	VFAQVKQIY	0,9197
A01 A24	HLA-A*29:06	827	TLADAGFIKQY	0,5983
A01 A24	HLA-A*29:06	865	LTDEMIAQY	0,819
A01 A24	HLA-A*29:06	896	IPFAMQMAY	0,5343
A01 A24	HLA-A*29:06	909	IGVTQNVLY	0,5165
A01 A24	HLA-A*29:06	1059	GVVFLHVTY	0,7218
A01 A24	HLA-A*29:06	1102	WFVTQRNFY	0,7439
A01 A24	HLA-A*29:06	1147	SFKEELDKY	0,6923
A01 A24	HLA-A*29:06	1264	VLKGVKLHY	0,8598
A01 A24	HLA-A*29:09	30	NSFTRGVYY	0,825
A01 A24	HLA-A*29:09	151	SWMESEFRVY	0,7114
A01 A24	HLA-A*29:09	162	SANNCTFEY	0,7814
A01 A24	HLA-A*29:09	192	FVFKNIDGY	0,9181
A01 A24	HLA-A*29:09	240	TLLALHRSY	0,6806
A01 A24	HLA-A*29:09	257	GWTAGAAAY	0,5826
A01 A24	HLA-A*29:09	257	GWTAGAAAYY	0,6803
A01 A24	HLA-A*29:09	258	WTAGAAAYY	0,9403
A01 A24	HLA-A*29:09	261	GAAAYYVGY	0,7714
A01 A24	HLA-A*29:09	360	NCVADYSVLY	0,6347
A01 A24	HLA-A*29:09	361	CVADYSVLY	0,9086
A01 A24	HLA-A*29:09	444	KVGGNYNYLY	0,5931
A01 A24	HLA-A*29:09	445	VGGNYNYLY	0,6436
A01 A24	HLA-A*29:09	496	GFQPTNGVGY	0,6858
A01 A24	HLA-A*29:09	604	TSNQVAVLY	0,8854
A01 A24	HLA-A*29:09	628	QLTPTWRVY	0,51
A01 A24	HLA-A*29:09	686	SVASQSIIAY	0,6064
A01 A24	HLA-A*29:09	687	VASQSIIAY	0,6791
A01 A24	HLA-A*29:09	781	VFAQVKQIY	0,9197
A01 A24	HLA-A*29:09	827	TLADAGFIKQY	0,5983
A01 A24	HLA-A*29:09	865	LTDEMIAQY	0,819
A01 A24	HLA-A*29:09	896	IPFAMQMAY	0,5343
A01 A24	HLA-A*29:09	909	IGVTQNVLY	0,5165
A01 A24	HLA-A*29:09	1059	GVVFLHVTY	0,7218
A01 A24	HLA-A*29:09	1102	WFVTQRNFY	0,7439
A01 A24	HLA-A*29:09	1147	SFKEELDKY	0,6923
A01 A24	HLA-A*29:09	1264	VLKGVKLHY	0,8598
A01 A24	HLA-A*29:10	30	NSFTRGVYY	0,825
A01 A24	HLA-A*29:10	151	SWMESEFRVY	0,7114
A01 A24	HLA-A*29:10	162	SANNCTFEY	0,7814
A01 A24	HLA-A*29:10	192	FVFKNIDGY	0,9181

A01 A24	HLA-A*29:10	240	TLLALHRSY	0,6806
A01 A24	HLA-A*29:10	257	GWTAGAAAY	0,5826
A01 A24	HLA-A*29:10	257	GWTAGAAAYY	0,6803
A01 A24	HLA-A*29:10	258	WTAGAAAYY	0,9403
A01 A24	HLA-A*29:10	261	GAAAYYVGY	0,7714
A01 A24	HLA-A*29:10	360	NCVADYSVLY	0,6347
A01 A24	HLA-A*29:10	361	CVADYSVLY	0,9086
A01 A24	HLA-A*29:10	444	KVGGNYNYLY	0,5931
A01 A24	HLA-A*29:10	445	VGGNYNYLY	0,6436
A01 A24	HLA-A*29:10	496	GFQPTNGVGY	0,6858
A01 A24	HLA-A*29:10	604	TSNQVAVLY	0,8854
A01 A24	HLA-A*29:10	628	QLTPTWRVY	0,51
A01 A24	HLA-A*29:10	686	SVASQSIIAY	0,6064
A01 A24	HLA-A*29:10	687	VASQSIIAY	0,6791
A01 A24	HLA-A*29:10	781	VFAQVKQIY	0,9197
A01 A24	HLA-A*29:10	827	TLADAGFIKQY	0,5983
A01 A24	HLA-A*29:10	865	LTDEMIAQY	0,819
A01 A24	HLA-A*29:10	896	IPFAMQMAY	0,5343
A01 A24	HLA-A*29:10	909	IGVTQNVLY	0,5165
A01 A24	HLA-A*29:10	1059	GVVFLHVTY	0,7218
A01 A24	HLA-A*29:10	1102	WFVTQRNFY	0,7439
A01 A24	HLA-A*29:10	1147	SFKEELDKY	0,6923
A01 A24	HLA-A*29:10	1264	VLKGVKLHY	0,8598
A01 A24	HLA-A*29:11	30	NSFTRGVYY	0,825
A01 A24	HLA-A*29:11	151	SWMESEFRVY	0,7114
A01 A24	HLA-A*29:11	162	SANNCTFEY	0,7814
A01 A24	HLA-A*29:11	192	FVFKNIDGY	0,9181
A01 A24	HLA-A*29:11	240	TLLALHRSY	0,6806
A01 A24	HLA-A*29:11	257	GWTAGAAAY	0,5826
A01 A24	HLA-A*29:11	257	GWTAGAAAYY	0,6803
A01 A24	HLA-A*29:11	258	WTAGAAAYY	0,9403
A01 A24	HLA-A*29:11	261	GAAAYYVGY	0,7714
A01 A24	HLA-A*29:11	360	NCVADYSVLY	0,6347
A01 A24	HLA-A*29:11	361	CVADYSVLY	0,9086
A01 A24	HLA-A*29:11	444	KVGGNYNYLY	0,5931
A01 A24	HLA-A*29:11	445	VGGNYNYLY	0,6436
A01 A24	HLA-A*29:11	496	GFQPTNGVGY	0,6858
A01 A24	HLA-A*29:11	604	TSNQVAVLY	0,8854
A01 A24	HLA-A*29:11	628	QLTPTWRVY	0,51
A01 A24	HLA-A*29:11	686	SVASQSIIAY	0,6064
A01 A24	HLA-A*29:11	687	VASQSIIAY	0,6791
A01 A24	HLA-A*29:11	781	VFAQVKQIY	0,9197
A01 A24	HLA-A*29:11	827	TLADAGFIKQY	0,5983
A01 A24	HLA-A*29:11	865	LTDEMIAQY	0,819
A01 A24	HLA-A*29:11	896	IPFAMQMAY	0,5343
A01 A24	HLA-A*29:11	909	IGVTQNVLY	0,5165
A01 A24	HLA-A*29:11	1059	GVVFLHVTY	0,7218
A01 A24	HLA-A*29:11	1102	WFVTQRNFY	0,7439
A01 A24	HLA-A*29:11	1147	SFKEELDKY	0,6923
A01 A24	HLA-A*29:11	1264	VLKGVKLHY	0,8598

A01 A24	HLA-A*29:12	30	NSFTRGVYY	0,825
A01 A24	HLA-A*29:12	151	SWMESEFRVY	0,7114
A01 A24	HLA-A*29:12	162	SANNCTFEY	0,7814
A01 A24	HLA-A*29:12	192	FVFKNIDGY	0,9181
A01 A24	HLA-A*29:12	240	TLLALHRSY	0,6806
A01 A24	HLA-A*29:12	257	GWTAGAAAY	0,5826
A01 A24	HLA-A*29:12	257	GWTAGAAAYY	0,6803
A01 A24	HLA-A*29:12	258	WTAGAAAYY	0,9403
A01 A24	HLA-A*29:12	261	GAAAYYVGY	0,7714
A01 A24	HLA-A*29:12	360	NCVADYSVLY	0,6347
A01 A24	HLA-A*29:12	361	CVADYSVLY	0,9086
A01 A24	HLA-A*29:12	444	KVGGNYNYLY	0,5931
A01 A24	HLA-A*29:12	445	VGGNYNYLY	0,6436
A01 A24	HLA-A*29:12	496	GFQPTNGVGY	0,6858
A01 A24	HLA-A*29:12	604	TSNQVAVLY	0,8854
A01 A24	HLA-A*29:12	628	QLTPTWRVY	0,51
A01 A24	HLA-A*29:12	686	SVASQSIIAY	0,6064
A01 A24	HLA-A*29:12	687	VASQSIIAY	0,6791
A01 A24	HLA-A*29:12	781	VFAQVKQIY	0,9197
A01 A24	HLA-A*29:12	827	TLADAGFIKQY	0,5983
A01 A24	HLA-A*29:12	865	LTDEMIAQY	0,819
A01 A24	HLA-A*29:12	896	IPFAMQMAY	0,5343
A01 A24	HLA-A*29:12	909	IGVTQNVLY	0,5165
A01 A24	HLA-A*29:12	1059	GVVFLHVTY	0,7218
A01 A24	HLA-A*29:12	1102	WFVTQRNFY	0,7439
A01 A24	HLA-A*29:12	1147	SFKEELDKY	0,6923
A01 A24	HLA-A*29:12	1264	VLKGVKLHY	0,8598
A02	HLA-A*02:01	109	TLDSKTQSL	0,6782
A02	HLA-A*02:01	133	FQFCNDPFL	0,518
A02	HLA-A*02:01	269	YLQPRTFLL	0,973
A02	HLA-A*02:01	386	KLNDLCFTNV	0,7108
A02	HLA-A*02:01	417	KIADYNYKL	0,909
A02	HLA-A*02:01	424	KLPDDFTGCV	0,5916
A02	HLA-A*02:01	515	FELLHAPATV	0,6033
A02	HLA-A*02:01	610	VLYQDVNCTEV	0,5092
A02	HLA-A*02:01	691	SIIAYTMSL	0,7995
A02	HLA-A*02:01	718	FTISVTTEI	0,5257
A02	HLA-A*02:01	821	LLFNKVTLA	0,7857
A02	HLA-A*02:01	857	GLTVLPPLL	0,6786
A02	HLA-A*02:01	915	VLYENQKLI	0,5378
A02	HLA-A*02:01	975	SVLNDILSRL	0,5124
A02	HLA-A*02:01	976	VLNDILSRL	0,9507
A02	HLA-A*02:01	983	RLDKVEAEV	0,8609
A02	HLA-A*02:01	1000	RLQSLQTYV	0,7431
A02	HLA-A*02:01	1047	YHLMSFPQSA	0,5452
A02	HLA-A*02:01	1048	HLMSFPQSA	0,7385
A02	HLA-A*02:01	1060	VVFLHVTYV	0,6948
A02	HLA-A*02:01	1185	RLNEVAKNL	0,6189
A02	HLA-A*02:01	1192	NLNESLIDL	0,6972
A02	HLA-A*02:01	1220	FIAGLIAIV	0,8207

A02	HLA-A*02:02	109	TLDSKTQSL	0,825
A02	HLA-A*02:02	133	FQFCNDPFL	0,5968
A02	HLA-A*02:02	269	YLQPRTFLL	0,9773
A02	HLA-A*02:02	386	KLNDLCFTNV	0,8261
A02	HLA-A*02:02	416	GKIADYNYKL	0,6474
A02	HLA-A*02:02	417	KIADYNYKL	0,9705
A02	HLA-A*02:02	424	KLPDDFTGCV	0,8161
A02	HLA-A*02:02	512	VLSFELLHA	0,5802
A02	HLA-A*02:02	515	FELLHAPATV	0,5863
A02	HLA-A*02:02	691	SIIAYTMSL	0,8402
A02	HLA-A*02:02	718	FTISVTTEI	0,6992
A02	HLA-A*02:02	821	LLFNKVTLA	0,8324
A02	HLA-A*02:02	857	GLTVLPPLL	0,7823
A02	HLA-A*02:02	869	MIAQYTSAL	0,7746
A02	HLA-A*02:02	915	VLYENQKLI	0,6825
A02	HLA-A*02:02	937	SLSSTASAL	0,6944
A02	HLA-A*02:02	947	KLQDVVNQNA	0,5801
A02	HLA-A*02:02	958	ALNTLVKQL	0,8332
A02	HLA-A*02:02	975	SVLNDILSRL	0,878
A02	HLA-A*02:02	976	VLNDILSRL	0,989
A02	HLA-A*02:02	983	RLDKVEAEV	0,8636
A02	HLA-A*02:02	1000	RLQSLQTYV	0,8614
A02	HLA-A*02:02	1047	YHLMSFPQSA	0,6493
A02	HLA-A*02:02	1048	HLMSFPQSA	0,8395
A02	HLA-A*02:02	1060	VVFLHVTYV	0,6976
A02	HLA-A*02:02	1095	FVSNGTHWFV	0,621
A02	HLA-A*02:02	1185	RLNEVAKNL	0,9052
A02	HLA-A*02:02	1192	NLNESLIDL	0,8555
A02	HLA-A*02:02	1196	SLIDLQEL	0,7114
A02	HLA-A*02:02	1220	FIAGLIAIV	0,8951
A02	HLA-A*02:03	28	YTNSFTRGV	0,6379
A02	HLA-A*02:03	109	TLDSKTQSL	0,7157
A02	HLA-A*02:03	117	LLIVNNATNV	0,6411
A02	HLA-A*02:03	118	LIVNNATNV	0,6321
A02	HLA-A*02:03	119	IVNNATNVV	0,611
A02	HLA-A*02:03	202	KIYSKHTPI	0,6197
A02	HLA-A*02:03	241	LLALHRSYL	0,6696
A02	HLA-A*02:03	269	YLQPRTFLL	0,9474
A02	HLA-A*02:03	386	KLNDLCFTNV	0,8966
A02	HLA-A*02:03	417	KIADYNYKL	0,8266
A02	HLA-A*02:03	424	KLPDDFTGCV	0,8342
A02	HLA-A*02:03	512	VLSFELLHA	0,6927
A02	HLA-A*02:03	515	FELLHAPATV	0,75
A02	HLA-A*02:03	516	ELLHAPATV	0,6201
A02	HLA-A*02:03	610	VLYQDVNCTEV	0,6785
A02	HLA-A*02:03	634	RVYSTGSNV	0,7597
A02	HLA-A*02:03	691	SIIAYTMSL	0,8766
A02	HLA-A*02:03	718	FTISVTTEI	0,7603
A02	HLA-A*02:03	762	QLNRALTGI	0,7873
A02	HLA-A*02:03	821	LLFNKVTLA	0,9477

A02	HLA-A*02:03	869	MIAQYTSAL	0,7884
A02	HLA-A*02:03	915	VLYENQKLI	0,8141
A02	HLA-A*02:03	937	SLSSTASAL	0,7506
A02	HLA-A*02:03	947	KLQDVVNQNA	0,6818
A02	HLA-A*02:03	958	ALNTLVKQL	0,8303
A02	HLA-A*02:03	975	SVLNDILSRL	0,8775
A02	HLA-A*02:03	976	VLNDILSRL	0,986
A02	HLA-A*02:03	983	RLDKVEAEV	0,7407
A02	HLA-A*02:03	995	RLITGRLQSL	0,6909
A02	HLA-A*02:03	1000	RLQSLQTYV	0,9011
A02	HLA-A*02:03	1047	YHLMSFPQSA	0,7925
A02	HLA-A*02:03	1048	HLMSFPQSA	0,9264
A02	HLA-A*02:03	1060	VVFLHVTYV	0,8543
A02	HLA-A*02:03	1171	GINASVVNI	0,7487
A02	HLA-A*02:03	1185	RLNEVAKNL	0,8898
A02	HLA-A*02:03	1192	NLNESLIDL	0,7558
A02	HLA-A*02:03	1220	FIAGLIAIV	0,9522
A02	HLA-A*02:04	269	YLQPRTFLL	0,8826
A02	HLA-A*02:04	417	KIADYNYKL	0,7529
A02	HLA-A*02:04	691	SIIAYTMSL	0,6521
A02	HLA-A*02:04	821	LLFNKVTLA	0,5441
A02	HLA-A*02:04	976	VLNDILSRL	0,7971
A02	HLA-A*02:04	1060	VVFLHVTYV	0,5925
A02	HLA-A*02:05	28	YTNSFTRGV	0,5152
A02	HLA-A*02:05	133	FQFCNDPFL	0,6267
A02	HLA-A*02:05	269	YLQPRTFLL	0,8636
A02	HLA-A*02:05	417	KIADYNYKL	0,9165
A02	HLA-A*02:05	424	KLPDDFTGCV	0,6183
A02	HLA-A*02:05	691	SIIAYTMSL	0,7523
A02	HLA-A*02:05	718	FTISVTTEI	0,7976
A02	HLA-A*02:05	777	NTQEVFAQV	0,5189
A02	HLA-A*02:05	869	MIAQYTSAL	0,6311
A02	HLA-A*02:05	975	SVLNDILSRL	0,5129
A02	HLA-A*02:05	976	VLNDILSRL	0,9314
A02	HLA-A*02:05	1000	RLQSLQTYV	0,5133
A02	HLA-A*02:05	1060	VVFLHVTYV	0,6498
A02	HLA-A*02:05	1185	RLNEVAKNL	0,5946
A02	HLA-A*02:05	1220	FIAGLIAIV	0,7813
A02	HLA-A*02:06	133	FQFCNDPFL	0,7104
A02	HLA-A*02:06	133	FQFCNDPFLGV	0,5205
A02	HLA-A*02:06	269	YLQPRTFLL	0,8955
A02	HLA-A*02:06	417	KIADYNYKL	0,9064
A02	HLA-A*02:06	424	KLPDDFTGCV	0,5686
A02	HLA-A*02:06	612	YQDVNCTEV	0,5256
A02	HLA-A*02:06	691	SIIAYTMSL	0,847
A02	HLA-A*02:06	712	IAIPTNFTI	0,5224
A02	HLA-A*02:06	718	FTISVTTEI	0,8184
A02	HLA-A*02:06	721	SVTTEILPV	0,5642
A02	HLA-A*02:06	821	LLFNKVTLA	0,5419
A02	HLA-A*02:06	894	LQIPFAMQM	0,6147

A02	HLA-A*02:06	976	VLNDILSRL	0,8346
A02	HLA-A*02:06	983	RLDKVEAEV	0,6135
A02	HLA-A*02:06	1048	HLMSFPQSA	0,5289
A02	HLA-A*02:06	1060	VVFLHVTYV	0,7813
A02	HLA-A*02:06	1136	TVYDPLQPEL	0,5968
A02	HLA-A*02:06	1220	FIAGLIAIV	0,81
A02	HLA-A*02:07	269	YLQPRTFLL	0,8775
A02	HLA-A*02:09	109	TLDSKTQSL	0,6782
A02	HLA-A*02:09	133	FQFCNDPFL	0,518
A02	HLA-A*02:09	269	YLQPRTFLL	0,973
A02	HLA-A*02:09	386	KLNDLCFTNV	0,7108
A02	HLA-A*02:09	417	KIADYNYKL	0,909
A02	HLA-A*02:09	424	KLPDDFTGCV	0,5916
A02	HLA-A*02:09	515	FELLHAPATV	0,6033
A02	HLA-A*02:09	610	VLYQDVNCTEV	0,5092
A02	HLA-A*02:09	691	SIIAYTMSL	0,7995
A02	HLA-A*02:09	718	FTISVTTEI	0,5257
A02	HLA-A*02:09	821	LLFNKVTLA	0,7857
A02	HLA-A*02:09	857	GLTVLPPLL	0,6786
A02	HLA-A*02:09	915	VLYENQKLI	0,5378
A02	HLA-A*02:09	975	SVLNDILSRL	0,5124
A02	HLA-A*02:09	976	VLNDILSRL	0,9507
A02	HLA-A*02:09	983	RLDKVEAEV	0,8609
A02	HLA-A*02:09	1000	RLQSLQTYV	0,7431
A02	HLA-A*02:09	1047	YHLMSFPQSA	0,5452
A02	HLA-A*02:09	1048	HLMSFPQSA	0,7385
A02	HLA-A*02:09	1060	VVFLHVTYV	0,6948
A02	HLA-A*02:09	1185	RLNEVAKNL	0,6189
A02	HLA-A*02:09	1192	NLNESLIDL	0,6972
A02	HLA-A*02:09	1220	FIAGLIAIV	0,8207
A02	HLA-A*02:11	109	TLDSKTQSL	0,8988
A02	HLA-A*02:11	269	YLQPRTFLL	0,9932
A02	HLA-A*02:11	386	KLNDLCFTNV	0,8871
A02	HLA-A*02:11	417	KIADYNYKL	0,97
A02	HLA-A*02:11	424	KLPDDFTGCV	0,7417
A02	HLA-A*02:11	515	FELLHAPATV	0,7889
A02	HLA-A*02:11	610	VLYQDVNCTEV	0,6974
A02	HLA-A*02:11	691	SIIAYTMSL	0,9432
A02	HLA-A*02:11	718	FTISVTTEI	0,8216
A02	HLA-A*02:11	821	LLFNKVTLA	0,9443
A02	HLA-A*02:11	857	GLTVLPPLL	0,7856
A02	HLA-A*02:11	915	VLYENQKLI	0,8632
A02	HLA-A*02:11	958	ALNTLVKQL	0,8141
A02	HLA-A*02:11	975	SVLNDILSRL	0,7945
A02	HLA-A*02:11	976	VLNDILSRL	0,9917
A02	HLA-A*02:11	983	RLDKVEAEV	0,9677
A02	HLA-A*02:11	1000	RLQSLQTYV	0,9419
A02	HLA-A*02:11	1047	YHLMSFPQSA	0,7518
A02	HLA-A*02:11	1048	HLMSFPQSA	0,9136
A02	HLA-A*02:11	1060	VVFLHVTYV	0,8865

A02	HLA-A*02:11	1171	GINASVVNI	0,7552
A02	HLA-A*02:11	1185	RLNEVAKNL	0,9099
A02	HLA-A*02:11	1192	NLNESLIDL	0,8952
A02	HLA-A*02:11	1220	FIAGLIAIV	0,9533
A02	HLA-A*02:12	109	TLDSKTQSL	0,8428
A02	HLA-A*02:12	269	YLQPRTFLL	0,9818
A02	HLA-A*02:12	386	KLNDLCFTNV	0,7871
A02	HLA-A*02:12	417	KIADYNYKL	0,9054
A02	HLA-A*02:12	424	KLPDDFTGCV	0,6822
A02	HLA-A*02:12	515	FELLHAPATV	0,681
A02	HLA-A*02:12	610	VLYQDVNCTEV	0,5467
A02	HLA-A*02:12	691	SIIAYTMSL	0,7857
A02	HLA-A*02:12	718	FTISVTTEI	0,5137
A02	HLA-A*02:12	821	LLFNKVTLA	0,8068
A02	HLA-A*02:12	857	GLTVLPPLL	0,6662
A02	HLA-A*02:12	915	VLYENQKLI	0,6143
A02	HLA-A*02:12	958	ALNTLVKQL	0,608
A02	HLA-A*02:12	975	SVLNDILSRL	0,6353
A02	HLA-A*02:12	976	VLNDILSRL	0,9646
A02	HLA-A*02:12	983	RLDKVEAEV	0,9238
A02	HLA-A*02:12	1000	RLQSLQTYV	0,8752
A02	HLA-A*02:12	1047	YHLMSFPQSA	0,6061
A02	HLA-A*02:12	1048	HLMSFPQSA	0,7977
A02	HLA-A*02:12	1060	VVFLHVTYV	0,6387
A02	HLA-A*02:12	1185	RLNEVAKNL	0,7519
A02	HLA-A*02:12	1192	NLNESLIDL	0,8032
A02	HLA-A*02:12	1196	SLIDLQEL	0,5049
A02	HLA-A*02:12	1220	FIAGLIAIV	0,8684
A02	HLA-A*02:13	109	TLDSKTQSL	0,6983
A02	HLA-A*02:13	269	YLQPRTFLL	0,941
A02	HLA-A*02:13	386	KLNDLCFTNV	0,8077
A02	HLA-A*02:13	417	KIADYNYKL	0,6473
A02	HLA-A*02:13	424	KLPDDFTGCV	0,6563
A02	HLA-A*02:13	515	FELLHAPATV	0,6753
A02	HLA-A*02:13	610	VLYQDVNCTEV	0,5488
A02	HLA-A*02:13	634	RVYSTGSNV	0,5202
A02	HLA-A*02:13	691	SIIAYTMSL	0,7124
A02	HLA-A*02:13	762	QLNRALTGI	0,5324
A02	HLA-A*02:13	821	LLFNKVTLA	0,8697
A02	HLA-A*02:13	915	VLYENQKLI	0,7047
A02	HLA-A*02:13	937	SLSSTASAL	0,5679
A02	HLA-A*02:13	958	ALNTLVKQL	0,6032
A02	HLA-A*02:13	975	SVLNDILSRL	0,6032
A02	HLA-A*02:13	976	VLNDILSRL	0,945
A02	HLA-A*02:13	983	RLDKVEAEV	0,7965
A02	HLA-A*02:13	1000	RLQSLQTYV	0,8865
A02	HLA-A*02:13	1047	YHLMSFPQSA	0,5921
A02	HLA-A*02:13	1048	HLMSFPQSA	0,8168
A02	HLA-A*02:13	1060	VVFLHVTYV	0,6866
A02	HLA-A*02:13	1171	GINASVVNI	0,5495

A02	HLA-A*02:13	1185	RLNEVAKNL	0,7327
A02	HLA-A*02:13	1192	NLNESLIDL	0,6257
A02	HLA-A*02:13	1220	FIAGLIAIV	0,8509
A02	HLA-A*02:14	133	FQFCNDPFL	0,6515
A02	HLA-A*02:14	269	YLQPRTFLL	0,8753
A02	HLA-A*02:14	417	KIADYNYKL	0,8777
A02	HLA-A*02:14	424	KLPDDFTGCV	0,5064
A02	HLA-A*02:14	691	SIIAYTMSL	0,7378
A02	HLA-A*02:14	718	FTISVTTEI	0,6932
A02	HLA-A*02:14	894	LQIPFAMQM	0,5806
A02	HLA-A*02:14	976	VLNDILSRL	0,8034
A02	HLA-A*02:14	983	RLDKVEAEV	0,5388
A02	HLA-A*02:14	1060	VVFLHVTYV	0,6298
A02	HLA-A*02:14	1220	FIAGLIAIV	0,6444
A02	HLA-A*02:16	109	TLDSKTQSL	0,7274
A02	HLA-A*02:16	133	FQFCNDPFL	0,6062
A02	HLA-A*02:16	222	ALEPLVDLPIGI	0,5004
A02	HLA-A*02:16	269	YLQPRTFLL	0,9796
A02	HLA-A*02:16	386	KLNDLCFTNV	0,7336
A02	HLA-A*02:16	417	KIADYNYKL	0,9083
A02	HLA-A*02:16	424	KLPDDFTGCV	0,6179
A02	HLA-A*02:16	515	FELLHAPATV	0,7196
A02	HLA-A*02:16	610	VLYQDVNCTEV	0,6397
A02	HLA-A*02:16	691	SIIAYTMSL	0,8424
A02	HLA-A*02:16	718	FTISVTTEI	0,6337
A02	HLA-A*02:16	821	LLFNKVTLA	0,8492
A02	HLA-A*02:16	857	GLTVLPPLL	0,7288
A02	HLA-A*02:16	915	VLYENQKLI	0,6401
A02	HLA-A*02:16	958	ALNTLVKQL	0,5125
A02	HLA-A*02:16	975	SVLNDILSRL	0,55
A02	HLA-A*02:16	976	VLNDILSRL	0,953
A02	HLA-A*02:16	983	RLDKVEAEV	0,9079
A02	HLA-A*02:16	1000	RLQSLQTYV	0,8152
A02	HLA-A*02:16	1047	YHLMSFPQSA	0,6323
A02	HLA-A*02:16	1048	HLMSFPQSA	0,8072
A02	HLA-A*02:16	1060	VVFLHVTYV	0,7386
A02	HLA-A*02:16	1185	RLNEVAKNL	0,6572
A02	HLA-A*02:16	1192	NLNESLIDL	0,7695
A02	HLA-A*02:16	1220	FIAGLIAIV	0,8906
A02	HLA-A*02:17	269	YLQPRTFLL	0,8989
A02	HLA-A*02:17	417	KIADYNYKL	0,7084
A02	HLA-A*02:17	691	SIIAYTMSL	0,5715
A02	HLA-A*02:17	976	VLNDILSRL	0,7759
A02	HLA-A*02:18	269	YLQPRTFLL	0,8775
A02	HLA-A*02:19	109	TLDSKTQSL	0,8021
A02	HLA-A*02:19	269	YLQPRTFLL	0,9638
A02	HLA-A*02:19	386	KLNDLCFTNV	0,6484
A02	HLA-A*02:19	417	KIADYNYKL	0,7644
A02	HLA-A*02:19	424	KLPDDFTGCV	0,5568
A02	HLA-A*02:19	515	FELLHAPATV	0,5795

A02	HLA-A*02:19	516	ELLHAPATV	0,6189
A02	HLA-A*02:19	691	SIIAYTMSL	0,6332
A02	HLA-A*02:19	821	LLFNKVTLA	0,6129
A02	HLA-A*02:19	975	SVLNDILSRL	0,5189
A02	HLA-A*02:19	976	VLNDILSRL	0,9385
A02	HLA-A*02:19	983	RLDKVEAEV	0,8427
A02	HLA-A*02:19	1000	RLQSLQTYV	0,7225
A02	HLA-A*02:19	1048	HLMSFPQSA	0,6609
A02	HLA-A*02:19	1185	RLNEVAKNL	0,5986
A02	HLA-A*02:19	1192	NLNESLIDL	0,8007
A02	HLA-A*02:19	1220	FIAGLIAIV	0,8159
A02	HLA-A*02:20	269	YLQPRTFLL	0,9372
A02	HLA-A*02:20	386	KLNDLCFTNV	0,609
A02	HLA-A*02:20	417	KIADYNYKL	0,8535
A02	HLA-A*02:20	691	SIIAYTMSL	0,7417
A02	HLA-A*02:20	718	FTISVTTEI	0,5048
A02	HLA-A*02:20	821	LLFNKVTLA	0,6533
A02	HLA-A*02:20	857	GLTVLPPLL	0,5648
A02	HLA-A*02:20	976	VLNDILSRL	0,883
A02	HLA-A*02:20	983	RLDKVEAEV	0,7865
A02	HLA-A*02:20	1000	RLQSLQTYV	0,6742
A02	HLA-A*02:20	1048	HLMSFPQSA	0,6259
A02	HLA-A*02:20	1060	VVFLHVTYV	0,6374
A02	HLA-A*02:20	1192	NLNESLIDL	0,5432
A02	HLA-A*02:20	1220	FIAGLIAIV	0,6882
A02	HLA-A*02:21	133	FQFCNDPFL	0,7104
A02	HLA-A*02:21	133	FQFCNDPFLGV	0,5205
A02	HLA-A*02:21	269	YLQPRTFLL	0,8955
A02	HLA-A*02:21	417	KIADYNYKL	0,9064
A02	HLA-A*02:21	424	KLPDDFTGCV	0,5686
A02	HLA-A*02:21	612	YQDVNCTEV	0,5256
A02	HLA-A*02:21	691	SIIAYTMSL	0,847
A02	HLA-A*02:21	712	IAIPTNFTI	0,5224
A02	HLA-A*02:21	718	FTISVTTEI	0,8184
A02	HLA-A*02:21	721	SVTTEILPV	0,5642
A02	HLA-A*02:21	821	LLFNKVTLA	0,5419
A02	HLA-A*02:21	894	LQIPFAMQM	0,6147
A02	HLA-A*02:21	976	VLNDILSRL	0,8346
A02	HLA-A*02:21	983	RLDKVEAEV	0,6135
A02	HLA-A*02:21	1048	HLMSFPQSA	0,5289
A02	HLA-A*02:21	1060	VVFLHVTYV	0,7813
A02	HLA-A*02:21	1136	TVYDPLQPEL	0,5968
A02	HLA-A*02:21	1220	FIAGLIAIV	0,81
A02	HLA-A*02:22	109	TLDSKTQSL	0,8377
A02	HLA-A*02:22	133	FQFCNDPFL	0,6508
A02	HLA-A*02:22	269	YLQPRTFLL	0,9799
A02	HLA-A*02:22	386	KLNDLCFTNV	0,8656
A02	HLA-A*02:22	417	KIADYNYKL	0,9698
A02	HLA-A*02:22	424	KLPDDFTGCV	0,831
A02	HLA-A*02:22	512	VLSFELLHA	0,665

A02	HLA-A*02:22	515	FELLHAPATV	0,7224
A02	HLA-A*02:22	610	VLYQDVNCTEV	0,651
A02	HLA-A*02:22	691	SIIAYTMSL	0,882
A02	HLA-A*02:22	718	FTISVTTEI	0,8007
A02	HLA-A*02:22	762	QLNRALTGI	0,6498
A02	HLA-A*02:22	821	LLFNKVTLA	0,9056
A02	HLA-A*02:22	857	GLTVLPPLL	0,7806
A02	HLA-A*02:22	869	MIAQYTSAL	0,8034
A02	HLA-A*02:22	915	VLYENQKLI	0,746
A02	HLA-A*02:22	937	SLSSTASAL	0,7146
A02	HLA-A*02:22	947	KLQDVVNQNA	0,6761
A02	HLA-A*02:22	958	ALNTLVKQL	0,8347
A02	HLA-A*02:22	975	SVLNDILSRL	0,8914
A02	HLA-A*02:22	976	VLNDILSRL	0,9899
A02	HLA-A*02:22	983	RLDKVEAEV	0,8989
A02	HLA-A*02:22	1000	RLQSLQTYV	0,9071
A02	HLA-A*02:22	1047	YHLMSFPQSA	0,7592
A02	HLA-A*02:22	1048	HLMSFPQSA	0,902
A02	HLA-A*02:22	1060	VVFLHVTYV	0,7982
A02	HLA-A*02:22	1095	FVSNGTHWFV	0,6858
A02	HLA-A*02:22	1171	GINASVVNI	0,6497
A02	HLA-A*02:22	1185	RLNEVAKNL	0,8958
A02	HLA-A*02:22	1192	NLNESLIDL	0,8894
A02	HLA-A*02:22	1196	SLIDLQEL	0,7342
A02	HLA-A*02:22	1220	FIAGLIAIV	0,9461
A02	HLA-A*02:24	109	TLDSKTQSL	0,6782
A02	HLA-A*02:24	133	FQFCNDPFL	0,518
A02	HLA-A*02:24	269	YLQPRTFLL	0,973
A02	HLA-A*02:24	386	KLNDLCFTNV	0,7108
A02	HLA-A*02:24	417	KIADYNYKL	0,909
A02	HLA-A*02:24	424	KLPDDFTGCV	0,5916
A02	HLA-A*02:24	515	FELLHAPATV	0,6033
A02	HLA-A*02:24	610	VLYQDVNCTEV	0,5092
A02	HLA-A*02:24	691	SIIAYTMSL	0,7995
A02	HLA-A*02:24	718	FTISVTTEI	0,5257
A02	HLA-A*02:24	821	LLFNKVTLA	0,7857
A02	HLA-A*02:24	857	GLTVLPPLL	0,6786
A02	HLA-A*02:24	915	VLYENQKLI	0,5378
A02	HLA-A*02:24	975	SVLNDILSRL	0,5124
A02	HLA-A*02:24	976	VLNDILSRL	0,9507
A02	HLA-A*02:24	983	RLDKVEAEV	0,8609
A02	HLA-A*02:24	1000	RLQSLQTYV	0,7431
A02	HLA-A*02:24	1047	YHLMSFPQSA	0,5452
A02	HLA-A*02:24	1048	HLMSFPQSA	0,7385
A02	HLA-A*02:24	1060	VVFLHVTYV	0,6948
A02	HLA-A*02:24	1185	RLNEVAKNL	0,6189
A02	HLA-A*02:24	1192	NLNESLIDL	0,6972
A02	HLA-A*02:24	1220	FIAGLIAIV	0,8207
A02	HLA-A*02:25	109	TLDSKTQSL	0,6782
A02	HLA-A*02:25	133	FQFCNDPFL	0,518

A02	HLA-A*02:25	269	YLQPRTFLL	0,973
A02	HLA-A*02:25	386	KLNDLCFTNV	0,7108
A02	HLA-A*02:25	417	KIADYNYKL	0,909
A02	HLA-A*02:25	424	KLPDDFTGCV	0,5916
A02	HLA-A*02:25	515	FELLHAPATV	0,6033
A02	HLA-A*02:25	610	VLYQDVNCTEV	0,5092
A02	HLA-A*02:25	691	SIIAYTMSL	0,7995
A02	HLA-A*02:25	718	FTISVTTEI	0,5257
A02	HLA-A*02:25	821	LLFNKVTLA	0,7857
A02	HLA-A*02:25	857	GLTVLPPLL	0,6786
A02	HLA-A*02:25	915	VLYENQKLI	0,5378
A02	HLA-A*02:25	975	SVLNDILSRL	0,5124
A02	HLA-A*02:25	976	VLNDILSRL	0,9507
A02	HLA-A*02:25	983	RLDKVEAEV	0,8609
A02	HLA-A*02:25	1000	RLQSLQTYV	0,7431
A02	HLA-A*02:25	1047	YHLMSFPQSA	0,5452
A02	HLA-A*02:25	1048	HLMSFPQSA	0,7385
A02	HLA-A*02:25	1060	VVFLHVTYV	0,6948
A02	HLA-A*02:25	1185	RLNEVAKNL	0,6189
A02	HLA-A*02:25	1192	NLNESLIDL	0,6972
A02	HLA-A*02:25	1220	FIAGLIAIV	0,8207
A02	HLA-A*02:26	269	YLQPRTFLL	0,9227
A02	HLA-A*02:26	386	KLNDLCFTNV	0,779
A02	HLA-A*02:26	417	KIADYNYKL	0,6418
A02	HLA-A*02:26	424	KLPDDFTGCV	0,6197
A02	HLA-A*02:26	515	FELLHAPATV	0,6456
A02	HLA-A*02:26	610	VLYQDVNCTEV	0,564
A02	HLA-A*02:26	634	RVYSTGSNV	0,5791
A02	HLA-A*02:26	691	SIIAYTMSL	0,75
A02	HLA-A*02:26	718	FTISVTTEI	0,5223
A02	HLA-A*02:26	821	LLFNKVTLA	0,8849
A02	HLA-A*02:26	915	VLYENQKLI	0,6862
A02	HLA-A*02:26	975	SVLNDILSRL	0,5439
A02	HLA-A*02:26	976	VLNDILSRL	0,9394
A02	HLA-A*02:26	983	RLDKVEAEV	0,7333
A02	HLA-A*02:26	1000	RLQSLQTYV	0,7922
A02	HLA-A*02:26	1047	YHLMSFPQSA	0,5858
A02	HLA-A*02:26	1048	HLMSFPQSA	0,8161
A02	HLA-A*02:26	1060	VVFLHVTYV	0,7683
A02	HLA-A*02:26	1171	GINASVVNI	0,5171
A02	HLA-A*02:26	1185	RLNEVAKNL	0,664
A02	HLA-A*02:26	1192	NLNESLIDL	0,524
A02	HLA-A*02:26	1220	FIAGLIAIV	0,8354
A02	HLA-A*02:27	109	TLDSKTQSL	0,7308
A02	HLA-A*02:27	269	YLQPRTFLL	0,9647
A02	HLA-A*02:27	386	KLNDLCFTNV	0,7459
A02	HLA-A*02:27	417	KIADYNYKL	0,7613
A02	HLA-A*02:27	424	KLPDDFTGCV	0,5772
A02	HLA-A*02:27	515	FELLHAPATV	0,627
A02	HLA-A*02:27	610	VLYQDVNCTEV	0,514

A02	HLA-A*02:27	691	SIIAYTMSL	0,6826
A02	HLA-A*02:27	821	LLFNKVTLA	0,8062
A02	HLA-A*02:27	857	GLTVLPPLL	0,5289
A02	HLA-A*02:27	915	VLYENQKLI	0,6196
A02	HLA-A*02:27	958	ALNTLVKQL	0,5442
A02	HLA-A*02:27	975	SVLNDILSRL	0,5493
A02	HLA-A*02:27	976	VLNDILSRL	0,9341
A02	HLA-A*02:27	983	RLDKVEAEV	0,8729
A02	HLA-A*02:27	1000	RLQSLQTYV	0,8703
A02	HLA-A*02:27	1047	YHLMSFPQSA	0,5242
A02	HLA-A*02:27	1048	HLMSFPQSA	0,7531
A02	HLA-A*02:27	1060	VVFLHVTYV	0,5815
A02	HLA-A*02:27	1185	RLNEVAKNL	0,685
A02	HLA-A*02:27	1192	NLNESLIDL	0,6819
A02	HLA-A*02:27	1220	FIAGLIAIV	0,798
A02	HLA-A*02:28	133	FQFCNDPFL	0,7104
A02	HLA-A*02:28	133	FQFCNDPFLGV	0,5205
A02	HLA-A*02:28	269	YLQPRTFLL	0,8955
A02	HLA-A*02:28	417	KIADYNYKL	0,9064
A02	HLA-A*02:28	424	KLPDDFTGCV	0,5686
A02	HLA-A*02:28	612	YQDVNCTEV	0,5256
A02	HLA-A*02:28	691	SIIAYTMSL	0,847
A02	HLA-A*02:28	712	IAIPTNFTI	0,5224
A02	HLA-A*02:28	718	FTISVTTEI	0,8184
A02	HLA-A*02:28	721	SVTTEILPV	0,5642
A02	HLA-A*02:28	821	LLFNKVTLA	0,5419
A02	HLA-A*02:28	894	LQIPFAMQM	0,6147
A02	HLA-A*02:28	976	VLNDILSRL	0,8346
A02	HLA-A*02:28	983	RLDKVEAEV	0,6135
A02	HLA-A*02:28	1048	HLMSFPQSA	0,5289
A02	HLA-A*02:28	1060	VVFLHVTYV	0,7813
A02	HLA-A*02:28	1136	TVYDPLQPEL	0,5968
A02	HLA-A*02:28	1220	FIAGLIAIV	0,81
A02	HLA-A*02:30	109	TLDSKTQSL	0,6782
A02	HLA-A*02:30	133	FQFCNDPFL	0,518
A02	HLA-A*02:30	269	YLQPRTFLL	0,973
A02	HLA-A*02:30	386	KLNDLCFTNV	0,7108
A02	HLA-A*02:30	417	KIADYNYKL	0,909
A02	HLA-A*02:30	424	KLPDDFTGCV	0,5916
A02	HLA-A*02:30	515	FELLHAPATV	0,6033
A02	HLA-A*02:30	610	VLYQDVNCTEV	0,5092
A02	HLA-A*02:30	691	SIIAYTMSL	0,7995
A02	HLA-A*02:30	718	FTISVTTEI	0,5257
A02	HLA-A*02:30	821	LLFNKVTLA	0,7857
A02	HLA-A*02:30	857	GLTVLPPLL	0,6786
A02	HLA-A*02:30	915	VLYENQKLI	0,5378
A02	HLA-A*02:30	975	SVLNDILSRL	0,5124
A02	HLA-A*02:30	976	VLNDILSRL	0,9507
A02	HLA-A*02:30	983	RLDKVEAEV	0,8609
A02	HLA-A*02:30	1000	RLQSLQTYV	0,7431

A02	HLA-A*02:30	1047	YHLMSFPQSA	0,5452
A02	HLA-A*02:30	1048	HLMSFPQSA	0,7385
A02	HLA-A*02:30	1060	VVFLHVTYV	0,6948
A02	HLA-A*02:30	1185	RLNEVAKNL	0,6189
A02	HLA-A*02:30	1192	NLNESLIDL	0,6972
A02	HLA-A*02:30	1220	FIAGLIAIV	0,8207
A02	HLA-A*02:31	109	TLDSKTQSL	0,6782
A02	HLA-A*02:31	133	FQFCNDPFL	0,518
A02	HLA-A*02:31	269	YLQPRTFLL	0,973
A02	HLA-A*02:31	386	KLNDLCFTNV	0,7108
A02	HLA-A*02:31	417	KIADYNYKL	0,909
A02	HLA-A*02:31	424	KLPDDFTGCV	0,5916
A02	HLA-A*02:31	515	FELLHAPATV	0,6033
A02	HLA-A*02:31	610	VLYQDVNCTEV	0,5092
A02	HLA-A*02:31	691	SIIAYTMSL	0,7995
A02	HLA-A*02:31	718	FTISVTTEI	0,5257
A02	HLA-A*02:31	821	LLFNKVTLA	0,7857
A02	HLA-A*02:31	857	GLTVLPPLL	0,6786
A02	HLA-A*02:31	915	VLYENQKLI	0,5378
A02	HLA-A*02:31	975	SVLNDILSRL	0,5124
A02	HLA-A*02:31	976	VLNDILSRL	0,9507
A02	HLA-A*02:31	983	RLDKVEAEV	0,8609
A02	HLA-A*02:31	1000	RLQSLQTYV	0,7431
A02	HLA-A*02:31	1047	YHLMSFPQSA	0,5452
A02	HLA-A*02:31	1048	HLMSFPQSA	0,7385
A02	HLA-A*02:31	1060	VVFLHVTYV	0,6948
A02	HLA-A*02:31	1185	RLNEVAKNL	0,6189
A02	HLA-A*02:31	1192	NLNESLIDL	0,6972
A02	HLA-A*02:31	1220	FIAGLIAIV	0,8207
A02	HLA-A*02:36	109	TLDSKTQSL	0,6446
A02	HLA-A*02:36	269	YLQPRTFLL	0,9384
A02	HLA-A*02:36	386	KLNDLCFTNV	0,5465
A02	HLA-A*02:36	417	KIADYNYKL	0,763
A02	HLA-A*02:36	515	FELLHAPATV	0,5014
A02	HLA-A*02:36	516	ELLHAPATV	0,5347
A02	HLA-A*02:36	691	SIIAYTMSL	0,6521
A02	HLA-A*02:36	821	LLFNKVTLA	0,595
A02	HLA-A*02:36	976	VLNDILSRL	0,9103
A02	HLA-A*02:36	983	RLDKVEAEV	0,7362
A02	HLA-A*02:36	1000	RLQSLQTYV	0,5633
A02	HLA-A*02:36	1048	HLMSFPQSA	0,5791
A02	HLA-A*02:36	1060	VVFLHVTYV	0,5499
A02	HLA-A*02:36	1192	NLNESLIDL	0,6714
A02	HLA-A*02:36	1220	FIAGLIAIV	0,7437
A02	HLA-A*02:38	109	TLDSKTQSL	0,6763
A02	HLA-A*02:38	269	YLQPRTFLL	0,8978
A02	HLA-A*02:38	386	KLNDLCFTNV	0,6697
A02	HLA-A*02:38	417	KIADYNYKL	0,6147
A02	HLA-A*02:38	424	KLPDDFTGCV	0,5937
A02	HLA-A*02:38	515	FELLHAPATV	0,6135

A02	HLA-A*02:38	610	VLYQDVNCTEV	0,5165
A02	HLA-A*02:38	691	SIIAYTMSL	0,7292
A02	HLA-A*02:38	718	FTISVTTEI	0,5114
A02	HLA-A*02:38	821	LLFNKVTLA	0,7991
A02	HLA-A*02:38	915	VLYENQKLI	0,6879
A02	HLA-A*02:38	937	SLSSTASAL	0,5212
A02	HLA-A*02:38	958	ALNTLVKQL	0,5209
A02	HLA-A*02:38	975	SVLNDILSRL	0,5586
A02	HLA-A*02:38	976	VLNDILSRL	0,9012
A02	HLA-A*02:38	983	RLDKVEAEV	0,7049
A02	HLA-A*02:38	1000	RLQSLQTYV	0,7606
A02	HLA-A*02:38	1047	YHLMSFPQSA	0,5778
A02	HLA-A*02:38	1048	HLMSFPQSA	0,7518
A02	HLA-A*02:38	1060	VVFLHVTYV	0,6814
A02	HLA-A*02:38	1171	GINASVVNI	0,5375
A02	HLA-A*02:38	1185	RLNEVAKNL	0,6207
A02	HLA-A*02:38	1192	NLNESLIDL	0,6694
A02	HLA-A*02:38	1220	FIAGLIAIV	0,8041
A02	HLA-A*02:39	269	YLQPRTFLL	0,9532
A02	HLA-A*02:39	417	KIADYNYKL	0,7841
A02	HLA-A*02:39	691	SIIAYTMSL	0,5907
A02	HLA-A*02:39	821	LLFNKVTLA	0,5231
A02	HLA-A*02:39	857	GLTVLPPLL	0,5068
A02	HLA-A*02:39	976	VLNDILSRL	0,8817
A02	HLA-A*02:39	983	RLDKVEAEV	0,6488
A02	HLA-A*02:39	1000	RLQSLQTYV	0,5067
A02	HLA-A*02:39	1192	NLNESLIDL	0,5275
A02	HLA-A*02:39	1220	FIAGLIAIV	0,6464
A02	HLA-A*02:40	109	TLDSKTQSL	0,6782
A02	HLA-A*02:40	133	FQFCNDPFL	0,518
A02	HLA-A*02:40	269	YLQPRTFLL	0,973
A02	HLA-A*02:40	386	KLNDLCFTNV	0,7108
A02	HLA-A*02:40	417	KIADYNYKL	0,909
A02	HLA-A*02:40	424	KLPDDFTGCV	0,5916
A02	HLA-A*02:40	515	FELLHAPATV	0,6033
A02	HLA-A*02:40	610	VLYQDVNCTEV	0,5092
A02	HLA-A*02:40	691	SIIAYTMSL	0,7995
A02	HLA-A*02:40	718	FTISVTTEI	0,5257
A02	HLA-A*02:40	821	LLFNKVTLA	0,7857
A02	HLA-A*02:40	857	GLTVLPPLL	0,6786
A02	HLA-A*02:40	915	VLYENQKLI	0,5378
A02	HLA-A*02:40	975	SVLNDILSRL	0,5124
A02	HLA-A*02:40	976	VLNDILSRL	0,9507
A02	HLA-A*02:40	983	RLDKVEAEV	0,8609
A02	HLA-A*02:40	1000	RLQSLQTYV	0,7431
A02	HLA-A*02:40	1047	YHLMSFPQSA	0,5452
A02	HLA-A*02:40	1048	HLMSFPQSA	0,7385
A02	HLA-A*02:40	1060	VVFLHVTYV	0,6948
A02	HLA-A*02:40	1185	RLNEVAKNL	0,6189
A02	HLA-A*02:40	1192	NLNESLIDL	0,6972

A02	HLA-A*02:40	1220	FIAGLIAIV	0,8207
A02	HLA-A*02:44	109	TLDSKTQSL	0,722
A02	HLA-A*02:44	133	FQFCNDPFL	0,6901
A02	HLA-A*02:44	269	YLQPRTFLL	0,9483
A02	HLA-A*02:44	386	KLNDLCFTNV	0,6273
A02	HLA-A*02:44	417	KIADYNYKL	0,9374
A02	HLA-A*02:44	424	KLPDDFTGCV	0,713
A02	HLA-A*02:44	612	YQDVNCTEV	0,6481
A02	HLA-A*02:44	691	SIIAYTMSL	0,873
A02	HLA-A*02:44	718	FTISVTTEI	0,8345
A02	HLA-A*02:44	721	SVTTEILPV	0,6283
A02	HLA-A*02:44	777	NTQEVFAQV	0,5675
A02	HLA-A*02:44	786	KQIYKTPPI	0,5169
A02	HLA-A*02:44	821	LLFNKVTLA	0,6213
A02	HLA-A*02:44	869	MIAQYTSAL	0,5812
A02	HLA-A*02:44	976	VLNDILSRL	0,9188
A02	HLA-A*02:44	983	RLDKVEAEV	0,7861
A02	HLA-A*02:44	1000	RLQSLQTYV	0,7229
A02	HLA-A*02:44	1048	HLMSFPQSA	0,6725
A02	HLA-A*02:44	1060	VVFLHVTYV	0,7638
A02	HLA-A*02:44	1095	FVSNGTHWFV	0,5522
A02	HLA-A*02:44	1136	TVYDPLQPEL	0,5349
A02	HLA-A*02:44	1171	GINASVVNI	0,5357
A02	HLA-A*02:44	1185	RLNEVAKNL	0,5876
A02	HLA-A*02:44	1192	NLNESLIDL	0,6815
A02	HLA-A*02:44	1196	SLIDLQEL	0,5121
A02	HLA-A*02:44	1220	FIAGLIAIV	0,868
A02	HLA-A*02:45	269	YLQPRTFLL	0,87
A02	HLA-A*02:45	417	KIADYNYKL	0,7495
A02	HLA-A*02:45	691	SIIAYTMSL	0,5724
A02	HLA-A*02:45	976	VLNDILSRL	0,7909
A02	HLA-A*02:45	983	RLDKVEAEV	0,6527
A02	HLA-A*02:46	269	YLQPRTFLL	0,897
A02	HLA-A*02:46	417	KIADYNYKL	0,7857
A02	HLA-A*02:46	691	SIIAYTMSL	0,5832
A02	HLA-A*02:46	976	VLNDILSRL	0,8016
A02	HLA-A*02:46	983	RLDKVEAEV	0,6558
A02	HLA-A*02:47	109	TLDSKTQSL	0,8176
A02	HLA-A*02:47	133	FQFCNDPFL	0,5963
A02	HLA-A*02:47	225	PLVDLPIGI	0,5831
A02	HLA-A*02:47	269	YLQPRTFLL	0,9772
A02	HLA-A*02:47	386	KLNDLCFTNV	0,8117
A02	HLA-A*02:47	416	GKIADYNYKL	0,6744
A02	HLA-A*02:47	417	KIADYNYKL	0,9749
A02	HLA-A*02:47	424	KLPDDFTGCV	0,8227
A02	HLA-A*02:47	691	SIIAYTMSL	0,8546
A02	HLA-A*02:47	718	FTISVTTEI	0,7036
A02	HLA-A*02:47	821	LLFNKVTLA	0,8262
A02	HLA-A*02:47	857	GLTVLPPLL	0,7895
A02	HLA-A*02:47	869	MIAQYTSAL	0,7897

A02	HLA-A*02:47	915	VLYENQKLI	0,6812
A02	HLA-A*02:47	937	SLSSTASAL	0,6691
A02	HLA-A*02:47	958	ALNTLVKQL	0,8279
A02	HLA-A*02:47	975	SVLNDILSRL	0,8782
A02	HLA-A*02:47	976	VLNDILSRL	0,9886
A02	HLA-A*02:47	983	RLDKVEAEV	0,849
A02	HLA-A*02:47	1000	RLQSLQTYV	0,8402
A02	HLA-A*02:47	1047	YHLMSFPQSA	0,6304
A02	HLA-A*02:47	1048	HLMSFPQSA	0,8224
A02	HLA-A*02:47	1060	VVFLHVTYV	0,7118
A02	HLA-A*02:47	1095	FVSNGTHWFV	0,6164
A02	HLA-A*02:47	1185	RLNEVAKNL	0,9004
A02	HLA-A*02:47	1192	NLNESLIDL	0,8471
A02	HLA-A*02:47	1196	SLIDLQEL	0,7353
A02	HLA-A*02:47	1220	FIAGLIAIV	0,8847
A02	HLA-A*02:48	109	TLDSKTQSL	0,52
A02	HLA-A*02:48	269	YLQPRTFLL	0,9298
A02	HLA-A*02:48	417	KIADYNYKL	0,8626
A02	HLA-A*02:48	691	SIIAYTMSL	0,69
A02	HLA-A*02:48	976	VLNDILSRL	0,8779
A02	HLA-A*02:48	983	RLDKVEAEV	0,7153
A02	HLA-A*02:48	1000	RLQSLQTYV	0,6002
A02	HLA-A*02:48	1060	VVFLHVTYV	0,5094
A02	HLA-A*02:48	1185	RLNEVAKNL	0,5841
A02	HLA-A*02:49	109	TLDSKTQSL	0,8693
A02	HLA-A*02:49	269	YLQPRTFLL	0,9729
A02	HLA-A*02:49	386	KLNDLCFTNV	0,6741
A02	HLA-A*02:49	417	KIADYNYKL	0,8873
A02	HLA-A*02:49	424	KLPDDFTGCV	0,6103
A02	HLA-A*02:49	515	FELLHAPATV	0,5508
A02	HLA-A*02:49	612	YQDVNCTEV	0,5076
A02	HLA-A*02:49	691	SIIAYTMSL	0,6942
A02	HLA-A*02:49	821	LLFNKVTLA	0,6712
A02	HLA-A*02:49	857	GLTVLPPLL	0,5991
A02	HLA-A*02:49	975	SVLNDILSRL	0,5907
A02	HLA-A*02:49	976	VLNDILSRL	0,9452
A02	HLA-A*02:49	982	SRLDKVEAEV	0,5478
A02	HLA-A*02:49	983	RLDKVEAEV	0,9308
A02	HLA-A*02:49	1000	RLQSLQTYV	0,8043
A02	HLA-A*02:49	1048	HLMSFPQSA	0,6591
A02	HLA-A*02:49	1060	VVFLHVTYV	0,571
A02	HLA-A*02:49	1185	RLNEVAKNL	0,6724
A02	HLA-A*02:49	1192	NLNESLIDL	0,7823
A02	HLA-A*02:49	1220	FIAGLIAIV	0,7966
A02	HLA-A*02:51	133	FQFCNDPFL	0,7104
A02	HLA-A*02:51	133	FQFCNDPFLGV	0,5205
A02	HLA-A*02:51	269	YLQPRTFLL	0,8955
A02	HLA-A*02:51	417	KIADYNYKL	0,9064
A02	HLA-A*02:51	424	KLPDDFTGCV	0,5686
A02	HLA-A*02:51	612	YQDVNCTEV	0,5256

A02	HLA-A*02:51	691	SIIAYTMSL	0,847
A02	HLA-A*02:51	712	IAIPTNFTI	0,5224
A02	HLA-A*02:51	718	FTISVTTEI	0,8184
A02	HLA-A*02:51	721	SVTTEILPV	0,5642
A02	HLA-A*02:51	821	LLFNKVTLA	0,5419
A02	HLA-A*02:51	894	LQIPFAMQM	0,6147
A02	HLA-A*02:51	976	VLNDILSRL	0,8346
A02	HLA-A*02:51	983	RLDKVEAEV	0,6135
A02	HLA-A*02:51	1048	HLMSFPQSA	0,5289
A02	HLA-A*02:51	1060	VVFLHVTYV	0,7813
A02	HLA-A*02:51	1136	TVYDPLQPEL	0,5968
A02	HLA-A*02:51	1220	FIAGLIAIV	0,81
A02	HLA-A*02:54	109	TLDSKTQSL	0,6942
A02	HLA-A*02:54	269	YLQPRTFLL	0,9082
A02	HLA-A*02:54	417	KIADYNYKL	0,8163
A02	HLA-A*02:54	424	KLPDDFTGCV	0,5607
A02	HLA-A*02:54	516	ELLHAPATV	0,5493
A02	HLA-A*02:54	612	YQDVNCTEV	0,5542
A02	HLA-A*02:54	691	SIIAYTMSL	0,7472
A02	HLA-A*02:54	718	FTISVTTEI	0,731
A02	HLA-A*02:54	721	SVTTEILPV	0,5157
A02	HLA-A*02:54	777	NTQEVFAQV	0,6031
A02	HLA-A*02:54	976	VLNDILSRL	0,8799
A02	HLA-A*02:54	983	RLDKVEAEV	0,6743
A02	HLA-A*02:54	1000	RLQSLQTYV	0,5446
A02	HLA-A*02:54	1048	HLMSFPQSA	0,532
A02	HLA-A*02:54	1060	VVFLHVTYV	0,6044
A02	HLA-A*02:54	1192	NLNESLIDL	0,6437
A02	HLA-A*02:54	1220	FIAGLIAIV	0,7718
A02	HLA-A*02:56	269	YLQPRTFLL	0,6126
A02	HLA-A*02:56	417	KIADYNYKL	0,6147
A02	HLA-A*02:56	691	SIIAYTMSL	0,5351
A02	HLA-A*02:56	1060	VVFLHVTYV	0,5227
A02	HLA-A*02:57	269	YLQPRTFLL	0,7289
A02	HLA-A*02:57	417	KIADYNYKL	0,7431
A02	HLA-A*02:57	691	SIIAYTMSL	0,6331
A02	HLA-A*02:57	976	VLNDILSRL	0,5643
A02	HLA-A*02:57	1060	VVFLHVTYV	0,5368
A02	HLA-A*02:58	109	TLDSKTQSL	0,6907
A02	HLA-A*02:58	269	YLQPRTFLL	0,9723
A02	HLA-A*02:58	386	KLNDLCFTNV	0,6236
A02	HLA-A*02:58	417	KIADYNYKL	0,9119
A02	HLA-A*02:58	424	KLPDDFTGCV	0,5701
A02	HLA-A*02:58	691	SIIAYTMSL	0,7337
A02	HLA-A*02:58	821	LLFNKVTLA	0,6551
A02	HLA-A*02:58	857	GLTVLPPLL	0,6884
A02	HLA-A*02:58	975	SVLNDILSRL	0,5159
A02	HLA-A*02:58	976	VLNDILSRL	0,95
A02	HLA-A*02:58	983	RLDKVEAEV	0,8408
A02	HLA-A*02:58	1000	RLQSLQTYV	0,656

A02	HLA-A*02:58	1048	HLMSFPQSA	0,623
A02	HLA-A*02:58	1060	VVFLHVTYV	0,5564
A02	HLA-A*02:58	1185	RLNEVAKNL	0,6577
A02	HLA-A*02:58	1192	NLNESLIDL	0,6601
A02	HLA-A*02:58	1220	FIAGLIAIV	0,707
A02	HLA-A*02:59	109	TLDSKTQSL	0,6782
A02	HLA-A*02:59	133	FQFCNDPFL	0,518
A02	HLA-A*02:59	269	YLQPRTFLL	0,973
A02	HLA-A*02:59	386	KLNDLCFTNV	0,7108
A02	HLA-A*02:59	417	KIADYNYKL	0,909
A02	HLA-A*02:59	424	KLPDDFTGCV	0,5916
A02	HLA-A*02:59	515	FELLHAPATV	0,6033
A02	HLA-A*02:59	610	VLYQDVNCTEV	0,5092
A02	HLA-A*02:59	691	SIIAYTMSL	0,7995
A02	HLA-A*02:59	718	FTISVTTEI	0,5257
A02	HLA-A*02:59	821	LLFNKVTLA	0,7857
A02	HLA-A*02:59	857	GLTVLPPLL	0,6786
A02	HLA-A*02:59	915	VLYENQKLI	0,5378
A02	HLA-A*02:59	975	SVLNDILSRL	0,5124
A02	HLA-A*02:59	976	VLNDILSRL	0,9507
A02	HLA-A*02:59	983	RLDKVEAEV	0,8609
A02	HLA-A*02:59	1000	RLQSLQTYV	0,7431
A02	HLA-A*02:59	1047	YHLMSFPQSA	0,5452
A02	HLA-A*02:59	1048	HLMSFPQSA	0,7385
A02	HLA-A*02:59	1060	VVFLHVTYV	0,6948
A02	HLA-A*02:59	1185	RLNEVAKNL	0,6189
A02	HLA-A*02:59	1192	NLNESLIDL	0,6972
A02	HLA-A*02:59	1220	FIAGLIAIV	0,8207
A02	HLA-A*02:61	133	FQFCNDPFL	0,7104
A02	HLA-A*02:61	133	FQFCNDPFLGV	0,5205
A02	HLA-A*02:61	269	YLQPRTFLL	0,8955
A02	HLA-A*02:61	417	KIADYNYKL	0,9064
A02	HLA-A*02:61	424	KLPDDFTGCV	0,5686
A02	HLA-A*02:61	612	YQDVNCTEV	0,5256
A02	HLA-A*02:61	691	SIIAYTMSL	0,847
A02	HLA-A*02:61	712	IAIPTNFTI	0,5224
A02	HLA-A*02:61	718	FTISVTTEI	0,8184
A02	HLA-A*02:61	721	SVTTEILPV	0,5642
A02	HLA-A*02:61	821	LLFNKVTLA	0,5419
A02	HLA-A*02:61	894	LQIPFAMQM	0,6147
A02	HLA-A*02:61	976	VLNDILSRL	0,8346
A02	HLA-A*02:61	983	RLDKVEAEV	0,6135
A02	HLA-A*02:61	1048	HLMSFPQSA	0,5289
A02	HLA-A*02:61	1060	VVFLHVTYV	0,7813
A02	HLA-A*02:61	1136	TVYDPLQPEL	0,5968
A02	HLA-A*02:61	1220	FIAGLIAIV	0,81
A02	HLA-A*02:62	269	YLQPRTFLL	0,778
A02	HLA-A*02:62	417	KIADYNYKL	0,6875
A02	HLA-A*02:62	691	SIIAYTMSL	0,6033
A02	HLA-A*02:62	821	LLFNKVTLA	0,5193

A02	HLA-A*02:62	976	VLNDILSRL	0,681
A02	HLA-A*02:62	983	RLDKVEAEV	0,636
A02	HLA-A*02:62	1000	RLQSLQTYV	0,5378
A02	HLA-A*02:62	1060	VVFLHVTYV	0,6207
A02	HLA-A*02:62	1220	FIAGLIAIV	0,5269
A02	HLA-A*02:63	109	TLDSKTQSL	0,825
A02	HLA-A*02:63	133	FQFCNDPFL	0,5968
A02	HLA-A*02:63	269	YLQPRTFLL	0,9773
A02	HLA-A*02:63	386	KLNDLCFTNV	0,8261
A02	HLA-A*02:63	416	GKIADYNYKL	0,6474
A02	HLA-A*02:63	417	KIADYNYKL	0,9705
A02	HLA-A*02:63	424	KLPDDFTGCV	0,8161
A02	HLA-A*02:63	512	VLSFELLHA	0,5802
A02	HLA-A*02:63	515	FELLHAPATV	0,5863
A02	HLA-A*02:63	691	SIIAYTMSL	0,8402
A02	HLA-A*02:63	718	FTISVTTEI	0,6992
A02	HLA-A*02:63	821	LLFNKVTLA	0,8324
A02	HLA-A*02:63	857	GLTVLPPLL	0,7823
A02	HLA-A*02:63	869	MIAQYTSAL	0,7746
A02	HLA-A*02:63	915	VLYENQKLI	0,6825
A02	HLA-A*02:63	937	SLSSTASAL	0,6944
A02	HLA-A*02:63	947	KLQDVVNQNA	0,5801
A02	HLA-A*02:63	958	ALNTLVKQL	0,8332
A02	HLA-A*02:63	975	SVLNDILSRL	0,878
A02	HLA-A*02:63	976	VLNDILSRL	0,989
A02	HLA-A*02:63	983	RLDKVEAEV	0,8636
A02	HLA-A*02:63	1000	RLQSLQTYV	0,8614
A02	HLA-A*02:63	1047	YHLMSFPQSA	0,6493
A02	HLA-A*02:63	1048	HLMSFPQSA	0,8395
A02	HLA-A*02:63	1060	VVFLHVTYV	0,6976
A02	HLA-A*02:63	1095	FVSNGTHWFV	0,621
A02	HLA-A*02:63	1185	RLNEVAKNL	0,9052
A02	HLA-A*02:63	1192	NLNESLIDL	0,8555
A02	HLA-A*02:63	1196	SLIDLQEL	0,7114
A02	HLA-A*02:63	1220	FIAGLIAIV	0,8951
A02	HLA-A*02:66	109	TLDSKTQSL	0,6782
A02	HLA-A*02:66	133	FQFCNDPFL	0,518
A02	HLA-A*02:66	269	YLQPRTFLL	0,973
A02	HLA-A*02:66	386	KLNDLCFTNV	0,7108
A02	HLA-A*02:66	417	KIADYNYKL	0,909
A02	HLA-A*02:66	424	KLPDDFTGCV	0,5916
A02	HLA-A*02:66	515	FELLHAPATV	0,6033
A02	HLA-A*02:66	610	VLYQDVNCTEV	0,5092
A02	HLA-A*02:66	691	SIIAYTMSL	0,7995
A02	HLA-A*02:66	718	FTISVTTEI	0,5257
A02	HLA-A*02:66	821	LLFNKVTLA	0,7857
A02	HLA-A*02:66	857	GLTVLPPLL	0,6786
A02	HLA-A*02:66	915	VLYENQKLI	0,5378
A02	HLA-A*02:66	975	SVLNDILSRL	0,5124
A02	HLA-A*02:66	976	VLNDILSRL	0,9507

A02	HLA-A*02:66	983	RLDKVEAEV	0,8609
A02	HLA-A*02:66	1000	RLQSLQTYV	0,7431
A02	HLA-A*02:66	1047	YHLMSFPQSA	0,5452
A02	HLA-A*02:66	1048	HLMSFPQSA	0,7385
A02	HLA-A*02:66	1060	VVFLHVTYV	0,6948
A02	HLA-A*02:66	1185	RLNEVAKNL	0,6189
A02	HLA-A*02:66	1192	NLNESLIDL	0,6972
A02	HLA-A*02:66	1220	FIAGLIAIV	0,8207
A02	HLA-A*02:67	109	TLDSKTQSL	0,6782
A02	HLA-A*02:67	133	FQFCNDPFL	0,518
A02	HLA-A*02:67	269	YLQPRTFLL	0,973
A02	HLA-A*02:67	386	KLNDLCFTNV	0,7108
A02	HLA-A*02:67	417	KIADYNYKL	0,909
A02	HLA-A*02:67	424	KLPDDFTGCV	0,5916
A02	HLA-A*02:67	515	FELLHAPATV	0,6033
A02	HLA-A*02:67	610	VLYQDVNCTEV	0,5092
A02	HLA-A*02:67	691	SIIAYTMSL	0,7995
A02	HLA-A*02:67	718	FTISVTTEI	0,5257
A02	HLA-A*02:67	821	LLFNKVTLA	0,7857
A02	HLA-A*02:67	857	GLTVLPPLL	0,6786
A02	HLA-A*02:67	915	VLYENQKLI	0,5378
A02	HLA-A*02:67	975	SVLNDILSRL	0,5124
A02	HLA-A*02:67	976	VLNDILSRL	0,9507
A02	HLA-A*02:67	983	RLDKVEAEV	0,8609
A02	HLA-A*02:67	1000	RLQSLQTYV	0,7431
A02	HLA-A*02:67	1047	YHLMSFPQSA	0,5452
A02	HLA-A*02:67	1048	HLMSFPQSA	0,7385
A02	HLA-A*02:67	1060	VVFLHVTYV	0,6948
A02	HLA-A*02:67	1185	RLNEVAKNL	0,6189
A02	HLA-A*02:67	1192	NLNESLIDL	0,6972
A02	HLA-A*02:67	1220	FIAGLIAIV	0,8207
A02	HLA-A*02:68	109	TLDSKTQSL	0,6782
A02	HLA-A*02:68	133	FQFCNDPFL	0,518
A02	HLA-A*02:68	269	YLQPRTFLL	0,973
A02	HLA-A*02:68	386	KLNDLCFTNV	0,7108
A02	HLA-A*02:68	417	KIADYNYKL	0,909
A02	HLA-A*02:68	424	KLPDDFTGCV	0,5916
A02	HLA-A*02:68	515	FELLHAPATV	0,6033
A02	HLA-A*02:68	610	VLYQDVNCTEV	0,5092
A02	HLA-A*02:68	691	SIIAYTMSL	0,7995
A02	HLA-A*02:68	718	FTISVTTEI	0,5257
A02	HLA-A*02:68	821	LLFNKVTLA	0,7857
A02	HLA-A*02:68	857	GLTVLPPLL	0,6786
A02	HLA-A*02:68	915	VLYENQKLI	0,5378
A02	HLA-A*02:68	975	SVLNDILSRL	0,5124
A02	HLA-A*02:68	976	VLNDILSRL	0,9507
A02	HLA-A*02:68	983	RLDKVEAEV	0,8609
A02	HLA-A*02:68	1000	RLQSLQTYV	0,7431
A02	HLA-A*02:68	1047	YHLMSFPQSA	0,5452
A02	HLA-A*02:68	1048	HLMSFPQSA	0,7385

A02	HLA-A*02:68	1060	VVFLHVTYV	0,6948
A02	HLA-A*02:68	1185	RLNEVAKNL	0,6189
A02	HLA-A*02:68	1192	NLNESLIDL	0,6972
A02	HLA-A*02:68	1220	FIAGLIAIV	0,8207
A02	HLA-A*02:69	109	TLDSKTQSL	0,8988
A02	HLA-A*02:69	269	YLQPRTFLL	0,9932
A02	HLA-A*02:69	386	KLNDLCFTNV	0,8871
A02	HLA-A*02:69	417	KIADYNYKL	0,97
A02	HLA-A*02:69	424	KLPDDFTGCV	0,7417
A02	HLA-A*02:69	515	FELLHAPATV	0,7889
A02	HLA-A*02:69	610	VLYQDVNCTEV	0,6974
A02	HLA-A*02:69	691	SIIAYTMSL	0,9432
A02	HLA-A*02:69	718	FTISVTTEI	0,8216
A02	HLA-A*02:69	821	LLFNKVTLA	0,9443
A02	HLA-A*02:69	857	GLTVLPPLL	0,7856
A02	HLA-A*02:69	915	VLYENQKLI	0,8632
A02	HLA-A*02:69	958	ALNTLVKQL	0,8141
A02	HLA-A*02:69	975	SVLNDILSRL	0,7945
A02	HLA-A*02:69	976	VLNDILSRL	0,9917
A02	HLA-A*02:69	983	RLDKVEAEV	0,9677
A02	HLA-A*02:69	1000	RLQSLQTYV	0,9419
A02	HLA-A*02:69	1047	YHLMSFPQSA	0,7518
A02	HLA-A*02:69	1048	HLMSFPQSA	0,9136
A02	HLA-A*02:69	1060	VVFLHVTYV	0,8865
A02	HLA-A*02:69	1171	GINASVVNI	0,7552
A02	HLA-A*02:69	1185	RLNEVAKNL	0,9099
A02	HLA-A*02:69	1192	NLNESLIDL	0,8952
A02	HLA-A*02:69	1220	FIAGLIAIV	0,9533
A02	HLA-A*02:70	109	TLDSKTQSL	0,6782
A02	HLA-A*02:70	133	FQFCNDPFL	0,518
A02	HLA-A*02:70	269	YLQPRTFLL	0,973
A02	HLA-A*02:70	386	KLNDLCFTNV	0,7108
A02	HLA-A*02:70	417	KIADYNYKL	0,909
A02	HLA-A*02:70	424	KLPDDFTGCV	0,5916
A02	HLA-A*02:70	515	FELLHAPATV	0,6033
A02	HLA-A*02:70	610	VLYQDVNCTEV	0,5092
A02	HLA-A*02:70	691	SIIAYTMSL	0,7995
A02	HLA-A*02:70	718	FTISVTTEI	0,5257
A02	HLA-A*02:70	821	LLFNKVTLA	0,7857
A02	HLA-A*02:70	857	GLTVLPPLL	0,6786
A02	HLA-A*02:70	915	VLYENQKLI	0,5378
A02	HLA-A*02:70	975	SVLNDILSRL	0,5124
A02	HLA-A*02:70	976	VLNDILSRL	0,9507
A02	HLA-A*02:70	983	RLDKVEAEV	0,8609
A02	HLA-A*02:70	1000	RLQSLQTYV	0,7431
A02	HLA-A*02:70	1047	YHLMSFPQSA	0,5452
A02	HLA-A*02:70	1048	HLMSFPQSA	0,7385
A02	HLA-A*02:70	1060	VVFLHVTYV	0,6948
A02	HLA-A*02:70	1185	RLNEVAKNL	0,6189
A02	HLA-A*02:70	1192	NLNESLIDL	0,6972

A02	HLA-A*02:70	1220	FIAGLIAIV	0,8207
A02	HLA-A*02:71	109	TLDSKTQSL	0,6782
A02	HLA-A*02:71	133	FQFCNDPFL	0,518
A02	HLA-A*02:71	269	YLQPRTFLL	0,973
A02	HLA-A*02:71	386	KLNDLCFTNV	0,7108
A02	HLA-A*02:71	417	KIADYNYKL	0,909
A02	HLA-A*02:71	424	KLPDDFTGCV	0,5916
A02	HLA-A*02:71	515	FELLHAPATV	0,6033
A02	HLA-A*02:71	610	VLYQDVNCTEV	0,5092
A02	HLA-A*02:71	691	SIIAYTMSL	0,7995
A02	HLA-A*02:71	718	FTISVTTEI	0,5257
A02	HLA-A*02:71	821	LLFNKVTLA	0,7857
A02	HLA-A*02:71	857	GLTVLPPLL	0,6786
A02	HLA-A*02:71	915	VLYENQKLI	0,5378
A02	HLA-A*02:71	975	SVLNDILSRL	0,5124
A02	HLA-A*02:71	976	VLNDILSRL	0,9507
A02	HLA-A*02:71	983	RLDKVEAEV	0,8609
A02	HLA-A*02:71	1000	RLQSLQTYV	0,7431
A02	HLA-A*02:71	1047	YHLMSFPQSA	0,5452
A02	HLA-A*02:71	1048	HLMSFPQSA	0,7385
A02	HLA-A*02:71	1060	VVFLHVTYV	0,6948
A02	HLA-A*02:71	1185	RLNEVAKNL	0,6189
A02	HLA-A*02:71	1192	NLNESLIDL	0,6972
A02	HLA-A*02:71	1220	FIAGLIAIV	0,8207
A02	HLA-A*02:72	133	FQFCNDPFL	0,7104
A02	HLA-A*02:72	133	FQFCNDPFLGV	0,5205
A02	HLA-A*02:72	269	YLQPRTFLL	0,8955
A02	HLA-A*02:72	417	KIADYNYKL	0,9064
A02	HLA-A*02:72	424	KLPDDFTGCV	0,5686
A02	HLA-A*02:72	612	YQDVNCTEV	0,5256
A02	HLA-A*02:72	691	SIIAYTMSL	0,847
A02	HLA-A*02:72	712	IAIPTNFTI	0,5224
A02	HLA-A*02:72	718	FTISVTTEI	0,8184
A02	HLA-A*02:72	721	SVTTEILPV	0,5642
A02	HLA-A*02:72	821	LLFNKVTLA	0,5419
A02	HLA-A*02:72	894	LQIPFAMQM	0,6147
A02	HLA-A*02:72	976	VLNDILSRL	0,8346
A02	HLA-A*02:72	983	RLDKVEAEV	0,6135
A02	HLA-A*02:72	1048	HLMSFPQSA	0,5289
A02	HLA-A*02:72	1060	VVFLHVTYV	0,7813
A02	HLA-A*02:72	1136	TVYDPLQPEL	0,5968
A02	HLA-A*02:72	1220	FIAGLIAIV	0,81
A02	HLA-A*02:74	109	TLDSKTQSL	0,6782
A02	HLA-A*02:74	133	FQFCNDPFL	0,518
A02	HLA-A*02:74	269	YLQPRTFLL	0,973
A02	HLA-A*02:74	386	KLNDLCFTNV	0,7108
A02	HLA-A*02:74	417	KIADYNYKL	0,909
A02	HLA-A*02:74	424	KLPDDFTGCV	0,5916
A02	HLA-A*02:74	515	FELLHAPATV	0,6033
A02	HLA-A*02:74	610	VLYQDVNCTEV	0,5092

A02	HLA-A*02:74	691	SIIAYTMSL	0,7995
A02	HLA-A*02:74	718	FTISVTTEI	0,5257
A02	HLA-A*02:74	821	LLFNKVTLA	0,7857
A02	HLA-A*02:74	857	GLTVLPPLL	0,6786
A02	HLA-A*02:74	915	VLYENQKLI	0,5378
A02	HLA-A*02:74	975	SVLNDILSRL	0,5124
A02	HLA-A*02:74	976	VLNDILSRL	0,9507
A02	HLA-A*02:74	983	RLDKVEAEV	0,8609
A02	HLA-A*02:74	1000	RLQSLQTYV	0,7431
A02	HLA-A*02:74	1047	YHLMSFPQSA	0,5452
A02	HLA-A*02:74	1048	HLMSFPQSA	0,7385
A02	HLA-A*02:74	1060	VVFLHVTYV	0,6948
A02	HLA-A*02:74	1185	RLNEVAKNL	0,6189
A02	HLA-A*02:74	1192	NLNESLIDL	0,6972
A02	HLA-A*02:74	1220	FIAGLIAIV	0,8207
A02	HLA-A*02:75	109	TLDSKTQSL	0,6782
A02	HLA-A*02:75	133	FQFCNDPFL	0,518
A02	HLA-A*02:75	269	YLQPRTFLL	0,973
A02	HLA-A*02:75	386	KLNDLCFTNV	0,7108
A02	HLA-A*02:75	417	KIADYNYKL	0,909
A02	HLA-A*02:75	424	KLPDDFTGCV	0,5916
A02	HLA-A*02:75	515	FELLHAPATV	0,6033
A02	HLA-A*02:75	610	VLYQDVNCTEV	0,5092
A02	HLA-A*02:75	691	SIIAYTMSL	0,7995
A02	HLA-A*02:75	718	FTISVTTEI	0,5257
A02	HLA-A*02:75	821	LLFNKVTLA	0,7857
A02	HLA-A*02:75	857	GLTVLPPLL	0,6786
A02	HLA-A*02:75	915	VLYENQKLI	0,5378
A02	HLA-A*02:75	975	SVLNDILSRL	0,5124
A02	HLA-A*02:75	976	VLNDILSRL	0,9507
A02	HLA-A*02:75	983	RLDKVEAEV	0,8609
A02	HLA-A*02:75	1000	RLQSLQTYV	0,7431
A02	HLA-A*02:75	1047	YHLMSFPQSA	0,5452
A02	HLA-A*02:75	1048	HLMSFPQSA	0,7385
A02	HLA-A*02:75	1060	VVFLHVTYV	0,6948
A02	HLA-A*02:75	1185	RLNEVAKNL	0,6189
A02	HLA-A*02:75	1192	NLNESLIDL	0,6972
A02	HLA-A*02:75	1220	FIAGLIAIV	0,8207
A02	HLA-A*02:77	109	TLDSKTQSL	0,6782
A02	HLA-A*02:77	133	FQFCNDPFL	0,518
A02	HLA-A*02:77	269	YLQPRTFLL	0,973
A02	HLA-A*02:77	386	KLNDLCFTNV	0,7108
A02	HLA-A*02:77	417	KIADYNYKL	0,909
A02	HLA-A*02:77	424	KLPDDFTGCV	0,5916
A02	HLA-A*02:77	515	FELLHAPATV	0,6033
A02	HLA-A*02:77	610	VLYQDVNCTEV	0,5092
A02	HLA-A*02:77	691	SIIAYTMSL	0,7995
A02	HLA-A*02:77	718	FTISVTTEI	0,5257
A02	HLA-A*02:77	821	LLFNKVTLA	0,7857
A02	HLA-A*02:77	857	GLTVLPPLL	0,6786

A02	HLA-A*02:77	915	VLYENQKLI	0,5378
A02	HLA-A*02:77	975	SVLNDILSRL	0,5124
A02	HLA-A*02:77	976	VLNDILSRL	0,9507
A02	HLA-A*02:77	983	RLDKVEAEV	0,8609
A02	HLA-A*02:77	1000	RLQSLQTYV	0,7431
A02	HLA-A*02:77	1047	YHLMSFPQSA	0,5452
A02	HLA-A*02:77	1048	HLMSFPQSA	0,7385
A02	HLA-A*02:77	1060	VVFLHVTYV	0,6948
A02	HLA-A*02:77	1185	RLNEVAKNL	0,6189
A02	HLA-A*02:77	1192	NLNESLIDL	0,6972
A02	HLA-A*02:77	1220	FIAGLIAIV	0,8207
A02	HLA-A*02:78	28	YTNSFTRGV	0,6301
A02	HLA-A*02:78	62	VTWFHAIHV	0,7122
A02	HLA-A*02:78	269	YLQPRTFLL	0,5176
A02	HLA-A*02:78	417	KIADYNYKL	0,8511
A02	HLA-A*02:78	634	RVYSTGSNV	0,6719
A02	HLA-A*02:78	691	SIIAYTMSL	0,7755
A02	HLA-A*02:78	712	IAIPTNFTI	0,5958
A02	HLA-A*02:78	718	FTISVTTEI	0,6858
A02	HLA-A*02:78	721	SVTTEILPV	0,5092
A02	HLA-A*02:78	777	NTQEVFAQV	0,5878
A02	HLA-A*02:78	1060	VVFLHVTYV	0,7321
A02	HLA-A*02:78	1136	TVYDPLQPEL	0,5843
A02	HLA-A*02:78	1171	GINASVVNI	0,5371
A02	HLA-A*02:79	133	FQFCNDPFL	0,7104
A02	HLA-A*02:79	133	FQFCNDPFLGV	0,5205
A02	HLA-A*02:79	269	YLQPRTFLL	0,8955
A02	HLA-A*02:79	417	KIADYNYKL	0,9064
A02	HLA-A*02:79	424	KLPDDFTGCV	0,5686
A02	HLA-A*02:79	612	YQDVNCTEV	0,5256
A02	HLA-A*02:79	691	SIIAYTMSL	0,847
A02	HLA-A*02:79	712	IAIPTNFTI	0,5224
A02	HLA-A*02:79	718	FTISVTTEI	0,8184
A02	HLA-A*02:79	721	SVTTEILPV	0,5642
A02	HLA-A*02:79	821	LLFNKVTLA	0,5419
A02	HLA-A*02:79	894	LQIPFAMQM	0,6147
A02	HLA-A*02:79	976	VLNDILSRL	0,8346
A02	HLA-A*02:79	983	RLDKVEAEV	0,6135
A02	HLA-A*02:79	1048	HLMSFPQSA	0,5289
A02	HLA-A*02:79	1060	VVFLHVTYV	0,7813
A02	HLA-A*02:79	1136	TVYDPLQPEL	0,5968
A02	HLA-A*02:79	1220	FIAGLIAIV	0,81
A02	HLA-A*02:85	109	TLDSKTQSL	0,6782
A02	HLA-A*02:85	133	FQFCNDPFL	0,518
A02	HLA-A*02:85	269	YLQPRTFLL	0,973
A02	HLA-A*02:85	386	KLNDLCFTNV	0,7108
A02	HLA-A*02:85	417	KIADYNYKL	0,909
A02	HLA-A*02:85	424	KLPDDFTGCV	0,5916
A02	HLA-A*02:85	515	FELLHAPATV	0,6033
A02	HLA-A*02:85	610	VLYQDVNCTEV	0,5092

A02	HLA-A*02:85	691	SIIAYTMSL	0,7995
A02	HLA-A*02:85	718	FTISVTTEI	0,5257
A02	HLA-A*02:85	821	LLFNKVTLA	0,7857
A02	HLA-A*02:85	857	GLTVLPPLL	0,6786
A02	HLA-A*02:85	915	VLYENQKLI	0,5378
A02	HLA-A*02:85	975	SVLNDILSRL	0,5124
A02	HLA-A*02:85	976	VLNDILSRL	0,9507
A02	HLA-A*02:85	983	RLDKVEAEV	0,8609
A02	HLA-A*02:85	1000	RLQSLQTYV	0,7431
A02	HLA-A*02:85	1047	YHLMSFPQSA	0,5452
A02	HLA-A*02:85	1048	HLMSFPQSA	0,7385
A02	HLA-A*02:85	1060	VVFLHVTYV	0,6948
A02	HLA-A*02:85	1185	RLNEVAKNL	0,6189
A02	HLA-A*02:85	1192	NLNESLIDL	0,6972
A02	HLA-A*02:85	1220	FIAGLIAIV	0,8207
A02	HLA-A*02:86	109	TLDSKTQSL	0,6782
A02	HLA-A*02:86	133	FQFCNDPFL	0,518
A02	HLA-A*02:86	269	YLQPRTFLL	0,973
A02	HLA-A*02:86	386	KLNDLCFTNV	0,7108
A02	HLA-A*02:86	417	KIADYNYKL	0,909
A02	HLA-A*02:86	424	KLPDDFTGCV	0,5916
A02	HLA-A*02:86	515	FELLHAPATV	0,6033
A02	HLA-A*02:86	610	VLYQDVNCTEV	0,5092
A02	HLA-A*02:86	691	SIIAYTMSL	0,7995
A02	HLA-A*02:86	718	FTISVTTEI	0,5257
A02	HLA-A*02:86	821	LLFNKVTLA	0,7857
A02	HLA-A*02:86	857	GLTVLPPLL	0,6786
A02	HLA-A*02:86	915	VLYENQKLI	0,5378
A02	HLA-A*02:86	975	SVLNDILSRL	0,5124
A02	HLA-A*02:86	976	VLNDILSRL	0,9507
A02	HLA-A*02:86	983	RLDKVEAEV	0,8609
A02	HLA-A*02:86	1000	RLQSLQTYV	0,7431
A02	HLA-A*02:86	1047	YHLMSFPQSA	0,5452
A02	HLA-A*02:86	1048	HLMSFPQSA	0,7385
A02	HLA-A*02:86	1060	VVFLHVTYV	0,6948
A02	HLA-A*02:86	1185	RLNEVAKNL	0,6189
A02	HLA-A*02:86	1192	NLNESLIDL	0,6972
A02	HLA-A*02:86	1220	FIAGLIAIV	0,8207
A02	HLA-A*68:02	28	YTNSFTRGV	0,845
A02	HLA-A*68:02	122	NATNVVIKV	0,7315
A02	HLA-A*68:02	258	WTAGAAAYYV	0,5761
A02	HLA-A*68:02	259	TAGAAAYYV	0,5379
A02	HLA-A*68:02	340	EVFNATRFA	0,7317
A02	HLA-A*68:02	394	NVYADSFVI	0,6993
A02	HLA-A*68:02	495	YGFQPTNGV	0,5183
A02	HLA-A*68:02	568	DIADTTDAV	0,7805
A02	HLA-A*68:02	603	NTSNQVAVL	0,5558
A02	HLA-A*68:02	691	SIIAYTMSL	0,6292
A02	HLA-A*68:02	704	SVAYSNNSI	0,7306
A02	HLA-A*68:02	717	NFTISVTTEI	0,6193

A02	HLA-A*68:02	718	FTISVTTEI	0,9208
A02	HLA-A*68:02	734	TSVDCTMYI	0,5115
A02	HLA-A*68:02	777	NTQEVFAQV	0,9221
A02	HLA-A*68:02	780	EVFAQVKQI	0,6531
A02	HLA-A*68:02	869	MIAQYTSAL	0,6202
A02	HLA-A*68:02	886	WTFGAGAAL	0,732
A02	HLA-A*68:02	907	NGIGVTQNV	0,5186
A02	HLA-A*68:02	940	STASALGKL	0,5142
A02	HLA-A*68:02	1060	VVFLHVTYV	0,6649
A02	HLA-A*68:02	1095	FVSNGTHWFV	0,5041
A02	HLA-A*68:02	1128	VVIGIVNNTV	0,5513
A02	HLA-A*68:02	1136	TVYDPLQPEL	0,6066
A02	HLA-A*68:02	1168	DISGINASV	0,8721
A02	HLA-A*68:02	1220	FIAGLIAIV	0,636
A02	HLA-A*68:27	28	YTNSFTRGV	0,845
A02	HLA-A*68:27	122	NATNVVIKV	0,7315
A02	HLA-A*68:27	258	WTAGAAAYYV	0,5761
A02	HLA-A*68:27	259	TAGAAAYYV	0,5379
A02	HLA-A*68:27	340	EVFNATRFA	0,7317
A02	HLA-A*68:27	394	NVYADSFVI	0,6993
A02	HLA-A*68:27	495	YGFQPTNGV	0,5183
A02	HLA-A*68:27	568	DIADTTDAV	0,7805
A02	HLA-A*68:27	603	NTSNQVAVL	0,5558
A02	HLA-A*68:27	691	SIIAYTMSL	0,6292
A02	HLA-A*68:27	704	SVAYSNNSI	0,7306
A02	HLA-A*68:27	717	NFTISVTTEI	0,6193
A02	HLA-A*68:27	718	FTISVTTEI	0,9208
A02	HLA-A*68:27	734	TSVDCTMYI	0,5115
A02	HLA-A*68:27	777	NTQEVFAQV	0,9221
A02	HLA-A*68:27	780	EVFAQVKQI	0,6531
A02	HLA-A*68:27	869	MIAQYTSAL	0,6202
A02	HLA-A*68:27	886	WTFGAGAAL	0,732
A02	HLA-A*68:27	907	NGIGVTQNV	0,5186
A02	HLA-A*68:27	940	STASALGKL	0,5142
A02	HLA-A*68:27	1060	VVFLHVTYV	0,6649
A02	HLA-A*68:27	1095	FVSNGTHWFV	0,5041
A02	HLA-A*68:27	1128	VVIGIVNNTV	0,5513
A02	HLA-A*68:27	1136	TVYDPLQPEL	0,6066
A02	HLA-A*68:27	1168	DISGINASV	0,8721
A02	HLA-A*68:27	1220	FIAGLIAIV	0,636
A02	HLA-A*68:28	28	YTNSFTRGV	0,5476
A02	HLA-A*68:28	568	DIADTTDAV	0,6854
A02	HLA-A*68:28	718	FTISVTTEI	0,6638
A02	HLA-A*68:28	777	NTQEVFAQV	0,7667
A02	HLA-A*68:28	1168	DISGINASV	0,5957
A02	HLA-A*69:01	28	YTNSFTRGV	0,6754
A02	HLA-A*69:01	62	VTWFHAIHV	0,5639
A02	HLA-A*69:01	122	NATNVVIKV	0,6388
A02	HLA-A*69:01	394	NVYADSFVI	0,702
A02	HLA-A*69:01	568	DIADTTDAV	0,62

A02	HLA-A*69:01	691	SIIAYTMSL	0,6455
A02	HLA-A*69:01	718	FTISVTTEI	0,8233
A02	HLA-A*69:01	777	NTQEVFAQV	0,8811
A02	HLA-A*69:01	780	EVFAQVKQI	0,5161
A02	HLA-A*69:01	886	WTFGAGAAL	0,6113
A02	HLA-A*69:01	1060	VVFLHVTYV	0,6659
A02	HLA-A*69:01	1136	TVYDPLQPEL	0,6066
A02	HLA-A*69:01	1168	DISGINASV	0,7179
A02	HLA-A*69:01	1220	FIAGLIAIV	0,5133
A03	HLA-A*03:01	35	GVYYPDKVFR	0,568
A03	HLA-A*03:01	41	KVFRSSVLH	0,6944
A03	HLA-A*03:01	89	GVYFASTEK	0,9487
A03	HLA-A*03:01	142	GVYYHKNNK	0,8871
A03	HLA-A*03:01	269	YLQPRTFLLK	0,5577
A03	HLA-A*03:01	302	TLKSFTVEK	0,8988
A03	HLA-A*03:01	311	GIYQTSNFR	0,6304
A03	HLA-A*03:01	349	SVYAWNRKR	0,6333
A03	HLA-A*03:01	367	VLYNSASFSTFK	0,5358
A03	HLA-A*03:01	378	KCYGVSPTK	0,5631
A03	HLA-A*03:01	408	RQIAPGQTGK	0,8957
A03	HLA-A*03:01	409	QIAPGQTGK	0,6982
A03	HLA-A*03:01	454	RLFRKSNLK	0,9589
A03	HLA-A*03:01	529	KSTNLVKNK	0,5122
A03	HLA-A*03:01	724	TEILPVSMTK	0,6838
A03	HLA-A*03:01	786	KQIYKTPPIK	0,8503
A03	HLA-A*03:01	787	QIYKTPPIK	0,8934
A03	HLA-A*03:01	805	ILPDPSKPSK	0,5588
A03	HLA-A*03:01	826	VTLADAGFIK	0,5395
A03	HLA-A*03:01	827	TLADAGFIK	0,7267
A03	HLA-A*03:01	924	ANQFNSAIGK	0,5091
A03	HLA-A*03:01	939	SSTASALGK	0,5175
A03	HLA-A*03:01	1019	RASANLAATK	0,5521
A03	HLA-A*03:01	1020	ASANLAATK	0,7894
A03	HLA-A*03:01	1064	HVTYVPAQEK	0,6951
A03	HLA-A*03:01	1065	VTYVPAQEK	0,9087
A03	HLA-A*03:01	1099	GTHWFVTQR	0,5011
A03	HLA-A*03:01	1196	SLIDLQELGK	0,6419
A03	HLA-A*03:01	1264	VLKGVKLHY	0,6718
A03	HLA-A*03:02	35	GVYYPDKVFR	0,6162
A03	HLA-A*03:02	89	GVYFASTEK	0,8282
A03	HLA-A*03:02	142	GVYYHKNNK	0,6008
A03	HLA-A*03:02	269	YLQPRTFLLK	0,5505
A03	HLA-A*03:02	292	ALDPLSETK	0,7332
A03	HLA-A*03:02	302	TLKSFTVEK	0,8355
A03	HLA-A*03:02	311	GIYQTSNFR	0,6485
A03	HLA-A*03:02	349	SVYAWNRKR	0,5515
A03	HLA-A*03:02	408	RQIAPGQTGK	0,607
A03	HLA-A*03:02	454	RLFRKSNLK	0,8224
A03	HLA-A*03:02	724	TEILPVSMTK	0,5896
A03	HLA-A*03:02	725	EILPVSMTK	0,5252

A03	HLA-A*03:02	787	QIYKTPPIK	0,6603
A03	HLA-A*03:02	805	ILPDPSKPSK	0,52
A03	HLA-A*03:02	826	VTLADAGFIK	0,5723
A03	HLA-A*03:02	827	TLADAGFIK	0,8003
A03	HLA-A*03:02	975	SVLNDILSR	0,6428
A03	HLA-A*03:02	1020	ASANLAATK	0,6797
A03	HLA-A*03:02	1065	VTYVPAQEK	0,744
A03	HLA-A*03:02	1099	GTHWFVTQR	0,5647
A03	HLA-A*03:02	1196	SLIDLQELGK	0,5484
A03	HLA-A*03:04	35	GVYYPDKVFR	0,568
A03	HLA-A*03:04	41	KVFRSSVLH	0,6944
A03	HLA-A*03:04	89	GVYFASTEK	0,9487
A03	HLA-A*03:04	142	GVYYHKNNK	0,8871
A03	HLA-A*03:04	269	YLQPRTFLLK	0,5577
A03	HLA-A*03:04	302	TLKSFTVEK	0,8988
A03	HLA-A*03:04	311	GIYQTSNFR	0,6304
A03	HLA-A*03:04	349	SVYAWNRKR	0,6333
A03	HLA-A*03:04	367	VLYNSASFSTFK	0,5358
A03	HLA-A*03:04	378	KCYGVSPTK	0,5631
A03	HLA-A*03:04	408	RQIAPGQTGK	0,8957
A03	HLA-A*03:04	409	QIAPGQTGK	0,6982
A03	HLA-A*03:04	454	RLFRKSNLK	0,9589
A03	HLA-A*03:04	529	KSTNLVKNK	0,5122
A03	HLA-A*03:04	724	TEILPVSMTK	0,6838
A03	HLA-A*03:04	786	KQIYKTPPIK	0,8503
A03	HLA-A*03:04	787	QIYKTPPIK	0,8934
A03	HLA-A*03:04	805	ILPDPSKPSK	0,5588
A03	HLA-A*03:04	826	VTLADAGFIK	0,5395
A03	HLA-A*03:04	827	TLADAGFIK	0,7267
A03	HLA-A*03:04	924	ANQFNSAIGK	0,5091
A03	HLA-A*03:04	939	SSTASALGK	0,5175
A03	HLA-A*03:04	1019	RASANLAATK	0,5521
A03	HLA-A*03:04	1020	ASANLAATK	0,7894
A03	HLA-A*03:04	1064	HVTYVPAQEK	0,6951
A03	HLA-A*03:04	1065	VTYVPAQEK	0,9087
A03	HLA-A*03:04	1099	GTHWFVTQR	0,5011
A03	HLA-A*03:04	1196	SLIDLQELGK	0,6419
A03	HLA-A*03:04	1264	VLKGVKLHY	0,6718
A03	HLA-A*03:05	35	GVYYPDKVFR	0,568
A03	HLA-A*03:05	41	KVFRSSVLH	0,6944
A03	HLA-A*03:05	89	GVYFASTEK	0,9487
A03	HLA-A*03:05	142	GVYYHKNNK	0,8871
A03	HLA-A*03:05	269	YLQPRTFLLK	0,5577
A03	HLA-A*03:05	302	TLKSFTVEK	0,8988
A03	HLA-A*03:05	311	GIYQTSNFR	0,6304
A03	HLA-A*03:05	349	SVYAWNRKR	0,6333
A03	HLA-A*03:05	367	VLYNSASFSTFK	0,5358
A03	HLA-A*03:05	378	KCYGVSPTK	0,5631
A03	HLA-A*03:05	408	RQIAPGQTGK	0,8957
A03	HLA-A*03:05	409	QIAPGQTGK	0,6982

A03	HLA-A*03:05	454	RLFRKSNLK	0,9589
A03	HLA-A*03:05	529	KSTNLVKNK	0,5122
A03	HLA-A*03:05	724	TEILPVSMTK	0,6838
A03	HLA-A*03:05	786	KQIYKTPPIK	0,8503
A03	HLA-A*03:05	787	QIYKTPPIK	0,8934
A03	HLA-A*03:05	805	ILPDPSKPSK	0,5588
A03	HLA-A*03:05	826	VTLADAGFIK	0,5395
A03	HLA-A*03:05	827	TLADAGFIK	0,7267
A03	HLA-A*03:05	924	ANQFNSAIGK	0,5091
A03	HLA-A*03:05	939	SSTASALGK	0,5175
A03	HLA-A*03:05	1019	RASANLAATK	0,5521
A03	HLA-A*03:05	1020	ASANLAATK	0,7894
A03	HLA-A*03:05	1064	HVTYVPAQEK	0,6951
A03	HLA-A*03:05	1065	VTYVPAQEK	0,9087
A03	HLA-A*03:05	1099	GTHWFVTQR	0,5011
A03	HLA-A*03:05	1196	SLIDLQELGK	0,6419
A03	HLA-A*03:05	1264	VLKGVKLHY	0,6718
A03	HLA-A*03:06	35	GVYYPDKVFR	0,568
A03	HLA-A*03:06	41	KVFRSSVLH	0,6944
A03	HLA-A*03:06	89	GVYFASTEK	0,9487
A03	HLA-A*03:06	142	GVYYHKNNK	0,8871
A03	HLA-A*03:06	269	YLQPRTFLLK	0,5577
A03	HLA-A*03:06	302	TLKSFTVEK	0,8988
A03	HLA-A*03:06	311	GIYQTSNFR	0,6304
A03	HLA-A*03:06	349	SVYAWNRKR	0,6333
A03	HLA-A*03:06	367	VLYNSASFSTFK	0,5358
A03	HLA-A*03:06	378	KCYGVSPTK	0,5631
A03	HLA-A*03:06	408	RQIAPGQTGK	0,8957
A03	HLA-A*03:06	409	QIAPGQTGK	0,6982
A03	HLA-A*03:06	454	RLFRKSNLK	0,9589
A03	HLA-A*03:06	529	KSTNLVKNK	0,5122
A03	HLA-A*03:06	724	TEILPVSMTK	0,6838
A03	HLA-A*03:06	786	KQIYKTPPIK	0,8503
A03	HLA-A*03:06	787	QIYKTPPIK	0,8934
A03	HLA-A*03:06	805	ILPDPSKPSK	0,5588
A03	HLA-A*03:06	826	VTLADAGFIK	0,5395
A03	HLA-A*03:06	827	TLADAGFIK	0,7267
A03	HLA-A*03:06	924	ANQFNSAIGK	0,5091
A03	HLA-A*03:06	939	SSTASALGK	0,5175
A03	HLA-A*03:06	1019	RASANLAATK	0,5521
A03	HLA-A*03:06	1020	ASANLAATK	0,7894
A03	HLA-A*03:06	1064	HVTYVPAQEK	0,6951
A03	HLA-A*03:06	1065	VTYVPAQEK	0,9087
A03	HLA-A*03:06	1099	GTHWFVTQR	0,5011
A03	HLA-A*03:06	1196	SLIDLQELGK	0,6419
A03	HLA-A*03:06	1264	VLKGVKLHY	0,6718
A03	HLA-A*03:07	35	GVYYPDKVFR	0,7348
A03	HLA-A*03:07	89	GVYFASTEK	0,8648
A03	HLA-A*03:07	142	GVYYHKNNK	0,6702
A03	HLA-A*03:07	292	ALDPLSETK	0,6955

A03	HLA-A*03:07	302	TLKSFTVEK	0,8407
A03	HLA-A*03:07	311	GIYQTSNFR	0,7033
A03	HLA-A*03:07	349	SVYAWNRKR	0,6804
A03	HLA-A*03:07	370	NSASFSTFK	0,5695
A03	HLA-A*03:07	408	RQIAPGQTGK	0,6507
A03	HLA-A*03:07	454	RLFRKSNLK	0,8435
A03	HLA-A*03:07	458	KSNLKPFER	0,5539
A03	HLA-A*03:07	724	TEILPVSMTK	0,6479
A03	HLA-A*03:07	725	EILPVSMTK	0,6008
A03	HLA-A*03:07	786	KQIYKTPPIK	0,5627
A03	HLA-A*03:07	787	QIYKTPPIK	0,757
A03	HLA-A*03:07	805	ILPDPSKPSK	0,5158
A03	HLA-A*03:07	826	VTLADAGFIK	0,6103
A03	HLA-A*03:07	827	TLADAGFIK	0,8004
A03	HLA-A*03:07	975	SVLNDILSR	0,744
A03	HLA-A*03:07	1020	ASANLAATK	0,7263
A03	HLA-A*03:07	1064	HVTYVPAQEK	0,5071
A03	HLA-A*03:07	1065	VTYVPAQEK	0,8254
A03	HLA-A*03:07	1099	GTHWFVTQR	0,6685
A03	HLA-A*03:07	1196	SLIDLQELGK	0,6149
A03	HLA-A*03:08	35	GVYYPDKVFR	0,5777
A03	HLA-A*03:08	41	KVFRSSVLH	0,5336
A03	HLA-A*03:08	89	GVYFASTEK	0,8234
A03	HLA-A*03:08	142	GVYYHKNNK	0,7338
A03	HLA-A*03:08	302	TLKSFTVEK	0,7823
A03	HLA-A*03:08	311	GIYQTSNFR	0,623
A03	HLA-A*03:08	349	SVYAWNRKR	0,6585
A03	HLA-A*03:08	408	RQIAPGQTGK	0,7314
A03	HLA-A*03:08	454	RLFRKSNLK	0,9008
A03	HLA-A*03:08	786	KQIYKTPPIK	0,6142
A03	HLA-A*03:08	787	QIYKTPPIK	0,679
A03	HLA-A*03:08	1020	ASANLAATK	0,5793
A03	HLA-A*03:08	1065	VTYVPAQEK	0,7028
A03	HLA-A*03:08	1099	GTHWFVTQR	0,5336
A03	HLA-A*03:10	35	GVYYPDKVFR	0,6162
A03	HLA-A*03:10	89	GVYFASTEK	0,8282
A03	HLA-A*03:10	142	GVYYHKNNK	0,6008
A03	HLA-A*03:10	269	YLQPRTFLLK	0,5505
A03	HLA-A*03:10	292	ALDPLSETK	0,7332
A03	HLA-A*03:10	302	TLKSFTVEK	0,8355
A03	HLA-A*03:10	311	GIYQTSNFR	0,6485
A03	HLA-A*03:10	349	SVYAWNRKR	0,5515
A03	HLA-A*03:10	408	RQIAPGQTGK	0,607
A03	HLA-A*03:10	454	RLFRKSNLK	0,8224
A03	HLA-A*03:10	724	TEILPVSMTK	0,5896
A03	HLA-A*03:10	725	EILPVSMTK	0,5252
A03	HLA-A*03:10	787	QIYKTPPIK	0,6603
A03	HLA-A*03:10	805	ILPDPSKPSK	0,52
A03	HLA-A*03:10	826	VTLADAGFIK	0,5723
A03	HLA-A*03:10	827	TLADAGFIK	0,8003

A03	HLA-A*03:10	975	SVLNDILSR	0,6428
A03	HLA-A*03:10	1020	ASANLAATK	0,6797
A03	HLA-A*03:10	1065	VTYVPAQEK	0,744
A03	HLA-A*03:10	1099	GTHWFVTQR	0,5647
A03	HLA-A*03:10	1196	SLIDLQELGK	0,5484
A03	HLA-A*03:12	35	GVYYPDKVFR	0,6548
A03	HLA-A*03:12	41	KVFRSSVLH	0,7788
A03	HLA-A*03:12	69	HVSGTNGTK	0,5995
A03	HLA-A*03:12	89	GVYFASTEK	0,9495
A03	HLA-A*03:12	142	GVYYHKNNK	0,8976
A03	HLA-A*03:12	302	TLKSFTVEK	0,8641
A03	HLA-A*03:12	311	GIYQTSNFR	0,6653
A03	HLA-A*03:12	348	ASVYAWNRK	0,6546
A03	HLA-A*03:12	349	SVYAWNRKR	0,7468
A03	HLA-A*03:12	370	NSASFSTFK	0,6426
A03	HLA-A*03:12	375	STFKCYGVSPTK	0,5581
A03	HLA-A*03:12	378	KCYGVSPTK	0,6783
A03	HLA-A*03:12	408	RQIAPGQTGK	0,8931
A03	HLA-A*03:12	409	QIAPGQTGK	0,7544
A03	HLA-A*03:12	454	RLFRKSNLK	0,9223
A03	HLA-A*03:12	458	KSNLKPFER	0,5408
A03	HLA-A*03:12	529	KSTNLVKNK	0,7666
A03	HLA-A*03:12	550	GVLTESNKK	0,6481
A03	HLA-A*03:12	724	TEILPVSMTK	0,6899
A03	HLA-A*03:12	725	EILPVSMTK	0,7052
A03	HLA-A*03:12	786	KQIYKTPPIK	0,7729
A03	HLA-A*03:12	787	QIYKTPPIK	0,8972
A03	HLA-A*03:12	803	SQILPDPSK	0,5075
A03	HLA-A*03:12	826	VTLADAGFIK	0,5973
A03	HLA-A*03:12	827	TLADAGFIK	0,6931
A03	HLA-A*03:12	845	AARDLICAQK	0,5375
A03	HLA-A*03:12	924	ANQFNSAIGK	0,679
A03	HLA-A*03:12	925	NQFNSAIGK	0,6061
A03	HLA-A*03:12	939	SSTASALGK	0,7158
A03	HLA-A*03:12	956	AQALNTLVK	0,5799
A03	HLA-A*03:12	975	SVLNDILSR	0,7042
A03	HLA-A*03:12	1019	RASANLAATK	0,6895
A03	HLA-A*03:12	1020	ASANLAATK	0,8887
A03	HLA-A*03:12	1064	HVTYVPAQEK	0,7577
A03	HLA-A*03:12	1065	VTYVPAQEK	0,9408
A03	HLA-A*03:12	1099	GTHWFVTQR	0,7035
A03	HLA-A*03:12	1196	SLIDLQELGK	0,5515
A03	HLA-A*03:12	1264	VLKGVKLHY	0,6239
A03	HLA-A*03:13	35	GVYYPDKVFR	0,568
A03	HLA-A*03:13	41	KVFRSSVLH	0,6944
A03	HLA-A*03:13	89	GVYFASTEK	0,9487
A03	HLA-A*03:13	142	GVYYHKNNK	0,8871
A03	HLA-A*03:13	269	YLQPRTFLLK	0,5577
A03	HLA-A*03:13	302	TLKSFTVEK	0,8988
A03	HLA-A*03:13	311	GIYQTSNFR	0,6304

A03	HLA-A*03:13	349	SVYAWNRKR	0,6333
A03	HLA-A*03:13	367	VLYNSASFSTFK	0,5358
A03	HLA-A*03:13	378	KCYGVSPTK	0,5631
A03	HLA-A*03:13	408	RQIAPGQTGK	0,8957
A03	HLA-A*03:13	409	QIAPGQTGK	0,6982
A03	HLA-A*03:13	454	RLFRKSNLK	0,9589
A03	HLA-A*03:13	529	KSTNLVKNK	0,5122
A03	HLA-A*03:13	724	TEILPVSMTK	0,6838
A03	HLA-A*03:13	786	KQIYKTPPIK	0,8503
A03	HLA-A*03:13	787	QIYKTPPIK	0,8934
A03	HLA-A*03:13	805	ILPDPSKPSK	0,5588
A03	HLA-A*03:13	826	VTLADAGFIK	0,5395
A03	HLA-A*03:13	827	TLADAGFIK	0,7267
A03	HLA-A*03:13	924	ANQFNSAIGK	0,5091
A03	HLA-A*03:13	939	SSTASALGK	0,5175
A03	HLA-A*03:13	1019	RASANLAATK	0,5521
A03	HLA-A*03:13	1020	ASANLAATK	0,7894
A03	HLA-A*03:13	1064	HVTYVPAQEK	0,6951
A03	HLA-A*03:13	1065	VTYVPAQEK	0,9087
A03	HLA-A*03:13	1099	GTHWFVTQR	0,5011
A03	HLA-A*03:13	1196	SLIDLQELGK	0,6419
A03	HLA-A*03:13	1264	VLKGVKLHY	0,6718
A03	HLA-A*03:14	35	GVYYPDKVFR	0,568
A03	HLA-A*03:14	41	KVFRSSVLH	0,6944
A03	HLA-A*03:14	89	GVYFASTEK	0,9487
A03	HLA-A*03:14	142	GVYYHKNNK	0,8871
A03	HLA-A*03:14	269	YLQPRTFLLK	0,5577
A03	HLA-A*03:14	302	TLKSFTVEK	0,8988
A03	HLA-A*03:14	311	GIYQTSNFR	0,6304
A03	HLA-A*03:14	349	SVYAWNRKR	0,6333
A03	HLA-A*03:14	367	VLYNSASFSTFK	0,5358
A03	HLA-A*03:14	378	KCYGVSPTK	0,5631
A03	HLA-A*03:14	408	RQIAPGQTGK	0,8957
A03	HLA-A*03:14	409	QIAPGQTGK	0,6982
A03	HLA-A*03:14	454	RLFRKSNLK	0,9589
A03	HLA-A*03:14	529	KSTNLVKNK	0,5122
A03	HLA-A*03:14	724	TEILPVSMTK	0,6838
A03	HLA-A*03:14	786	KQIYKTPPIK	0,8503
A03	HLA-A*03:14	787	QIYKTPPIK	0,8934
A03	HLA-A*03:14	805	ILPDPSKPSK	0,5588
A03	HLA-A*03:14	826	VTLADAGFIK	0,5395
A03	HLA-A*03:14	827	TLADAGFIK	0,7267
A03	HLA-A*03:14	924	ANQFNSAIGK	0,5091
A03	HLA-A*03:14	939	SSTASALGK	0,5175
A03	HLA-A*03:14	1019	RASANLAATK	0,5521
A03	HLA-A*03:14	1020	ASANLAATK	0,7894
A03	HLA-A*03:14	1064	HVTYVPAQEK	0,6951
A03	HLA-A*03:14	1065	VTYVPAQEK	0,9087
A03	HLA-A*03:14	1099	GTHWFVTQR	0,5011
A03	HLA-A*03:14	1196	SLIDLQELGK	0,6419

A03	HLA-A*03:14	1264	VLKGVKLHY	0,6718
A03	HLA-A*03:16	35	GVYYPDKVFR	0,568
A03	HLA-A*03:16	41	KVFRSSVLH	0,6944
A03	HLA-A*03:16	89	GVYFASTEK	0,9487
A03	HLA-A*03:16	142	GVYYHKNNK	0,8871
A03	HLA-A*03:16	269	YLQPRTFLLK	0,5577
A03	HLA-A*03:16	302	TLKSFTVEK	0,8988
A03	HLA-A*03:16	311	GIYQTSNFR	0,6304
A03	HLA-A*03:16	349	SVYAWNRKR	0,6333
A03	HLA-A*03:16	367	VLYNSASFSTFK	0,5358
A03	HLA-A*03:16	378	KCYGVSPTK	0,5631
A03	HLA-A*03:16	408	RQIAPGQTGK	0,8957
A03	HLA-A*03:16	409	QIAPGQTGK	0,6982
A03	HLA-A*03:16	454	RLFRKSNLK	0,9589
A03	HLA-A*03:16	529	KSTNLVKNK	0,5122
A03	HLA-A*03:16	724	TEILPVSMTK	0,6838
A03	HLA-A*03:16	786	KQIYKTPPIK	0,8503
A03	HLA-A*03:16	787	QIYKTPPIK	0,8934
A03	HLA-A*03:16	805	ILPDPSKPSK	0,5588
A03	HLA-A*03:16	826	VTLADAGFIK	0,5395
A03	HLA-A*03:16	827	TLADAGFIK	0,7267
A03	HLA-A*03:16	924	ANQFNSAIGK	0,5091
A03	HLA-A*03:16	939	SSTASALGK	0,5175
A03	HLA-A*03:16	1019	RASANLAATK	0,5521
A03	HLA-A*03:16	1020	ASANLAATK	0,7894
A03	HLA-A*03:16	1064	HVTYVPAQEK	0,6951
A03	HLA-A*03:16	1065	VTYVPAQEK	0,9087
A03	HLA-A*03:16	1099	GTHWFVTQR	0,5011
A03	HLA-A*03:16	1196	SLIDLQELGK	0,6419
A03	HLA-A*03:16	1264	VLKGVKLHY	0,6718
A03	HLA-A*03:17	35	GVYYPDKVFR	0,568
A03	HLA-A*03:17	41	KVFRSSVLH	0,6944
A03	HLA-A*03:17	89	GVYFASTEK	0,9487
A03	HLA-A*03:17	142	GVYYHKNNK	0,8871
A03	HLA-A*03:17	269	YLQPRTFLLK	0,5577
A03	HLA-A*03:17	302	TLKSFTVEK	0,8988
A03	HLA-A*03:17	311	GIYQTSNFR	0,6304
A03	HLA-A*03:17	349	SVYAWNRKR	0,6333
A03	HLA-A*03:17	367	VLYNSASFSTFK	0,5358
A03	HLA-A*03:17	378	KCYGVSPTK	0,5631
A03	HLA-A*03:17	408	RQIAPGQTGK	0,8957
A03	HLA-A*03:17	409	QIAPGQTGK	0,6982
A03	HLA-A*03:17	454	RLFRKSNLK	0,9589
A03	HLA-A*03:17	529	KSTNLVKNK	0,5122
A03	HLA-A*03:17	724	TEILPVSMTK	0,6838
A03	HLA-A*03:17	786	KQIYKTPPIK	0,8503
A03	HLA-A*03:17	787	QIYKTPPIK	0,8934
A03	HLA-A*03:17	805	ILPDPSKPSK	0,5588
A03	HLA-A*03:17	826	VTLADAGFIK	0,5395
A03	HLA-A*03:17	827	TLADAGFIK	0,7267

A03	HLA-A*03:17	924	ANQFNSAIGK	0,5091
A03	HLA-A*03:17	939	SSTASALGK	0,5175
A03	HLA-A*03:17	1019	RASANLAATK	0,5521
A03	HLA-A*03:17	1020	ASANLAATK	0,7894
A03	HLA-A*03:17	1064	HVTYVPAQEK	0,6951
A03	HLA-A*03:17	1065	VTYVPAQEK	0,9087
A03	HLA-A*03:17	1099	GTHWFVTQR	0,5011
A03	HLA-A*03:17	1196	SLIDLQELGK	0,6419
A03	HLA-A*03:17	1264	VLKGVKLHY	0,6718
A03	HLA-A*11:01	35	GVYYPDKVFR	0,6555
A03	HLA-A*11:01	89	GVYFASTEK	0,9386
A03	HLA-A*11:01	142	GVYYHKNNK	0,7229
A03	HLA-A*11:01	292	ALDPLSETK	0,6795
A03	HLA-A*11:01	302	TLKSFTVEK	0,819
A03	HLA-A*11:01	311	GIYQTSNFR	0,5901
A03	HLA-A*11:01	348	ASVYAWNRK	0,6551
A03	HLA-A*11:01	349	SVYAWNRKR	0,576
A03	HLA-A*11:01	369	YNSASFSTFK	0,5561
A03	HLA-A*11:01	370	NSASFSTFK	0,8025
A03	HLA-A*11:01	375	STFKCYGVSPTK	0,6638
A03	HLA-A*11:01	408	RQIAPGQTGK	0,6249
A03	HLA-A*11:01	409	QIAPGQTGK	0,6748
A03	HLA-A*11:01	454	RLFRKSNLK	0,6772
A03	HLA-A*11:01	550	GVLTESNKK	0,7312
A03	HLA-A*11:01	686	SVASQSIIAY	0,5292
A03	HLA-A*11:01	723	TTEILPVSMTK	0,5964
A03	HLA-A*11:01	724	TEILPVSMTK	0,7926
A03	HLA-A*11:01	725	EILPVSMTK	0,7733
A03	HLA-A*11:01	787	QIYKTPPIK	0,7378
A03	HLA-A*11:01	825	KVTLADAGFIK	0,5709
A03	HLA-A*11:01	826	VTLADAGFIK	0,7264
A03	HLA-A*11:01	827	TLADAGFIK	0,8305
A03	HLA-A*11:01	925	NQFNSAIGK	0,5165
A03	HLA-A*11:01	939	SSTASALGK	0,7797
A03	HLA-A*11:01	956	AQALNTLVK	0,5151
A03	HLA-A*11:01	975	SVLNDILSR	0,8499
A03	HLA-A*11:01	975	SVLNDILSRLDK	0,5938
A03	HLA-A*11:01	1019	RASANLAATK	0,5185
A03	HLA-A*11:01	1020	ASANLAATK	0,9119
A03	HLA-A*11:01	1064	HVTYVPAQEK	0,6484
A03	HLA-A*11:01	1065	VTYVPAQEK	0,9153
A03	HLA-A*11:01	1099	GTHWFVTQR	0,7768
A03	HLA-A*11:01	1196	SLIDLQELGK	0,5993
A03	HLA-A*11:02	35	GVYYPDKVFR	0,6555
A03	HLA-A*11:02	89	GVYFASTEK	0,9386
A03	HLA-A*11:02	142	GVYYHKNNK	0,7229
A03	HLA-A*11:02	292	ALDPLSETK	0,6795
A03	HLA-A*11:02	302	TLKSFTVEK	0,819
A03	HLA-A*11:02	311	GIYQTSNFR	0,5901
A03	HLA-A*11:02	348	ASVYAWNRK	0,6551

A03	HLA-A*11:02	349	SVYAWNRKR	0,576
A03	HLA-A*11:02	369	YNSASFSTFK	0,5561
A03	HLA-A*11:02	370	NSASFSTFK	0,8025
A03	HLA-A*11:02	375	STFKCYGVSPTK	0,6638
A03	HLA-A*11:02	408	RQIAPGQTGK	0,6249
A03	HLA-A*11:02	409	QIAPGQTGK	0,6748
A03	HLA-A*11:02	454	RLFRKSNLK	0,6772
A03	HLA-A*11:02	550	GVLTESNKK	0,7312
A03	HLA-A*11:02	686	SVASQSIIAY	0,5292
A03	HLA-A*11:02	723	TTEILPVSMTK	0,5964
A03	HLA-A*11:02	724	TEILPVSMTK	0,7926
A03	HLA-A*11:02	725	EILPVSMTK	0,7733
A03	HLA-A*11:02	787	QIYKTPPIK	0,7378
A03	HLA-A*11:02	825	KVTLADAGFIK	0,5709
A03	HLA-A*11:02	826	VTLADAGFIK	0,7264
A03	HLA-A*11:02	827	TLADAGFIK	0,8305
A03	HLA-A*11:02	925	NQFNSAIGK	0,5165
A03	HLA-A*11:02	939	SSTASALGK	0,7797
A03	HLA-A*11:02	956	AQALNTLVK	0,5151
A03	HLA-A*11:02	975	SVLNDILSR	0,8499
A03	HLA-A*11:02	975	SVLNDILSRLDK	0,5938
A03	HLA-A*11:02	1019	RASANLAATK	0,5185
A03	HLA-A*11:02	1020	ASANLAATK	0,9119
A03	HLA-A*11:02	1064	HVTYVPAQEK	0,6484
A03	HLA-A*11:02	1065	VTYVPAQEK	0,9153
A03	HLA-A*11:02	1099	GTHWFVTQR	0,7768
A03	HLA-A*11:02	1196	SLIDLQELGK	0,5993
A03	HLA-A*11:03	35	GVYYPDKVFR	0,5729
A03	HLA-A*11:03	41	KVFRSSVLH	0,6359
A03	HLA-A*11:03	69	HVSGTNGTK	0,5574
A03	HLA-A*11:03	89	GVYFASTEK	0,9479
A03	HLA-A*11:03	142	GVYYHKNNK	0,8636
A03	HLA-A*11:03	257	GWTAGAAAYY	0,5027
A03	HLA-A*11:03	292	ALDPLSETK	0,6109
A03	HLA-A*11:03	302	TLKSFTVEK	0,8758
A03	HLA-A*11:03	311	GIYQTSNFR	0,6106
A03	HLA-A*11:03	348	ASVYAWNRK	0,6559
A03	HLA-A*11:03	349	SVYAWNRKR	0,6192
A03	HLA-A*11:03	369	YNSASFSTFK	0,5161
A03	HLA-A*11:03	370	NSASFSTFK	0,7603
A03	HLA-A*11:03	375	STFKCYGVSPTK	0,6584
A03	HLA-A*11:03	408	RQIAPGQTGK	0,8115
A03	HLA-A*11:03	409	QIAPGQTGK	0,7924
A03	HLA-A*11:03	454	RLFRKSNLK	0,839
A03	HLA-A*11:03	529	KSTNLVKNK	0,6205
A03	HLA-A*11:03	550	GVLTESNKK	0,7419
A03	HLA-A*11:03	686	SVASQSIIAY	0,5979
A03	HLA-A*11:03	723	TTEILPVSMTK	0,5555
A03	HLA-A*11:03	724	TEILPVSMTK	0,7868
A03	HLA-A*11:03	725	EILPVSMTK	0,7674

A03	HLA-A*11:03	786	KQIYKTPPIK	0,5919
A03	HLA-A*11:03	787	QIYKTPPIK	0,8358
A03	HLA-A*11:03	825	KVTLADAGFIK	0,5148
A03	HLA-A*11:03	826	VTLADAGFIK	0,6588
A03	HLA-A*11:03	827	TLADAGFIK	0,7979
A03	HLA-A*11:03	924	ANQFNSAIGK	0,5982
A03	HLA-A*11:03	925	NQFNSAIGK	0,5795
A03	HLA-A*11:03	939	SSTASALGK	0,7993
A03	HLA-A*11:03	956	AQALNTLVK	0,5795
A03	HLA-A*11:03	975	SVLNDILSR	0,7656
A03	HLA-A*11:03	975	SVLNDILSRLDK	0,5176
A03	HLA-A*11:03	1019	RASANLAATK	0,6171
A03	HLA-A*11:03	1020	ASANLAATK	0,9033
A03	HLA-A*11:03	1064	HVTYVPAQEK	0,7064
A03	HLA-A*11:03	1065	VTYVPAQEK	0,9204
A03	HLA-A*11:03	1099	GTHWFVTQR	0,7038
A03	HLA-A*11:03	1196	SLIDLQELGK	0,6467
A03	HLA-A*11:04	35	GVYYPDKVFR	0,6723
A03	HLA-A*11:04	89	GVYFASTEK	0,9041
A03	HLA-A*11:04	142	GVYYHKNNK	0,7342
A03	HLA-A*11:04	292	ALDPLSETK	0,5968
A03	HLA-A*11:04	302	TLKSFTVEK	0,8186
A03	HLA-A*11:04	311	GIYQTSNFR	0,6771
A03	HLA-A*11:04	319	RVQPTESIVR	0,5363
A03	HLA-A*11:04	348	ASVYAWNRK	0,5541
A03	HLA-A*11:04	349	SVYAWNRKR	0,646
A03	HLA-A*11:04	370	NSASFSTFK	0,6355
A03	HLA-A*11:04	375	STFKCYGVSPTK	0,5031
A03	HLA-A*11:04	408	RQIAPGQTGK	0,7357
A03	HLA-A*11:04	409	QIAPGQTGK	0,6296
A03	HLA-A*11:04	454	RLFRKSNLK	0,8162
A03	HLA-A*11:04	458	KSNLKPFER	0,6282
A03	HLA-A*11:04	529	KSTNLVKNK	0,5664
A03	HLA-A*11:04	550	GVLTESNKK	0,6125
A03	HLA-A*11:04	724	TEILPVSMTK	0,653
A03	HLA-A*11:04	725	EILPVSMTK	0,6293
A03	HLA-A*11:04	786	KQIYKTPPIK	0,5121
A03	HLA-A*11:04	787	QIYKTPPIK	0,7537
A03	HLA-A*11:04	825	KVTLADAGFIK	0,5571
A03	HLA-A*11:04	826	VTLADAGFIK	0,5926
A03	HLA-A*11:04	827	TLADAGFIK	0,7565
A03	HLA-A*11:04	939	SSTASALGK	0,6578
A03	HLA-A*11:04	956	AQALNTLVK	0,5269
A03	HLA-A*11:04	975	SVLNDILSR	0,7843
A03	HLA-A*11:04	1019	RASANLAATK	0,5722
A03	HLA-A*11:04	1020	ASANLAATK	0,8712
A03	HLA-A*11:04	1064	HVTYVPAQEK	0,5792
A03	HLA-A*11:04	1065	VTYVPAQEK	0,8811
A03	HLA-A*11:04	1099	GTHWFVTQR	0,7363
A03	HLA-A*11:05	35	GVYYPDKVFR	0,6555

A03	HLA-A*11:05	89	GVYFASTEK	0,9386
A03	HLA-A*11:05	142	GVYYHKNNK	0,7229
A03	HLA-A*11:05	292	ALDPLSETK	0,6795
A03	HLA-A*11:05	302	TLKSFTVEK	0,819
A03	HLA-A*11:05	311	GIYQTSNFR	0,5901
A03	HLA-A*11:05	348	ASVYAWNRK	0,6551
A03	HLA-A*11:05	349	SVYAWNRKR	0,576
A03	HLA-A*11:05	369	YNSASFSTFK	0,5561
A03	HLA-A*11:05	370	NSASFSTFK	0,8025
A03	HLA-A*11:05	375	STFKCYGVSPTK	0,6638
A03	HLA-A*11:05	408	RQIAPGQTGK	0,6249
A03	HLA-A*11:05	409	QIAPGQTGK	0,6748
A03	HLA-A*11:05	454	RLFRKSNLK	0,6772
A03	HLA-A*11:05	550	GVLTESNKK	0,7312
A03	HLA-A*11:05	686	SVASQSIIAY	0,5292
A03	HLA-A*11:05	723	TTEILPVSMTK	0,5964
A03	HLA-A*11:05	724	TEILPVSMTK	0,7926
A03	HLA-A*11:05	725	EILPVSMTK	0,7733
A03	HLA-A*11:05	787	QIYKTPPIK	0,7378
A03	HLA-A*11:05	825	KVTLADAGFIK	0,5709
A03	HLA-A*11:05	826	VTLADAGFIK	0,7264
A03	HLA-A*11:05	827	TLADAGFIK	0,8305
A03	HLA-A*11:05	925	NQFNSAIGK	0,5165
A03	HLA-A*11:05	939	SSTASALGK	0,7797
A03	HLA-A*11:05	956	AQALNTLVK	0,5151
A03	HLA-A*11:05	975	SVLNDILSR	0,8499
A03	HLA-A*11:05	975	SVLNDILSRLDK	0,5938
A03	HLA-A*11:05	1019	RASANLAATK	0,5185
A03	HLA-A*11:05	1020	ASANLAATK	0,9119
A03	HLA-A*11:05	1064	HVTYVPAQEK	0,6484
A03	HLA-A*11:05	1065	VTYVPAQEK	0,9153
A03	HLA-A*11:05	1099	GTHWFVTQR	0,7768
A03	HLA-A*11:05	1196	SLIDLQELGK	0,5993
A03	HLA-A*11:07	35	GVYYPDKVFR	0,6555
A03	HLA-A*11:07	89	GVYFASTEK	0,9386
A03	HLA-A*11:07	142	GVYYHKNNK	0,7229
A03	HLA-A*11:07	292	ALDPLSETK	0,6795
A03	HLA-A*11:07	302	TLKSFTVEK	0,819
A03	HLA-A*11:07	311	GIYQTSNFR	0,5901
A03	HLA-A*11:07	348	ASVYAWNRK	0,6551
A03	HLA-A*11:07	349	SVYAWNRKR	0,576
A03	HLA-A*11:07	369	YNSASFSTFK	0,5561
A03	HLA-A*11:07	370	NSASFSTFK	0,8025
A03	HLA-A*11:07	375	STFKCYGVSPTK	0,6638
A03	HLA-A*11:07	408	RQIAPGQTGK	0,6249
A03	HLA-A*11:07	409	QIAPGQTGK	0,6748
A03	HLA-A*11:07	454	RLFRKSNLK	0,6772
A03	HLA-A*11:07	550	GVLTESNKK	0,7312
A03	HLA-A*11:07	686	SVASQSIIAY	0,5292
A03	HLA-A*11:07	723	TTEILPVSMTK	0,5964

A03	HLA-A*11:07	724	TEILPVSMTK	0,7926
A03	HLA-A*11:07	725	EILPVSMTK	0,7733
A03	HLA-A*11:07	787	QIYKTPPIK	0,7378
A03	HLA-A*11:07	825	KVTLADAGFIK	0,5709
A03	HLA-A*11:07	826	VTLADAGFIK	0,7264
A03	HLA-A*11:07	827	TLADAGFIK	0,8305
A03	HLA-A*11:07	925	NQFNSAIGK	0,5165
A03	HLA-A*11:07	939	SSTASALGK	0,7797
A03	HLA-A*11:07	956	AQALNTLVK	0,5151
A03	HLA-A*11:07	975	SVLNDILSR	0,8499
A03	HLA-A*11:07	975	SVLNDILSRLDK	0,5938
A03	HLA-A*11:07	1019	RASANLAATK	0,5185
A03	HLA-A*11:07	1020	ASANLAATK	0,9119
A03	HLA-A*11:07	1064	HVTYVPAQEK	0,6484
A03	HLA-A*11:07	1065	VTYVPAQEK	0,9153
A03	HLA-A*11:07	1099	GTHWFVTQR	0,7768
A03	HLA-A*11:07	1196	SLIDLQELGK	0,5993
A03	HLA-A*11:08	30	NSFTRGVYY	0,6147
A03	HLA-A*11:08	41	KVFRSSVLH	0,5716
A03	HLA-A*11:08	69	HVSGTNGTK	0,5285
A03	HLA-A*11:08	89	GVYFASTEK	0,9181
A03	HLA-A*11:08	142	GVYYHKNNK	0,7168
A03	HLA-A*11:08	257	GWTAGAAAYY	0,6585
A03	HLA-A*11:08	258	WTAGAAAYY	0,5874
A03	HLA-A*11:08	261	GAAAYYVGY	0,5405
A03	HLA-A*11:08	292	ALDPLSETK	0,7101
A03	HLA-A*11:08	302	TLKSFTVEK	0,8239
A03	HLA-A*11:08	311	GIYQTSNFR	0,5198
A03	HLA-A*11:08	348	ASVYAWNRK	0,6142
A03	HLA-A*11:08	349	SVYAWNRKR	0,5031
A03	HLA-A*11:08	361	CVADYSVLY	0,5983
A03	HLA-A*11:08	369	YNSASFSTFK	0,5604
A03	HLA-A*11:08	370	NSASFSTFK	0,8068
A03	HLA-A*11:08	375	STFKCYGVSPTK	0,5765
A03	HLA-A*11:08	408	RQIAPGQTGK	0,7216
A03	HLA-A*11:08	409	QIAPGQTGK	0,7553
A03	HLA-A*11:08	454	RLFRKSNLK	0,707
A03	HLA-A*11:08	550	GVLTESNKK	0,7019
A03	HLA-A*11:08	604	TSNQVAVLY	0,6574
A03	HLA-A*11:08	686	SVASQSIIAY	0,7162
A03	HLA-A*11:08	723	TTEILPVSMTK	0,519
A03	HLA-A*11:08	724	TEILPVSMTK	0,7064
A03	HLA-A*11:08	725	EILPVSMTK	0,7265
A03	HLA-A*11:08	787	QIYKTPPIK	0,7324
A03	HLA-A*11:08	805	ILPDPSKPSK	0,536
A03	HLA-A*11:08	825	KVTLADAGFIK	0,5029
A03	HLA-A*11:08	826	VTLADAGFIK	0,6667
A03	HLA-A*11:08	827	TLADAGFIK	0,8043
A03	HLA-A*11:08	925	NQFNSAIGK	0,5146
A03	HLA-A*11:08	939	SSTASALGK	0,7826

A03	HLA-A*11:08	956	AQALNTLVK	0,5229
A03	HLA-A*11:08	975	SVLNDILSR	0,7316
A03	HLA-A*11:08	1019	RASANLAATK	0,5652
A03	HLA-A*11:08	1020	ASANLAATK	0,8837
A03	HLA-A*11:08	1064	HVTYVPAQEK	0,6371
A03	HLA-A*11:08	1065	VTYVPAQEK	0,8948
A03	HLA-A*11:08	1099	GTHWFVTQR	0,6403
A03	HLA-A*11:08	1196	SLIDLQELGK	0,5719
A03	HLA-A*11:09	35	GVYYPDKVFR	0,6555
A03	HLA-A*11:09	89	GVYFASTEK	0,9386
A03	HLA-A*11:09	142	GVYYHKNNK	0,7229
A03	HLA-A*11:09	292	ALDPLSETK	0,6795
A03	HLA-A*11:09	302	TLKSFTVEK	0,819
A03	HLA-A*11:09	311	GIYQTSNFR	0,5901
A03	HLA-A*11:09	348	ASVYAWNRK	0,6551
A03	HLA-A*11:09	349	SVYAWNRKR	0,576
A03	HLA-A*11:09	369	YNSASFSTFK	0,5561
A03	HLA-A*11:09	370	NSASFSTFK	0,8025
A03	HLA-A*11:09	375	STFKCYGVSPTK	0,6638
A03	HLA-A*11:09	408	RQIAPGQTGK	0,6249
A03	HLA-A*11:09	409	QIAPGQTGK	0,6748
A03	HLA-A*11:09	454	RLFRKSNLK	0,6772
A03	HLA-A*11:09	550	GVLTESNKK	0,7312
A03	HLA-A*11:09	686	SVASQSIIAY	0,5292
A03	HLA-A*11:09	723	TTEILPVSMTK	0,5964
A03	HLA-A*11:09	724	TEILPVSMTK	0,7926
A03	HLA-A*11:09	725	EILPVSMTK	0,7733
A03	HLA-A*11:09	787	QIYKTPPIK	0,7378
A03	HLA-A*11:09	825	KVTLADAGFIK	0,5709
A03	HLA-A*11:09	826	VTLADAGFIK	0,7264
A03	HLA-A*11:09	827	TLADAGFIK	0,8305
A03	HLA-A*11:09	925	NQFNSAIGK	0,5165
A03	HLA-A*11:09	939	SSTASALGK	0,7797
A03	HLA-A*11:09	956	AQALNTLVK	0,5151
A03	HLA-A*11:09	975	SVLNDILSR	0,8499
A03	HLA-A*11:09	975	SVLNDILSRLDK	0,5938
A03	HLA-A*11:09	1019	RASANLAATK	0,5185
A03	HLA-A*11:09	1020	ASANLAATK	0,9119
A03	HLA-A*11:09	1064	HVTYVPAQEK	0,6484
A03	HLA-A*11:09	1065	VTYVPAQEK	0,9153
A03	HLA-A*11:09	1099	GTHWFVTQR	0,7768
A03	HLA-A*11:09	1196	SLIDLQELGK	0,5993
A03	HLA-A*11:10	30	NSFTRGVYY	0,5648
A03	HLA-A*11:10	88	DGVYFASTEK	0,5092
A03	HLA-A*11:10	89	GVYFASTEK	0,6152
A03	HLA-A*11:10	258	WTAGAAAYY	0,5276
A03	HLA-A*11:10	361	CVADYSVLY	0,5044
A03	HLA-A*11:10	370	NSASFSTFK	0,7786
A03	HLA-A*11:10	724	TEILPVSMTK	0,5103
A03	HLA-A*11:10	725	EILPVSMTK	0,8498

A03	HLA-A*11:10	817	FIEDLLFNK	0,5044
A03	HLA-A*11:10	827	TLADAGFIK	0,6366
A03	HLA-A*11:10	975	SVLNDILSR	0,6653
A03	HLA-A*11:10	1065	VTYVPAQEK	0,5627
A03	HLA-A*11:10	1173	NASVVNIQK	0,5907
A03	HLA-A*11:12	35	GVYYPDKVFR	0,6555
A03	HLA-A*11:12	89	GVYFASTEK	0,9386
A03	HLA-A*11:12	142	GVYYHKNNK	0,7229
A03	HLA-A*11:12	292	ALDPLSETK	0,6795
A03	HLA-A*11:12	302	TLKSFTVEK	0,819
A03	HLA-A*11:12	311	GIYQTSNFR	0,5901
A03	HLA-A*11:12	348	ASVYAWNRK	0,6551
A03	HLA-A*11:12	349	SVYAWNRKR	0,576
A03	HLA-A*11:12	369	YNSASFSTFK	0,5561
A03	HLA-A*11:12	370	NSASFSTFK	0,8025
A03	HLA-A*11:12	375	STFKCYGVSPTK	0,6638
A03	HLA-A*11:12	408	RQIAPGQTGK	0,6249
A03	HLA-A*11:12	409	QIAPGQTGK	0,6748
A03	HLA-A*11:12	454	RLFRKSNLK	0,6772
A03	HLA-A*11:12	550	GVLTESNKK	0,7312
A03	HLA-A*11:12	686	SVASQSIIAY	0,5292
A03	HLA-A*11:12	723	TTEILPVSMTK	0,5964
A03	HLA-A*11:12	724	TEILPVSMTK	0,7926
A03	HLA-A*11:12	725	EILPVSMTK	0,7733
A03	HLA-A*11:12	787	QIYKTPPIK	0,7378
A03	HLA-A*11:12	825	KVTLADAGFIK	0,5709
A03	HLA-A*11:12	826	VTLADAGFIK	0,7264
A03	HLA-A*11:12	827	TLADAGFIK	0,8305
A03	HLA-A*11:12	925	NQFNSAIGK	0,5165
A03	HLA-A*11:12	939	SSTASALGK	0,7797
A03	HLA-A*11:12	956	AQALNTLVK	0,5151
A03	HLA-A*11:12	975	SVLNDILSR	0,8499
A03	HLA-A*11:12	975	SVLNDILSRLDK	0,5938
A03	HLA-A*11:12	1019	RASANLAATK	0,5185
A03	HLA-A*11:12	1020	ASANLAATK	0,9119
A03	HLA-A*11:12	1064	HVTYVPAQEK	0,6484
A03	HLA-A*11:12	1065	VTYVPAQEK	0,9153
A03	HLA-A*11:12	1099	GTHWFVTQR	0,7768
A03	HLA-A*11:12	1196	SLIDLQELGK	0,5993
A03	HLA-A*11:13	35	GVYYPDKVFR	0,6555
A03	HLA-A*11:13	89	GVYFASTEK	0,9386
A03	HLA-A*11:13	142	GVYYHKNNK	0,7229
A03	HLA-A*11:13	292	ALDPLSETK	0,6795
A03	HLA-A*11:13	302	TLKSFTVEK	0,819
A03	HLA-A*11:13	311	GIYQTSNFR	0,5901
A03	HLA-A*11:13	348	ASVYAWNRK	0,6551
A03	HLA-A*11:13	349	SVYAWNRKR	0,576
A03	HLA-A*11:13	369	YNSASFSTFK	0,5561
A03	HLA-A*11:13	370	NSASFSTFK	0,8025
A03	HLA-A*11:13	375	STFKCYGVSPTK	0,6638

A03	HLA-A*11:13	408	RQIAPGQTGK	0,6249
A03	HLA-A*11:13	409	QIAPGQTGK	0,6748
A03	HLA-A*11:13	454	RLFRKSNLK	0,6772
A03	HLA-A*11:13	550	GVLTESNKK	0,7312
A03	HLA-A*11:13	686	SVASQSIIAY	0,5292
A03	HLA-A*11:13	723	TTEILPVSMTK	0,5964
A03	HLA-A*11:13	724	TEILPVSMTK	0,7926
A03	HLA-A*11:13	725	EILPVSMTK	0,7733
A03	HLA-A*11:13	787	QIYKTPPIK	0,7378
A03	HLA-A*11:13	825	KVTLADAGFIK	0,5709
A03	HLA-A*11:13	826	VTLADAGFIK	0,7264
A03	HLA-A*11:13	827	TLADAGFIK	0,8305
A03	HLA-A*11:13	925	NQFNSAIGK	0,5165
A03	HLA-A*11:13	939	SSTASALGK	0,7797
A03	HLA-A*11:13	956	AQALNTLVK	0,5151
A03	HLA-A*11:13	975	SVLNDILSR	0,8499
A03	HLA-A*11:13	975	SVLNDILSRLDK	0,5938
A03	HLA-A*11:13	1019	RASANLAATK	0,5185
A03	HLA-A*11:13	1020	ASANLAATK	0,9119
A03	HLA-A*11:13	1064	HVTYVPAQEK	0,6484
A03	HLA-A*11:13	1065	VTYVPAQEK	0,9153
A03	HLA-A*11:13	1099	GTHWFVTQR	0,7768
A03	HLA-A*11:13	1196	SLIDLQELGK	0,5993
A03	HLA-A*11:14	41	KVFRSSVLH	0,5801
A03	HLA-A*11:14	89	GVYFASTEK	0,9113
A03	HLA-A*11:14	142	GVYYHKNNK	0,7242
A03	HLA-A*11:14	292	ALDPLSETK	0,552
A03	HLA-A*11:14	302	TLKSFTVEK	0,7933
A03	HLA-A*11:14	311	GIYQTSNFR	0,5287
A03	HLA-A*11:14	348	ASVYAWNRK	0,5921
A03	HLA-A*11:14	349	SVYAWNRKR	0,524
A03	HLA-A*11:14	370	NSASFSTFK	0,7046
A03	HLA-A*11:14	375	STFKCYGVSPTK	0,5354
A03	HLA-A*11:14	408	RQIAPGQTGK	0,6772
A03	HLA-A*11:14	409	QIAPGQTGK	0,6664
A03	HLA-A*11:14	454	RLFRKSNLK	0,7323
A03	HLA-A*11:14	550	GVLTESNKK	0,6399
A03	HLA-A*11:14	686	SVASQSIIAY	0,5437
A03	HLA-A*11:14	724	TEILPVSMTK	0,6944
A03	HLA-A*11:14	725	EILPVSMTK	0,6759
A03	HLA-A*11:14	787	QIYKTPPIK	0,728
A03	HLA-A*11:14	826	VTLADAGFIK	0,595
A03	HLA-A*11:14	827	TLADAGFIK	0,7304
A03	HLA-A*11:14	924	ANQFNSAIGK	0,5078
A03	HLA-A*11:14	925	NQFNSAIGK	0,519
A03	HLA-A*11:14	939	SSTASALGK	0,7137
A03	HLA-A*11:14	956	AQALNTLVK	0,5035
A03	HLA-A*11:14	975	SVLNDILSR	0,743
A03	HLA-A*11:14	1019	RASANLAATK	0,5006
A03	HLA-A*11:14	1020	ASANLAATK	0,857

A03	HLA-A*11:14	1064	HVTYVPAQEK	0,5851
A03	HLA-A*11:14	1065	VTYVPAQEK	0,8766
A03	HLA-A*11:14	1099	GTHWFVTQR	0,6316
A03	HLA-A*11:14	1196	SLIDLQELGK	0,536
A03	HLA-A*11:15	35	GVYYPDKVFR	0,6555
A03	HLA-A*11:15	89	GVYFASTEK	0,9386
A03	HLA-A*11:15	142	GVYYHKNNK	0,7229
A03	HLA-A*11:15	292	ALDPLSETK	0,6795
A03	HLA-A*11:15	302	TLKSFTVEK	0,819
A03	HLA-A*11:15	311	GIYQTSNFR	0,5901
A03	HLA-A*11:15	348	ASVYAWNRK	0,6551
A03	HLA-A*11:15	349	SVYAWNRKR	0,576
A03	HLA-A*11:15	369	YNSASFSTFK	0,5561
A03	HLA-A*11:15	370	NSASFSTFK	0,8025
A03	HLA-A*11:15	375	STFKCYGVSPTK	0,6638
A03	HLA-A*11:15	408	RQIAPGQTGK	0,6249
A03	HLA-A*11:15	409	QIAPGQTGK	0,6748
A03	HLA-A*11:15	454	RLFRKSNLK	0,6772
A03	HLA-A*11:15	550	GVLTESNKK	0,7312
A03	HLA-A*11:15	686	SVASQSIIAY	0,5292
A03	HLA-A*11:15	723	TTEILPVSMTK	0,5964
A03	HLA-A*11:15	724	TEILPVSMTK	0,7926
A03	HLA-A*11:15	725	EILPVSMTK	0,7733
A03	HLA-A*11:15	787	QIYKTPPIK	0,7378
A03	HLA-A*11:15	825	KVTLADAGFIK	0,5709
A03	HLA-A*11:15	826	VTLADAGFIK	0,7264
A03	HLA-A*11:15	827	TLADAGFIK	0,8305
A03	HLA-A*11:15	925	NQFNSAIGK	0,5165
A03	HLA-A*11:15	939	SSTASALGK	0,7797
A03	HLA-A*11:15	956	AQALNTLVK	0,5151
A03	HLA-A*11:15	975	SVLNDILSR	0,8499
A03	HLA-A*11:15	975	SVLNDILSRLDK	0,5938
A03	HLA-A*11:15	1019	RASANLAATK	0,5185
A03	HLA-A*11:15	1020	ASANLAATK	0,9119
A03	HLA-A*11:15	1064	HVTYVPAQEK	0,6484
A03	HLA-A*11:15	1065	VTYVPAQEK	0,9153
A03	HLA-A*11:15	1099	GTHWFVTQR	0,7768
A03	HLA-A*11:15	1196	SLIDLQELGK	0,5993
A03	HLA-A*11:16	35	GVYYPDKVFR	0,6555
A03	HLA-A*11:16	89	GVYFASTEK	0,9386
A03	HLA-A*11:16	142	GVYYHKNNK	0,7229
A03	HLA-A*11:16	292	ALDPLSETK	0,6795
A03	HLA-A*11:16	302	TLKSFTVEK	0,819
A03	HLA-A*11:16	311	GIYQTSNFR	0,5901
A03	HLA-A*11:16	348	ASVYAWNRK	0,6551
A03	HLA-A*11:16	349	SVYAWNRKR	0,576
A03	HLA-A*11:16	369	YNSASFSTFK	0,5561
A03	HLA-A*11:16	370	NSASFSTFK	0,8025
A03	HLA-A*11:16	375	STFKCYGVSPTK	0,6638
A03	HLA-A*11:16	408	RQIAPGQTGK	0,6249

A03	HLA-A*11:16	409	QIAPGQTGK	0,6748
A03	HLA-A*11:16	454	RLFRKSNLK	0,6772
A03	HLA-A*11:16	550	GVLTESNKK	0,7312
A03	HLA-A*11:16	686	SVASQSIIAY	0,5292
A03	HLA-A*11:16	723	TTEILPVSMTK	0,5964
A03	HLA-A*11:16	724	TEILPVSMTK	0,7926
A03	HLA-A*11:16	725	EILPVSMTK	0,7733
A03	HLA-A*11:16	787	QIYKTPPIK	0,7378
A03	HLA-A*11:16	825	KVTLADAGFIK	0,5709
A03	HLA-A*11:16	826	VTLADAGFIK	0,7264
A03	HLA-A*11:16	827	TLADAGFIK	0,8305
A03	HLA-A*11:16	925	NQFNSAIGK	0,5165
A03	HLA-A*11:16	939	SSTASALGK	0,7797
A03	HLA-A*11:16	956	AQALNTLVK	0,5151
A03	HLA-A*11:16	975	SVLNDILSR	0,8499
A03	HLA-A*11:16	975	SVLNDILSRLDK	0,5938
A03	HLA-A*11:16	1019	RASANLAATK	0,5185
A03	HLA-A*11:16	1020	ASANLAATK	0,9119
A03	HLA-A*11:16	1064	HVTYVPAQEK	0,6484
A03	HLA-A*11:16	1065	VTYVPAQEK	0,9153
A03	HLA-A*11:16	1099	GTHWFVTQR	0,7768
A03	HLA-A*11:16	1196	SLIDLQELGK	0,5993
A03	HLA-A*11:20	35	GVYYPDKVFR	0,5729
A03	HLA-A*11:20	41	KVFRSSVLH	0,6359
A03	HLA-A*11:20	69	HVSGTNGTK	0,5574
A03	HLA-A*11:20	89	GVYFASTEK	0,9479
A03	HLA-A*11:20	142	GVYYHKNNK	0,8636
A03	HLA-A*11:20	257	GWTAGAAAYY	0,5027
A03	HLA-A*11:20	292	ALDPLSETK	0,6109
A03	HLA-A*11:20	302	TLKSFTVEK	0,8758
A03	HLA-A*11:20	311	GIYQTSNFR	0,6106
A03	HLA-A*11:20	348	ASVYAWNRK	0,6559
A03	HLA-A*11:20	349	SVYAWNRKR	0,6192
A03	HLA-A*11:20	369	YNSASFSTFK	0,5161
A03	HLA-A*11:20	370	NSASFSTFK	0,7603
A03	HLA-A*11:20	375	STFKCYGVSPTK	0,6584
A03	HLA-A*11:20	408	RQIAPGQTGK	0,8115
A03	HLA-A*11:20	409	QIAPGQTGK	0,7924
A03	HLA-A*11:20	454	RLFRKSNLK	0,839
A03	HLA-A*11:20	529	KSTNLVKNK	0,6205
A03	HLA-A*11:20	550	GVLTESNKK	0,7419
A03	HLA-A*11:20	686	SVASQSIIAY	0,5979
A03	HLA-A*11:20	723	TTEILPVSMTK	0,5555
A03	HLA-A*11:20	724	TEILPVSMTK	0,7868
A03	HLA-A*11:20	725	EILPVSMTK	0,7674
A03	HLA-A*11:20	786	KQIYKTPPIK	0,5919
A03	HLA-A*11:20	787	QIYKTPPIK	0,8358
A03	HLA-A*11:20	825	KVTLADAGFIK	0,5148
A03	HLA-A*11:20	826	VTLADAGFIK	0,6588
A03	HLA-A*11:20	827	TLADAGFIK	0,7979

A03	HLA-A*11:20	924	ANQFNSAIGK	0,5982
A03	HLA-A*11:20	925	NQFNSAIGK	0,5795
A03	HLA-A*11:20	939	SSTASALGK	0,7993
A03	HLA-A*11:20	956	AQALNTLVK	0,5795
A03	HLA-A*11:20	975	SVLNDILSR	0,7656
A03	HLA-A*11:20	975	SVLNDILSRLDK	0,5176
A03	HLA-A*11:20	1019	RASANLAATK	0,6171
A03	HLA-A*11:20	1020	ASANLAATK	0,9033
A03	HLA-A*11:20	1064	HVTYVPAQEK	0,7064
A03	HLA-A*11:20	1065	VTYVPAQEK	0,9204
A03	HLA-A*11:20	1099	GTHWFVTQR	0,7038
A03	HLA-A*11:20	1196	SLIDLQELGK	0,6467
A03	HLA-A*11:23	35	GVYYPDKVFR	0,6555
A03	HLA-A*11:23	89	GVYFASTEK	0,9386
A03	HLA-A*11:23	142	GVYYHKNNK	0,7229
A03	HLA-A*11:23	292	ALDPLSETK	0,6795
A03	HLA-A*11:23	302	TLKSFTVEK	0,819
A03	HLA-A*11:23	311	GIYQTSNFR	0,5901
A03	HLA-A*11:23	348	ASVYAWNRK	0,6551
A03	HLA-A*11:23	349	SVYAWNRKR	0,576
A03	HLA-A*11:23	369	YNSASFSTFK	0,5561
A03	HLA-A*11:23	370	NSASFSTFK	0,8025
A03	HLA-A*11:23	375	STFKCYGVSPTK	0,6638
A03	HLA-A*11:23	408	RQIAPGQTGK	0,6249
A03	HLA-A*11:23	409	QIAPGQTGK	0,6748
A03	HLA-A*11:23	454	RLFRKSNLK	0,6772
A03	HLA-A*11:23	550	GVLTESNKK	0,7312
A03	HLA-A*11:23	686	SVASQSIIAY	0,5292
A03	HLA-A*11:23	723	TTEILPVSMTK	0,5964
A03	HLA-A*11:23	724	TEILPVSMTK	0,7926
A03	HLA-A*11:23	725	EILPVSMTK	0,7733
A03	HLA-A*11:23	787	QIYKTPPIK	0,7378
A03	HLA-A*11:23	825	KVTLADAGFIK	0,5709
A03	HLA-A*11:23	826	VTLADAGFIK	0,7264
A03	HLA-A*11:23	827	TLADAGFIK	0,8305
A03	HLA-A*11:23	925	NQFNSAIGK	0,5165
A03	HLA-A*11:23	939	SSTASALGK	0,7797
A03	HLA-A*11:23	956	AQALNTLVK	0,5151
A03	HLA-A*11:23	975	SVLNDILSR	0,8499
A03	HLA-A*11:23	975	SVLNDILSRLDK	0,5938
A03	HLA-A*11:23	1019	RASANLAATK	0,5185
A03	HLA-A*11:23	1020	ASANLAATK	0,9119
A03	HLA-A*11:23	1064	HVTYVPAQEK	0,6484
A03	HLA-A*11:23	1065	VTYVPAQEK	0,9153
A03	HLA-A*11:23	1099	GTHWFVTQR	0,7768
A03	HLA-A*11:23	1196	SLIDLQELGK	0,5993
A03	HLA-A*31:01	34	RGVYYPDKVFR	0,5574
A03	HLA-A*31:01	35	GVYYPDKVFR	0,7294
A03	HLA-A*31:01	36	VYYPDKVFR	0,8085
A03	HLA-A*31:01	150	KSWMESEFR	0,5583

A03	HLA-A*31:01	182	KQGNFKNLR	0,6717
A03	HLA-A*31:01	310	KGIYQTSNFR	0,5075
A03	HLA-A*31:01	311	GIYQTSNFR	0,6612
A03	HLA-A*31:01	319	RVQPTESIVR	0,5738
A03	HLA-A*31:01	346	RFASVYAWNR	0,7025
A03	HLA-A*31:01	349	SVYAWNRKR	0,8646
A03	HLA-A*31:01	395	VYADSFVIR	0,575
A03	HLA-A*31:01	446	GGNYNYLYR	0,5086
A03	HLA-A*31:01	454	RLFRKSNLK	0,5237
A03	HLA-A*31:01	458	KSNLKPFER	0,8687
A03	HLA-A*31:01	558	KFLPFQQFGR	0,6235
A03	HLA-A*31:01	677	QTNSPRRAR	0,6557
A03	HLA-A*31:01	975	SVLNDILSR	0,6352
A03	HLA-A*31:01	1099	GTHWFVTQR	0,837
A03	HLA-A*31:03	34	RGVYYPDKVFR	0,6638
A03	HLA-A*31:03	35	GVYYPDKVFR	0,8323
A03	HLA-A*31:03	36	VYYPDKVFR	0,882
A03	HLA-A*31:03	142	GVYYHKNNK	0,5143
A03	HLA-A*31:03	150	KSWMESEFR	0,7064
A03	HLA-A*31:03	182	KQGNFKNLR	0,768
A03	HLA-A*31:03	193	VFKNIDGYFK	0,6092
A03	HLA-A*31:03	237	RFQTLLALHR	0,5604
A03	HLA-A*31:03	264	AYYVGYLQPR	0,5113
A03	HLA-A*31:03	302	TLKSFTVEK	0,6023
A03	HLA-A*31:03	310	KGIYQTSNFR	0,6
A03	HLA-A*31:03	311	GIYQTSNFR	0,7783
A03	HLA-A*31:03	319	RVQPTESIVR	0,6913
A03	HLA-A*31:03	346	RFASVYAWNR	0,73
A03	HLA-A*31:03	348	ASVYAWNRKR	0,5374
A03	HLA-A*31:03	349	SVYAWNRKR	0,8673
A03	HLA-A*31:03	394	NVYADSFVIR	0,5006
A03	HLA-A*31:03	395	VYADSFVIR	0,708
A03	HLA-A*31:03	446	GGNYNYLYR	0,7092
A03	HLA-A*31:03	449	YNYLYRLFR	0,5488
A03	HLA-A*31:03	454	RLFRKSNLK	0,6239
A03	HLA-A*31:03	457	RKSNLKPFER	0,5092
A03	HLA-A*31:03	458	KSNLKPFER	0,9139
A03	HLA-A*31:03	558	KFLPFQQFGR	0,7268
A03	HLA-A*31:03	637	STGSNVFQTR	0,6297
A03	HLA-A*31:03	638	TGSNVFQTR	0,5408
A03	HLA-A*31:03	677	QTNSPRRAR	0,6697
A03	HLA-A*31:03	757	GSFCTQLNR	0,5163
A03	HLA-A*31:03	975	SVLNDILSR	0,8099
A03	HLA-A*31:03	991	VQIDRLITGR	0,5083
A03	HLA-A*31:03	1099	GTHWFVTQR	0,8669
A03	HLA-A*31:04	34	RGVYYPDKVFR	0,6638
A03	HLA-A*31:04	35	GVYYPDKVFR	0,8323
A03	HLA-A*31:04	36	VYYPDKVFR	0,882
A03	HLA-A*31:04	142	GVYYHKNNK	0,5143
A03	HLA-A*31:04	150	KSWMESEFR	0,7064

A03	HLA-A*31:04	182	KQGNFKNLR	0,768
A03	HLA-A*31:04	193	VFKNIDGYFK	0,6092
A03	HLA-A*31:04	237	RFQTLLALHR	0,5604
A03	HLA-A*31:04	264	AYYVGYLQPR	0,5113
A03	HLA-A*31:04	302	TLKSFTVEK	0,6023
A03	HLA-A*31:04	310	KGIYQTSNFR	0,6
A03	HLA-A*31:04	311	GIYQTSNFR	0,7783
A03	HLA-A*31:04	319	RVQPTESIVR	0,6913
A03	HLA-A*31:04	346	RFASVYAWNR	0,73
A03	HLA-A*31:04	348	ASVYAWNRKR	0,5374
A03	HLA-A*31:04	349	SVYAWNRKR	0,8673
A03	HLA-A*31:04	394	NVYADSFVIR	0,5006
A03	HLA-A*31:04	395	VYADSFVIR	0,708
A03	HLA-A*31:04	446	GGNYNYLYR	0,7092
A03	HLA-A*31:04	449	YNYLYRLFR	0,5488
A03	HLA-A*31:04	454	RLFRKSNLK	0,6239
A03	HLA-A*31:04	457	RKSNLKPFER	0,5092
A03	HLA-A*31:04	458	KSNLKPFER	0,9139
A03	HLA-A*31:04	558	KFLPFQQFGR	0,7268
A03	HLA-A*31:04	637	STGSNVFQTR	0,6297
A03	HLA-A*31:04	638	TGSNVFQTR	0,5408
A03	HLA-A*31:04	677	QTNSPRRAR	0,6697
A03	HLA-A*31:04	757	GSFCTQLNR	0,5163
A03	HLA-A*31:04	975	SVLNDILSR	0,8099
A03	HLA-A*31:04	991	VQIDRLITGR	0,5083
A03	HLA-A*31:04	1099	GTHWFVTQR	0,8669
A03	HLA-A*31:05	35	GVYYPDKVFR	0,5445
A03	HLA-A*31:05	36	VYYPDKVFR	0,7165
A03	HLA-A*31:05	346	RFASVYAWNR	0,6108
A03	HLA-A*31:05	349	SVYAWNRKR	0,6829
A03	HLA-A*31:05	395	VYADSFVIR	0,5499
A03	HLA-A*31:05	458	KSNLKPFER	0,6747
A03	HLA-A*31:05	1099	GTHWFVTQR	0,6122
A03	HLA-A*31:06	34	RGVYYPDKVFR	0,5993
A03	HLA-A*31:06	35	GVYYPDKVFR	0,8058
A03	HLA-A*31:06	36	VYYPDKVFR	0,8626
A03	HLA-A*31:06	150	KSWMESEFR	0,6719
A03	HLA-A*31:06	182	KQGNFKNLR	0,7159
A03	HLA-A*31:06	193	VFKNIDGYFK	0,5689
A03	HLA-A*31:06	237	RFQTLLALHR	0,5435
A03	HLA-A*31:06	302	TLKSFTVEK	0,5191
A03	HLA-A*31:06	310	KGIYQTSNFR	0,5598
A03	HLA-A*31:06	311	GIYQTSNFR	0,7562
A03	HLA-A*31:06	319	RVQPTESIVR	0,6572
A03	HLA-A*31:06	346	RFASVYAWNR	0,7268
A03	HLA-A*31:06	349	SVYAWNRKR	0,8534
A03	HLA-A*31:06	395	VYADSFVIR	0,6684
A03	HLA-A*31:06	446	GGNYNYLYR	0,647
A03	HLA-A*31:06	449	YNYLYRLFR	0,5126
A03	HLA-A*31:06	454	RLFRKSNLK	0,5816

A03	HLA-A*31:06	458	KSNLKPFER	0,8983
A03	HLA-A*31:06	558	KFLPFQQFGR	0,6977
A03	HLA-A*31:06	637	STGSNVFQTR	0,5673
A03	HLA-A*31:06	677	QTNSPRRAR	0,5945
A03	HLA-A*31:06	975	SVLNDILSR	0,7759
A03	HLA-A*31:06	1099	GTHWFVTQR	0,856
A03	HLA-A*31:09	34	RGVYYPDKVFR	0,5141
A03	HLA-A*31:09	35	GVYYPDKVFR	0,663
A03	HLA-A*31:09	36	VYYPDKVFR	0,7682
A03	HLA-A*31:09	150	KSWMESEFR	0,517
A03	HLA-A*31:09	182	KQGNFKNLR	0,6571
A03	HLA-A*31:09	311	GIYQTSNFR	0,5434
A03	HLA-A*31:09	346	RFASVYAWNR	0,6162
A03	HLA-A*31:09	349	SVYAWNRKR	0,8004
A03	HLA-A*31:09	395	VYADSFVIR	0,5259
A03	HLA-A*31:09	458	KSNLKPFER	0,8223
A03	HLA-A*31:09	558	KFLPFQQFGR	0,5629
A03	HLA-A*31:09	677	QTNSPRRAR	0,5307
A03	HLA-A*31:09	975	SVLNDILSR	0,5479
A03	HLA-A*31:09	1099	GTHWFVTQR	0,7562
A03	HLA-A*31:11	34	RGVYYPDKVFR	0,5574
A03	HLA-A*31:11	35	GVYYPDKVFR	0,7294
A03	HLA-A*31:11	36	VYYPDKVFR	0,8085
A03	HLA-A*31:11	150	KSWMESEFR	0,5583
A03	HLA-A*31:11	182	KQGNFKNLR	0,6717
A03	HLA-A*31:11	310	KGIYQTSNFR	0,5075
A03	HLA-A*31:11	311	GIYQTSNFR	0,6612
A03	HLA-A*31:11	319	RVQPTESIVR	0,5738
A03	HLA-A*31:11	346	RFASVYAWNR	0,7025
A03	HLA-A*31:11	349	SVYAWNRKR	0,8646
A03	HLA-A*31:11	395	VYADSFVIR	0,575
A03	HLA-A*31:11	446	GGNYNYLYR	0,5086
A03	HLA-A*31:11	454	RLFRKSNLK	0,5237
A03	HLA-A*31:11	458	KSNLKPFER	0,8687
A03	HLA-A*31:11	558	KFLPFQQFGR	0,6235
A03	HLA-A*31:11	677	QTNSPRRAR	0,6557
A03	HLA-A*31:11	975	SVLNDILSR	0,6352
A03	HLA-A*31:11	1099	GTHWFVTQR	0,837
A03	HLA-A*33:01	36	VYYPDKVFR	0,6674
A03	HLA-A*33:01	265	YYVGYLQPR	0,6111
A03	HLA-A*33:01	349	SVYAWNRKR	0,5816
A03	HLA-A*33:01	394	NVYADSFVIR	0,575
A03	HLA-A*33:01	395	VYADSFVIR	0,5728
A03	HLA-A*33:01	448	NYNYLYRLFR	0,5281
A03	HLA-A*33:04	36	VYYPDKVFR	0,6674
A03	HLA-A*33:04	265	YYVGYLQPR	0,6111
A03	HLA-A*33:04	349	SVYAWNRKR	0,5816
A03	HLA-A*33:04	394	NVYADSFVIR	0,575
A03	HLA-A*33:04	395	VYADSFVIR	0,5728
A03	HLA-A*33:04	448	NYNYLYRLFR	0,5281

A03	HLA-A*33:05	36	VYYPDKVFR	0,6674
A03	HLA-A*33:05	265	YYVGYLQPR	0,6111
A03	HLA-A*33:05	349	SVYAWNRKR	0,5816
A03	HLA-A*33:05	394	NVYADSFVIR	0,575
A03	HLA-A*33:05	395	VYADSFVIR	0,5728
A03	HLA-A*33:05	448	NYNYLYRLFR	0,5281
A03	HLA-A*33:06	36	VYYPDKVFR	0,6561
A03	HLA-A*33:06	265	YYVGYLQPR	0,5103
A03	HLA-A*33:06	349	SVYAWNRKR	0,7608
A03	HLA-A*33:06	394	NVYADSFVIR	0,5705
A03	HLA-A*33:06	395	VYADSFVIR	0,5307
A03	HLA-A*33:06	677	QTNSPRRAR	0,5806
A03	HLA-A*33:06	975	SVLNDILSR	0,5342
A03	HLA-A*33:06	1099	GTHWFVTQR	0,5592
A03	HLA-A*33:07	36	VYYPDKVFR	0,6674
A03	HLA-A*33:07	265	YYVGYLQPR	0,6111
A03	HLA-A*33:07	349	SVYAWNRKR	0,5816
A03	HLA-A*33:07	394	NVYADSFVIR	0,575
A03	HLA-A*33:07	395	VYADSFVIR	0,5728
A03	HLA-A*33:07	448	NYNYLYRLFR	0,5281
A03	HLA-A*34:02	30	NSFTRGVYY	0,7033
A03	HLA-A*34:02	35	GVYYPDKVFR	0,5826
A03	HLA-A*34:02	69	HVSGTNGTK	0,6254
A03	HLA-A*34:02	88	DGVYFASTEK	0,5696
A03	HLA-A*34:02	89	GVYFASTEK	0,81
A03	HLA-A*34:02	142	GVYYHKNNK	0,7107
A03	HLA-A*34:02	192	FVFKNIDGY	0,5463
A03	HLA-A*34:02	198	DGYFKIYSK	0,5064
A03	HLA-A*34:02	258	WTAGAAAYY	0,585
A03	HLA-A*34:02	302	TLKSFTVEK	0,7513
A03	HLA-A*34:02	311	GIYQTSNFR	0,5989
A03	HLA-A*34:02	349	SVYAWNRKR	0,7569
A03	HLA-A*34:02	370	NSASFSTFK	0,7929
A03	HLA-A*34:02	394	NVYADSFVIR	0,553
A03	HLA-A*34:02	409	QIAPGQTGK	0,7709
A03	HLA-A*34:02	724	TEILPVSMTK	0,6467
A03	HLA-A*34:02	725	EILPVSMTK	0,8798
A03	HLA-A*34:02	782	FAQVKQIYK	0,569
A03	HLA-A*34:02	787	QIYKTPPIK	0,744
A03	HLA-A*34:02	827	TLADAGFIK	0,6884
A03	HLA-A*34:02	975	SVLNDILSR	0,7688
A03	HLA-A*34:02	1020	ASANLAATK	0,5816
A03	HLA-A*34:02	1064	HVTYVPAQEK	0,7046
A03	HLA-A*34:02	1065	VTYVPAQEK	0,8154
A03	HLA-A*34:02	1099	GTHWFVTQR	0,5926
A03	HLA-A*34:02	1173	NASVVNIQK	0,5921
A03	HLA-A*34:03	30	NSFTRGVYY	0,7033
A03	HLA-A*34:03	35	GVYYPDKVFR	0,5826
A03	HLA-A*34:03	69	HVSGTNGTK	0,6254
A03	HLA-A*34:03	88	DGVYFASTEK	0,5696

A03	HLA-A*34:03	89	GVYFASTEK	0,81
A03	HLA-A*34:03	142	GVYYHKNNK	0,7107
A03	HLA-A*34:03	192	FVFKNIDGY	0,5463
A03	HLA-A*34:03	198	DGYFKIYSK	0,5064
A03	HLA-A*34:03	258	WTAGAAAYY	0,585
A03	HLA-A*34:03	302	TLKSFTVEK	0,7513
A03	HLA-A*34:03	311	GIYQTSNFR	0,5989
A03	HLA-A*34:03	349	SVYAWNRKR	0,7569
A03	HLA-A*34:03	370	NSASFSTFK	0,7929
A03	HLA-A*34:03	394	NVYADSFVIR	0,553
A03	HLA-A*34:03	409	QIAPGQTGK	0,7709
A03	HLA-A*34:03	724	TEILPVSMTK	0,6467
A03	HLA-A*34:03	725	EILPVSMTK	0,8798
A03	HLA-A*34:03	782	FAQVKQIYK	0,569
A03	HLA-A*34:03	787	QIYKTPPIK	0,744
A03	HLA-A*34:03	827	TLADAGFIK	0,6884
A03	HLA-A*34:03	975	SVLNDILSR	0,7688
A03	HLA-A*34:03	1020	ASANLAATK	0,5816
A03	HLA-A*34:03	1064	HVTYVPAQEK	0,7046
A03	HLA-A*34:03	1065	VTYVPAQEK	0,8154
A03	HLA-A*34:03	1099	GTHWFVTQR	0,5926
A03	HLA-A*34:03	1173	NASVVNIQK	0,5921
A03	HLA-A*34:04	30	NSFTRGVYY	0,7033
A03	HLA-A*34:04	35	GVYYPDKVFR	0,5826
A03	HLA-A*34:04	69	HVSGTNGTK	0,6254
A03	HLA-A*34:04	88	DGVYFASTEK	0,5696
A03	HLA-A*34:04	89	GVYFASTEK	0,81
A03	HLA-A*34:04	142	GVYYHKNNK	0,7107
A03	HLA-A*34:04	192	FVFKNIDGY	0,5463
A03	HLA-A*34:04	198	DGYFKIYSK	0,5064
A03	HLA-A*34:04	258	WTAGAAAYY	0,585
A03	HLA-A*34:04	302	TLKSFTVEK	0,7513
A03	HLA-A*34:04	311	GIYQTSNFR	0,5989
A03	HLA-A*34:04	349	SVYAWNRKR	0,7569
A03	HLA-A*34:04	370	NSASFSTFK	0,7929
A03	HLA-A*34:04	394	NVYADSFVIR	0,553
A03	HLA-A*34:04	409	QIAPGQTGK	0,7709
A03	HLA-A*34:04	724	TEILPVSMTK	0,6467
A03	HLA-A*34:04	725	EILPVSMTK	0,8798
A03	HLA-A*34:04	782	FAQVKQIYK	0,569
A03	HLA-A*34:04	787	QIYKTPPIK	0,744
A03	HLA-A*34:04	827	TLADAGFIK	0,6884
A03	HLA-A*34:04	975	SVLNDILSR	0,7688
A03	HLA-A*34:04	1020	ASANLAATK	0,5816
A03	HLA-A*34:04	1064	HVTYVPAQEK	0,7046
A03	HLA-A*34:04	1065	VTYVPAQEK	0,8154
A03	HLA-A*34:04	1099	GTHWFVTQR	0,5926
A03	HLA-A*34:04	1173	NASVVNIQK	0,5921
A03	HLA-A*34:06	30	NSFTRGVYY	0,6353
A03	HLA-A*34:06	192	FVFKNIDGY	0,6689

A03	HLA-A*34:06	258	WTAGAAAYY	0,787
A03	HLA-A*34:06	349	SVYAWNRKR	0,6008
A03	HLA-A*34:06	360	NCVADYSVLY	0,5204
A03	HLA-A*34:06	361	CVADYSVLY	0,6106
A03	HLA-A*34:06	604	TSNQVAVLY	0,5152
A03	HLA-A*34:06	725	EILPVSMTK	0,7361
A03	HLA-A*34:06	865	LTDEMIAQY	0,516
A03	HLA-A*34:06	975	SVLNDILSR	0,591
A03	HLA-A*66:01	192	FVFKNIDGY	0,6341
A03	HLA-A*66:01	258	WTAGAAAYY	0,7115
A03	HLA-A*66:01	725	EILPVSMTK	0,6045
A03	HLA-A*66:02	69	HVSGTNGTK	0,5701
A03	HLA-A*66:02	192	FVFKNIDGY	0,6529
A03	HLA-A*66:02	258	WTAGAAAYY	0,7098
A03	HLA-A*66:02	349	SVYAWNRKR	0,538
A03	HLA-A*66:02	725	EILPVSMTK	0,694
A03	HLA-A*66:03	192	FVFKNIDGY	0,6992
A03	HLA-A*66:03	258	WTAGAAAYY	0,7192
A03	HLA-A*66:03	349	SVYAWNRKR	0,5229
A03	HLA-A*66:04	192	FVFKNIDGY	0,5137
A03	HLA-A*66:04	258	WTAGAAAYY	0,5766
A03	HLA-A*66:04	725	EILPVSMTK	0,603
A03	HLA-A*68:01	30	NSFTRGVYY	0,668
A03	HLA-A*68:01	35	GVYYPDKVFR	0,7011
A03	HLA-A*68:01	69	HVSGTNGTK	0,6905
A03	HLA-A*68:01	69	HVSGTNGTKR	0,6265
A03	HLA-A*68:01	88	DGVYFASTEK	0,6542
A03	HLA-A*68:01	89	GVYFASTEK	0,6612
A03	HLA-A*68:01	94	STEKSNIIR	0,5226
A03	HLA-A*68:01	192	FVFKNIDGY	0,6109
A03	HLA-A*68:01	198	DGYFKIYSK	0,5463
A03	HLA-A*68:01	228	DLPIGINITR	0,6101
A03	HLA-A*68:01	258	WTAGAAAYY	0,739
A03	HLA-A*68:01	302	TLKSFTVEK	0,5381
A03	HLA-A*68:01	311	GIYQTSNFR	0,6323
A03	HLA-A*68:01	347	FASVYAWNR	0,6502
A03	HLA-A*68:01	349	SVYAWNRKR	0,7833
A03	HLA-A*68:01	361	CVADYSVLY	0,538
A03	HLA-A*68:01	369	YNSASFSTFK	0,5211
A03	HLA-A*68:01	370	NSASFSTFK	0,8693
A03	HLA-A*68:01	394	NVYADSFVIR	0,8218
A03	HLA-A*68:01	400	FVIRGDEVR	0,7176
A03	HLA-A*68:01	409	QIAPGQTGK	0,6056
A03	HLA-A*68:01	501	NGVGYQPYR	0,5133
A03	HLA-A*68:01	568	DIADTTDAVR	0,5526
A03	HLA-A*68:01	604	TSNQVAVLY	0,5237
A03	HLA-A*68:01	637	STGSNVFQTR	0,6834
A03	HLA-A*68:01	677	QTNSPRRAR	0,5628
A03	HLA-A*68:01	725	EILPVSMTK	0,8883
A03	HLA-A*68:01	777	NTQEVFAQVK	0,5821

A03	HLA-A*68:01	782	FAQVKQIYK	0,557
A03	HLA-A*68:01	817	FIEDLLFNK	0,5167
A03	HLA-A*68:01	827	TLADAGFIK	0,6674
A03	HLA-A*68:01	975	SVLNDILSR	0,7755
A03	HLA-A*68:01	1020	ASANLAATK	0,5422
A03	HLA-A*68:01	1064	HVTYVPAQEK	0,6592
A03	HLA-A*68:01	1065	VTYVPAQEK	0,6604
A03	HLA-A*68:01	1099	GTHWFVTQR	0,7781
A03	HLA-A*68:01	1173	NASVVNIQK	0,8236
A03	HLA-A*68:03	30	NSFTRGVYY	0,52
A03	HLA-A*68:03	35	GVYYPDKVFR	0,583
A03	HLA-A*68:03	69	HVSGTNGTKR	0,5195
A03	HLA-A*68:03	192	FVFKNIDGY	0,5247
A03	HLA-A*68:03	228	DLPIGINITR	0,5626
A03	HLA-A*68:03	258	WTAGAAAYY	0,6587
A03	HLA-A*68:03	311	GIYQTSNFR	0,5487
A03	HLA-A*68:03	347	FASVYAWNR	0,5769
A03	HLA-A*68:03	349	SVYAWNRKR	0,6915
A03	HLA-A*68:03	370	NSASFSTFK	0,6811
A03	HLA-A*68:03	394	NVYADSFVIR	0,7431
A03	HLA-A*68:03	400	FVIRGDEVR	0,6382
A03	HLA-A*68:03	637	STGSNVFQTR	0,5981
A03	HLA-A*68:03	725	EILPVSMTK	0,7062
A03	HLA-A*68:03	975	SVLNDILSR	0,7019
A03	HLA-A*68:03	1099	GTHWFVTQR	0,6828
A03	HLA-A*68:03	1173	NASVVNIQK	0,5167
A03	HLA-A*68:04	35	GVYYPDKVFR	0,5995
A03	HLA-A*68:04	69	HVSGTNGTKR	0,5678
A03	HLA-A*68:04	228	DLPIGINITR	0,5843
A03	HLA-A*68:04	258	WTAGAAAYY	0,5018
A03	HLA-A*68:04	311	GIYQTSNFR	0,5866
A03	HLA-A*68:04	347	FASVYAWNR	0,6029
A03	HLA-A*68:04	349	SVYAWNRKR	0,7626
A03	HLA-A*68:04	370	NSASFSTFK	0,6296
A03	HLA-A*68:04	394	NVYADSFVIR	0,7377
A03	HLA-A*68:04	400	FVIRGDEVR	0,6688
A03	HLA-A*68:04	568	DIADTTDAVR	0,5213
A03	HLA-A*68:04	637	STGSNVFQTR	0,6194
A03	HLA-A*68:04	677	QTNSPRRAR	0,5976
A03	HLA-A*68:04	725	EILPVSMTK	0,6512
A03	HLA-A*68:04	975	SVLNDILSR	0,7053
A03	HLA-A*68:04	1099	GTHWFVTQR	0,6927
A03	HLA-A*68:08	30	NSFTRGVYY	0,6319
A03	HLA-A*68:08	35	GVYYPDKVFR	0,6146
A03	HLA-A*68:08	89	GVYFASTEK	0,5888
A03	HLA-A*68:08	192	FVFKNIDGY	0,5341
A03	HLA-A*68:08	258	WTAGAAAYY	0,6296
A03	HLA-A*68:08	311	GIYQTSNFR	0,5259
A03	HLA-A*68:08	349	SVYAWNRKR	0,7003
A03	HLA-A*68:08	361	CVADYSVLY	0,5107

A03	HLA-A*68:08	370	NSASFSTFK	0,7548
A03	HLA-A*68:08	394	NVYADSFVIR	0,7003
A03	HLA-A*68:08	725	EILPVSMTK	0,8497
A03	HLA-A*68:08	817	FIEDLLFNK	0,54
A03	HLA-A*68:08	827	TLADAGFIK	0,6538
A03	HLA-A*68:08	975	SVLNDILSR	0,7898
A03	HLA-A*68:08	1065	VTYVPAQEK	0,5951
A03	HLA-A*68:08	1099	GTHWFVTQR	0,627
A03	HLA-A*68:08	1173	NASVVNIQK	0,7036
A03	HLA-A*68:09	30	NSFTRGVYY	0,5551
A03	HLA-A*68:09	35	GVYYPDKVFR	0,5657
A03	HLA-A*68:09	89	GVYFASTEK	0,5852
A03	HLA-A*68:09	94	STEKSNIIR	0,5242
A03	HLA-A*68:09	258	WTAGAAAYY	0,6062
A03	HLA-A*68:09	302	TLKSFTVEK	0,5606
A03	HLA-A*68:09	311	GIYQTSNFR	0,5449
A03	HLA-A*68:09	349	SVYAWNRKR	0,6482
A03	HLA-A*68:09	370	NSASFSTFK	0,7717
A03	HLA-A*68:09	394	NVYADSFVIR	0,6549
A03	HLA-A*68:09	409	QIAPGQTGK	0,5013
A03	HLA-A*68:09	725	EILPVSMTK	0,8372
A03	HLA-A*68:09	782	FAQVKQIYK	0,5222
A03	HLA-A*68:09	817	FIEDLLFNK	0,5307
A03	HLA-A*68:09	827	TLADAGFIK	0,7002
A03	HLA-A*68:09	975	SVLNDILSR	0,773
A03	HLA-A*68:09	1065	VTYVPAQEK	0,5868
A03	HLA-A*68:09	1099	GTHWFVTQR	0,6191
A03	HLA-A*68:09	1173	NASVVNIQK	0,6784
A03	HLA-A*68:10	35	GVYYPDKVFR	0,8083
A03	HLA-A*68:10	69	HVSGTNGTK	0,53
A03	HLA-A*68:10	89	GVYFASTEK	0,8449
A03	HLA-A*68:10	142	GVYYHKNNK	0,6813
A03	HLA-A*68:10	150	KSWMESEFR	0,5571
A03	HLA-A*68:10	302	TLKSFTVEK	0,7187
A03	HLA-A*68:10	310	KGIYQTSNFR	0,5025
A03	HLA-A*68:10	311	GIYQTSNFR	0,7383
A03	HLA-A*68:10	319	RVQPTESIVR	0,5288
A03	HLA-A*68:10	348	ASVYAWNRK	0,6444
A03	HLA-A*68:10	349	SVYAWNRKR	0,7606
A03	HLA-A*68:10	369	YNSASFSTFK	0,5505
A03	HLA-A*68:10	370	NSASFSTFK	0,782
A03	HLA-A*68:10	394	NVYADSFVIR	0,5174
A03	HLA-A*68:10	408	RQIAPGQTGK	0,6091
A03	HLA-A*68:10	409	QIAPGQTGK	0,6387
A03	HLA-A*68:10	454	RLFRKSNLK	0,6427
A03	HLA-A*68:10	458	KSNLKPFER	0,7028
A03	HLA-A*68:10	529	KSTNLVKNK	0,5693
A03	HLA-A*68:10	550	GVLTESNKK	0,5463
A03	HLA-A*68:10	637	STGSNVFQTR	0,6581
A03	HLA-A*68:10	724	TEILPVSMTK	0,5558

A03	HLA-A*68:10	725	EILPVSMTK	0,6932
A03	HLA-A*68:10	757	GSFCTQLNR	0,5531
A03	HLA-A*68:10	782	FAQVKQIYK	0,5621
A03	HLA-A*68:10	787	QIYKTPPIK	0,5872
A03	HLA-A*68:10	826	VTLADAGFIK	0,6153
A03	HLA-A*68:10	827	TLADAGFIK	0,7116
A03	HLA-A*68:10	939	SSTASALGK	0,5627
A03	HLA-A*68:10	975	SVLNDILSR	0,813
A03	HLA-A*68:10	1019	RASANLAATK	0,5906
A03	HLA-A*68:10	1020	ASANLAATK	0,8515
A03	HLA-A*68:10	1064	HVTYVPAQEK	0,6145
A03	HLA-A*68:10	1065	VTYVPAQEK	0,8181
A03	HLA-A*68:10	1099	GTHWFVTQR	0,8652
A03	HLA-A*68:10	1173	NASVVNIQK	0,5721
A03	HLA-A*68:12	30	NSFTRGVYY	0,668
A03	HLA-A*68:12	35	GVYYPDKVFR	0,7011
A03	HLA-A*68:12	69	HVSGTNGTK	0,6905
A03	HLA-A*68:12	69	HVSGTNGTKR	0,6265
A03	HLA-A*68:12	88	DGVYFASTEK	0,6542
A03	HLA-A*68:12	89	GVYFASTEK	0,6612
A03	HLA-A*68:12	94	STEKSNIIR	0,5226
A03	HLA-A*68:12	192	FVFKNIDGY	0,6109
A03	HLA-A*68:12	198	DGYFKIYSK	0,5463
A03	HLA-A*68:12	228	DLPIGINITR	0,6101
A03	HLA-A*68:12	258	WTAGAAAYY	0,739
A03	HLA-A*68:12	302	TLKSFTVEK	0,5381
A03	HLA-A*68:12	311	GIYQTSNFR	0,6323
A03	HLA-A*68:12	347	FASVYAWNR	0,6502
A03	HLA-A*68:12	349	SVYAWNRKR	0,7833
A03	HLA-A*68:12	361	CVADYSVLY	0,538
A03	HLA-A*68:12	369	YNSASFSTFK	0,5211
A03	HLA-A*68:12	370	NSASFSTFK	0,8693
A03	HLA-A*68:12	394	NVYADSFVIR	0,8218
A03	HLA-A*68:12	400	FVIRGDEVR	0,7176
A03	HLA-A*68:12	409	QIAPGQTGK	0,6056
A03	HLA-A*68:12	501	NGVGYQPYR	0,5133
A03	HLA-A*68:12	568	DIADTTDAVR	0,5526
A03	HLA-A*68:12	604	TSNQVAVLY	0,5237
A03	HLA-A*68:12	637	STGSNVFQTR	0,6834
A03	HLA-A*68:12	677	QTNSPRRAR	0,5628
A03	HLA-A*68:12	725	EILPVSMTK	0,8883
A03	HLA-A*68:12	777	NTQEVFAQVK	0,5821
A03	HLA-A*68:12	782	FAQVKQIYK	0,557
A03	HLA-A*68:12	817	FIEDLLFNK	0,5167
A03	HLA-A*68:12	827	TLADAGFIK	0,6674
A03	HLA-A*68:12	975	SVLNDILSR	0,7755
A03	HLA-A*68:12	1020	ASANLAATK	0,5422
A03	HLA-A*68:12	1064	HVTYVPAQEK	0,6592
A03	HLA-A*68:12	1065	VTYVPAQEK	0,6604
A03	HLA-A*68:12	1099	GTHWFVTQR	0,7781

A03	HLA-A*68:12	1173	NASVVNIQK	0,8236
A03	HLA-A*68:13	35	GVYYPDKVFR	0,8316
A03	HLA-A*68:13	69	HVSGTNGTK	0,5191
A03	HLA-A*68:13	89	GVYFASTEK	0,8528
A03	HLA-A*68:13	142	GVYYHKNNK	0,6657
A03	HLA-A*68:13	258	WTAGAAAYY	0,5568
A03	HLA-A*68:13	302	TLKSFTVEK	0,7097
A03	HLA-A*68:13	311	GIYQTSNFR	0,7417
A03	HLA-A*68:13	348	ASVYAWNRK	0,588
A03	HLA-A*68:13	349	SVYAWNRKR	0,7769
A03	HLA-A*68:13	369	YNSASFSTFK	0,5945
A03	HLA-A*68:13	370	NSASFSTFK	0,7984
A03	HLA-A*68:13	394	NVYADSFVIR	0,6004
A03	HLA-A*68:13	400	FVIRGDEVR	0,5034
A03	HLA-A*68:13	409	QIAPGQTGK	0,6035
A03	HLA-A*68:13	454	RLFRKSNLK	0,5075
A03	HLA-A*68:13	458	KSNLKPFER	0,5406
A03	HLA-A*68:13	637	STGSNVFQTR	0,6827
A03	HLA-A*68:13	724	TEILPVSMTK	0,541
A03	HLA-A*68:13	725	EILPVSMTK	0,7319
A03	HLA-A*68:13	757	GSFCTQLNR	0,5489
A03	HLA-A*68:13	782	FAQVKQIYK	0,5799
A03	HLA-A*68:13	787	QIYKTPPIK	0,5619
A03	HLA-A*68:13	826	VTLADAGFIK	0,6185
A03	HLA-A*68:13	827	TLADAGFIK	0,6995
A03	HLA-A*68:13	939	SSTASALGK	0,5057
A03	HLA-A*68:13	975	SVLNDILSR	0,8131
A03	HLA-A*68:13	1020	ASANLAATK	0,8167
A03	HLA-A*68:13	1064	HVTYVPAQEK	0,6451
A03	HLA-A*68:13	1065	VTYVPAQEK	0,823
A03	HLA-A*68:13	1099	GTHWFVTQR	0,8791
A03	HLA-A*68:13	1173	NASVVNIQK	0,58
A03	HLA-A*68:14	35	GVYYPDKVFR	0,8083
A03	HLA-A*68:14	69	HVSGTNGTK	0,53
A03	HLA-A*68:14	89	GVYFASTEK	0,8449
A03	HLA-A*68:14	142	GVYYHKNNK	0,6813
A03	HLA-A*68:14	150	KSWMESEFR	0,5571
A03	HLA-A*68:14	302	TLKSFTVEK	0,7187
A03	HLA-A*68:14	310	KGIYQTSNFR	0,5025
A03	HLA-A*68:14	311	GIYQTSNFR	0,7383
A03	HLA-A*68:14	319	RVQPTESIVR	0,5288
A03	HLA-A*68:14	348	ASVYAWNRK	0,6444
A03	HLA-A*68:14	349	SVYAWNRKR	0,7606
A03	HLA-A*68:14	369	YNSASFSTFK	0,5505
A03	HLA-A*68:14	370	NSASFSTFK	0,782
A03	HLA-A*68:14	394	NVYADSFVIR	0,5174
A03	HLA-A*68:14	408	RQIAPGQTGK	0,6091
A03	HLA-A*68:14	409	QIAPGQTGK	0,6387
A03	HLA-A*68:14	454	RLFRKSNLK	0,6427
A03	HLA-A*68:14	458	KSNLKPFER	0,7028

A03	HLA-A*68:14	529	KSTNLVKNK	0,5693
A03	HLA-A*68:14	550	GVLTESNKK	0,5463
A03	HLA-A*68:14	637	STGSNVFQTR	0,6581
A03	HLA-A*68:14	724	TEILPVSMTK	0,5558
A03	HLA-A*68:14	725	EILPVSMTK	0,6932
A03	HLA-A*68:14	757	GSFCTQLNR	0,5531
A03	HLA-A*68:14	782	FAQVKQIYK	0,5621
A03	HLA-A*68:14	787	QIYKTPPIK	0,5872
A03	HLA-A*68:14	826	VTLADAGFIK	0,6153
A03	HLA-A*68:14	827	TLADAGFIK	0,7116
A03	HLA-A*68:14	939	SSTASALGK	0,5627
A03	HLA-A*68:14	975	SVLNDILSR	0,813
A03	HLA-A*68:14	1019	RASANLAATK	0,5906
A03	HLA-A*68:14	1020	ASANLAATK	0,8515
A03	HLA-A*68:14	1064	HVTYVPAQEK	0,6145
A03	HLA-A*68:14	1065	VTYVPAQEK	0,8181
A03	HLA-A*68:14	1099	GTHWFVTQR	0,8652
A03	HLA-A*68:14	1173	NASVVNIQK	0,5721
A03	HLA-A*68:16	30	NSFTRGVYY	0,668
A03	HLA-A*68:16	35	GVYYPDKVFR	0,7011
A03	HLA-A*68:16	69	HVSGTNGTK	0,6905
A03	HLA-A*68:16	69	HVSGTNGTKR	0,6265
A03	HLA-A*68:16	88	DGVYFASTEK	0,6542
A03	HLA-A*68:16	89	GVYFASTEK	0,6612
A03	HLA-A*68:16	94	STEKSNIIR	0,5226
A03	HLA-A*68:16	192	FVFKNIDGY	0,6109
A03	HLA-A*68:16	198	DGYFKIYSK	0,5463
A03	HLA-A*68:16	228	DLPIGINITR	0,6101
A03	HLA-A*68:16	258	WTAGAAAYY	0,739
A03	HLA-A*68:16	302	TLKSFTVEK	0,5381
A03	HLA-A*68:16	311	GIYQTSNFR	0,6323
A03	HLA-A*68:16	347	FASVYAWNR	0,6502
A03	HLA-A*68:16	349	SVYAWNRKR	0,7833
A03	HLA-A*68:16	361	CVADYSVLY	0,538
A03	HLA-A*68:16	369	YNSASFSTFK	0,5211
A03	HLA-A*68:16	370	NSASFSTFK	0,8693
A03	HLA-A*68:16	394	NVYADSFVIR	0,8218
A03	HLA-A*68:16	400	FVIRGDEVR	0,7176
A03	HLA-A*68:16	409	QIAPGQTGK	0,6056
A03	HLA-A*68:16	501	NGVGYQPYR	0,5133
A03	HLA-A*68:16	568	DIADTTDAVR	0,5526
A03	HLA-A*68:16	604	TSNQVAVLY	0,5237
A03	HLA-A*68:16	637	STGSNVFQTR	0,6834
A03	HLA-A*68:16	677	QTNSPRRAR	0,5628
A03	HLA-A*68:16	725	EILPVSMTK	0,8883
A03	HLA-A*68:16	777	NTQEVFAQVK	0,5821
A03	HLA-A*68:16	782	FAQVKQIYK	0,557
A03	HLA-A*68:16	817	FIEDLLFNK	0,5167
A03	HLA-A*68:16	827	TLADAGFIK	0,6674
A03	HLA-A*68:16	975	SVLNDILSR	0,7755

A03	HLA-A*68:16	1020	ASANLAATK	0,5422
A03	HLA-A*68:16	1064	HVTYVPAQEK	0,6592
A03	HLA-A*68:16	1065	VTYVPAQEK	0,6604
A03	HLA-A*68:16	1099	GTHWFVTQR	0,7781
A03	HLA-A*68:16	1173	NASVVNIQK	0,8236
A03	HLA-A*68:19	30	NSFTRGVYY	0,668
A03	HLA-A*68:19	35	GVYYPDKVFR	0,7011
A03	HLA-A*68:19	69	HVSGTNGTK	0,6905
A03	HLA-A*68:19	69	HVSGTNGTKR	0,6265
A03	HLA-A*68:19	88	DGVYFASTEK	0,6542
A03	HLA-A*68:19	89	GVYFASTEK	0,6612
A03	HLA-A*68:19	94	STEKSNIIR	0,5226
A03	HLA-A*68:19	192	FVFKNIDGY	0,6109
A03	HLA-A*68:19	198	DGYFKIYSK	0,5463
A03	HLA-A*68:19	228	DLPIGINITR	0,6101
A03	HLA-A*68:19	258	WTAGAAAYY	0,739
A03	HLA-A*68:19	302	TLKSFTVEK	0,5381
A03	HLA-A*68:19	311	GIYQTSNFR	0,6323
A03	HLA-A*68:19	347	FASVYAWNR	0,6502
A03	HLA-A*68:19	349	SVYAWNRKR	0,7833
A03	HLA-A*68:19	361	CVADYSVLY	0,538
A03	HLA-A*68:19	369	YNSASFSTFK	0,5211
A03	HLA-A*68:19	370	NSASFSTFK	0,8693
A03	HLA-A*68:19	394	NVYADSFVIR	0,8218
A03	HLA-A*68:19	400	FVIRGDEVR	0,7176
A03	HLA-A*68:19	409	QIAPGQTGK	0,6056
A03	HLA-A*68:19	501	NGVGYQPYR	0,5133
A03	HLA-A*68:19	568	DIADTTDAVR	0,5526
A03	HLA-A*68:19	604	TSNQVAVLY	0,5237
A03	HLA-A*68:19	637	STGSNVFQTR	0,6834
A03	HLA-A*68:19	677	QTNSPRRAR	0,5628
A03	HLA-A*68:19	725	EILPVSMTK	0,8883
A03	HLA-A*68:19	777	NTQEVFAQVK	0,5821
A03	HLA-A*68:19	782	FAQVKQIYK	0,557
A03	HLA-A*68:19	817	FIEDLLFNK	0,5167
A03	HLA-A*68:19	827	TLADAGFIK	0,6674
A03	HLA-A*68:19	975	SVLNDILSR	0,7755
A03	HLA-A*68:19	1020	ASANLAATK	0,5422
A03	HLA-A*68:19	1064	HVTYVPAQEK	0,6592
A03	HLA-A*68:19	1065	VTYVPAQEK	0,6604
A03	HLA-A*68:19	1099	GTHWFVTQR	0,7781
A03	HLA-A*68:19	1173	NASVVNIQK	0,8236
A03	HLA-A*68:21	30	NSFTRGVYY	0,668
A03	HLA-A*68:21	35	GVYYPDKVFR	0,7011
A03	HLA-A*68:21	69	HVSGTNGTK	0,6905
A03	HLA-A*68:21	69	HVSGTNGTKR	0,6265
A03	HLA-A*68:21	88	DGVYFASTEK	0,6542
A03	HLA-A*68:21	89	GVYFASTEK	0,6612
A03	HLA-A*68:21	94	STEKSNIIR	0,5226
A03	HLA-A*68:21	192	FVFKNIDGY	0,6109

A03	HLA-A*68:21	198	DGYFKIYSK	0,5463
A03	HLA-A*68:21	228	DLPIGINITR	0,6101
A03	HLA-A*68:21	258	WTAGAAAYY	0,739
A03	HLA-A*68:21	302	TLKSFTVEK	0,5381
A03	HLA-A*68:21	311	GIYQTSNFR	0,6323
A03	HLA-A*68:21	347	FASVYAWNR	0,6502
A03	HLA-A*68:21	349	SVYAWNRKR	0,7833
A03	HLA-A*68:21	361	CVADYSVLY	0,538
A03	HLA-A*68:21	369	YNSASFSTFK	0,5211
A03	HLA-A*68:21	370	NSASFSTFK	0,8693
A03	HLA-A*68:21	394	NVYADSFVIR	0,8218
A03	HLA-A*68:21	400	FVIRGDEVR	0,7176
A03	HLA-A*68:21	409	QIAPGQTGK	0,6056
A03	HLA-A*68:21	501	NGVGYQPYR	0,5133
A03	HLA-A*68:21	568	DIADTTDAVR	0,5526
A03	HLA-A*68:21	604	TSNQVAVLY	0,5237
A03	HLA-A*68:21	637	STGSNVFQTR	0,6834
A03	HLA-A*68:21	677	QTNSPRRAR	0,5628
A03	HLA-A*68:21	725	EILPVSMTK	0,8883
A03	HLA-A*68:21	777	NTQEVFAQVK	0,5821
A03	HLA-A*68:21	782	FAQVKQIYK	0,557
A03	HLA-A*68:21	817	FIEDLLFNK	0,5167
A03	HLA-A*68:21	827	TLADAGFIK	0,6674
A03	HLA-A*68:21	975	SVLNDILSR	0,7755
A03	HLA-A*68:21	1020	ASANLAATK	0,5422
A03	HLA-A*68:21	1064	HVTYVPAQEK	0,6592
A03	HLA-A*68:21	1065	VTYVPAQEK	0,6604
A03	HLA-A*68:21	1099	GTHWFVTQR	0,7781
A03	HLA-A*68:21	1173	NASVVNIQK	0,8236
A03	HLA-A*68:22	30	NSFTRGVYY	0,668
A03	HLA-A*68:22	35	GVYYPDKVFR	0,7011
A03	HLA-A*68:22	69	HVSGTNGTK	0,6905
A03	HLA-A*68:22	69	HVSGTNGTKR	0,6265
A03	HLA-A*68:22	88	DGVYFASTEK	0,6542
A03	HLA-A*68:22	89	GVYFASTEK	0,6612
A03	HLA-A*68:22	94	STEKSNIIR	0,5226
A03	HLA-A*68:22	192	FVFKNIDGY	0,6109
A03	HLA-A*68:22	198	DGYFKIYSK	0,5463
A03	HLA-A*68:22	228	DLPIGINITR	0,6101
A03	HLA-A*68:22	258	WTAGAAAYY	0,739
A03	HLA-A*68:22	302	TLKSFTVEK	0,5381
A03	HLA-A*68:22	311	GIYQTSNFR	0,6323
A03	HLA-A*68:22	347	FASVYAWNR	0,6502
A03	HLA-A*68:22	349	SVYAWNRKR	0,7833
A03	HLA-A*68:22	361	CVADYSVLY	0,538
A03	HLA-A*68:22	369	YNSASFSTFK	0,5211
A03	HLA-A*68:22	370	NSASFSTFK	0,8693
A03	HLA-A*68:22	394	NVYADSFVIR	0,8218
A03	HLA-A*68:22	400	FVIRGDEVR	0,7176
A03	HLA-A*68:22	409	QIAPGQTGK	0,6056

A03	HLA-A*68:22	501	NGVGYQPYR	0,5133
A03	HLA-A*68:22	568	DIADTTDAVR	0,5526
A03	HLA-A*68:22	604	TSNQVAVLY	0,5237
A03	HLA-A*68:22	637	STGSNVFQTR	0,6834
A03	HLA-A*68:22	677	QTNSPRRAR	0,5628
A03	HLA-A*68:22	725	EILPVSMTK	0,8883
A03	HLA-A*68:22	777	NTQEVFAQVK	0,5821
A03	HLA-A*68:22	782	FAQVKQIYK	0,557
A03	HLA-A*68:22	817	FIEDLLFNK	0,5167
A03	HLA-A*68:22	827	TLADAGFIK	0,6674
A03	HLA-A*68:22	975	SVLNDILSR	0,7755
A03	HLA-A*68:22	1020	ASANLAATK	0,5422
A03	HLA-A*68:22	1064	HVTYVPAQEK	0,6592
A03	HLA-A*68:22	1065	VTYVPAQEK	0,6604
A03	HLA-A*68:22	1099	GTHWFVTQR	0,7781
A03	HLA-A*68:22	1173	NASVVNIQK	0,8236
A03	HLA-A*68:24	30	NSFTRGVYY	0,668
A03	HLA-A*68:24	35	GVYYPDKVFR	0,7011
A03	HLA-A*68:24	69	HVSGTNGTK	0,6905
A03	HLA-A*68:24	69	HVSGTNGTKR	0,6265
A03	HLA-A*68:24	88	DGVYFASTEK	0,6542
A03	HLA-A*68:24	89	GVYFASTEK	0,6612
A03	HLA-A*68:24	94	STEKSNIIR	0,5226
A03	HLA-A*68:24	192	FVFKNIDGY	0,6109
A03	HLA-A*68:24	198	DGYFKIYSK	0,5463
A03	HLA-A*68:24	228	DLPIGINITR	0,6101
A03	HLA-A*68:24	258	WTAGAAAYY	0,739
A03	HLA-A*68:24	302	TLKSFTVEK	0,5381
A03	HLA-A*68:24	311	GIYQTSNFR	0,6323
A03	HLA-A*68:24	347	FASVYAWNR	0,6502
A03	HLA-A*68:24	349	SVYAWNRKR	0,7833
A03	HLA-A*68:24	361	CVADYSVLY	0,538
A03	HLA-A*68:24	369	YNSASFSTFK	0,5211
A03	HLA-A*68:24	370	NSASFSTFK	0,8693
A03	HLA-A*68:24	394	NVYADSFVIR	0,8218
A03	HLA-A*68:24	400	FVIRGDEVR	0,7176
A03	HLA-A*68:24	409	QIAPGQTGK	0,6056
A03	HLA-A*68:24	501	NGVGYQPYR	0,5133
A03	HLA-A*68:24	568	DIADTTDAVR	0,5526
A03	HLA-A*68:24	604	TSNQVAVLY	0,5237
A03	HLA-A*68:24	637	STGSNVFQTR	0,6834
A03	HLA-A*68:24	677	QTNSPRRAR	0,5628
A03	HLA-A*68:24	725	EILPVSMTK	0,8883
A03	HLA-A*68:24	777	NTQEVFAQVK	0,5821
A03	HLA-A*68:24	782	FAQVKQIYK	0,557
A03	HLA-A*68:24	817	FIEDLLFNK	0,5167
A03	HLA-A*68:24	827	TLADAGFIK	0,6674
A03	HLA-A*68:24	975	SVLNDILSR	0,7755
A03	HLA-A*68:24	1020	ASANLAATK	0,5422
A03	HLA-A*68:24	1064	HVTYVPAQEK	0,6592

A03	HLA-A*68:24	1065	VTYVPAQEK	0,6604
A03	HLA-A*68:24	1099	GTHWFVTQR	0,7781
A03	HLA-A*68:24	1173	NASVVNIQK	0,8236
A03	HLA-A*68:25	30	NSFTRGVYY	0,668
A03	HLA-A*68:25	35	GVYYPDKVFR	0,7011
A03	HLA-A*68:25	69	HVSGTNGTK	0,6905
A03	HLA-A*68:25	69	HVSGTNGTKR	0,6265
A03	HLA-A*68:25	88	DGVYFASTEK	0,6542
A03	HLA-A*68:25	89	GVYFASTEK	0,6612
A03	HLA-A*68:25	94	STEKSNIIR	0,5226
A03	HLA-A*68:25	192	FVFKNIDGY	0,6109
A03	HLA-A*68:25	198	DGYFKIYSK	0,5463
A03	HLA-A*68:25	228	DLPIGINITR	0,6101
A03	HLA-A*68:25	258	WTAGAAAYY	0,739
A03	HLA-A*68:25	302	TLKSFTVEK	0,5381
A03	HLA-A*68:25	311	GIYQTSNFR	0,6323
A03	HLA-A*68:25	347	FASVYAWNR	0,6502
A03	HLA-A*68:25	349	SVYAWNRKR	0,7833
A03	HLA-A*68:25	361	CVADYSVLY	0,538
A03	HLA-A*68:25	369	YNSASFSTFK	0,5211
A03	HLA-A*68:25	370	NSASFSTFK	0,8693
A03	HLA-A*68:25	394	NVYADSFVIR	0,8218
A03	HLA-A*68:25	400	FVIRGDEVR	0,7176
A03	HLA-A*68:25	409	QIAPGQTGK	0,6056
A03	HLA-A*68:25	501	NGVGYQPYR	0,5133
A03	HLA-A*68:25	568	DIADTTDAVR	0,5526
A03	HLA-A*68:25	604	TSNQVAVLY	0,5237
A03	HLA-A*68:25	637	STGSNVFQTR	0,6834
A03	HLA-A*68:25	677	QTNSPRRAR	0,5628
A03	HLA-A*68:25	725	EILPVSMTK	0,8883
A03	HLA-A*68:25	777	NTQEVFAQVK	0,5821
A03	HLA-A*68:25	782	FAQVKQIYK	0,557
A03	HLA-A*68:25	817	FIEDLLFNK	0,5167
A03	HLA-A*68:25	827	TLADAGFIK	0,6674
A03	HLA-A*68:25	975	SVLNDILSR	0,7755
A03	HLA-A*68:25	1020	ASANLAATK	0,5422
A03	HLA-A*68:25	1064	HVTYVPAQEK	0,6592
A03	HLA-A*68:25	1065	VTYVPAQEK	0,6604
A03	HLA-A*68:25	1099	GTHWFVTQR	0,7781
A03	HLA-A*68:25	1173	NASVVNIQK	0,8236
A03	HLA-A*68:26	258	WTAGAAAYY	0,5231
A03	HLA-A*68:26	370	NSASFSTFK	0,5588
A03	HLA-A*68:26	725	EILPVSMTK	0,699
A03	HLA-A*68:26	827	TLADAGFIK	0,5161
A03	HLA-A*74:01	349	SVYAWNRKR	0,536
A03	HLA-A*74:02	349	SVYAWNRKR	0,536
A03	HLA-A*74:03	349	SVYAWNRKR	0,536
A03	HLA-A*74:05	35	GVYYPDKVFR	0,5051
A03	HLA-A*74:05	349	SVYAWNRKR	0,5895
A03	HLA-A*74:05	454	RLFRKSNLK	0,5208

A03	HLA-A*74:05	1099	GTHWFVTQR	0,5226
A03	HLA-A*74:07	35	GVYYPDKVFR	0,5118
A03	HLA-A*74:07	349	SVYAWNRKR	0,6319
A03	HLA-A*74:07	1099	GTHWFVTQR	0,5316
A03	HLA-A*74:08	349	SVYAWNRKR	0,536
A03	HLA-A*74:09	349	SVYAWNRKR	0,536
A03	HLA-A*74:11	349	SVYAWNRKR	0,536
A24	HLA-A*23:01	57	PFFSNVTWF	0,9141
A24	HLA-A*23:01	78	RFDNPVLPF	0,8396
A24	HLA-A*23:01	143	VYYHKNNKSW	0,7142
A24	HLA-A*23:01	144	YYHKNNKSW	0,8036
A24	HLA-A*23:01	159	VYSSANNCTF	0,917
A24	HLA-A*23:01	169	EYVSQPFLM	0,8499
A24	HLA-A*23:01	193	VFKNIDGYF	0,7818
A24	HLA-A*23:01	203	IYSKHTPINL	0,6028
A24	HLA-A*23:01	264	AYYVGYLQPRTF	0,7912
A24	HLA-A*23:01	265	YYVGYLQPRTF	0,879
A24	HLA-A*23:01	268	GYLQPRTFLL	0,7046
A24	HLA-A*23:01	269	YLQPRTFLL	0,5573
A24	HLA-A*23:01	312	IYQTSNFRV	0,6966
A24	HLA-A*23:01	328	RFPNITNLCPF	0,8158
A24	HLA-A*23:01	368	LYNSASFSTF	0,8553
A24	HLA-A*23:01	395	VYADSFVI	0,5149
A24	HLA-A*23:01	448	NYNYLYRLF	0,8881
A24	HLA-A*23:01	488	CYFPLQSYGF	0,6136
A24	HLA-A*23:01	489	YFPLQSYGF	0,9108
A24	HLA-A*23:01	504	GYQPYRVVVLSF	0,667
A24	HLA-A*23:01	507	PYRVVVLSF	0,8298
A24	HLA-A*23:01	558	KFLPFQQF	0,7881
A24	HLA-A*23:01	634	RVYSTGSNVF	0,5782
A24	HLA-A*23:01	635	VYSTGSNVF	0,934
A24	HLA-A*23:01	788	IYKTPPIKDF	0,9066
A24	HLA-A*23:01	816	SFIEDLLF	0,7909
A24	HLA-A*23:01	880	GTITSGWTF	0,5317
A24	HLA-A*23:01	898	FAMQMAYRF	0,5028
A24	HLA-A*23:01	1066	TYVPAQEKNF	0,9478
A24	HLA-A*23:01	1094	VFVSNGTHW	0,8761
A24	HLA-A*23:01	1094	VFVSNGTHWF	0,8258
A24	HLA-A*23:01	1101	HWFVTQRNF	0,7845
A24	HLA-A*23:01	1137	VYDPLQPELDSF	0,7515
A24	HLA-A*23:01	1208	QYIKWPWYI	0,9638
A24	HLA-A*23:01	1208	QYIKWPWYIW	0,7814
A24	HLA-A*23:01	1211	KWPWYIWLGF	0,6037
A24	HLA-A*23:01	1216	IWLGFIAGL	0,5925
A24	HLA-A*23:02	57	PFFSNVTWF	0,8811
A24	HLA-A*23:02	143	VYYHKNNKSW	0,7728
A24	HLA-A*23:02	144	YYHKNNKSW	0,8613
A24	HLA-A*23:02	159	VYSSANNCTF	0,9503
A24	HLA-A*23:02	169	EYVSQPFLM	0,8797
A24	HLA-A*23:02	193	VFKNIDGYF	0,8456

A24	HLA-A*23:02	203	IYSKHTPINL	0,6962
A24	HLA-A*23:02	264	AYYVGYLQPRTF	0,759
A24	HLA-A*23:02	265	YYVGYLQPRTF	0,9004
A24	HLA-A*23:02	268	GYLQPRTFLL	0,6697
A24	HLA-A*23:02	269	YLQPRTFLL	0,5368
A24	HLA-A*23:02	312	IYQTSNFRV	0,6415
A24	HLA-A*23:02	328	RFPNITNLCPF	0,8669
A24	HLA-A*23:02	368	LYNSASFSTF	0,901
A24	HLA-A*23:02	448	NYNYLYRLF	0,9202
A24	HLA-A*23:02	488	CYFPLQSYGF	0,6273
A24	HLA-A*23:02	489	YFPLQSYGF	0,9384
A24	HLA-A*23:02	504	GYQPYRVVVLSF	0,6274
A24	HLA-A*23:02	507	PYRVVVLSF	0,8272
A24	HLA-A*23:02	634	RVYSTGSNVF	0,7093
A24	HLA-A*23:02	635	VYSTGSNVF	0,9475
A24	HLA-A*23:02	755	QYGSFCTQL	0,5573
A24	HLA-A*23:02	788	IYKTPPIKDF	0,9187
A24	HLA-A*23:02	1066	TYVPAQEKNF	0,9671
A24	HLA-A*23:02	1094	VFVSNGTHW	0,8829
A24	HLA-A*23:02	1094	VFVSNGTHWF	0,8701
A24	HLA-A*23:02	1101	HWFVTQRNF	0,7966
A24	HLA-A*23:02	1208	QYIKWPWYI	0,9423
A24	HLA-A*23:02	1208	QYIKWPWYIW	0,7679
A24	HLA-A*23:02	1211	KWPWYIWLGF	0,5976
A24	HLA-A*23:03	57	PFFSNVTWF	0,9141
A24	HLA-A*23:03	78	RFDNPVLPF	0,8396
A24	HLA-A*23:03	143	VYYHKNNKSW	0,7142
A24	HLA-A*23:03	144	YYHKNNKSW	0,8036
A24	HLA-A*23:03	159	VYSSANNCTF	0,917
A24	HLA-A*23:03	169	EYVSQPFLM	0,8499
A24	HLA-A*23:03	193	VFKNIDGYF	0,7818
A24	HLA-A*23:03	203	IYSKHTPINL	0,6028
A24	HLA-A*23:03	264	AYYVGYLQPRTF	0,7912
A24	HLA-A*23:03	265	YYVGYLQPRTF	0,879
A24	HLA-A*23:03	268	GYLQPRTFLL	0,7046
A24	HLA-A*23:03	269	YLQPRTFLL	0,5573
A24	HLA-A*23:03	312	IYQTSNFRV	0,6966
A24	HLA-A*23:03	328	RFPNITNLCPF	0,8158
A24	HLA-A*23:03	368	LYNSASFSTF	0,8553
A24	HLA-A*23:03	395	VYADSFVI	0,5149
A24	HLA-A*23:03	448	NYNYLYRLF	0,8881
A24	HLA-A*23:03	488	CYFPLQSYGF	0,6136
A24	HLA-A*23:03	489	YFPLQSYGF	0,9108
A24	HLA-A*23:03	504	GYQPYRVVVLSF	0,667
A24	HLA-A*23:03	507	PYRVVVLSF	0,8298
A24	HLA-A*23:03	558	KFLPFQQF	0,7881
A24	HLA-A*23:03	634	RVYSTGSNVF	0,5782
A24	HLA-A*23:03	635	VYSTGSNVF	0,934
A24	HLA-A*23:03	788	IYKTPPIKDF	0,9066
A24	HLA-A*23:03	816	SFIEDLLF	0,7909

A24	HLA-A*23:03	880	GTITSGWTF	0,5317
A24	HLA-A*23:03	898	FAMQMAYRF	0,5028
A24	HLA-A*23:03	1066	TYVPAQEKNF	0,9478
A24	HLA-A*23:03	1094	VFVSNGTHW	0,8761
A24	HLA-A*23:03	1094	VFVSNGTHWF	0,8258
A24	HLA-A*23:03	1101	HWFVTQRNF	0,7845
A24	HLA-A*23:03	1137	VYDPLQPELDSF	0,7515
A24	HLA-A*23:03	1208	QYIKWPWYI	0,9638
A24	HLA-A*23:03	1208	QYIKWPWYIW	0,7814
A24	HLA-A*23:03	1211	KWPWYIWLGF	0,6037
A24	HLA-A*23:03	1216	IWLGFIAGL	0,5925
A24	HLA-A*23:04	36	VYYPDKVF	0,9008
A24	HLA-A*23:04	37	YYPDKVFRSSVL	0,5679
A24	HLA-A*23:04	57	PFFSNVTWF	0,9013
A24	HLA-A*23:04	77	KRFDNPVLPF	0,6675
A24	HLA-A*23:04	78	RFDNPVLPF	0,9308
A24	HLA-A*23:04	143	VYYHKNNKSW	0,7934
A24	HLA-A*23:04	144	YYHKNNKSW	0,9179
A24	HLA-A*23:04	151	SWMESEFRV	0,667
A24	HLA-A*23:04	159	VYSSANNCTF	0,9321
A24	HLA-A*23:04	167	TFEYVSQPF	0,5826
A24	HLA-A*23:04	168	FEYVSQPFLM	0,5386
A24	HLA-A*23:04	169	EYVSQPFLM	0,8708
A24	HLA-A*23:04	193	VFKNIDGYF	0,8925
A24	HLA-A*23:04	203	IYSKHTPINL	0,679
A24	HLA-A*23:04	247	SYLTPGDSSSGW	0,6431
A24	HLA-A*23:04	264	AYYVGYLQPRTF	0,8651
A24	HLA-A*23:04	265	YYVGYLQPRTF	0,926
A24	HLA-A*23:04	265	YYVGYLQPRTFL	0,5396
A24	HLA-A*23:04	267	VGYLQPRTFL	0,6172
A24	HLA-A*23:04	268	GYLQPRTF	0,7597
A24	HLA-A*23:04	268	GYLQPRTFL	0,9029
A24	HLA-A*23:04	268	GYLQPRTFLL	0,8435
A24	HLA-A*23:04	269	YLQPRTFLL	0,5793
A24	HLA-A*23:04	312	IYQTSNFRV	0,807
A24	HLA-A*23:04	328	RFPNITNLCPF	0,8874
A24	HLA-A*23:04	345	TRFASVYAW	0,5445
A24	HLA-A*23:04	346	RFASVYAW	0,6882
A24	HLA-A*23:04	368	LYNSASFSTF	0,8917
A24	HLA-A*23:04	395	VYADSFVI	0,5709
A24	HLA-A*23:04	448	NYNYLYRLF	0,8977
A24	HLA-A*23:04	488	CYFPLQSYGF	0,7405
A24	HLA-A*23:04	489	YFPLQSYGF	0,9665
A24	HLA-A*23:04	504	GYQPYRVVV	0,6984
A24	HLA-A*23:04	504	GYQPYRVVVL	0,6433
A24	HLA-A*23:04	504	GYQPYRVVVLSF	0,8025
A24	HLA-A*23:04	507	PYRVVVLSF	0,8366
A24	HLA-A*23:04	558	KFLPFQQF	0,8975
A24	HLA-A*23:04	634	RVYSTGSNVF	0,7676
A24	HLA-A*23:04	635	VYSTGSNVF	0,9621

A24	HLA-A*23:04	659	SYECDIPIGAGI	0,5668
A24	HLA-A*23:04	706	AYSNNSIAI	0,7351
A24	HLA-A*23:04	755	QYGSFCTQL	0,6733
A24	HLA-A*23:04	788	IYKTPPIKDF	0,9258
A24	HLA-A*23:04	816	SFIEDLLF	0,8315
A24	HLA-A*23:04	880	GTITSGWTF	0,6365
A24	HLA-A*23:04	898	FAMQMAYRF	0,5384
A24	HLA-A*23:04	1051	SFPQSAPHGVVF	0,7156
A24	HLA-A*23:04	1066	TYVPAQEKNF	0,9578
A24	HLA-A*23:04	1094	VFVSNGTHW	0,9161
A24	HLA-A*23:04	1094	VFVSNGTHWF	0,829
A24	HLA-A*23:04	1101	HWFVTQRNF	0,9099
A24	HLA-A*23:04	1137	VYDPLQPEL	0,9435
A24	HLA-A*23:04	1137	VYDPLQPELDSF	0,802
A24	HLA-A*23:04	1147	SFKEELDKYF	0,5695
A24	HLA-A*23:04	1205	KYEQYIKW	0,612
A24	HLA-A*23:04	1208	QYIKWPWYI	0,9656
A24	HLA-A*23:04	1208	QYIKWPWYIW	0,7453
A24	HLA-A*23:04	1211	KWPWYIWLGF	0,7165
A24	HLA-A*23:04	1216	IWLGFIAGL	0,7942
A24	HLA-A*23:04	1219	GFIAGLIAI	0,5361
A24	HLA-A*23:06	57	PFFSNVTWF	0,9141
A24	HLA-A*23:06	78	RFDNPVLPF	0,8396
A24	HLA-A*23:06	143	VYYHKNNKSW	0,7142
A24	HLA-A*23:06	144	YYHKNNKSW	0,8036
A24	HLA-A*23:06	159	VYSSANNCTF	0,917
A24	HLA-A*23:06	169	EYVSQPFLM	0,8499
A24	HLA-A*23:06	193	VFKNIDGYF	0,7818
A24	HLA-A*23:06	203	IYSKHTPINL	0,6028
A24	HLA-A*23:06	264	AYYVGYLQPRTF	0,7912
A24	HLA-A*23:06	265	YYVGYLQPRTF	0,879
A24	HLA-A*23:06	268	GYLQPRTFLL	0,7046
A24	HLA-A*23:06	269	YLQPRTFLL	0,5573
A24	HLA-A*23:06	312	IYQTSNFRV	0,6966
A24	HLA-A*23:06	328	RFPNITNLCPF	0,8158
A24	HLA-A*23:06	368	LYNSASFSTF	0,8553
A24	HLA-A*23:06	395	VYADSFVI	0,5149
A24	HLA-A*23:06	448	NYNYLYRLF	0,8881
A24	HLA-A*23:06	488	CYFPLQSYGF	0,6136
A24	HLA-A*23:06	489	YFPLQSYGF	0,9108
A24	HLA-A*23:06	504	GYQPYRVVVLSF	0,667
A24	HLA-A*23:06	507	PYRVVVLSF	0,8298
A24	HLA-A*23:06	558	KFLPFQQF	0,7881
A24	HLA-A*23:06	634	RVYSTGSNVF	0,5782
A24	HLA-A*23:06	635	VYSTGSNVF	0,934
A24	HLA-A*23:06	788	IYKTPPIKDF	0,9066
A24	HLA-A*23:06	816	SFIEDLLF	0,7909
A24	HLA-A*23:06	880	GTITSGWTF	0,5317
A24	HLA-A*23:06	898	FAMQMAYRF	0,5028
A24	HLA-A*23:06	1066	TYVPAQEKNF	0,9478

A24	HLA-A*23:06	1094	VFVSNGTHW	0,8761
A24	HLA-A*23:06	1094	VFVSNGTHWF	0,8258
A24	HLA-A*23:06	1101	HWFVTQRNF	0,7845
A24	HLA-A*23:06	1137	VYDPLQPELDSF	0,7515
A24	HLA-A*23:06	1208	QYIKWPWYI	0,9638
A24	HLA-A*23:06	1208	QYIKWPWYIW	0,7814
A24	HLA-A*23:06	1211	KWPWYIWLGF	0,6037
A24	HLA-A*23:06	1216	IWLGFIAGL	0,5925
A24	HLA-A*23:10	57	PFFSNVTWF	0,8852
A24	HLA-A*23:10	78	RFDNPVLPF	0,8057
A24	HLA-A*23:10	159	VYSSANNCTF	0,8567
A24	HLA-A*23:10	169	EYVSQPFLM	0,8502
A24	HLA-A*23:10	264	AYYVGYLQPRTF	0,6408
A24	HLA-A*23:10	265	YYVGYLQPRTF	0,7982
A24	HLA-A*23:10	268	GYLQPRTFLL	0,6349
A24	HLA-A*23:10	269	YLQPRTFLL	0,5339
A24	HLA-A*23:10	312	IYQTSNFRV	0,5674
A24	HLA-A*23:10	328	RFPNITNLCPF	0,6995
A24	HLA-A*23:10	368	LYNSASFSTF	0,7785
A24	HLA-A*23:10	448	NYNYLYRLF	0,8162
A24	HLA-A*23:10	488	CYFPLQSYGF	0,5004
A24	HLA-A*23:10	489	YFPLQSYGF	0,8984
A24	HLA-A*23:10	504	GYQPYRVVVLSF	0,5485
A24	HLA-A*23:10	507	PYRVVVLSF	0,7181
A24	HLA-A*23:10	635	VYSTGSNVF	0,8708
A24	HLA-A*23:10	816	SFIEDLLF	0,7518
A24	HLA-A*23:10	880	GTITSGWTF	0,6006
A24	HLA-A*23:10	898	FAMQMAYRF	0,5102
A24	HLA-A*23:10	1066	TYVPAQEKNF	0,9105
A24	HLA-A*23:10	1094	VFVSNGTHW	0,8044
A24	HLA-A*23:10	1094	VFVSNGTHWF	0,7522
A24	HLA-A*23:10	1101	HWFVTQRNF	0,6918
A24	HLA-A*23:10	1137	VYDPLQPELDSF	0,682
A24	HLA-A*23:10	1208	QYIKWPWYI	0,9438
A24	HLA-A*23:10	1208	QYIKWPWYIW	0,6787
A24	HLA-A*23:10	1216	IWLGFIAGL	0,5657
A24	HLA-A*24:02	37	YYPDKVFRSSVL	0,601
A24	HLA-A*24:02	57	PFFSNVTWF	0,8728
A24	HLA-A*24:02	78	RFDNPVLPF	0,8538
A24	HLA-A*24:02	143	VYYHKNNKSW	0,7378
A24	HLA-A*24:02	144	YYHKNNKSW	0,8416
A24	HLA-A*24:02	151	SWMESEFRV	0,5046
A24	HLA-A*24:02	159	VYSSANNCTF	0,9582
A24	HLA-A*24:02	167	TFEYVSQPF	0,5365
A24	HLA-A*24:02	169	EYVSQPFLM	0,8484
A24	HLA-A*24:02	203	IYSKHTPINL	0,7085
A24	HLA-A*24:02	264	AYYVGYLQPRTF	0,8037
A24	HLA-A*24:02	265	YYVGYLQPRTF	0,9005
A24	HLA-A*24:02	268	GYLQPRTFLL	0,731
A24	HLA-A*24:02	269	YLQPRTFLL	0,5359

A24	HLA-A*24:02	312	IYQTSNFRV	0,785
A24	HLA-A*24:02	328	RFPNITNLCPF	0,8789
A24	HLA-A*24:02	346	RFASVYAW	0,521
A24	HLA-A*24:02	368	LYNSASFSTF	0,914
A24	HLA-A*24:02	395	VYADSFVI	0,6394
A24	HLA-A*24:02	448	NYNYLYRLF	0,9353
A24	HLA-A*24:02	488	CYFPLQSYGF	0,5885
A24	HLA-A*24:02	489	YFPLQSYGF	0,9442
A24	HLA-A*24:02	504	GYQPYRVVVLSF	0,6707
A24	HLA-A*24:02	507	PYRVVVLSF	0,8166
A24	HLA-A*24:02	558	KFLPFQQF	0,6948
A24	HLA-A*24:02	634	RVYSTGSNVF	0,7146
A24	HLA-A*24:02	635	VYSTGSNVF	0,9696
A24	HLA-A*24:02	755	QYGSFCTQL	0,5614
A24	HLA-A*24:02	788	IYKTPPIKDF	0,9226
A24	HLA-A*24:02	816	SFIEDLLF	0,7253
A24	HLA-A*24:02	1066	TYVPAQEKNF	0,9479
A24	HLA-A*24:02	1094	VFVSNGTHW	0,8658
A24	HLA-A*24:02	1094	VFVSNGTHWF	0,8335
A24	HLA-A*24:02	1101	HWFVTQRNF	0,754
A24	HLA-A*24:02	1137	VYDPLQPELDSF	0,8174
A24	HLA-A*24:02	1208	QYIKWPWYI	0,9613
A24	HLA-A*24:02	1208	QYIKWPWYIW	0,7582
A24	HLA-A*24:02	1211	KWPWYIWLGF	0,6582
A24	HLA-A*24:03	36	VYYPDKVF	0,8969
A24	HLA-A*24:03	37	YYPDKVFRSSVL	0,7149
A24	HLA-A*24:03	57	PFFSNVTWF	0,8822
A24	HLA-A*24:03	77	KRFDNPVLPF	0,6134
A24	HLA-A*24:03	78	RFDNPVLPF	0,9523
A24	HLA-A*24:03	143	VYYHKNNKSW	0,8351
A24	HLA-A*24:03	144	YYHKNNKSW	0,9523
A24	HLA-A*24:03	151	SWMESEFRV	0,7048
A24	HLA-A*24:03	159	VYSSANNCTF	0,9732
A24	HLA-A*24:03	167	TFEYVSQPF	0,6889
A24	HLA-A*24:03	169	EYVSQPFLM	0,8798
A24	HLA-A*24:03	193	VFKNIDGYF	0,8984
A24	HLA-A*24:03	203	IYSKHTPINL	0,8029
A24	HLA-A*24:03	247	SYLTPGDSSSGW	0,7197
A24	HLA-A*24:03	264	AYYVGYLQPRTF	0,8966
A24	HLA-A*24:03	265	YYVGYLQPRTF	0,9506
A24	HLA-A*24:03	265	YYVGYLQPRTFL	0,6242
A24	HLA-A*24:03	267	VGYLQPRTFL	0,7184
A24	HLA-A*24:03	268	GYLQPRTF	0,7874
A24	HLA-A*24:03	268	GYLQPRTFL	0,9337
A24	HLA-A*24:03	268	GYLQPRTFLL	0,8923
A24	HLA-A*24:03	312	IYQTSNFRV	0,8841
A24	HLA-A*24:03	328	RFPNITNLCPF	0,9389
A24	HLA-A*24:03	346	RFASVYAW	0,7335
A24	HLA-A*24:03	350	VYAWNRKRI	0,6686
A24	HLA-A*24:03	368	LYNSASFSTF	0,9453

A24	HLA-A*24:03	379	CYGVSPTKL	0,6202
A24	HLA-A*24:03	395	VYADSFVI	0,6956
A24	HLA-A*24:03	448	NYNYLYRLF	0,9379
A24	HLA-A*24:03	488	CYFPLQSYGF	0,7404
A24	HLA-A*24:03	489	YFPLQSYGF	0,9778
A24	HLA-A*24:03	504	GYQPYRVVV	0,7982
A24	HLA-A*24:03	504	GYQPYRVVVL	0,7444
A24	HLA-A*24:03	504	GYQPYRVVVLSF	0,8581
A24	HLA-A*24:03	507	PYRVVVLSF	0,8533
A24	HLA-A*24:03	558	KFLPFQQF	0,8636
A24	HLA-A*24:03	632	TWRVYSTGSNVF	0,5957
A24	HLA-A*24:03	634	RVYSTGSNVF	0,8822
A24	HLA-A*24:03	635	VYSTGSNVF	0,9872
A24	HLA-A*24:03	659	SYECDIPIGAGI	0,6187
A24	HLA-A*24:03	706	AYSNNSIAI	0,8828
A24	HLA-A*24:03	755	QYGSFCTQL	0,7929
A24	HLA-A*24:03	788	IYKTPPIKDF	0,9538
A24	HLA-A*24:03	816	SFIEDLLF	0,8244
A24	HLA-A*24:03	1051	SFPQSAPHGVVF	0,8397
A24	HLA-A*24:03	1066	TYVPAQEKNF	0,97
A24	HLA-A*24:03	1094	VFVSNGTHW	0,9343
A24	HLA-A*24:03	1094	VFVSNGTHWF	0,8723
A24	HLA-A*24:03	1101	HWFVTQRNF	0,9047
A24	HLA-A*24:03	1137	VYDPLQPEL	0,9496
A24	HLA-A*24:03	1137	VYDPLQPELDSF	0,8822
A24	HLA-A*24:03	1147	SFKEELDKYF	0,6438
A24	HLA-A*24:03	1205	KYEQYIKW	0,6328
A24	HLA-A*24:03	1208	QYIKWPWYI	0,9753
A24	HLA-A*24:03	1208	QYIKWPWYIW	0,7703
A24	HLA-A*24:03	1211	KWPWYIWLGF	0,7747
A24	HLA-A*24:03	1216	IWLGFIAGL	0,7773
A24	HLA-A*24:05	37	YYPDKVFRSSVL	0,601
A24	HLA-A*24:05	57	PFFSNVTWF	0,8728
A24	HLA-A*24:05	78	RFDNPVLPF	0,8538
A24	HLA-A*24:05	143	VYYHKNNKSW	0,7378
A24	HLA-A*24:05	144	YYHKNNKSW	0,8416
A24	HLA-A*24:05	151	SWMESEFRV	0,5046
A24	HLA-A*24:05	159	VYSSANNCTF	0,9582
A24	HLA-A*24:05	167	TFEYVSQPF	0,5365
A24	HLA-A*24:05	169	EYVSQPFLM	0,8484
A24	HLA-A*24:05	203	IYSKHTPINL	0,7085
A24	HLA-A*24:05	264	AYYVGYLQPRTF	0,8037
A24	HLA-A*24:05	265	YYVGYLQPRTF	0,9005
A24	HLA-A*24:05	268	GYLQPRTFLL	0,731
A24	HLA-A*24:05	269	YLQPRTFLL	0,5359
A24	HLA-A*24:05	312	IYQTSNFRV	0,785
A24	HLA-A*24:05	328	RFPNITNLCPF	0,8789
A24	HLA-A*24:05	346	RFASVYAW	0,521
A24	HLA-A*24:05	368	LYNSASFSTF	0,914
A24	HLA-A*24:05	395	VYADSFVI	0,6394

A24	HLA-A*24:05	448	NYNYLYRLF	0,9353
A24	HLA-A*24:05	488	CYFPLQSYGF	0,5885
A24	HLA-A*24:05	489	YFPLQSYGF	0,9442
A24	HLA-A*24:05	504	GYQPYRVVVLSF	0,6707
A24	HLA-A*24:05	507	PYRVVVLSF	0,8166
A24	HLA-A*24:05	558	KFLPFQQF	0,6948
A24	HLA-A*24:05	634	RVYSTGSNVF	0,7146
A24	HLA-A*24:05	635	VYSTGSNVF	0,9696
A24	HLA-A*24:05	755	QYGSFCTQL	0,5614
A24	HLA-A*24:05	788	IYKTPPIKDF	0,9226
A24	HLA-A*24:05	816	SFIEDLLF	0,7253
A24	HLA-A*24:05	1066	TYVPAQEKNF	0,9479
A24	HLA-A*24:05	1094	VFVSNGTHW	0,8658
A24	HLA-A*24:05	1094	VFVSNGTHWF	0,8335
A24	HLA-A*24:05	1101	HWFVTQRNF	0,754
A24	HLA-A*24:05	1137	VYDPLQPELDSF	0,8174
A24	HLA-A*24:05	1208	QYIKWPWYI	0,9613
A24	HLA-A*24:05	1208	QYIKWPWYIW	0,7582
A24	HLA-A*24:05	1211	KWPWYIWLGF	0,6582
A24	HLA-A*24:06	57	PFFSNVTWF	0,8811
A24	HLA-A*24:06	143	VYYHKNNKSW	0,7728
A24	HLA-A*24:06	144	YYHKNNKSW	0,8613
A24	HLA-A*24:06	159	VYSSANNCTF	0,9503
A24	HLA-A*24:06	169	EYVSQPFLM	0,8797
A24	HLA-A*24:06	193	VFKNIDGYF	0,8456
A24	HLA-A*24:06	203	IYSKHTPINL	0,6962
A24	HLA-A*24:06	264	AYYVGYLQPRTF	0,759
A24	HLA-A*24:06	265	YYVGYLQPRTF	0,9004
A24	HLA-A*24:06	268	GYLQPRTFLL	0,6697
A24	HLA-A*24:06	269	YLQPRTFLL	0,5368
A24	HLA-A*24:06	312	IYQTSNFRV	0,6415
A24	HLA-A*24:06	328	RFPNITNLCPF	0,8669
A24	HLA-A*24:06	368	LYNSASFSTF	0,901
A24	HLA-A*24:06	448	NYNYLYRLF	0,9202
A24	HLA-A*24:06	488	CYFPLQSYGF	0,6273
A24	HLA-A*24:06	489	YFPLQSYGF	0,9384
A24	HLA-A*24:06	504	GYQPYRVVVLSF	0,6274
A24	HLA-A*24:06	507	PYRVVVLSF	0,8272
A24	HLA-A*24:06	634	RVYSTGSNVF	0,7093
A24	HLA-A*24:06	635	VYSTGSNVF	0,9475
A24	HLA-A*24:06	755	QYGSFCTQL	0,5573
A24	HLA-A*24:06	788	IYKTPPIKDF	0,9187
A24	HLA-A*24:06	1066	TYVPAQEKNF	0,9671
A24	HLA-A*24:06	1094	VFVSNGTHW	0,8829
A24	HLA-A*24:06	1094	VFVSNGTHWF	0,8701
A24	HLA-A*24:06	1101	HWFVTQRNF	0,7966
A24	HLA-A*24:06	1208	QYIKWPWYI	0,9423
A24	HLA-A*24:06	1208	QYIKWPWYIW	0,7679
A24	HLA-A*24:06	1211	KWPWYIWLGF	0,5976
A24	HLA-A*24:08	144	YYHKNNKSW	0,7358

A24	HLA-A*24:08	159	VYSSANNCTF	0,8525
A24	HLA-A*24:08	265	YYVGYLQPRTF	0,757
A24	HLA-A*24:08	268	GYLQPRTFLL	0,6018
A24	HLA-A*24:08	269	YLQPRTFLL	0,6645
A24	HLA-A*24:08	312	IYQTSNFRV	0,6407
A24	HLA-A*24:08	328	RFPNITNLCPF	0,7387
A24	HLA-A*24:08	368	LYNSASFSTF	0,7867
A24	HLA-A*24:08	448	NYNYLYRLF	0,827
A24	HLA-A*24:08	489	YFPLQSYGF	0,8868
A24	HLA-A*24:08	635	VYSTGSNVF	0,8901
A24	HLA-A*24:08	1094	VFVSNGTHWF	0,7487
A24	HLA-A*24:08	1208	QYIKWPWYI	0,9046
A24	HLA-A*24:08	1208	QYIKWPWYIW	0,6181
A24	HLA-A*24:10	23	QLPPAYTNSF	0,6067
A24	HLA-A*24:10	36	VYYPDKVF	0,8127
A24	HLA-A*24:10	37	YYPDKVFRSSVL	0,7336
A24	HLA-A*24:10	57	PFFSNVTWF	0,8735
A24	HLA-A*24:10	77	KRFDNPVLPF	0,571
A24	HLA-A*24:10	78	RFDNPVLPF	0,9417
A24	HLA-A*24:10	143	VYYHKNNKSW	0,6813
A24	HLA-A*24:10	144	YYHKNNKSW	0,898
A24	HLA-A*24:10	151	SWMESEFRV	0,7017
A24	HLA-A*24:10	159	VYSSANNCTF	0,9516
A24	HLA-A*24:10	167	TFEYVSQPF	0,6744
A24	HLA-A*24:10	168	FEYVSQPFLM	0,5891
A24	HLA-A*24:10	169	EYVSQPFLM	0,8711
A24	HLA-A*24:10	193	VFKNIDGYF	0,8319
A24	HLA-A*24:10	203	IYSKHTPINL	0,7032
A24	HLA-A*24:10	247	SYLTPGDSSSGW	0,6388
A24	HLA-A*24:10	264	AYYVGYLQPRTF	0,8043
A24	HLA-A*24:10	265	YYVGYLQPRTF	0,8996
A24	HLA-A*24:10	265	YYVGYLQPRTFL	0,5933
A24	HLA-A*24:10	267	VGYLQPRTFL	0,6401
A24	HLA-A*24:10	268	GYLQPRTF	0,6374
A24	HLA-A*24:10	268	GYLQPRTFL	0,8962
A24	HLA-A*24:10	268	GYLQPRTFLL	0,8395
A24	HLA-A*24:10	269	YLQPRTFLL	0,5904
A24	HLA-A*24:10	312	IYQTSNFRV	0,8099
A24	HLA-A*24:10	328	RFPNITNLCPF	0,8972
A24	HLA-A*24:10	346	RFASVYAW	0,5964
A24	HLA-A*24:10	368	LYNSASFSTF	0,9153
A24	HLA-A*24:10	395	VYADSFVI	0,6432
A24	HLA-A*24:10	448	NYNYLYRLF	0,9031
A24	HLA-A*24:10	488	CYFPLQSYGF	0,6933
A24	HLA-A*24:10	489	YFPLQSYGF	0,9707
A24	HLA-A*24:10	504	GYQPYRVVV	0,6833
A24	HLA-A*24:10	504	GYQPYRVVVL	0,6625
A24	HLA-A*24:10	504	GYQPYRVVVLSF	0,7723
A24	HLA-A*24:10	507	PYRVVVLSF	0,7928
A24	HLA-A*24:10	558	KFLPFQQF	0,7572

A24	HLA-A*24:10	634	RVYSTGSNVF	0,7497
A24	HLA-A*24:10	635	VYSTGSNVF	0,9761
A24	HLA-A*24:10	659	SYECDIPIGAGI	0,5966
A24	HLA-A*24:10	706	AYSNNSIAI	0,8424
A24	HLA-A*24:10	755	QYGSFCTQL	0,7571
A24	HLA-A*24:10	788	IYKTPPIKDF	0,8924
A24	HLA-A*24:10	816	SFIEDLLF	0,8218
A24	HLA-A*24:10	880	GTITSGWTF	0,6067
A24	HLA-A*24:10	1051	SFPQSAPHGVVF	0,8219
A24	HLA-A*24:10	1066	TYVPAQEKNF	0,9499
A24	HLA-A*24:10	1094	VFVSNGTHW	0,8862
A24	HLA-A*24:10	1094	VFVSNGTHWF	0,8275
A24	HLA-A*24:10	1101	HWFVTQRNF	0,8486
A24	HLA-A*24:10	1137	VYDPLQPEL	0,9463
A24	HLA-A*24:10	1137	VYDPLQPELDSF	0,8552
A24	HLA-A*24:10	1147	SFKEELDKYF	0,621
A24	HLA-A*24:10	1208	QYIKWPWYI	0,9619
A24	HLA-A*24:10	1208	QYIKWPWYIW	0,6962
A24	HLA-A*24:10	1211	KWPWYIWLGF	0,6791
A24	HLA-A*24:10	1216	IWLGFIAGL	0,748
A24	HLA-A*24:13	57	PFFSNVTWF	0,9141
A24	HLA-A*24:13	78	RFDNPVLPF	0,8396
A24	HLA-A*24:13	143	VYYHKNNKSW	0,7142
A24	HLA-A*24:13	144	YYHKNNKSW	0,8036
A24	HLA-A*24:13	159	VYSSANNCTF	0,917
A24	HLA-A*24:13	169	EYVSQPFLM	0,8499
A24	HLA-A*24:13	193	VFKNIDGYF	0,7818
A24	HLA-A*24:13	203	IYSKHTPINL	0,6028
A24	HLA-A*24:13	264	AYYVGYLQPRTF	0,7912
A24	HLA-A*24:13	265	YYVGYLQPRTF	0,879
A24	HLA-A*24:13	268	GYLQPRTFLL	0,7046
A24	HLA-A*24:13	269	YLQPRTFLL	0,5573
A24	HLA-A*24:13	312	IYQTSNFRV	0,6966
A24	HLA-A*24:13	328	RFPNITNLCPF	0,8158
A24	HLA-A*24:13	368	LYNSASFSTF	0,8553
A24	HLA-A*24:13	395	VYADSFVI	0,5149
A24	HLA-A*24:13	448	NYNYLYRLF	0,8881
A24	HLA-A*24:13	488	CYFPLQSYGF	0,6136
A24	HLA-A*24:13	489	YFPLQSYGF	0,9108
A24	HLA-A*24:13	504	GYQPYRVVVLSF	0,667
A24	HLA-A*24:13	507	PYRVVVLSF	0,8298
A24	HLA-A*24:13	558	KFLPFQQF	0,7881
A24	HLA-A*24:13	634	RVYSTGSNVF	0,5782
A24	HLA-A*24:13	635	VYSTGSNVF	0,934
A24	HLA-A*24:13	788	IYKTPPIKDF	0,9066
A24	HLA-A*24:13	816	SFIEDLLF	0,7909
A24	HLA-A*24:13	880	GTITSGWTF	0,5317
A24	HLA-A*24:13	898	FAMQMAYRF	0,5028
A24	HLA-A*24:13	1066	TYVPAQEKNF	0,9478
A24	HLA-A*24:13	1094	VFVSNGTHW	0,8761

A24	HLA-A*24:13	1094	VFVSNGTHWF	0,8258
A24	HLA-A*24:13	1101	HWFVTQRNF	0,7845
A24	HLA-A*24:13	1137	VYDPLQPELDSF	0,7515
A24	HLA-A*24:13	1208	QYIKWPWYI	0,9638
A24	HLA-A*24:13	1208	QYIKWPWYIW	0,7814
A24	HLA-A*24:13	1211	KWPWYIWLGF	0,6037
A24	HLA-A*24:13	1216	IWLGFIAGL	0,5925
A24	HLA-A*24:18	36	VYYPDKVF	0,8173
A24	HLA-A*24:18	57	PFFSNVTWF	0,7076
A24	HLA-A*24:18	77	KRFDNPVLPF	0,5982
A24	HLA-A*24:18	78	RFDNPVLPF	0,8452
A24	HLA-A*24:18	143	VYYHKNNKSW	0,6438
A24	HLA-A*24:18	144	YYHKNNKSW	0,7986
A24	HLA-A*24:18	159	VYSSANNCTF	0,8212
A24	HLA-A*24:18	169	EYVSQPFLM	0,6396
A24	HLA-A*24:18	193	VFKNIDGYF	0,8479
A24	HLA-A*24:18	203	IYSKHTPINL	0,5908
A24	HLA-A*24:18	264	AYYVGYLQPRTF	0,7906
A24	HLA-A*24:18	265	YYVGYLQPRTF	0,7951
A24	HLA-A*24:18	267	VGYLQPRTFL	0,5749
A24	HLA-A*24:18	268	GYLQPRTF	0,7049
A24	HLA-A*24:18	268	GYLQPRTFL	0,8826
A24	HLA-A*24:18	268	GYLQPRTFLL	0,7029
A24	HLA-A*24:18	312	IYQTSNFRV	0,625
A24	HLA-A*24:18	328	RFPNITNLCPF	0,6955
A24	HLA-A*24:18	350	VYAWNRKRI	0,5642
A24	HLA-A*24:18	368	LYNSASFSTF	0,7395
A24	HLA-A*24:18	448	NYNYLYRLF	0,8086
A24	HLA-A*24:18	488	CYFPLQSYGF	0,5236
A24	HLA-A*24:18	489	YFPLQSYGF	0,8335
A24	HLA-A*24:18	504	GYQPYRVVV	0,659
A24	HLA-A*24:18	504	GYQPYRVVVL	0,5063
A24	HLA-A*24:18	504	GYQPYRVVVLSF	0,6043
A24	HLA-A*24:18	507	PYRVVVLSF	0,7472
A24	HLA-A*24:18	558	KFLPFQQF	0,7919
A24	HLA-A*24:18	634	RVYSTGSNVF	0,6968
A24	HLA-A*24:18	635	VYSTGSNVF	0,9155
A24	HLA-A*24:18	706	AYSNNSIAI	0,6343
A24	HLA-A*24:18	755	QYGSFCTQL	0,5402
A24	HLA-A*24:18	788	IYKTPPIKDF	0,8671
A24	HLA-A*24:18	1066	TYVPAQEKNF	0,7987
A24	HLA-A*24:18	1094	VFVSNGTHW	0,7787
A24	HLA-A*24:18	1094	VFVSNGTHWF	0,6654
A24	HLA-A*24:18	1101	HWFVTQRNF	0,8544
A24	HLA-A*24:18	1137	VYDPLQPEL	0,8216
A24	HLA-A*24:18	1208	QYIKWPWYI	0,8782
A24	HLA-A*24:18	1211	KWPWYIWLGF	0,5123
A24	HLA-A*24:18	1216	IWLGFIAGL	0,7231
A24	HLA-A*24:20	37	YYPDKVFRSSVL	0,601
A24	HLA-A*24:20	57	PFFSNVTWF	0,8728

A24	HLA-A*24:20	78	RFDNPVLPF	0,8538
A24	HLA-A*24:20	143	VYYHKNNKSW	0,7378
A24	HLA-A*24:20	144	YYHKNNKSW	0,8416
A24	HLA-A*24:20	151	SWMESEFRV	0,5046
A24	HLA-A*24:20	159	VYSSANNCTF	0,9582
A24	HLA-A*24:20	167	TFEYVSQPF	0,5365
A24	HLA-A*24:20	169	EYVSQPFLM	0,8484
A24	HLA-A*24:20	203	IYSKHTPINL	0,7085
A24	HLA-A*24:20	264	AYYVGYLQPRTF	0,8037
A24	HLA-A*24:20	265	YYVGYLQPRTF	0,9005
A24	HLA-A*24:20	268	GYLQPRTFLL	0,731
A24	HLA-A*24:20	269	YLQPRTFLL	0,5359
A24	HLA-A*24:20	312	IYQTSNFRV	0,785
A24	HLA-A*24:20	328	RFPNITNLCPF	0,8789
A24	HLA-A*24:20	346	RFASVYAW	0,521
A24	HLA-A*24:20	368	LYNSASFSTF	0,914
A24	HLA-A*24:20	395	VYADSFVI	0,6394
A24	HLA-A*24:20	448	NYNYLYRLF	0,9353
A24	HLA-A*24:20	488	CYFPLQSYGF	0,5885
A24	HLA-A*24:20	489	YFPLQSYGF	0,9442
A24	HLA-A*24:20	504	GYQPYRVVVLSF	0,6707
A24	HLA-A*24:20	507	PYRVVVLSF	0,8166
A24	HLA-A*24:20	558	KFLPFQQF	0,6948
A24	HLA-A*24:20	634	RVYSTGSNVF	0,7146
A24	HLA-A*24:20	635	VYSTGSNVF	0,9696
A24	HLA-A*24:20	755	QYGSFCTQL	0,5614
A24	HLA-A*24:20	788	IYKTPPIKDF	0,9226
A24	HLA-A*24:20	816	SFIEDLLF	0,7253
A24	HLA-A*24:20	1066	TYVPAQEKNF	0,9479
A24	HLA-A*24:20	1094	VFVSNGTHW	0,8658
A24	HLA-A*24:20	1094	VFVSNGTHWF	0,8335
A24	HLA-A*24:20	1101	HWFVTQRNF	0,754
A24	HLA-A*24:20	1137	VYDPLQPELDSF	0,8174
A24	HLA-A*24:20	1208	QYIKWPWYI	0,9613
A24	HLA-A*24:20	1208	QYIKWPWYIW	0,7582
A24	HLA-A*24:20	1211	KWPWYIWLGF	0,6582
A24	HLA-A*24:21	37	YYPDKVFRSSVL	0,601
A24	HLA-A*24:21	57	PFFSNVTWF	0,8728
A24	HLA-A*24:21	78	RFDNPVLPF	0,8538
A24	HLA-A*24:21	143	VYYHKNNKSW	0,7378
A24	HLA-A*24:21	144	YYHKNNKSW	0,8416
A24	HLA-A*24:21	151	SWMESEFRV	0,5046
A24	HLA-A*24:21	159	VYSSANNCTF	0,9582
A24	HLA-A*24:21	167	TFEYVSQPF	0,5365
A24	HLA-A*24:21	169	EYVSQPFLM	0,8484
A24	HLA-A*24:21	203	IYSKHTPINL	0,7085
A24	HLA-A*24:21	264	AYYVGYLQPRTF	0,8037
A24	HLA-A*24:21	265	YYVGYLQPRTF	0,9005
A24	HLA-A*24:21	268	GYLQPRTFLL	0,731
A24	HLA-A*24:21	269	YLQPRTFLL	0,5359

A24	HLA-A*24:21	312	IYQTSNFRV	0,785
A24	HLA-A*24:21	328	RFPNITNLCPF	0,8789
A24	HLA-A*24:21	346	RFASVYAW	0,521
A24	HLA-A*24:21	368	LYNSASFSTF	0,914
A24	HLA-A*24:21	395	VYADSFVI	0,6394
A24	HLA-A*24:21	448	NYNYLYRLF	0,9353
A24	HLA-A*24:21	488	CYFPLQSYGF	0,5885
A24	HLA-A*24:21	489	YFPLQSYGF	0,9442
A24	HLA-A*24:21	504	GYQPYRVVVLSF	0,6707
A24	HLA-A*24:21	507	PYRVVVLSF	0,8166
A24	HLA-A*24:21	558	KFLPFQQF	0,6948
A24	HLA-A*24:21	634	RVYSTGSNVF	0,7146
A24	HLA-A*24:21	635	VYSTGSNVF	0,9696
A24	HLA-A*24:21	755	QYGSFCTQL	0,5614
A24	HLA-A*24:21	788	IYKTPPIKDF	0,9226
A24	HLA-A*24:21	816	SFIEDLLF	0,7253
A24	HLA-A*24:21	1066	TYVPAQEKNF	0,9479
A24	HLA-A*24:21	1094	VFVSNGTHW	0,8658
A24	HLA-A*24:21	1094	VFVSNGTHWF	0,8335
A24	HLA-A*24:21	1101	HWFVTQRNF	0,754
A24	HLA-A*24:21	1137	VYDPLQPELDSF	0,8174
A24	HLA-A*24:21	1208	QYIKWPWYI	0,9613
A24	HLA-A*24:21	1208	QYIKWPWYIW	0,7582
A24	HLA-A*24:21	1211	KWPWYIWLGF	0,6582
A24	HLA-A*24:22	23	QLPPAYTNSF	0,6934
A24	HLA-A*24:22	36	VYYPDKVF	0,8478
A24	HLA-A*24:22	37	YYPDKVFRSSVL	0,692
A24	HLA-A*24:22	47	VLHSTQDLF	0,6614
A24	HLA-A*24:22	57	PFFSNVTWF	0,8749
A24	HLA-A*24:22	77	KRFDNPVLPF	0,6655
A24	HLA-A*24:22	78	RFDNPVLPF	0,861
A24	HLA-A*24:22	143	VYYHKNNKSW	0,8066
A24	HLA-A*24:22	144	YYHKNNKSW	0,9429
A24	HLA-A*24:22	151	SWMESEFRV	0,6473
A24	HLA-A*24:22	159	VYSSANNCTF	0,9584
A24	HLA-A*24:22	167	TFEYVSQPF	0,6382
A24	HLA-A*24:22	169	EYVSQPFLM	0,8978
A24	HLA-A*24:22	193	VFKNIDGYF	0,9224
A24	HLA-A*24:22	203	IYSKHTPINL	0,7532
A24	HLA-A*24:22	247	SYLTPGDSSSGW	0,7072
A24	HLA-A*24:22	264	AYYVGYLQPRTF	0,8149
A24	HLA-A*24:22	265	YYVGYLQPRTF	0,9218
A24	HLA-A*24:22	268	GYLQPRTFL	0,8871
A24	HLA-A*24:22	268	GYLQPRTFLL	0,7943
A24	HLA-A*24:22	269	YLQPRTFLL	0,6428
A24	HLA-A*24:22	312	IYQTSNFRV	0,7597
A24	HLA-A*24:22	328	RFPNITNLCPF	0,8917
A24	HLA-A*24:22	346	RFASVYAW	0,6744
A24	HLA-A*24:22	350	VYAWNRKRI	0,6447
A24	HLA-A*24:22	368	LYNSASFSTF	0,9108

A24	HLA-A*24:22	369	YNSASFSTF	0,6618
A24	HLA-A*24:22	379	CYGVSPTKL	0,6159
A24	HLA-A*24:22	448	NYNYLYRLF	0,9199
A24	HLA-A*24:22	488	CYFPLQSYGF	0,766
A24	HLA-A*24:22	489	YFPLQSYGF	0,9702
A24	HLA-A*24:22	504	GYQPYRVVV	0,6915
A24	HLA-A*24:22	504	GYQPYRVVVL	0,6647
A24	HLA-A*24:22	504	GYQPYRVVVLSF	0,7575
A24	HLA-A*24:22	507	PYRVVVLSF	0,8211
A24	HLA-A*24:22	558	KFLPFQQF	0,7981
A24	HLA-A*24:22	634	RVYSTGSNVF	0,8412
A24	HLA-A*24:22	635	VYSTGSNVF	0,9766
A24	HLA-A*24:22	659	SYECDIPIGAGI	0,6229
A24	HLA-A*24:22	706	AYSNNSIAI	0,7925
A24	HLA-A*24:22	755	QYGSFCTQL	0,7423
A24	HLA-A*24:22	788	IYKTPPIKDF	0,9284
A24	HLA-A*24:22	816	SFIEDLLF	0,7943
A24	HLA-A*24:22	880	GTITSGWTF	0,6649
A24	HLA-A*24:22	1051	SFPQSAPHGVVF	0,8172
A24	HLA-A*24:22	1066	TYVPAQEKNF	0,9704
A24	HLA-A*24:22	1094	VFVSNGTHW	0,9217
A24	HLA-A*24:22	1094	VFVSNGTHWF	0,8558
A24	HLA-A*24:22	1095	FVSNGTHWF	0,6853
A24	HLA-A*24:22	1101	HWFVTQRNF	0,884
A24	HLA-A*24:22	1137	VYDPLQPEL	0,9126
A24	HLA-A*24:22	1137	VYDPLQPELDSF	0,8166
A24	HLA-A*24:22	1147	SFKEELDKYF	0,7313
A24	HLA-A*24:22	1208	QYIKWPWYI	0,9408
A24	HLA-A*24:22	1208	QYIKWPWYIW	0,7516
A24	HLA-A*24:22	1211	KWPWYIWLGF	0,6645
A24	HLA-A*24:22	1216	IWLGFIAGL	0,7116
A24	HLA-A*24:23	36	VYYPDKVF	0,8969
A24	HLA-A*24:23	37	YYPDKVFRSSVL	0,7149
A24	HLA-A*24:23	57	PFFSNVTWF	0,8822
A24	HLA-A*24:23	77	KRFDNPVLPF	0,6134
A24	HLA-A*24:23	78	RFDNPVLPF	0,9523
A24	HLA-A*24:23	143	VYYHKNNKSW	0,8351
A24	HLA-A*24:23	144	YYHKNNKSW	0,9523
A24	HLA-A*24:23	151	SWMESEFRV	0,7048
A24	HLA-A*24:23	159	VYSSANNCTF	0,9732
A24	HLA-A*24:23	167	TFEYVSQPF	0,6889
A24	HLA-A*24:23	169	EYVSQPFLM	0,8798
A24	HLA-A*24:23	193	VFKNIDGYF	0,8984
A24	HLA-A*24:23	203	IYSKHTPINL	0,8029
A24	HLA-A*24:23	247	SYLTPGDSSSGW	0,7197
A24	HLA-A*24:23	264	AYYVGYLQPRTF	0,8966
A24	HLA-A*24:23	265	YYVGYLQPRTF	0,9506
A24	HLA-A*24:23	265	YYVGYLQPRTFL	0,6242
A24	HLA-A*24:23	267	VGYLQPRTFL	0,7184
A24	HLA-A*24:23	268	GYLQPRTF	0,7874

A24	HLA-A*24:23	268	GYLQPRTFL	0,9337
A24	HLA-A*24:23	268	GYLQPRTFLL	0,8923
A24	HLA-A*24:23	312	IYQTSNFRV	0,8841
A24	HLA-A*24:23	328	RFPNITNLCPF	0,9389
A24	HLA-A*24:23	346	RFASVYAW	0,7335
A24	HLA-A*24:23	350	VYAWNRKRI	0,6686
A24	HLA-A*24:23	368	LYNSASFSTF	0,9453
A24	HLA-A*24:23	379	CYGVSPTKL	0,6202
A24	HLA-A*24:23	395	VYADSFVI	0,6956
A24	HLA-A*24:23	448	NYNYLYRLF	0,9379
A24	HLA-A*24:23	488	CYFPLQSYGF	0,7404
A24	HLA-A*24:23	489	YFPLQSYGF	0,9778
A24	HLA-A*24:23	504	GYQPYRVVV	0,7982
A24	HLA-A*24:23	504	GYQPYRVVVL	0,7444
A24	HLA-A*24:23	504	GYQPYRVVVLSF	0,8581
A24	HLA-A*24:23	507	PYRVVVLSF	0,8533
A24	HLA-A*24:23	558	KFLPFQQF	0,8636
A24	HLA-A*24:23	632	TWRVYSTGSNVF	0,5957
A24	HLA-A*24:23	634	RVYSTGSNVF	0,8822
A24	HLA-A*24:23	635	VYSTGSNVF	0,9872
A24	HLA-A*24:23	659	SYECDIPIGAGI	0,6187
A24	HLA-A*24:23	706	AYSNNSIAI	0,8828
A24	HLA-A*24:23	755	QYGSFCTQL	0,7929
A24	HLA-A*24:23	788	IYKTPPIKDF	0,9538
A24	HLA-A*24:23	816	SFIEDLLF	0,8244
A24	HLA-A*24:23	1051	SFPQSAPHGVVF	0,8397
A24	HLA-A*24:23	1066	TYVPAQEKNF	0,97
A24	HLA-A*24:23	1094	VFVSNGTHW	0,9343
A24	HLA-A*24:23	1094	VFVSNGTHWF	0,8723
A24	HLA-A*24:23	1101	HWFVTQRNF	0,9047
A24	HLA-A*24:23	1137	VYDPLQPEL	0,9496
A24	HLA-A*24:23	1137	VYDPLQPELDSF	0,8822
A24	HLA-A*24:23	1147	SFKEELDKYF	0,6438
A24	HLA-A*24:23	1205	KYEQYIKW	0,6328
A24	HLA-A*24:23	1208	QYIKWPWYI	0,9753
A24	HLA-A*24:23	1208	QYIKWPWYIW	0,7703
A24	HLA-A*24:23	1211	KWPWYIWLGF	0,7747
A24	HLA-A*24:23	1216	IWLGFIAGL	0,7773
A24	HLA-A*24:26	37	YYPDKVFRSSVL	0,601
A24	HLA-A*24:26	57	PFFSNVTWF	0,8728
A24	HLA-A*24:26	78	RFDNPVLPF	0,8538
A24	HLA-A*24:26	143	VYYHKNNKSW	0,7378
A24	HLA-A*24:26	144	YYHKNNKSW	0,8416
A24	HLA-A*24:26	151	SWMESEFRV	0,5046
A24	HLA-A*24:26	159	VYSSANNCTF	0,9582
A24	HLA-A*24:26	167	TFEYVSQPF	0,5365
A24	HLA-A*24:26	169	EYVSQPFLM	0,8484
A24	HLA-A*24:26	203	IYSKHTPINL	0,7085
A24	HLA-A*24:26	264	AYYVGYLQPRTF	0,8037
A24	HLA-A*24:26	265	YYVGYLQPRTF	0,9005

A24	HLA-A*24:26	268	GYLQPRTFLL	0,731
A24	HLA-A*24:26	269	YLQPRTFLL	0,5359
A24	HLA-A*24:26	312	IYQTSNFRV	0,785
A24	HLA-A*24:26	328	RFPNITNLCPF	0,8789
A24	HLA-A*24:26	346	RFASVYAW	0,521
A24	HLA-A*24:26	368	LYNSASFSTF	0,914
A24	HLA-A*24:26	395	VYADSFVI	0,6394
A24	HLA-A*24:26	448	NYNYLYRLF	0,9353
A24	HLA-A*24:26	488	CYFPLQSYGF	0,5885
A24	HLA-A*24:26	489	YFPLQSYGF	0,9442
A24	HLA-A*24:26	504	GYQPYRVVVLSF	0,6707
A24	HLA-A*24:26	507	PYRVVVLSF	0,8166
A24	HLA-A*24:26	558	KFLPFQQF	0,6948
A24	HLA-A*24:26	634	RVYSTGSNVF	0,7146
A24	HLA-A*24:26	635	VYSTGSNVF	0,9696
A24	HLA-A*24:26	755	QYGSFCTQL	0,5614
A24	HLA-A*24:26	788	IYKTPPIKDF	0,9226
A24	HLA-A*24:26	816	SFIEDLLF	0,7253
A24	HLA-A*24:26	1066	TYVPAQEKNF	0,9479
A24	HLA-A*24:26	1094	VFVSNGTHW	0,8658
A24	HLA-A*24:26	1094	VFVSNGTHWF	0,8335
A24	HLA-A*24:26	1101	HWFVTQRNF	0,754
A24	HLA-A*24:26	1137	VYDPLQPELDSF	0,8174
A24	HLA-A*24:26	1208	QYIKWPWYI	0,9613
A24	HLA-A*24:26	1208	QYIKWPWYIW	0,7582
A24	HLA-A*24:26	1211	KWPWYIWLGF	0,6582
A24	HLA-A*24:27	37	YYPDKVFRSSVL	0,601
A24	HLA-A*24:27	57	PFFSNVTWF	0,8728
A24	HLA-A*24:27	78	RFDNPVLPF	0,8538
A24	HLA-A*24:27	143	VYYHKNNKSW	0,7378
A24	HLA-A*24:27	144	YYHKNNKSW	0,8416
A24	HLA-A*24:27	151	SWMESEFRV	0,5046
A24	HLA-A*24:27	159	VYSSANNCTF	0,9582
A24	HLA-A*24:27	167	TFEYVSQPF	0,5365
A24	HLA-A*24:27	169	EYVSQPFLM	0,8484
A24	HLA-A*24:27	203	IYSKHTPINL	0,7085
A24	HLA-A*24:27	264	AYYVGYLQPRTF	0,8037
A24	HLA-A*24:27	265	YYVGYLQPRTF	0,9005
A24	HLA-A*24:27	268	GYLQPRTFLL	0,731
A24	HLA-A*24:27	269	YLQPRTFLL	0,5359
A24	HLA-A*24:27	312	IYQTSNFRV	0,785
A24	HLA-A*24:27	328	RFPNITNLCPF	0,8789
A24	HLA-A*24:27	346	RFASVYAW	0,521
A24	HLA-A*24:27	368	LYNSASFSTF	0,914
A24	HLA-A*24:27	395	VYADSFVI	0,6394
A24	HLA-A*24:27	448	NYNYLYRLF	0,9353
A24	HLA-A*24:27	488	CYFPLQSYGF	0,5885
A24	HLA-A*24:27	489	YFPLQSYGF	0,9442
A24	HLA-A*24:27	504	GYQPYRVVVLSF	0,6707
A24	HLA-A*24:27	507	PYRVVVLSF	0,8166

A24	HLA-A*24:27	558	KFLPFQQF	0,6948
A24	HLA-A*24:27	634	RVYSTGSNVF	0,7146
A24	HLA-A*24:27	635	VYSTGSNVF	0,9696
A24	HLA-A*24:27	755	QYGSFCTQL	0,5614
A24	HLA-A*24:27	788	IYKTPPIKDF	0,9226
A24	HLA-A*24:27	816	SFIEDLLF	0,7253
A24	HLA-A*24:27	1066	TYVPAQEKNF	0,9479
A24	HLA-A*24:27	1094	VFVSNGTHW	0,8658
A24	HLA-A*24:27	1094	VFVSNGTHWF	0,8335
A24	HLA-A*24:27	1101	HWFVTQRNF	0,754
A24	HLA-A*24:27	1137	VYDPLQPELDSF	0,8174
A24	HLA-A*24:27	1208	QYIKWPWYI	0,9613
A24	HLA-A*24:27	1208	QYIKWPWYIW	0,7582
A24	HLA-A*24:27	1211	KWPWYIWLGF	0,6582
A24	HLA-A*24:28	151	SWMESEFRV	0,6568
A24	HLA-A*24:28	269	YLQPRTFLL	0,5702
A24	HLA-A*24:28	312	IYQTSNFRV	0,5721
A24	HLA-A*24:28	1208	QYIKWPWYI	0,7279
A24	HLA-A*24:28	1216	IWLGFIAGL	0,697
A24	HLA-A*24:29	37	YYPDKVFRSSVL	0,601
A24	HLA-A*24:29	57	PFFSNVTWF	0,8728
A24	HLA-A*24:29	78	RFDNPVLPF	0,8538
A24	HLA-A*24:29	143	VYYHKNNKSW	0,7378
A24	HLA-A*24:29	144	YYHKNNKSW	0,8416
A24	HLA-A*24:29	151	SWMESEFRV	0,5046
A24	HLA-A*24:29	159	VYSSANNCTF	0,9582
A24	HLA-A*24:29	167	TFEYVSQPF	0,5365
A24	HLA-A*24:29	169	EYVSQPFLM	0,8484
A24	HLA-A*24:29	203	IYSKHTPINL	0,7085
A24	HLA-A*24:29	264	AYYVGYLQPRTF	0,8037
A24	HLA-A*24:29	265	YYVGYLQPRTF	0,9005
A24	HLA-A*24:29	268	GYLQPRTFLL	0,731
A24	HLA-A*24:29	269	YLQPRTFLL	0,5359
A24	HLA-A*24:29	312	IYQTSNFRV	0,785
A24	HLA-A*24:29	328	RFPNITNLCPF	0,8789
A24	HLA-A*24:29	346	RFASVYAW	0,521
A24	HLA-A*24:29	368	LYNSASFSTF	0,914
A24	HLA-A*24:29	395	VYADSFVI	0,6394
A24	HLA-A*24:29	448	NYNYLYRLF	0,9353
A24	HLA-A*24:29	488	CYFPLQSYGF	0,5885
A24	HLA-A*24:29	489	YFPLQSYGF	0,9442
A24	HLA-A*24:29	504	GYQPYRVVVLSF	0,6707
A24	HLA-A*24:29	507	PYRVVVLSF	0,8166
A24	HLA-A*24:29	558	KFLPFQQF	0,6948
A24	HLA-A*24:29	634	RVYSTGSNVF	0,7146
A24	HLA-A*24:29	635	VYSTGSNVF	0,9696
A24	HLA-A*24:29	755	QYGSFCTQL	0,5614
A24	HLA-A*24:29	788	IYKTPPIKDF	0,9226
A24	HLA-A*24:29	816	SFIEDLLF	0,7253
A24	HLA-A*24:29	1066	TYVPAQEKNF	0,9479

A24	HLA-A*24:29	1094	VFVSNGTHW	0,8658
A24	HLA-A*24:29	1094	VFVSNGTHWF	0,8335
A24	HLA-A*24:29	1101	HWFVTQRNF	0,754
A24	HLA-A*24:29	1137	VYDPLQPELDSF	0,8174
A24	HLA-A*24:29	1208	QYIKWPWYI	0,9613
A24	HLA-A*24:29	1208	QYIKWPWYIW	0,7582
A24	HLA-A*24:29	1211	KWPWYIWLGF	0,6582
A24	HLA-A*24:33	36	VYYPDKVF	0,8969
A24	HLA-A*24:33	37	YYPDKVFRSSVL	0,7149
A24	HLA-A*24:33	57	PFFSNVTWF	0,8822
A24	HLA-A*24:33	77	KRFDNPVLPF	0,6134
A24	HLA-A*24:33	78	RFDNPVLPF	0,9523
A24	HLA-A*24:33	143	VYYHKNNKSW	0,8351
A24	HLA-A*24:33	144	YYHKNNKSW	0,9523
A24	HLA-A*24:33	151	SWMESEFRV	0,7048
A24	HLA-A*24:33	159	VYSSANNCTF	0,9732
A24	HLA-A*24:33	167	TFEYVSQPF	0,6889
A24	HLA-A*24:33	169	EYVSQPFLM	0,8798
A24	HLA-A*24:33	193	VFKNIDGYF	0,8984
A24	HLA-A*24:33	203	IYSKHTPINL	0,8029
A24	HLA-A*24:33	247	SYLTPGDSSSGW	0,7197
A24	HLA-A*24:33	264	AYYVGYLQPRTF	0,8966
A24	HLA-A*24:33	265	YYVGYLQPRTF	0,9506
A24	HLA-A*24:33	265	YYVGYLQPRTFL	0,6242
A24	HLA-A*24:33	267	VGYLQPRTFL	0,7184
A24	HLA-A*24:33	268	GYLQPRTF	0,7874
A24	HLA-A*24:33	268	GYLQPRTFL	0,9337
A24	HLA-A*24:33	268	GYLQPRTFLL	0,8923
A24	HLA-A*24:33	312	IYQTSNFRV	0,8841
A24	HLA-A*24:33	328	RFPNITNLCPF	0,9389
A24	HLA-A*24:33	346	RFASVYAW	0,7335
A24	HLA-A*24:33	350	VYAWNRKRI	0,6686
A24	HLA-A*24:33	368	LYNSASFSTF	0,9453
A24	HLA-A*24:33	379	CYGVSPTKL	0,6202
A24	HLA-A*24:33	395	VYADSFVI	0,6956
A24	HLA-A*24:33	448	NYNYLYRLF	0,9379
A24	HLA-A*24:33	488	CYFPLQSYGF	0,7404
A24	HLA-A*24:33	489	YFPLQSYGF	0,9778
A24	HLA-A*24:33	504	GYQPYRVVV	0,7982
A24	HLA-A*24:33	504	GYQPYRVVVL	0,7444
A24	HLA-A*24:33	504	GYQPYRVVVLSF	0,8581
A24	HLA-A*24:33	507	PYRVVVLSF	0,8533
A24	HLA-A*24:33	558	KFLPFQQF	0,8636
A24	HLA-A*24:33	632	TWRVYSTGSNVF	0,5957
A24	HLA-A*24:33	634	RVYSTGSNVF	0,8822
A24	HLA-A*24:33	635	VYSTGSNVF	0,9872
A24	HLA-A*24:33	659	SYECDIPIGAGI	0,6187
A24	HLA-A*24:33	706	AYSNNSIAI	0,8828
A24	HLA-A*24:33	755	QYGSFCTQL	0,7929
A24	HLA-A*24:33	788	IYKTPPIKDF	0,9538

A24	HLA-A*24:33	816	SFIEDLLF	0,8244
A24	HLA-A*24:33	1051	SFPQSAPHGVVF	0,8397
A24	HLA-A*24:33	1066	TYVPAQEKNF	0,97
A24	HLA-A*24:33	1094	VFVSNGTHW	0,9343
A24	HLA-A*24:33	1094	VFVSNGTHWF	0,8723
A24	HLA-A*24:33	1101	HWFVTQRNF	0,9047
A24	HLA-A*24:33	1137	VYDPLQPEL	0,9496
A24	HLA-A*24:33	1137	VYDPLQPELDSF	0,8822
A24	HLA-A*24:33	1147	SFKEELDKYF	0,6438
A24	HLA-A*24:33	1205	KYEQYIKW	0,6328
A24	HLA-A*24:33	1208	QYIKWPWYI	0,9753
A24	HLA-A*24:33	1208	QYIKWPWYIW	0,7703
A24	HLA-A*24:33	1211	KWPWYIWLGF	0,7747
A24	HLA-A*24:33	1216	IWLGFIAGL	0,7773
A24	HLA-A*24:34	37	YYPDKVFRSSVL	0,6196
A24	HLA-A*24:34	57	PFFSNVTWF	0,8335
A24	HLA-A*24:34	78	RFDNPVLPF	0,8388
A24	HLA-A*24:34	143	VYYHKNNKSW	0,6844
A24	HLA-A*24:34	144	YYHKNNKSW	0,819
A24	HLA-A*24:34	151	SWMESEFRV	0,5644
A24	HLA-A*24:34	159	VYSSANNCTF	0,9424
A24	HLA-A*24:34	167	TFEYVSQPF	0,5518
A24	HLA-A*24:34	169	EYVSQPFLM	0,8551
A24	HLA-A*24:34	193	VFKNIDGYF	0,7905
A24	HLA-A*24:34	203	IYSKHTPINL	0,7288
A24	HLA-A*24:34	264	AYYVGYLQPRTF	0,7698
A24	HLA-A*24:34	265	YYVGYLQPRTF	0,857
A24	HLA-A*24:34	268	GYLQPRTFLL	0,7636
A24	HLA-A*24:34	269	YLQPRTFLL	0,6367
A24	HLA-A*24:34	312	IYQTSNFRV	0,7925
A24	HLA-A*24:34	328	RFPNITNLCPF	0,8538
A24	HLA-A*24:34	350	VYAWNRKRI	0,5795
A24	HLA-A*24:34	368	LYNSASFSTF	0,9096
A24	HLA-A*24:34	395	VYADSFVI	0,6072
A24	HLA-A*24:34	448	NYNYLYRLF	0,9345
A24	HLA-A*24:34	488	CYFPLQSYGF	0,618
A24	HLA-A*24:34	489	YFPLQSYGF	0,9263
A24	HLA-A*24:34	504	GYQPYRVVVLSF	0,6666
A24	HLA-A*24:34	507	PYRVVVLSF	0,7841
A24	HLA-A*24:34	634	RVYSTGSNVF	0,6878
A24	HLA-A*24:34	635	VYSTGSNVF	0,9648
A24	HLA-A*24:34	706	AYSNNSIAI	0,7097
A24	HLA-A*24:34	755	QYGSFCTQL	0,6183
A24	HLA-A*24:34	788	IYKTPPIKDF	0,9047
A24	HLA-A*24:34	897	PFAMQMAYRF	0,5116
A24	HLA-A*24:34	1066	TYVPAQEKNF	0,9166
A24	HLA-A*24:34	1094	VFVSNGTHW	0,866
A24	HLA-A*24:34	1094	VFVSNGTHWF	0,8212
A24	HLA-A*24:34	1101	HWFVTQRNF	0,7483
A24	HLA-A*24:34	1137	VYDPLQPELDSF	0,7712

A24	HLA-A*24:34	1208	QYIKWPWYI	0,9658
A24	HLA-A*24:34	1208	QYIKWPWYIW	0,7348
A24	HLA-A*24:34	1211	KWPWYIWLGF	0,6265
A24	HLA-A*24:35	37	YYPDKVFRSSVL	0,601
A24	HLA-A*24:35	57	PFFSNVTWF	0,8728
A24	HLA-A*24:35	78	RFDNPVLPF	0,8538
A24	HLA-A*24:35	143	VYYHKNNKSW	0,7378
A24	HLA-A*24:35	144	YYHKNNKSW	0,8416
A24	HLA-A*24:35	151	SWMESEFRV	0,5046
A24	HLA-A*24:35	159	VYSSANNCTF	0,9582
A24	HLA-A*24:35	167	TFEYVSQPF	0,5365
A24	HLA-A*24:35	169	EYVSQPFLM	0,8484
A24	HLA-A*24:35	203	IYSKHTPINL	0,7085
A24	HLA-A*24:35	264	AYYVGYLQPRTF	0,8037
A24	HLA-A*24:35	265	YYVGYLQPRTF	0,9005
A24	HLA-A*24:35	268	GYLQPRTFLL	0,731
A24	HLA-A*24:35	269	YLQPRTFLL	0,5359
A24	HLA-A*24:35	312	IYQTSNFRV	0,785
A24	HLA-A*24:35	328	RFPNITNLCPF	0,8789
A24	HLA-A*24:35	346	RFASVYAW	0,521
A24	HLA-A*24:35	368	LYNSASFSTF	0,914
A24	HLA-A*24:35	395	VYADSFVI	0,6394
A24	HLA-A*24:35	448	NYNYLYRLF	0,9353
A24	HLA-A*24:35	488	CYFPLQSYGF	0,5885
A24	HLA-A*24:35	489	YFPLQSYGF	0,9442
A24	HLA-A*24:35	504	GYQPYRVVVLSF	0,6707
A24	HLA-A*24:35	507	PYRVVVLSF	0,8166
A24	HLA-A*24:35	558	KFLPFQQF	0,6948
A24	HLA-A*24:35	634	RVYSTGSNVF	0,7146
A24	HLA-A*24:35	635	VYSTGSNVF	0,9696
A24	HLA-A*24:35	755	QYGSFCTQL	0,5614
A24	HLA-A*24:35	788	IYKTPPIKDF	0,9226
A24	HLA-A*24:35	816	SFIEDLLF	0,7253
A24	HLA-A*24:35	1066	TYVPAQEKNF	0,9479
A24	HLA-A*24:35	1094	VFVSNGTHW	0,8658
A24	HLA-A*24:35	1094	VFVSNGTHWF	0,8335
A24	HLA-A*24:35	1101	HWFVTQRNF	0,754
A24	HLA-A*24:35	1137	VYDPLQPELDSF	0,8174
A24	HLA-A*24:35	1208	QYIKWPWYI	0,9613
A24	HLA-A*24:35	1208	QYIKWPWYIW	0,7582
A24	HLA-A*24:35	1211	KWPWYIWLGF	0,6582
A24	HLA-A*24:37	37	YYPDKVFRSSVL	0,601
A24	HLA-A*24:37	57	PFFSNVTWF	0,8728
A24	HLA-A*24:37	78	RFDNPVLPF	0,8538
A24	HLA-A*24:37	143	VYYHKNNKSW	0,7378
A24	HLA-A*24:37	144	YYHKNNKSW	0,8416
A24	HLA-A*24:37	151	SWMESEFRV	0,5046
A24	HLA-A*24:37	159	VYSSANNCTF	0,9582
A24	HLA-A*24:37	167	TFEYVSQPF	0,5365
A24	HLA-A*24:37	169	EYVSQPFLM	0,8484

A24	HLA-A*24:37	203	IYSKHTPINL	0,7085
A24	HLA-A*24:37	264	AYYVGYLQPRTF	0,8037
A24	HLA-A*24:37	265	YYVGYLQPRTF	0,9005
A24	HLA-A*24:37	268	GYLQPRTFLL	0,731
A24	HLA-A*24:37	269	YLQPRTFLL	0,5359
A24	HLA-A*24:37	312	IYQTSNFRV	0,785
A24	HLA-A*24:37	328	RFPNITNLCPF	0,8789
A24	HLA-A*24:37	346	RFASVYAW	0,521
A24	HLA-A*24:37	368	LYNSASFSTF	0,914
A24	HLA-A*24:37	395	VYADSFVI	0,6394
A24	HLA-A*24:37	448	NYNYLYRLF	0,9353
A24	HLA-A*24:37	488	CYFPLQSYGF	0,5885
A24	HLA-A*24:37	489	YFPLQSYGF	0,9442
A24	HLA-A*24:37	504	GYQPYRVVVLSF	0,6707
A24	HLA-A*24:37	507	PYRVVVLSF	0,8166
A24	HLA-A*24:37	558	KFLPFQQF	0,6948
A24	HLA-A*24:37	634	RVYSTGSNVF	0,7146
A24	HLA-A*24:37	635	VYSTGSNVF	0,9696
A24	HLA-A*24:37	755	QYGSFCTQL	0,5614
A24	HLA-A*24:37	788	IYKTPPIKDF	0,9226
A24	HLA-A*24:37	816	SFIEDLLF	0,7253
A24	HLA-A*24:37	1066	TYVPAQEKNF	0,9479
A24	HLA-A*24:37	1094	VFVSNGTHW	0,8658
A24	HLA-A*24:37	1094	VFVSNGTHWF	0,8335
A24	HLA-A*24:37	1101	HWFVTQRNF	0,754
A24	HLA-A*24:37	1137	VYDPLQPELDSF	0,8174
A24	HLA-A*24:37	1208	QYIKWPWYI	0,9613
A24	HLA-A*24:37	1208	QYIKWPWYIW	0,7582
A24	HLA-A*24:37	1211	KWPWYIWLGF	0,6582
A24	HLA-A*24:38	37	YYPDKVFRSSVL	0,601
A24	HLA-A*24:38	57	PFFSNVTWF	0,8728
A24	HLA-A*24:38	78	RFDNPVLPF	0,8538
A24	HLA-A*24:38	143	VYYHKNNKSW	0,7378
A24	HLA-A*24:38	144	YYHKNNKSW	0,8416
A24	HLA-A*24:38	151	SWMESEFRV	0,5046
A24	HLA-A*24:38	159	VYSSANNCTF	0,9582
A24	HLA-A*24:38	167	TFEYVSQPF	0,5365
A24	HLA-A*24:38	169	EYVSQPFLM	0,8484
A24	HLA-A*24:38	203	IYSKHTPINL	0,7085
A24	HLA-A*24:38	264	AYYVGYLQPRTF	0,8037
A24	HLA-A*24:38	265	YYVGYLQPRTF	0,9005
A24	HLA-A*24:38	268	GYLQPRTFLL	0,731
A24	HLA-A*24:38	269	YLQPRTFLL	0,5359
A24	HLA-A*24:38	312	IYQTSNFRV	0,785
A24	HLA-A*24:38	328	RFPNITNLCPF	0,8789
A24	HLA-A*24:38	346	RFASVYAW	0,521
A24	HLA-A*24:38	368	LYNSASFSTF	0,914
A24	HLA-A*24:38	395	VYADSFVI	0,6394
A24	HLA-A*24:38	448	NYNYLYRLF	0,9353
A24	HLA-A*24:38	488	CYFPLQSYGF	0,5885

A24	HLA-A*24:38	489	YFPLQSYGF	0,9442
A24	HLA-A*24:38	504	GYQPYRVVVLSF	0,6707
A24	HLA-A*24:38	507	PYRVVVLSF	0,8166
A24	HLA-A*24:38	558	KFLPFQQF	0,6948
A24	HLA-A*24:38	634	RVYSTGSNVF	0,7146
A24	HLA-A*24:38	635	VYSTGSNVF	0,9696
A24	HLA-A*24:38	755	QYGSFCTQL	0,5614
A24	HLA-A*24:38	788	IYKTPPIKDF	0,9226
A24	HLA-A*24:38	816	SFIEDLLF	0,7253
A24	HLA-A*24:38	1066	TYVPAQEKNF	0,9479
A24	HLA-A*24:38	1094	VFVSNGTHW	0,8658
A24	HLA-A*24:38	1094	VFVSNGTHWF	0,8335
A24	HLA-A*24:38	1101	HWFVTQRNF	0,754
A24	HLA-A*24:38	1137	VYDPLQPELDSF	0,8174
A24	HLA-A*24:38	1208	QYIKWPWYI	0,9613
A24	HLA-A*24:38	1208	QYIKWPWYIW	0,7582
A24	HLA-A*24:38	1211	KWPWYIWLGF	0,6582
A24	HLA-A*24:39	37	YYPDKVFRSSVL	0,601
A24	HLA-A*24:39	57	PFFSNVTWF	0,8728
A24	HLA-A*24:39	78	RFDNPVLPF	0,8538
A24	HLA-A*24:39	143	VYYHKNNKSW	0,7378
A24	HLA-A*24:39	144	YYHKNNKSW	0,8416
A24	HLA-A*24:39	151	SWMESEFRV	0,5046
A24	HLA-A*24:39	159	VYSSANNCTF	0,9582
A24	HLA-A*24:39	167	TFEYVSQPF	0,5365
A24	HLA-A*24:39	169	EYVSQPFLM	0,8484
A24	HLA-A*24:39	203	IYSKHTPINL	0,7085
A24	HLA-A*24:39	264	AYYVGYLQPRTF	0,8037
A24	HLA-A*24:39	265	YYVGYLQPRTF	0,9005
A24	HLA-A*24:39	268	GYLQPRTFLL	0,731
A24	HLA-A*24:39	269	YLQPRTFLL	0,5359
A24	HLA-A*24:39	312	IYQTSNFRV	0,785
A24	HLA-A*24:39	328	RFPNITNLCPF	0,8789
A24	HLA-A*24:39	346	RFASVYAW	0,521
A24	HLA-A*24:39	368	LYNSASFSTF	0,914
A24	HLA-A*24:39	395	VYADSFVI	0,6394
A24	HLA-A*24:39	448	NYNYLYRLF	0,9353
A24	HLA-A*24:39	488	CYFPLQSYGF	0,5885
A24	HLA-A*24:39	489	YFPLQSYGF	0,9442
A24	HLA-A*24:39	504	GYQPYRVVVLSF	0,6707
A24	HLA-A*24:39	507	PYRVVVLSF	0,8166
A24	HLA-A*24:39	558	KFLPFQQF	0,6948
A24	HLA-A*24:39	634	RVYSTGSNVF	0,7146
A24	HLA-A*24:39	635	VYSTGSNVF	0,9696
A24	HLA-A*24:39	755	QYGSFCTQL	0,5614
A24	HLA-A*24:39	788	IYKTPPIKDF	0,9226
A24	HLA-A*24:39	816	SFIEDLLF	0,7253
A24	HLA-A*24:39	1066	TYVPAQEKNF	0,9479
A24	HLA-A*24:39	1094	VFVSNGTHW	0,8658
A24	HLA-A*24:39	1094	VFVSNGTHWF	0,8335

A24	HLA-A*24:39	1101	HWFVTQRNF	0,754
A24	HLA-A*24:39	1137	VYDPLQPELDSF	0,8174
A24	HLA-A*24:39	1208	QYIKWPWYI	0,9613
A24	HLA-A*24:39	1208	QYIKWPWYIW	0,7582
A24	HLA-A*24:39	1211	KWPWYIWLGF	0,6582
A24	HLA-A*24:43	37	YYPDKVFRSSVL	0,601
A24	HLA-A*24:43	57	PFFSNVTWF	0,8728
A24	HLA-A*24:43	78	RFDNPVLPF	0,8538
A24	HLA-A*24:43	143	VYYHKNNKSW	0,7378
A24	HLA-A*24:43	144	YYHKNNKSW	0,8416
A24	HLA-A*24:43	151	SWMESEFRV	0,5046
A24	HLA-A*24:43	159	VYSSANNCTF	0,9582
A24	HLA-A*24:43	167	TFEYVSQPF	0,5365
A24	HLA-A*24:43	169	EYVSQPFLM	0,8484
A24	HLA-A*24:43	203	IYSKHTPINL	0,7085
A24	HLA-A*24:43	264	AYYVGYLQPRTF	0,8037
A24	HLA-A*24:43	265	YYVGYLQPRTF	0,9005
A24	HLA-A*24:43	268	GYLQPRTFLL	0,731
A24	HLA-A*24:43	269	YLQPRTFLL	0,5359
A24	HLA-A*24:43	312	IYQTSNFRV	0,785
A24	HLA-A*24:43	328	RFPNITNLCPF	0,8789
A24	HLA-A*24:43	346	RFASVYAW	0,521
A24	HLA-A*24:43	368	LYNSASFSTF	0,914
A24	HLA-A*24:43	395	VYADSFVI	0,6394
A24	HLA-A*24:43	448	NYNYLYRLF	0,9353
A24	HLA-A*24:43	488	CYFPLQSYGF	0,5885
A24	HLA-A*24:43	489	YFPLQSYGF	0,9442
A24	HLA-A*24:43	504	GYQPYRVVVLSF	0,6707
A24	HLA-A*24:43	507	PYRVVVLSF	0,8166
A24	HLA-A*24:43	558	KFLPFQQF	0,6948
A24	HLA-A*24:43	634	RVYSTGSNVF	0,7146
A24	HLA-A*24:43	635	VYSTGSNVF	0,9696
A24	HLA-A*24:43	755	QYGSFCTQL	0,5614
A24	HLA-A*24:43	788	IYKTPPIKDF	0,9226
A24	HLA-A*24:43	816	SFIEDLLF	0,7253
A24	HLA-A*24:43	1066	TYVPAQEKNF	0,9479
A24	HLA-A*24:43	1094	VFVSNGTHW	0,8658
A24	HLA-A*24:43	1094	VFVSNGTHWF	0,8335
A24	HLA-A*24:43	1101	HWFVTQRNF	0,754
A24	HLA-A*24:43	1137	VYDPLQPELDSF	0,8174
A24	HLA-A*24:43	1208	QYIKWPWYI	0,9613
A24	HLA-A*24:43	1208	QYIKWPWYIW	0,7582
A24	HLA-A*24:43	1211	KWPWYIWLGF	0,6582
A24	HLA-A*24:46	37	YYPDKVFRSSVL	0,5855
A24	HLA-A*24:46	57	PFFSNVTWF	0,8459
A24	HLA-A*24:46	78	RFDNPVLPF	0,8229
A24	HLA-A*24:46	159	VYSSANNCTF	0,9103
A24	HLA-A*24:46	169	EYVSQPFLM	0,851
A24	HLA-A*24:46	264	AYYVGYLQPRTF	0,6417
A24	HLA-A*24:46	265	YYVGYLQPRTF	0,8194

A24	HLA-A*24:46	268	GYLQPRTFLL	0,6533
A24	HLA-A*24:46	269	YLQPRTFLL	0,5285
A24	HLA-A*24:46	312	IYQTSNFRV	0,6584
A24	HLA-A*24:46	328	RFPNITNLCPF	0,7845
A24	HLA-A*24:46	368	LYNSASFSTF	0,8513
A24	HLA-A*24:46	395	VYADSFVI	0,5561
A24	HLA-A*24:46	448	NYNYLYRLF	0,871
A24	HLA-A*24:46	488	CYFPLQSYGF	0,5138
A24	HLA-A*24:46	489	YFPLQSYGF	0,9325
A24	HLA-A*24:46	504	GYQPYRVVVLSF	0,5468
A24	HLA-A*24:46	635	VYSTGSNVF	0,9278
A24	HLA-A*24:46	755	QYGSFCTQL	0,5152
A24	HLA-A*24:46	816	SFIEDLLF	0,7078
A24	HLA-A*24:46	1066	TYVPAQEKNF	0,9054
A24	HLA-A*24:46	1094	VFVSNGTHWF	0,756
A24	HLA-A*24:46	1137	VYDPLQPELDSF	0,7602
A24	HLA-A*24:46	1208	QYIKWPWYI	0,9343
A24	HLA-A*24:46	1208	QYIKWPWYIW	0,6481
A24	HLA-A*24:46	1211	KWPWYIWLGF	0,5239
A24	HLA-A*24:47	37	YYPDKVFRSSVL	0,601
A24	HLA-A*24:47	57	PFFSNVTWF	0,8728
A24	HLA-A*24:47	78	RFDNPVLPF	0,8538
A24	HLA-A*24:47	143	VYYHKNNKSW	0,7378
A24	HLA-A*24:47	144	YYHKNNKSW	0,8416
A24	HLA-A*24:47	151	SWMESEFRV	0,5046
A24	HLA-A*24:47	159	VYSSANNCTF	0,9582
A24	HLA-A*24:47	167	TFEYVSQPF	0,5365
A24	HLA-A*24:47	169	EYVSQPFLM	0,8484
A24	HLA-A*24:47	203	IYSKHTPINL	0,7085
A24	HLA-A*24:47	264	AYYVGYLQPRTF	0,8037
A24	HLA-A*24:47	265	YYVGYLQPRTF	0,9005
A24	HLA-A*24:47	268	GYLQPRTFLL	0,731
A24	HLA-A*24:47	269	YLQPRTFLL	0,5359
A24	HLA-A*24:47	312	IYQTSNFRV	0,785
A24	HLA-A*24:47	328	RFPNITNLCPF	0,8789
A24	HLA-A*24:47	346	RFASVYAW	0,521
A24	HLA-A*24:47	368	LYNSASFSTF	0,914
A24	HLA-A*24:47	395	VYADSFVI	0,6394
A24	HLA-A*24:47	448	NYNYLYRLF	0,9353
A24	HLA-A*24:47	488	CYFPLQSYGF	0,5885
A24	HLA-A*24:47	489	YFPLQSYGF	0,9442
A24	HLA-A*24:47	504	GYQPYRVVVLSF	0,6707
A24	HLA-A*24:47	507	PYRVVVLSF	0,8166
A24	HLA-A*24:47	558	KFLPFQQF	0,6948
A24	HLA-A*24:47	634	RVYSTGSNVF	0,7146
A24	HLA-A*24:47	635	VYSTGSNVF	0,9696
A24	HLA-A*24:47	755	QYGSFCTQL	0,5614
A24	HLA-A*24:47	788	IYKTPPIKDF	0,9226
A24	HLA-A*24:47	816	SFIEDLLF	0,7253
A24	HLA-A*24:47	1066	TYVPAQEKNF	0,9479

A24	HLA-A*24:47	1094	VFVSNGTHW	0,8658
A24	HLA-A*24:47	1094	VFVSNGTHWF	0,8335
A24	HLA-A*24:47	1101	HWFVTQRNF	0,754
A24	HLA-A*24:47	1137	VYDPLQPELDSF	0,8174
A24	HLA-A*24:47	1208	QYIKWPWYI	0,9613
A24	HLA-A*24:47	1208	QYIKWPWYIW	0,7582
A24	HLA-A*24:47	1211	KWPWYIWLGF	0,6582
A24	HLA-A*24:49	37	YYPDKVFRSSVL	0,601
A24	HLA-A*24:49	57	PFFSNVTWF	0,8728
A24	HLA-A*24:49	78	RFDNPVLPF	0,8538
A24	HLA-A*24:49	143	VYYHKNNKSW	0,7378
A24	HLA-A*24:49	144	YYHKNNKSW	0,8416
A24	HLA-A*24:49	151	SWMESEFRV	0,5046
A24	HLA-A*24:49	159	VYSSANNCTF	0,9582
A24	HLA-A*24:49	167	TFEYVSQPF	0,5365
A24	HLA-A*24:49	169	EYVSQPFLM	0,8484
A24	HLA-A*24:49	203	IYSKHTPINL	0,7085
A24	HLA-A*24:49	264	AYYVGYLQPRTF	0,8037
A24	HLA-A*24:49	265	YYVGYLQPRTF	0,9005
A24	HLA-A*24:49	268	GYLQPRTFLL	0,731
A24	HLA-A*24:49	269	YLQPRTFLL	0,5359
A24	HLA-A*24:49	312	IYQTSNFRV	0,785
A24	HLA-A*24:49	328	RFPNITNLCPF	0,8789
A24	HLA-A*24:49	346	RFASVYAW	0,521
A24	HLA-A*24:49	368	LYNSASFSTF	0,914
A24	HLA-A*24:49	395	VYADSFVI	0,6394
A24	HLA-A*24:49	448	NYNYLYRLF	0,9353
A24	HLA-A*24:49	488	CYFPLQSYGF	0,5885
A24	HLA-A*24:49	489	YFPLQSYGF	0,9442
A24	HLA-A*24:49	504	GYQPYRVVVLSF	0,6707
A24	HLA-A*24:49	507	PYRVVVLSF	0,8166
A24	HLA-A*24:49	558	KFLPFQQF	0,6948
A24	HLA-A*24:49	634	RVYSTGSNVF	0,7146
A24	HLA-A*24:49	635	VYSTGSNVF	0,9696
A24	HLA-A*24:49	755	QYGSFCTQL	0,5614
A24	HLA-A*24:49	788	IYKTPPIKDF	0,9226
A24	HLA-A*24:49	816	SFIEDLLF	0,7253
A24	HLA-A*24:49	1066	TYVPAQEKNF	0,9479
A24	HLA-A*24:49	1094	VFVSNGTHW	0,8658
A24	HLA-A*24:49	1094	VFVSNGTHWF	0,8335
A24	HLA-A*24:49	1101	HWFVTQRNF	0,754
A24	HLA-A*24:49	1137	VYDPLQPELDSF	0,8174
A24	HLA-A*24:49	1208	QYIKWPWYI	0,9613
A24	HLA-A*24:49	1208	QYIKWPWYIW	0,7582
A24	HLA-A*24:49	1211	KWPWYIWLGF	0,6582
B07	HLA-B*07:02	24	LPPAYTNSF	0,5263
B07	HLA-B*07:02	38	YPDKVFRSSV	0,5308
B07	HLA-B*07:02	208	TPINLVRDL	0,7332
B07	HLA-B*07:02	216	LPQGFSAL	0,6854
B07	HLA-B*07:02	462	KPFERDISTEI	0,6344

B07	HLA-B*07:02	506	QPYRVVVL	0,6389
B07	HLA-B*07:02	526	GPKKSTNLV	0,6037
B07	HLA-B*07:02	620	VPVAIHADQL	0,5101
B07	HLA-B*07:02	680	SPRRARSVA	0,7296
B07	HLA-B*07:02	714	IPTNFTISV	0,6596
B07	HLA-B*07:02	1056	APHGVVFL	0,6399
B07	HLA-B*07:02	1261	SEPVLKGVKL	0,6496
B07	HLA-B*07:03	24	LPPAYTNSF	0,6635
B07	HLA-B*07:03	38	YPDKVFRSSV	0,6468
B07	HLA-B*07:03	38	YPDKVFRSSVL	0,5441
B07	HLA-B*07:03	208	TPINLVRDL	0,7801
B07	HLA-B*07:03	216	LPQGFSAL	0,8331
B07	HLA-B*07:03	462	KPFERDISTEI	0,5705
B07	HLA-B*07:03	506	QPYRVVVL	0,8112
B07	HLA-B*07:03	526	GPKKSTNL	0,5568
B07	HLA-B*07:03	526	GPKKSTNLV	0,6514
B07	HLA-B*07:03	588	TPCSFGGVSV	0,525
B07	HLA-B*07:03	620	VPVAIHADQL	0,5699
B07	HLA-B*07:03	680	SPRRARSVA	0,7613
B07	HLA-B*07:03	714	IPTNFTISV	0,7313
B07	HLA-B*07:03	869	MIAQYTSAL	0,584
B07	HLA-B*07:03	1052	FPQSAPHGV	0,5776
B07	HLA-B*07:03	1052	FPQSAPHGVVF	0,6134
B07	HLA-B*07:03	1056	APHGVVFL	0,7644
B07	HLA-B*07:03	1261	SEPVLKGVKL	0,7262
B07	HLA-B*07:04	208	TPINLVRDL	0,6264
B07	HLA-B*07:04	462	KPFERDISTEI	0,5191
B07	HLA-B*07:04	506	QPYRVVVL	0,6271
B07	HLA-B*07:04	526	GPKKSTNLV	0,5173
B07	HLA-B*07:04	680	SPRRARSVA	0,6865
B07	HLA-B*07:04	714	IPTNFTISV	0,5207
B07	HLA-B*07:04	1261	SEPVLKGVKL	0,5427
B07	HLA-B*07:05	24	LPPAYTNSF	0,5378
B07	HLA-B*07:05	38	YPDKVFRSSV	0,6462
B07	HLA-B*07:05	208	TPINLVRDL	0,7572
B07	HLA-B*07:05	216	LPQGFSAL	0,7578
B07	HLA-B*07:05	462	KPFERDISTEI	0,6075
B07	HLA-B*07:05	506	QPYRVVVL	0,7004
B07	HLA-B*07:05	526	GPKKSTNLV	0,6522
B07	HLA-B*07:05	588	TPCSFGGVSV	0,5218
B07	HLA-B*07:05	620	VPVAIHADQL	0,5518
B07	HLA-B*07:05	680	SPRRARSVA	0,7396
B07	HLA-B*07:05	714	IPTNFTISV	0,75
B07	HLA-B*07:05	1056	APHGVVFL	0,6769
B07	HLA-B*07:05	1261	SEPVLKGVKL	0,6685
B07	HLA-B*07:06	24	LPPAYTNSF	0,5378
B07	HLA-B*07:06	38	YPDKVFRSSV	0,6462
B07	HLA-B*07:06	208	TPINLVRDL	0,7572
B07	HLA-B*07:06	216	LPQGFSAL	0,7578
B07	HLA-B*07:06	462	KPFERDISTEI	0,6075

B07	HLA-B*07:06	506	QPYRVVVL	0,7004
B07	HLA-B*07:06	526	GPKKSTNLV	0,6522
B07	HLA-B*07:06	588	TPCSFGGVSV	0,5218
B07	HLA-B*07:06	620	VPVAIHADQL	0,5518
B07	HLA-B*07:06	680	SPRRARSVA	0,7396
B07	HLA-B*07:06	714	IPTNFTISV	0,75
B07	HLA-B*07:06	1056	APHGVVFL	0,6769
B07	HLA-B*07:06	1261	SEPVLKGVKL	0,6685
B07	HLA-B*07:15	38	YPDKVFRSSV	0,6004
B07	HLA-B*07:15	208	TPINLVRDL	0,7101
B07	HLA-B*07:15	216	LPQGFSAL	0,6649
B07	HLA-B*07:15	506	QPYRVVVL	0,6118
B07	HLA-B*07:15	526	GPKKSTNLV	0,5338
B07	HLA-B*07:15	680	SPRRARSVA	0,6589
B07	HLA-B*07:15	714	IPTNFTISV	0,7194
B07	HLA-B*07:15	1052	FPQSAPHGV	0,5075
B07	HLA-B*07:15	1056	APHGVVFL	0,6254
B07	HLA-B*07:15	1261	SEPVLKGVKL	0,5393
B07	HLA-B*07:19	24	LPPAYTNSF	0,5493
B07	HLA-B*07:19	208	TPINLVRDL	0,7209
B07	HLA-B*07:19	216	LPQGFSAL	0,5972
B07	HLA-B*07:19	462	KPFERDISTEI	0,5462
B07	HLA-B*07:19	506	QPYRVVVL	0,744
B07	HLA-B*07:19	526	GPKKSTNLV	0,6123
B07	HLA-B*07:19	680	SPRRARSV	0,5347
B07	HLA-B*07:19	680	SPRRARSVA	0,7444
B07	HLA-B*07:19	714	IPTNFTISV	0,6042
B07	HLA-B*07:19	1056	APHGVVFL	0,5804
B07	HLA-B*07:19	1261	SEPVLKGVKL	0,6196
B07	HLA-B*07:20	24	LPPAYTNSF	0,5771
B07	HLA-B*07:20	208	TPINLVRDL	0,6858
B07	HLA-B*07:20	216	LPQGFSAL	0,5023
B07	HLA-B*07:21	24	LPPAYTNSF	0,5263
B07	HLA-B*07:21	38	YPDKVFRSSV	0,5308
B07	HLA-B*07:21	208	TPINLVRDL	0,7332
B07	HLA-B*07:21	216	LPQGFSAL	0,6854
B07	HLA-B*07:21	462	KPFERDISTEI	0,6344
B07	HLA-B*07:21	506	QPYRVVVL	0,6389
B07	HLA-B*07:21	526	GPKKSTNLV	0,6037
B07	HLA-B*07:21	620	VPVAIHADQL	0,5101
B07	HLA-B*07:21	680	SPRRARSVA	0,7296
B07	HLA-B*07:21	714	IPTNFTISV	0,6596
B07	HLA-B*07:21	1056	APHGVVFL	0,6399
B07	HLA-B*07:21	1261	SEPVLKGVKL	0,6496
B07	HLA-B*07:22	24	LPPAYTNSF	0,5263
B07	HLA-B*07:22	38	YPDKVFRSSV	0,5308
B07	HLA-B*07:22	208	TPINLVRDL	0,7332
B07	HLA-B*07:22	216	LPQGFSAL	0,6854
B07	HLA-B*07:22	462	KPFERDISTEI	0,6344
B07	HLA-B*07:22	506	QPYRVVVL	0,6389

B07	HLA-B*07:22	526	GPKKSTNLV	0,6037
B07	HLA-B*07:22	620	VPVAIHADQL	0,5101
B07	HLA-B*07:22	680	SPRRARSVA	0,7296
B07	HLA-B*07:22	714	IPTNFTISV	0,6596
B07	HLA-B*07:22	1056	APHGVVFL	0,6399
B07	HLA-B*07:22	1261	SEPVLKGVKL	0,6496
B07	HLA-B*07:24	24	LPPAYTNSF	0,6317
B07	HLA-B*07:24	38	YPDKVFRSSV	0,5009
B07	HLA-B*07:24	84	LPFNDGVYF	0,6784
B07	HLA-B*07:24	208	TPINLVRDL	0,787
B07	HLA-B*07:24	216	LPQGFSAL	0,6678
B07	HLA-B*07:24	506	QPYRVVVL	0,6042
B07	HLA-B*07:24	526	GPKKSTNLV	0,5315
B07	HLA-B*07:24	680	SPRRARSVA	0,6404
B07	HLA-B*07:24	714	IPTNFTISV	0,6579
B07	HLA-B*07:24	1052	FPQSAPHGVVF	0,5403
B07	HLA-B*07:24	1261	SEPVLKGVKL	0,6013
B07	HLA-B*07:25	24	LPPAYTNSF	0,6744
B07	HLA-B*07:25	84	LPFNDGVYF	0,6682
B07	HLA-B*07:25	208	TPINLVRDL	0,7696
B07	HLA-B*07:25	216	LPQGFSAL	0,5739
B07	HLA-B*07:25	506	QPYRVVVL	0,6556
B07	HLA-B*07:25	620	VPVAIHADQL	0,5421
B07	HLA-B*07:25	714	IPTNFTISV	0,6917
B07	HLA-B*07:25	1052	FPQSAPHGV	0,5272
B07	HLA-B*07:25	1052	FPQSAPHGVVF	0,524
B07	HLA-B*07:25	1056	APHGVVFL	0,6273
B07	HLA-B*07:25	1261	SEPVLKGVKL	0,6103
B07	HLA-B*07:25	1262	EPVLKGVKL	0,6809
B07	HLA-B*07:26	208	TPINLVRDL	0,6472
B07	HLA-B*07:26	506	QPYRVVVL	0,5503
B07	HLA-B*07:26	680	SPRRARSVA	0,542
B07	HLA-B*07:26	714	IPTNFTISV	0,5255
B07	HLA-B*07:30	24	LPPAYTNSF	0,5263
B07	HLA-B*07:30	38	YPDKVFRSSV	0,5308
B07	HLA-B*07:30	208	TPINLVRDL	0,7332
B07	HLA-B*07:30	216	LPQGFSAL	0,6854
B07	HLA-B*07:30	462	KPFERDISTEI	0,6344
B07	HLA-B*07:30	506	QPYRVVVL	0,6389
B07	HLA-B*07:30	526	GPKKSTNLV	0,6037
B07	HLA-B*07:30	620	VPVAIHADQL	0,5101
B07	HLA-B*07:30	680	SPRRARSVA	0,7296
B07	HLA-B*07:30	714	IPTNFTISV	0,6596
B07	HLA-B*07:30	1056	APHGVVFL	0,6399
B07	HLA-B*07:30	1261	SEPVLKGVKL	0,6496
B07	HLA-B*07:31	24	LPPAYTNSF	0,6652
B07	HLA-B*07:31	38	YPDKVFRSSV	0,6505
B07	HLA-B*07:31	84	LPFNDGVYF	0,7128
B07	HLA-B*07:31	208	TPINLVRDL	0,8331
B07	HLA-B*07:31	216	LPQGFSAL	0,8036

B07	HLA-B*07:31	229	LPIGINITRF	0,5034
B07	HLA-B*07:31	462	KPFERDISTEI	0,6827
B07	HLA-B*07:31	506	QPYRVVVL	0,7867
B07	HLA-B*07:31	526	GPKKSTNLV	0,7144
B07	HLA-B*07:31	620	VPVAIHADQL	0,6023
B07	HLA-B*07:31	680	SPRRARSVA	0,8018
B07	HLA-B*07:31	714	IPTNFTISV	0,7547
B07	HLA-B*07:31	1052	FPQSAPHGV	0,5262
B07	HLA-B*07:31	1052	FPQSAPHGVVF	0,5664
B07	HLA-B*07:31	1056	APHGVVFL	0,7394
B07	HLA-B*07:31	1261	SEPVLKGVKL	0,7452
B07	HLA-B*07:31	1262	EPVLKGVKL	0,7089
B07	HLA-B*07:33	24	LPPAYTNSF	0,5263
B07	HLA-B*07:33	38	YPDKVFRSSV	0,5308
B07	HLA-B*07:33	208	TPINLVRDL	0,7332
B07	HLA-B*07:33	216	LPQGFSAL	0,6854
B07	HLA-B*07:33	462	KPFERDISTEI	0,6344
B07	HLA-B*07:33	506	QPYRVVVL	0,6389
B07	HLA-B*07:33	526	GPKKSTNLV	0,6037
B07	HLA-B*07:33	620	VPVAIHADQL	0,5101
B07	HLA-B*07:33	680	SPRRARSVA	0,7296
B07	HLA-B*07:33	714	IPTNFTISV	0,6596
B07	HLA-B*07:33	1056	APHGVVFL	0,6399
B07	HLA-B*07:33	1261	SEPVLKGVKL	0,6496
B07	HLA-B*07:34	24	LPPAYTNSF	0,5137
B07	HLA-B*07:34	84	LPFNDGVYF	0,6562
B07	HLA-B*07:34	208	TPINLVRDL	0,7702
B07	HLA-B*07:34	216	LPQGFSAL	0,6635
B07	HLA-B*07:34	506	QPYRVVVL	0,7653
B07	HLA-B*07:34	526	GPKKSTNLV	0,5309
B07	HLA-B*07:34	680	SPRRARSVA	0,6471
B07	HLA-B*07:34	714	IPTNFTISV	0,7183
B07	HLA-B*07:34	1056	APHGVVFL	0,6103
B07	HLA-B*07:34	1261	SEPVLKGVKL	0,5539
B07	HLA-B*07:35	24	LPPAYTNSF	0,5263
B07	HLA-B*07:35	38	YPDKVFRSSV	0,5308
B07	HLA-B*07:35	208	TPINLVRDL	0,7332
B07	HLA-B*07:35	216	LPQGFSAL	0,6854
B07	HLA-B*07:35	462	KPFERDISTEI	0,6344
B07	HLA-B*07:35	506	QPYRVVVL	0,6389
B07	HLA-B*07:35	526	GPKKSTNLV	0,6037
B07	HLA-B*07:35	620	VPVAIHADQL	0,5101
B07	HLA-B*07:35	680	SPRRARSVA	0,7296
B07	HLA-B*07:35	714	IPTNFTISV	0,6596
B07	HLA-B*07:35	1056	APHGVVFL	0,6399
B07	HLA-B*07:35	1261	SEPVLKGVKL	0,6496
B07	HLA-B*07:39	24	LPPAYTNSF	0,5263
B07	HLA-B*07:39	38	YPDKVFRSSV	0,5308
B07	HLA-B*07:39	208	TPINLVRDL	0,7332
B07	HLA-B*07:39	216	LPQGFSAL	0,6854

B07	HLA-B*07:39	462	KPFERDISTEI	0,6344
B07	HLA-B*07:39	506	QPYRVVVL	0,6389
B07	HLA-B*07:39	526	GPKKSTNLV	0,6037
B07	HLA-B*07:39	620	VPVAIHADQL	0,5101
B07	HLA-B*07:39	680	SPRRARSVA	0,7296
B07	HLA-B*07:39	714	IPTNFTISV	0,6596
B07	HLA-B*07:39	1056	APHGVVFL	0,6399
B07	HLA-B*07:39	1261	SEPVLKGVKL	0,6496
B07	HLA-B*07:40	24	LPPAYTNSF	0,5378
B07	HLA-B*07:40	38	YPDKVFRSSV	0,6462
B07	HLA-B*07:40	208	TPINLVRDL	0,7572
B07	HLA-B*07:40	216	LPQGFSAL	0,7578
B07	HLA-B*07:40	462	KPFERDISTEI	0,6075
B07	HLA-B*07:40	506	QPYRVVVL	0,7004
B07	HLA-B*07:40	526	GPKKSTNLV	0,6522
B07	HLA-B*07:40	588	TPCSFGGVSV	0,5218
B07	HLA-B*07:40	620	VPVAIHADQL	0,5518
B07	HLA-B*07:40	680	SPRRARSVA	0,7396
B07	HLA-B*07:40	714	IPTNFTISV	0,75
B07	HLA-B*07:40	1056	APHGVVFL	0,6769
B07	HLA-B*07:40	1261	SEPVLKGVKL	0,6685
B07	HLA-B*07:41	24	LPPAYTNSF	0,5263
B07	HLA-B*07:41	38	YPDKVFRSSV	0,5308
B07	HLA-B*07:41	208	TPINLVRDL	0,7332
B07	HLA-B*07:41	216	LPQGFSAL	0,6854
B07	HLA-B*07:41	462	KPFERDISTEI	0,6344
B07	HLA-B*07:41	506	QPYRVVVL	0,6389
B07	HLA-B*07:41	526	GPKKSTNLV	0,6037
B07	HLA-B*07:41	620	VPVAIHADQL	0,5101
B07	HLA-B*07:41	680	SPRRARSVA	0,7296
B07	HLA-B*07:41	714	IPTNFTISV	0,6596
B07	HLA-B*07:41	1056	APHGVVFL	0,6399
B07	HLA-B*07:41	1261	SEPVLKGVKL	0,6496
B07	HLA-B*07:42	24	LPPAYTNSF	0,5263
B07	HLA-B*07:42	38	YPDKVFRSSV	0,5308
B07	HLA-B*07:42	208	TPINLVRDL	0,7332
B07	HLA-B*07:42	216	LPQGFSAL	0,6854
B07	HLA-B*07:42	462	KPFERDISTEI	0,6344
B07	HLA-B*07:42	506	QPYRVVVL	0,6389
B07	HLA-B*07:42	526	GPKKSTNLV	0,6037
B07	HLA-B*07:42	620	VPVAIHADQL	0,5101
B07	HLA-B*07:42	680	SPRRARSVA	0,7296
B07	HLA-B*07:42	714	IPTNFTISV	0,6596
B07	HLA-B*07:42	1056	APHGVVFL	0,6399
B07	HLA-B*07:42	1261	SEPVLKGVKL	0,6496
B07	HLA-B*07:43	84	LPFNDGVYF	0,6105
B07	HLA-B*07:43	208	TPINLVRDL	0,7315
B07	HLA-B*07:43	216	LPQGFSAL	0,5483
B07	HLA-B*07:43	506	QPYRVVVL	0,6776
B07	HLA-B*07:43	680	SPRRARSVA	0,6226

B07	HLA-B*07:43	714	IPTNFTISV	0,6031
B07	HLA-B*15:08	84	LPFNDGVYF	0,6442
B07	HLA-B*15:08	192	FVFKNIDGY	0,5399
B07	HLA-B*15:08	687	VASQSIIAY	0,7217
B07	HLA-B*15:08	699	LGAENSVAY	0,5058
B07	HLA-B*15:08	896	IPFAMQMAY	0,7301
B07	HLA-B*15:08	1054	QSAPHGVVF	0,5652
B07	HLA-B*35:01	24	LPPAYTNSF	0,8786
B07	HLA-B*35:01	30	NSFTRGVYY	0,5335
B07	HLA-B*35:01	56	LPFFSNVTW	0,8069
B07	HLA-B*35:01	83	VLPFNDGVYF	0,7228
B07	HLA-B*35:01	84	LPFNDGVY	0,824
B07	HLA-B*35:01	84	LPFNDGVYF	0,9877
B07	HLA-B*35:01	162	SANNCTFEY	0,6698
B07	HLA-B*35:01	192	FVFKNIDGY	0,7837
B07	HLA-B*35:01	229	LPIGINITRF	0,8394
B07	HLA-B*35:01	258	WTAGAAAYY	0,5393
B07	HLA-B*35:01	271	QPRTFLLKY	0,6565
B07	HLA-B*35:01	321	QPTESIVRF	0,9476
B07	HLA-B*35:01	343	NATRFASVY	0,7573
B07	HLA-B*35:01	361	CVADYSVLY	0,5167
B07	HLA-B*35:01	478	TPCNGVEGF	0,5961
B07	HLA-B*35:01	604	TSNQVAVLY	0,5492
B07	HLA-B*35:01	625	HADQLTPTW	0,7176
B07	HLA-B*35:01	664	IPIGAGICASY	0,8514
B07	HLA-B*35:01	687	VASQSIIAY	0,9634
B07	HLA-B*35:01	699	LGAENSVAY	0,8303
B07	HLA-B*35:01	714	IPTNFTISV	0,5007
B07	HLA-B*35:01	861	LPPLLTDEM	0,6186
B07	HLA-B*35:01	865	LTDEMIAQY	0,6118
B07	HLA-B*35:01	892	AALQIPFAM	0,5789
B07	HLA-B*35:01	895	QIPFAMQMAY	0,6098
B07	HLA-B*35:01	896	IPFAMQMAY	0,9872
B07	HLA-B*35:01	898	FAMQMAYRF	0,7448
B07	HLA-B*35:01	1052	FPQSAPHGVVF	0,8504
B07	HLA-B*35:01	1054	QSAPHGVVF	0,6307
B07	HLA-B*35:01	1095	FVSNGTHWF	0,5997
B07	HLA-B*35:03	84	LPFNDGVYF	0,8449
B07	HLA-B*35:03	714	IPTNFTISV	0,6549
B07	HLA-B*35:03	861	LPPLLTDEM	0,674
B07	HLA-B*35:03	1052	FPQSAPHGVVF	0,6557
B07	HLA-B*35:07	24	LPPAYTNSF	0,8786
B07	HLA-B*35:07	30	NSFTRGVYY	0,5335
B07	HLA-B*35:07	56	LPFFSNVTW	0,8069
B07	HLA-B*35:07	83	VLPFNDGVYF	0,7228
B07	HLA-B*35:07	84	LPFNDGVY	0,824
B07	HLA-B*35:07	84	LPFNDGVYF	0,9877
B07	HLA-B*35:07	162	SANNCTFEY	0,6698
B07	HLA-B*35:07	192	FVFKNIDGY	0,7837
B07	HLA-B*35:07	229	LPIGINITRF	0,8394

B07	HLA-B*35:07	258	WTAGAAAYY	0,5393
B07	HLA-B*35:07	271	QPRTFLLKY	0,6565
B07	HLA-B*35:07	321	QPTESIVRF	0,9476
B07	HLA-B*35:07	343	NATRFASVY	0,7573
B07	HLA-B*35:07	361	CVADYSVLY	0,5167
B07	HLA-B*35:07	478	TPCNGVEGF	0,5961
B07	HLA-B*35:07	604	TSNQVAVLY	0,5492
B07	HLA-B*35:07	625	HADQLTPTW	0,7176
B07	HLA-B*35:07	664	IPIGAGICASY	0,8514
B07	HLA-B*35:07	687	VASQSIIAY	0,9634
B07	HLA-B*35:07	699	LGAENSVAY	0,8303
B07	HLA-B*35:07	714	IPTNFTISV	0,5007
B07	HLA-B*35:07	861	LPPLLTDEM	0,6186
B07	HLA-B*35:07	865	LTDEMIAQY	0,6118
B07	HLA-B*35:07	892	AALQIPFAM	0,5789
B07	HLA-B*35:07	895	QIPFAMQMAY	0,6098
B07	HLA-B*35:07	896	IPFAMQMAY	0,9872
B07	HLA-B*35:07	898	FAMQMAYRF	0,7448
B07	HLA-B*35:07	1052	FPQSAPHGVVF	0,8504
B07	HLA-B*35:07	1054	QSAPHGVVF	0,6307
B07	HLA-B*35:07	1095	FVSNGTHWF	0,5997
B07	HLA-B*35:08	24	LPPAYTNSF	0,797
B07	HLA-B*35:08	56	LPFFSNVTW	0,7097
B07	HLA-B*35:08	84	LPFNDGVY	0,6312
B07	HLA-B*35:08	84	LPFNDGVYF	0,9511
B07	HLA-B*35:08	162	SANNCTFEY	0,5112
B07	HLA-B*35:08	229	LPIGINITRF	0,6418
B07	HLA-B*35:08	321	QPTESIVRF	0,8721
B07	HLA-B*35:08	343	NATRFASVY	0,5359
B07	HLA-B*35:08	664	IPIGAGICASY	0,6771
B07	HLA-B*35:08	687	VASQSIIAY	0,8861
B07	HLA-B*35:08	699	LGAENSVAY	0,6356
B07	HLA-B*35:08	861	LPPLLTDEM	0,5504
B07	HLA-B*35:08	896	IPFAMQMAY	0,9572
B07	HLA-B*35:08	1052	FPQSAPHGVVF	0,787
B07	HLA-B*35:11	24	LPPAYTNSF	0,8352
B07	HLA-B*35:11	30	NSFTRGVYY	0,7008
B07	HLA-B*35:11	56	LPFFSNVTW	0,6542
B07	HLA-B*35:11	83	VLPFNDGVYF	0,6879
B07	HLA-B*35:11	84	LPFNDGVY	0,8263
B07	HLA-B*35:11	84	LPFNDGVYF	0,9718
B07	HLA-B*35:11	160	YSSANNCTF	0,5303
B07	HLA-B*35:11	162	SANNCTFEY	0,5536
B07	HLA-B*35:11	192	FVFKNIDGY	0,7481
B07	HLA-B*35:11	212	LVRDLPQGF	0,575
B07	HLA-B*35:11	229	LPIGINITRF	0,775
B07	HLA-B*35:11	258	WTAGAAAYY	0,5592
B07	HLA-B*35:11	261	GAAAYYVGY	0,5071
B07	HLA-B*35:11	271	QPRTFLLKY	0,8033
B07	HLA-B*35:11	321	QPTESIVRF	0,879

B07	HLA-B*35:11	343	NATRFASVY	0,812
B07	HLA-B*35:11	366	SVLYNSASF	0,7009
B07	HLA-B*35:11	604	TSNQVAVLY	0,5291
B07	HLA-B*35:11	664	IPIGAGICASY	0,8485
B07	HLA-B*35:11	686	SVASQSIIAY	0,5827
B07	HLA-B*35:11	687	VASQSIIAY	0,9537
B07	HLA-B*35:11	699	LGAENSVAY	0,8145
B07	HLA-B*35:11	710	NSIAIPTNF	0,608
B07	HLA-B*35:11	865	LTDEMIAQY	0,5297
B07	HLA-B*35:11	869	MIAQYTSAL	0,5203
B07	HLA-B*35:11	886	WTFGAGAAL	0,5144
B07	HLA-B*35:11	892	AALQIPFAM	0,6266
B07	HLA-B*35:11	895	QIPFAMQMAY	0,6561
B07	HLA-B*35:11	896	IPFAMQMAY	0,9842
B07	HLA-B*35:11	898	FAMQMAYRF	0,6952
B07	HLA-B*35:11	1021	SANLAATKM	0,6657
B07	HLA-B*35:11	1052	FPQSAPHGVVF	0,7606
B07	HLA-B*35:11	1054	QSAPHGVVF	0,7902
B07	HLA-B*35:11	1095	FVSNGTHWF	0,6488
B07	HLA-B*35:11	1113	QIITTDNTF	0,5819
B07	HLA-B*35:14	24	LPPAYTNSF	0,7489
B07	HLA-B*35:14	84	LPFNDGVY	0,6173
B07	HLA-B*35:14	84	LPFNDGVYF	0,8767
B07	HLA-B*35:14	192	FVFKNIDGY	0,677
B07	HLA-B*35:14	229	LPIGINITRF	0,6057
B07	HLA-B*35:14	258	WTAGAAAYY	0,5528
B07	HLA-B*35:14	343	NATRFASVY	0,7039
B07	HLA-B*35:14	366	SVLYNSASF	0,5417
B07	HLA-B*35:14	664	IPIGAGICASY	0,6706
B07	HLA-B*35:14	686	SVASQSIIAY	0,5487
B07	HLA-B*35:14	687	VASQSIIAY	0,8589
B07	HLA-B*35:14	699	LGAENSVAY	0,6846
B07	HLA-B*35:14	896	IPFAMQMAY	0,9083
B07	HLA-B*35:14	898	FAMQMAYRF	0,501
B07	HLA-B*35:14	1052	FPQSAPHGVVF	0,5835
B07	HLA-B*35:14	1054	QSAPHGVVF	0,6632
B07	HLA-B*35:14	1095	FVSNGTHWF	0,5633
B07	HLA-B*35:15	24	LPPAYTNSF	0,7919
B07	HLA-B*35:15	56	LPFFSNVTW	0,7224
B07	HLA-B*35:15	83	VLPFNDGVYF	0,6829
B07	HLA-B*35:15	84	LPFNDGVY	0,7275
B07	HLA-B*35:15	84	LPFNDGVYF	0,9676
B07	HLA-B*35:15	162	SANNCTFEY	0,5711
B07	HLA-B*35:15	192	FVFKNIDGY	0,5935
B07	HLA-B*35:15	229	LPIGINITRF	0,7637
B07	HLA-B*35:15	321	QPTESIVRF	0,8872
B07	HLA-B*35:15	343	NATRFASVY	0,5685
B07	HLA-B*35:15	664	IPIGAGICASY	0,7825
B07	HLA-B*35:15	687	VASQSIIAY	0,9029
B07	HLA-B*35:15	699	LGAENSVAY	0,6765

B07	HLA-B*35:15	714	IPTNFTISV	0,5146
B07	HLA-B*35:15	861	LPPLLTDEM	0,5696
B07	HLA-B*35:15	892	AALQIPFAM	0,6734
B07	HLA-B*35:15	895	QIPFAMQMAY	0,5998
B07	HLA-B*35:15	896	IPFAMQMAY	0,9714
B07	HLA-B*35:15	898	FAMQMAYRF	0,665
B07	HLA-B*35:15	1052	FPQSAPHGVVF	0,781
B07	HLA-B*35:15	1054	QSAPHGVVF	0,5581
B07	HLA-B*35:21	24	LPPAYTNSF	0,8395
B07	HLA-B*35:21	30	NSFTRGVYY	0,7343
B07	HLA-B*35:21	56	LPFFSNVTW	0,7008
B07	HLA-B*35:21	83	VLPFNDGVYF	0,6004
B07	HLA-B*35:21	84	LPFNDGVY	0,8356
B07	HLA-B*35:21	84	LPFNDGVYF	0,9541
B07	HLA-B*35:21	138	DPFLGVYY	0,7423
B07	HLA-B*35:21	192	FVFKNIDGY	0,6843
B07	HLA-B*35:21	229	LPIGINITRF	0,7683
B07	HLA-B*35:21	271	QPRTFLLKY	0,8503
B07	HLA-B*35:21	321	QPTESIVRF	0,8652
B07	HLA-B*35:21	343	NATRFASVY	0,817
B07	HLA-B*35:21	366	SVLYNSASF	0,6264
B07	HLA-B*35:21	487	NCYFPLQSY	0,57
B07	HLA-B*35:21	490	FPLQSYGF	0,5592
B07	HLA-B*35:21	664	IPIGAGICASY	0,8085
B07	HLA-B*35:21	686	SVASQSIIAY	0,5007
B07	HLA-B*35:21	687	VASQSIIAY	0,9249
B07	HLA-B*35:21	699	LGAENSVAY	0,7697
B07	HLA-B*35:21	869	MIAQYTSAL	0,5073
B07	HLA-B*35:21	892	AALQIPFAM	0,5807
B07	HLA-B*35:21	895	QIPFAMQMAY	0,688
B07	HLA-B*35:21	896	IPFAMQMAY	0,9842
B07	HLA-B*35:21	897	PFAMQMAY	0,6134
B07	HLA-B*35:21	898	FAMQMAYRF	0,7194
B07	HLA-B*35:21	1021	SANLAATKM	0,5645
B07	HLA-B*35:21	1052	FPQSAPHGVVF	0,7764
B07	HLA-B*35:21	1054	QSAPHGVVF	0,7008
B07	HLA-B*35:21	1095	FVSNGTHWF	0,5717
B07	HLA-B*35:21	1130	IGIVNNTVY	0,5011
B07	HLA-B*35:22	24	LPPAYTNSF	0,6758
B07	HLA-B*35:22	84	LPFNDGVYF	0,8301
B07	HLA-B*35:22	216	LPQGFSAL	0,5263
B07	HLA-B*35:22	714	IPTNFTISV	0,8019
B07	HLA-B*35:22	861	LPPLLTDEM	0,6575
B07	HLA-B*35:22	896	IPFAMQMAY	0,568
B07	HLA-B*35:22	1052	FPQSAPHGV	0,7661
B07	HLA-B*35:22	1052	FPQSAPHGVVF	0,6166
B07	HLA-B*35:24	24	LPPAYTNSF	0,8659
B07	HLA-B*35:24	30	NSFTRGVYY	0,6
B07	HLA-B*35:24	56	LPFFSNVTW	0,8189
B07	HLA-B*35:24	56	LPFFSNVTWF	0,517

B07	HLA-B*35:24	83	VLPFNDGVYF	0,6239
B07	HLA-B*35:24	84	LPFNDGVY	0,8242
B07	HLA-B*35:24	84	LPFNDGVYF	0,9759
B07	HLA-B*35:24	138	DPFLGVYY	0,7649
B07	HLA-B*35:24	138	DPFLGVYYH	0,6062
B07	HLA-B*35:24	162	SANNCTFEY	0,5434
B07	HLA-B*35:24	192	FVFKNIDGY	0,6882
B07	HLA-B*35:24	229	LPIGINITRF	0,8081
B07	HLA-B*35:24	271	QPRTFLLKY	0,7209
B07	HLA-B*35:24	321	QPTESIVRF	0,9142
B07	HLA-B*35:24	343	NATRFASVY	0,7681
B07	HLA-B*35:24	490	FPLQSYGF	0,6001
B07	HLA-B*35:24	604	TSNQVAVLY	0,5175
B07	HLA-B*35:24	625	HADQLTPTW	0,6622
B07	HLA-B*35:24	664	IPIGAGICASY	0,7856
B07	HLA-B*35:24	687	VASQSIIAY	0,9318
B07	HLA-B*35:24	699	LGAENSVAY	0,7685
B07	HLA-B*35:24	714	IPTNFTISV	0,5262
B07	HLA-B*35:24	861	LPPLLTDEM	0,5723
B07	HLA-B*35:24	865	LTDEMIAQY	0,5411
B07	HLA-B*35:24	892	AALQIPFAM	0,5331
B07	HLA-B*35:24	895	QIPFAMQMAY	0,6479
B07	HLA-B*35:24	896	IPFAMQMAY	0,987
B07	HLA-B*35:24	897	PFAMQMAY	0,5448
B07	HLA-B*35:24	898	FAMQMAYRF	0,7695
B07	HLA-B*35:24	1052	FPQSAPHGVVF	0,8489
B07	HLA-B*35:24	1054	QSAPHGVVF	0,5377
B07	HLA-B*35:24	1095	FVSNGTHWF	0,5268
B07	HLA-B*35:31	38	YPDKVFRSSVL	0,5154
B07	HLA-B*35:31	84	LPFNDGVYF	0,7397
B07	HLA-B*35:31	216	LPQGFSAL	0,5549
B07	HLA-B*35:31	714	IPTNFTISV	0,825
B07	HLA-B*35:31	861	LPPLLTDEM	0,5885
B07	HLA-B*35:31	892	AALQIPFAM	0,5669
B07	HLA-B*35:31	1052	FPQSAPHGV	0,7222
B07	HLA-B*35:31	1052	FPQSAPHGVVF	0,5623
B07	HLA-B*35:32	24	LPPAYTNSF	0,9052
B07	HLA-B*35:32	30	NSFTRGVYY	0,5702
B07	HLA-B*35:32	56	LPFFSNVTW	0,8087
B07	HLA-B*35:32	56	LPFFSNVTWF	0,5164
B07	HLA-B*35:32	83	VLPFNDGVYF	0,7561
B07	HLA-B*35:32	84	LPFNDGVY	0,8314
B07	HLA-B*35:32	84	LPFNDGVYF	0,9881
B07	HLA-B*35:32	160	YSSANNCTF	0,5049
B07	HLA-B*35:32	162	SANNCTFEY	0,7098
B07	HLA-B*35:32	192	FVFKNIDGY	0,8032
B07	HLA-B*35:32	229	LPIGINITRF	0,8498
B07	HLA-B*35:32	258	WTAGAAAYY	0,5683
B07	HLA-B*35:32	271	QPRTFLLKY	0,6724
B07	HLA-B*35:32	321	QPTESIVRF	0,9486

B07	HLA-B*35:32	343	NATRFASVY	0,7659
B07	HLA-B*35:32	361	CVADYSVLY	0,53
B07	HLA-B*35:32	478	TPCNGVEGF	0,6358
B07	HLA-B*35:32	490	FPLQSYGF	0,5082
B07	HLA-B*35:32	604	TSNQVAVLY	0,5842
B07	HLA-B*35:32	625	HADQLTPTW	0,7442
B07	HLA-B*35:32	664	IPIGAGICASY	0,8564
B07	HLA-B*35:32	686	SVASQSIIAY	0,5225
B07	HLA-B*35:32	687	VASQSIIAY	0,9698
B07	HLA-B*35:32	699	LGAENSVAY	0,863
B07	HLA-B*35:32	710	NSIAIPTNF	0,5244
B07	HLA-B*35:32	714	IPTNFTISV	0,5438
B07	HLA-B*35:32	861	LPPLLTDEM	0,6652
B07	HLA-B*35:32	865	LTDEMIAQY	0,6517
B07	HLA-B*35:32	892	AALQIPFAM	0,6373
B07	HLA-B*35:32	895	QIPFAMQMAY	0,599
B07	HLA-B*35:32	896	IPFAMQMAY	0,9853
B07	HLA-B*35:32	898	FAMQMAYRF	0,7754
B07	HLA-B*35:32	1021	SANLAATKM	0,5136
B07	HLA-B*35:32	1052	FPQSAPHGV	0,5544
B07	HLA-B*35:32	1052	FPQSAPHGVVF	0,8763
B07	HLA-B*35:32	1054	QSAPHGVVF	0,697
B07	HLA-B*35:32	1095	FVSNGTHWF	0,6496
B07	HLA-B*35:32	1113	QIITTDNTF	0,5211
B07	HLA-B*35:33	714	IPTNFTISV	0,6955
B07	HLA-B*35:33	861	LPPLLTDEM	0,589
B07	HLA-B*35:35	24	LPPAYTNSF	0,8931
B07	HLA-B*35:35	30	NSFTRGVYY	0,6432
B07	HLA-B*35:35	38	YPDKVFRSS	0,5022
B07	HLA-B*35:35	56	LPFFSNVTW	0,8081
B07	HLA-B*35:35	56	LPFFSNVTWF	0,5997
B07	HLA-B*35:35	81	NPVLPFNDGVYF	0,5813
B07	HLA-B*35:35	83	VLPFNDGVYF	0,7798
B07	HLA-B*35:35	84	LPFNDGVY	0,8427
B07	HLA-B*35:35	84	LPFNDGVYF	0,987
B07	HLA-B*35:35	160	YSSANNCTF	0,5537
B07	HLA-B*35:35	162	SANNCTFEY	0,7379
B07	HLA-B*35:35	192	FVFKNIDGY	0,7847
B07	HLA-B*35:35	196	NIDGYFKIY	0,5682
B07	HLA-B*35:35	229	LPIGINITRF	0,8518
B07	HLA-B*35:35	258	WTAGAAAYY	0,6006
B07	HLA-B*35:35	261	GAAAYYVGY	0,5655
B07	HLA-B*35:35	271	QPRTFLLKY	0,7481
B07	HLA-B*35:35	321	QPTESIVRF	0,9497
B07	HLA-B*35:35	329	FPNITNLCPF	0,5517
B07	HLA-B*35:35	343	NATRFASVY	0,791
B07	HLA-B*35:35	361	CVADYSVLY	0,579
B07	HLA-B*35:35	366	SVLYNSASF	0,6081
B07	HLA-B*35:35	392	FTNVYADSF	0,5023
B07	HLA-B*35:35	462	KPFERDISTEIY	0,6963

B07	HLA-B*35:35	478	TPCNGVEGF	0,6736
B07	HLA-B*35:35	490	FPLQSYGF	0,6408
B07	HLA-B*35:35	604	TSNQVAVLY	0,653
B07	HLA-B*35:35	625	HADQLTPTW	0,77
B07	HLA-B*35:35	652	GAEHVNNSY	0,6983
B07	HLA-B*35:35	664	IPIGAGICASY	0,8472
B07	HLA-B*35:35	686	SVASQSIIAY	0,5942
B07	HLA-B*35:35	687	VASQSIIAY	0,9671
B07	HLA-B*35:35	699	LGAENSVAY	0,8503
B07	HLA-B*35:35	710	NSIAIPTNF	0,6147
B07	HLA-B*35:35	714	IPTNFTISV	0,6668
B07	HLA-B*35:35	861	LPPLLTDEM	0,6989
B07	HLA-B*35:35	865	LTDEMIAQY	0,7051
B07	HLA-B*35:35	869	MIAQYTSAL	0,5063
B07	HLA-B*35:35	886	WTFGAGAAL	0,5733
B07	HLA-B*35:35	892	AALQIPFAM	0,7769
B07	HLA-B*35:35	895	QIPFAMQMAY	0,6833
B07	HLA-B*35:35	896	IPFAMQMAY	0,9858
B07	HLA-B*35:35	897	PFAMQMAY	0,5282
B07	HLA-B*35:35	898	FAMQMAYRF	0,8293
B07	HLA-B*35:35	1021	SANLAATKM	0,6342
B07	HLA-B*35:35	1052	FPQSAPHGV	0,6179
B07	HLA-B*35:35	1052	FPQSAPHGVVF	0,8964
B07	HLA-B*35:35	1054	QSAPHGVVF	0,7525
B07	HLA-B*35:35	1056	APHGVVFLHVTY	0,6046
B07	HLA-B*35:35	1089	FPREGVFVS	0,5258
B07	HLA-B*35:35	1095	FVSNGTHWF	0,7045
B07	HLA-B*35:35	1113	QIITTDNTF	0,592
B07	HLA-B*35:36	84	LPFNDGVYF	0,8449
B07	HLA-B*35:36	714	IPTNFTISV	0,6549
B07	HLA-B*35:36	861	LPPLLTDEM	0,674
B07	HLA-B*35:36	1052	FPQSAPHGVVF	0,6557
B07	HLA-B*35:38	1052	FPQSAPHGVVF	0,6186
B07	HLA-B*35:41	24	LPPAYTNSF	0,9203
B07	HLA-B*35:41	30	NSFTRGVYY	0,69
B07	HLA-B*35:41	56	LPFFSNVTW	0,8713
B07	HLA-B*35:41	56	LPFFSNVTWF	0,6188
B07	HLA-B*35:41	81	NPVLPFNDGVYF	0,5272
B07	HLA-B*35:41	83	VLPFNDGVYF	0,7855
B07	HLA-B*35:41	84	LPFNDGVY	0,8727
B07	HLA-B*35:41	84	LPFNDGVYF	0,9927
B07	HLA-B*35:41	160	YSSANNCTF	0,5877
B07	HLA-B*35:41	162	SANNCTFEY	0,7881
B07	HLA-B*35:41	192	FVFKNIDGY	0,8905
B07	HLA-B*35:41	196	NIDGYFKIY	0,577
B07	HLA-B*35:41	229	LPIGINITRF	0,8935
B07	HLA-B*35:41	258	WTAGAAAYY	0,7113
B07	HLA-B*35:41	261	GAAAYYVGY	0,5904
B07	HLA-B*35:41	271	QPRTFLLKY	0,731
B07	HLA-B*35:41	321	QPTESIVRF	0,9644

B07	HLA-B*35:41	329	FPNITNLCPF	0,5798
B07	HLA-B*35:41	343	NATRFASVY	0,8709
B07	HLA-B*35:41	361	CVADYSVLY	0,6962
B07	HLA-B*35:41	366	SVLYNSASF	0,6032
B07	HLA-B*35:41	392	FTNVYADSF	0,5373
B07	HLA-B*35:41	462	KPFERDISTEIY	0,6461
B07	HLA-B*35:41	478	TPCNGVEGF	0,6721
B07	HLA-B*35:41	481	NGVEGFNCY	0,5089
B07	HLA-B*35:41	490	FPLQSYGF	0,5518
B07	HLA-B*35:41	604	TSNQVAVLY	0,7229
B07	HLA-B*35:41	625	HADQLTPTW	0,7962
B07	HLA-B*35:41	652	GAEHVNNSY	0,6893
B07	HLA-B*35:41	664	IPIGAGICASY	0,908
B07	HLA-B*35:41	686	SVASQSIIAY	0,6499
B07	HLA-B*35:41	687	VASQSIIAY	0,9842
B07	HLA-B*35:41	699	LGAENSVAY	0,9146
B07	HLA-B*35:41	710	NSIAIPTNF	0,6291
B07	HLA-B*35:41	714	IPTNFTISV	0,6147
B07	HLA-B*35:41	861	LPPLLTDEM	0,695
B07	HLA-B*35:41	865	LTDEMIAQY	0,7508
B07	HLA-B*35:41	892	AALQIPFAM	0,7116
B07	HLA-B*35:41	895	QIPFAMQMAY	0,7205
B07	HLA-B*35:41	896	IPFAMQMAY	0,993
B07	HLA-B*35:41	897	PFAMQMAY	0,5108
B07	HLA-B*35:41	898	FAMQMAYRF	0,8561
B07	HLA-B*35:41	1021	SANLAATKM	0,6172
B07	HLA-B*35:41	1052	FPQSAPHGV	0,5784
B07	HLA-B*35:41	1052	FPQSAPHGVVF	0,8981
B07	HLA-B*35:41	1054	QSAPHGVVF	0,7647
B07	HLA-B*35:41	1056	APHGVVFLHVTY	0,5536
B07	HLA-B*35:41	1089	FPREGVFVS	0,5633
B07	HLA-B*35:41	1095	FVSNGTHWF	0,7432
B07	HLA-B*35:41	1113	QIITTDNTF	0,6101
B07	HLA-B*35:41	1130	IGIVNNTVY	0,512
B07	HLA-B*35:42	24	LPPAYTNSF	0,8786
B07	HLA-B*35:42	30	NSFTRGVYY	0,5335
B07	HLA-B*35:42	56	LPFFSNVTW	0,8069
B07	HLA-B*35:42	83	VLPFNDGVYF	0,7228
B07	HLA-B*35:42	84	LPFNDGVY	0,824
B07	HLA-B*35:42	84	LPFNDGVYF	0,9877
B07	HLA-B*35:42	162	SANNCTFEY	0,6698
B07	HLA-B*35:42	192	FVFKNIDGY	0,7837
B07	HLA-B*35:42	229	LPIGINITRF	0,8394
B07	HLA-B*35:42	258	WTAGAAAYY	0,5393
B07	HLA-B*35:42	271	QPRTFLLKY	0,6565
B07	HLA-B*35:42	321	QPTESIVRF	0,9476
B07	HLA-B*35:42	343	NATRFASVY	0,7573
B07	HLA-B*35:42	361	CVADYSVLY	0,5167
B07	HLA-B*35:42	478	TPCNGVEGF	0,5961
B07	HLA-B*35:42	604	TSNQVAVLY	0,5492

B07	HLA-B*35:42	625	HADQLTPTW	0,7176
B07	HLA-B*35:42	664	IPIGAGICASY	0,8514
B07	HLA-B*35:42	687	VASQSIIAY	0,9634
B07	HLA-B*35:42	699	LGAENSVAY	0,8303
B07	HLA-B*35:42	714	IPTNFTISV	0,5007
B07	HLA-B*35:42	861	LPPLLTDEM	0,6186
B07	HLA-B*35:42	865	LTDEMIAQY	0,6118
B07	HLA-B*35:42	892	AALQIPFAM	0,5789
B07	HLA-B*35:42	895	QIPFAMQMAY	0,6098
B07	HLA-B*35:42	896	IPFAMQMAY	0,9872
B07	HLA-B*35:42	898	FAMQMAYRF	0,7448
B07	HLA-B*35:42	1052	FPQSAPHGVVF	0,8504
B07	HLA-B*35:42	1054	QSAPHGVVF	0,6307
B07	HLA-B*35:42	1095	FVSNGTHWF	0,5997
B07	HLA-B*35:43	24	LPPAYTNSF	0,7875
B07	HLA-B*35:43	30	NSFTRGVYY	0,5895
B07	HLA-B*35:43	84	LPFNDGVY	0,6447
B07	HLA-B*35:43	84	LPFNDGVYF	0,8888
B07	HLA-B*35:43	160	YSSANNCTF	0,5206
B07	HLA-B*35:43	192	FVFKNIDGY	0,7054
B07	HLA-B*35:43	229	LPIGINITRF	0,6427
B07	HLA-B*35:43	258	WTAGAAAYY	0,5829
B07	HLA-B*35:43	343	NATRFASVY	0,7189
B07	HLA-B*35:43	366	SVLYNSASF	0,6027
B07	HLA-B*35:43	664	IPIGAGICASY	0,6933
B07	HLA-B*35:43	686	SVASQSIIAY	0,5892
B07	HLA-B*35:43	687	VASQSIIAY	0,8891
B07	HLA-B*35:43	699	LGAENSVAY	0,7466
B07	HLA-B*35:43	896	IPFAMQMAY	0,9166
B07	HLA-B*35:43	898	FAMQMAYRF	0,543
B07	HLA-B*35:43	962	LVKQLSSNF	0,5124
B07	HLA-B*35:43	1052	FPQSAPHGVVF	0,6324
B07	HLA-B*35:43	1054	QSAPHGVVF	0,7377
B07	HLA-B*35:43	1095	FVSNGTHWF	0,6086
B07	HLA-B*35:43	1113	QIITTDNTF	0,5611
B07	HLA-B*35:45	84	LPFNDGVYF	0,7419
B07	HLA-B*35:45	687	VASQSIIAY	0,5885
B07	HLA-B*35:45	896	IPFAMQMAY	0,7957
B07	HLA-B*35:46	24	LPPAYTNSF	0,6364
B07	HLA-B*35:46	56	LPFFSNVTW	0,5165
B07	HLA-B*35:46	84	LPFNDGVYF	0,9096
B07	HLA-B*35:46	192	FVFKNIDGY	0,5961
B07	HLA-B*35:46	229	LPIGINITRF	0,5183
B07	HLA-B*35:46	321	QPTESIVRF	0,7199
B07	HLA-B*35:46	343	NATRFASVY	0,5076
B07	HLA-B*35:46	664	IPIGAGICASY	0,6105
B07	HLA-B*35:46	687	VASQSIIAY	0,8432
B07	HLA-B*35:46	699	LGAENSVAY	0,6048
B07	HLA-B*35:46	896	IPFAMQMAY	0,9239
B07	HLA-B*35:46	1052	FPQSAPHGVVF	0,5441

B07	HLA-B*35:46	1054	QSAPHGVVF	0,5022
B07	HLA-B*35:54	24	LPPAYTNSF	0,8786
B07	HLA-B*35:54	30	NSFTRGVYY	0,5335
B07	HLA-B*35:54	56	LPFFSNVTW	0,8069
B07	HLA-B*35:54	83	VLPFNDGVYF	0,7228
B07	HLA-B*35:54	84	LPFNDGVY	0,824
B07	HLA-B*35:54	84	LPFNDGVYF	0,9877
B07	HLA-B*35:54	162	SANNCTFEY	0,6698
B07	HLA-B*35:54	192	FVFKNIDGY	0,7837
B07	HLA-B*35:54	229	LPIGINITRF	0,8394
B07	HLA-B*35:54	258	WTAGAAAYY	0,5393
B07	HLA-B*35:54	271	QPRTFLLKY	0,6565
B07	HLA-B*35:54	321	QPTESIVRF	0,9476
B07	HLA-B*35:54	343	NATRFASVY	0,7573
B07	HLA-B*35:54	361	CVADYSVLY	0,5167
B07	HLA-B*35:54	478	TPCNGVEGF	0,5961
B07	HLA-B*35:54	604	TSNQVAVLY	0,5492
B07	HLA-B*35:54	625	HADQLTPTW	0,7176
B07	HLA-B*35:54	664	IPIGAGICASY	0,8514
B07	HLA-B*35:54	687	VASQSIIAY	0,9634
B07	HLA-B*35:54	699	LGAENSVAY	0,8303
B07	HLA-B*35:54	714	IPTNFTISV	0,5007
B07	HLA-B*35:54	861	LPPLLTDEM	0,6186
B07	HLA-B*35:54	865	LTDEMIAQY	0,6118
B07	HLA-B*35:54	892	AALQIPFAM	0,5789
B07	HLA-B*35:54	895	QIPFAMQMAY	0,6098
B07	HLA-B*35:54	896	IPFAMQMAY	0,9872
B07	HLA-B*35:54	898	FAMQMAYRF	0,7448
B07	HLA-B*35:54	1052	FPQSAPHGVVF	0,8504
B07	HLA-B*35:54	1054	QSAPHGVVF	0,6307
B07	HLA-B*35:54	1095	FVSNGTHWF	0,5997
B07	HLA-B*35:55	84	LPFNDGVYF	0,8449
B07	HLA-B*35:55	714	IPTNFTISV	0,6549
B07	HLA-B*35:55	861	LPPLLTDEM	0,674
B07	HLA-B*35:55	1052	FPQSAPHGVVF	0,6557
B07	HLA-B*35:57	24	LPPAYTNSF	0,8786
B07	HLA-B*35:57	30	NSFTRGVYY	0,5335
B07	HLA-B*35:57	56	LPFFSNVTW	0,8069
B07	HLA-B*35:57	83	VLPFNDGVYF	0,7228
B07	HLA-B*35:57	84	LPFNDGVY	0,824
B07	HLA-B*35:57	84	LPFNDGVYF	0,9877
B07	HLA-B*35:57	162	SANNCTFEY	0,6698
B07	HLA-B*35:57	192	FVFKNIDGY	0,7837
B07	HLA-B*35:57	229	LPIGINITRF	0,8394
B07	HLA-B*35:57	258	WTAGAAAYY	0,5393
B07	HLA-B*35:57	271	QPRTFLLKY	0,6565
B07	HLA-B*35:57	321	QPTESIVRF	0,9476
B07	HLA-B*35:57	343	NATRFASVY	0,7573
B07	HLA-B*35:57	361	CVADYSVLY	0,5167
B07	HLA-B*35:57	478	TPCNGVEGF	0,5961

B07	HLA-B*35:57	604	TSNQVAVLY	0,5492
B07	HLA-B*35:57	625	HADQLTPTW	0,7176
B07	HLA-B*35:57	664	IPIGAGICASY	0,8514
B07	HLA-B*35:57	687	VASQSIIAY	0,9634
B07	HLA-B*35:57	699	LGAENSVAY	0,8303
B07	HLA-B*35:57	714	IPTNFTISV	0,5007
B07	HLA-B*35:57	861	LPPLLTDEM	0,6186
B07	HLA-B*35:57	865	LTDEMIAQY	0,6118
B07	HLA-B*35:57	892	AALQIPFAM	0,5789
B07	HLA-B*35:57	895	QIPFAMQMAY	0,6098
B07	HLA-B*35:57	896	IPFAMQMAY	0,9872
B07	HLA-B*35:57	898	FAMQMAYRF	0,7448
B07	HLA-B*35:57	1052	FPQSAPHGVVF	0,8504
B07	HLA-B*35:57	1054	QSAPHGVVF	0,6307
B07	HLA-B*35:57	1095	FVSNGTHWF	0,5997
B07	HLA-B*35:61	24	LPPAYTNSF	0,797
B07	HLA-B*35:61	56	LPFFSNVTW	0,7097
B07	HLA-B*35:61	84	LPFNDGVY	0,6312
B07	HLA-B*35:61	84	LPFNDGVYF	0,9511
B07	HLA-B*35:61	162	SANNCTFEY	0,5112
B07	HLA-B*35:61	229	LPIGINITRF	0,6418
B07	HLA-B*35:61	321	QPTESIVRF	0,8721
B07	HLA-B*35:61	343	NATRFASVY	0,5359
B07	HLA-B*35:61	664	IPIGAGICASY	0,6771
B07	HLA-B*35:61	687	VASQSIIAY	0,8861
B07	HLA-B*35:61	699	LGAENSVAY	0,6356
B07	HLA-B*35:61	861	LPPLLTDEM	0,5504
B07	HLA-B*35:61	896	IPFAMQMAY	0,9572
B07	HLA-B*35:61	1052	FPQSAPHGVVF	0,787
B07	HLA-B*39:10	24	LPPAYTNSF	0,7059
B07	HLA-B*39:10	38	YPDKVFRSSV	0,6561
B07	HLA-B*39:10	38	YPDKVFRSSVL	0,6644
B07	HLA-B*39:10	84	LPFNDGVYF	0,7911
B07	HLA-B*39:10	208	TPINLVRDL	0,8013
B07	HLA-B*39:10	216	LPQGFSAL	0,7184
B07	HLA-B*39:10	229	LPIGINITRF	0,5061
B07	HLA-B*39:10	506	QPYRVVVL	0,6651
B07	HLA-B*39:10	714	IPTNFTISV	0,8392
B07	HLA-B*39:10	861	LPPLLTDEM	0,6235
B07	HLA-B*39:10	869	MIAQYTSAL	0,6143
B07	HLA-B*39:10	1052	FPQSAPHGV	0,8631
B07	HLA-B*39:10	1052	FPQSAPHGVV	0,603
B07	HLA-B*39:10	1052	FPQSAPHGVVF	0,7108
B07	HLA-B*39:10	1262	EPVLKGVKL	0,8325
B07	HLA-B*39:16	24	LPPAYTNSF	0,7059
B07	HLA-B*39:16	38	YPDKVFRSSV	0,6561
B07	HLA-B*39:16	38	YPDKVFRSSVL	0,6644
B07	HLA-B*39:16	84	LPFNDGVYF	0,7911
B07	HLA-B*39:16	208	TPINLVRDL	0,8013
B07	HLA-B*39:16	216	LPQGFSAL	0,7184

B07	HLA-B*39:16	229	LPIGINITRF	0,5061
B07	HLA-B*39:16	506	QPYRVVVL	0,6651
B07	HLA-B*39:16	714	IPTNFTISV	0,8392
B07	HLA-B*39:16	861	LPPLLTDEM	0,6235
B07	HLA-B*39:16	869	MIAQYTSAL	0,6143
B07	HLA-B*39:16	1052	FPQSAPHGV	0,8631
B07	HLA-B*39:16	1052	FPQSAPHGVV	0,603
B07	HLA-B*39:16	1052	FPQSAPHGVVF	0,7108
B07	HLA-B*39:16	1262	EPVLKGVKL	0,8325
B07	HLA-B*42:01	24	LPPAYTNSF	0,7878
B07	HLA-B*42:01	38	YPDKVFRSSV	0,6536
B07	HLA-B*42:01	84	LPFNDGVYF	0,7423
B07	HLA-B*42:01	208	TPINLVRDL	0,869
B07	HLA-B*42:01	216	LPQGFSAL	0,7808
B07	HLA-B*42:01	229	LPIGINITRF	0,5486
B07	HLA-B*42:01	506	QPYRVVVL	0,8243
B07	HLA-B*42:01	526	GPKKSTNLV	0,6419
B07	HLA-B*42:01	620	VPVAIHADQL	0,633
B07	HLA-B*42:01	714	IPTNFTISV	0,8435
B07	HLA-B*42:01	869	MIAQYTSAL	0,5968
B07	HLA-B*42:01	1052	FPQSAPHGV	0,7467
B07	HLA-B*42:01	1052	FPQSAPHGVVF	0,6491
B07	HLA-B*42:01	1056	APHGVVFL	0,7492
B07	HLA-B*42:01	1261	SEPVLKGVKL	0,7228
B07	HLA-B*42:01	1262	EPVLKGVKL	0,831
B07	HLA-B*42:04	38	YPDKVFRSSV	0,7244
B07	HLA-B*42:04	208	TPINLVRDL	0,7004
B07	HLA-B*42:04	216	LPQGFSAL	0,6226
B07	HLA-B*42:04	462	KPFERDIST	0,5873
B07	HLA-B*42:04	506	QPYRVVVL	0,6967
B07	HLA-B*42:04	526	GPKKSTNLV	0,662
B07	HLA-B*42:04	588	TPCSFGGVSV	0,5412
B07	HLA-B*42:04	664	IPIGAGICA	0,5648
B07	HLA-B*42:04	680	SPRRARSVA	0,6117
B07	HLA-B*42:04	714	IPTNFTISV	0,8825
B07	HLA-B*42:04	1052	FPQSAPHGV	0,8239
B07	HLA-B*42:04	1052	FPQSAPHGVV	0,6478
B07	HLA-B*42:04	1089	FPREGVFV	0,655
B07	HLA-B*42:04	1089	FPREGVFVS	0,5819
B07	HLA-B*42:05	24	LPPAYTNSF	0,7878
B07	HLA-B*42:05	38	YPDKVFRSSV	0,6536
B07	HLA-B*42:05	84	LPFNDGVYF	0,7423
B07	HLA-B*42:05	208	TPINLVRDL	0,869
B07	HLA-B*42:05	216	LPQGFSAL	0,7808
B07	HLA-B*42:05	229	LPIGINITRF	0,5486
B07	HLA-B*42:05	506	QPYRVVVL	0,8243
B07	HLA-B*42:05	526	GPKKSTNLV	0,6419
B07	HLA-B*42:05	620	VPVAIHADQL	0,633
B07	HLA-B*42:05	714	IPTNFTISV	0,8435
B07	HLA-B*42:05	869	MIAQYTSAL	0,5968

B07	HLA-B*42:05	1052	FPQSAPHGV	0,7467
B07	HLA-B*42:05	1052	FPQSAPHGVVF	0,6491
B07	HLA-B*42:05	1056	APHGVVFL	0,7492
B07	HLA-B*42:05	1261	SEPVLKGVKL	0,7228
B07	HLA-B*42:05	1262	EPVLKGVKL	0,831
B07	HLA-B*51:01	712	IAIPTNFTI	0,8936
B07	HLA-B*51:01	714	IPTNFTISV	0,9219
B07	HLA-B*51:02	712	IAIPTNFTI	0,9071
B07	HLA-B*51:02	714	IPTNFTISV	0,9166
B07	HLA-B*51:02	923	IANQFNSAI	0,7018
B07	HLA-B*51:02	1052	FPQSAPHGV	0,7998
B07	HLA-B*51:02	1052	FPQSAPHGVV	0,6058
B07	HLA-B*51:05	425	LPDDFTGCVI	0,5315
B07	HLA-B*51:09	712	IAIPTNFTI	0,7984
B07	HLA-B*51:09	714	IPTNFTISV	0,7885
B07	HLA-B*51:16	712	IAIPTNFTI	0,8496
B07	HLA-B*51:16	714	IPTNFTISV	0,882
B07	HLA-B*51:17	712	IAIPTNFTI	0,8936
B07	HLA-B*51:17	714	IPTNFTISV	0,9219
B07	HLA-B*51:18	712	IAIPTNFTI	0,8936
B07	HLA-B*51:18	714	IPTNFTISV	0,9219
B07	HLA-B*51:19	56	LPFFSNVTW	0,5994
B07	HLA-B*51:19	84	LPFNDGVYF	0,7163
B07	HLA-B*51:19	712	IAIPTNFTI	0,8629
B07	HLA-B*51:19	714	IPTNFTISV	0,8404
B07	HLA-B*51:21	712	IAIPTNFTI	0,8586
B07	HLA-B*51:21	714	IPTNFTISV	0,8761
B07	HLA-B*51:24	712	IAIPTNFTI	0,8936
B07	HLA-B*51:24	714	IPTNFTISV	0,9219
B07	HLA-B*51:26	712	IAIPTNFTI	0,8936
B07	HLA-B*51:26	714	IPTNFTISV	0,9219
B07	HLA-B*51:28	712	IAIPTNFTI	0,8936
B07	HLA-B*51:28	714	IPTNFTISV	0,9219
B07	HLA-B*51:29	425	LPDDFTGCVI	0,6074
B07	HLA-B*51:29	714	IPTNFTISV	0,8847
B07	HLA-B*51:30	712	IAIPTNFTI	0,8936
B07	HLA-B*51:30	714	IPTNFTISV	0,9219
B07	HLA-B*51:31	712	IAIPTNFTI	0,8324
B07	HLA-B*51:31	714	IPTNFTISV	0,8262
B07	HLA-B*51:32	712	IAIPTNFTI	0,8936
B07	HLA-B*51:32	714	IPTNFTISV	0,9219
B07	HLA-B*51:33	712	IAIPTNFTI	0,8936
B07	HLA-B*51:33	714	IPTNFTISV	0,9219
B07	HLA-B*51:34	712	IAIPTNFTI	0,8619
B07	HLA-B*51:34	714	IPTNFTISV	0,8627
B07	HLA-B*51:34	1052	FPQSAPHGVV	0,5175
B07	HLA-B*51:35	712	IAIPTNFTI	0,8936
B07	HLA-B*51:35	714	IPTNFTISV	0,9219
B07	HLA-B*51:36	714	IPTNFTISV	0,8244
B07	HLA-B*51:36	1052	FPQSAPHGVV	0,5349

B07	HLA-B*51:38	712	IAIPTNFTI	0,8936
B07	HLA-B*51:38	714	IPTNFTISV	0,9219
B07	HLA-B*53:01	24	LPPAYTNSF	0,7097
B07	HLA-B*53:01	55	FLPFFSNVTW	0,7688
B07	HLA-B*53:01	56	LPFFSNVTW	0,9556
B07	HLA-B*53:01	56	LPFFSNVTWF	0,502
B07	HLA-B*53:01	83	VLPFNDGVYF	0,7116
B07	HLA-B*53:01	84	LPFNDGVYF	0,967
B07	HLA-B*53:01	229	LPIGINITRF	0,8328
B07	HLA-B*53:01	250	TPGDSSSGW	0,8069
B07	HLA-B*53:01	320	VQPTESIVRF	0,5847
B07	HLA-B*53:01	321	QPTESIVRF	0,9157
B07	HLA-B*53:01	625	HADQLTPTW	0,9493
B07	HLA-B*53:01	714	IPTNFTISV	0,5509
B07	HLA-B*53:01	878	LAGTITSGW	0,6015
B07	HLA-B*53:01	896	IPFAMQMAY	0,8495
B07	HLA-B*53:01	898	FAMQMAYRF	0,7049
B07	HLA-B*53:01	1052	FPQSAPHGV	0,6048
B07	HLA-B*53:01	1052	FPQSAPHGVVF	0,6896
B07	HLA-B*53:02	24	LPPAYTNSF	0,6754
B07	HLA-B*53:02	53	DLFLPFFSNVTW	0,5156
B07	HLA-B*53:02	55	FLPFFSNVTW	0,7377
B07	HLA-B*53:02	56	LPFFSNVTW	0,9419
B07	HLA-B*53:02	83	VLPFNDGVYF	0,5685
B07	HLA-B*53:02	84	LPFNDGVYF	0,927
B07	HLA-B*53:02	229	LPIGINITRF	0,7712
B07	HLA-B*53:02	321	QPTESIVRF	0,8542
B07	HLA-B*53:02	625	HADQLTPTW	0,8778
B07	HLA-B*53:02	714	IPTNFTISV	0,5046
B07	HLA-B*53:02	896	IPFAMQMAY	0,8286
B07	HLA-B*53:02	898	FAMQMAYRF	0,7523
B07	HLA-B*53:02	1052	FPQSAPHGVVF	0,6331
B07	HLA-B*53:06	24	LPPAYTNSF	0,7435
B07	HLA-B*53:06	55	FLPFFSNVTW	0,611
B07	HLA-B*53:06	56	LPFFSNVTW	0,897
B07	HLA-B*53:06	84	LPFNDGVYF	0,9269
B07	HLA-B*53:06	229	LPIGINITRF	0,7787
B07	HLA-B*53:06	321	QPTESIVRF	0,8336
B07	HLA-B*53:06	490	FPLQSYGF	0,5333
B07	HLA-B*53:06	625	HADQLTPTW	0,7925
B07	HLA-B*53:06	896	IPFAMQMAY	0,8651
B07	HLA-B*53:06	898	FAMQMAYRF	0,7249
B07	HLA-B*53:06	1052	FPQSAPHGVVF	0,6456
B07	HLA-B*53:06	1095	FVSNGTHWF	0,5268
B07	HLA-B*53:08	24	LPPAYTNSF	0,7478
B07	HLA-B*53:08	55	FLPFFSNVTW	0,634
B07	HLA-B*53:08	56	LPFFSNVTW	0,9209
B07	HLA-B*53:08	83	VLPFNDGVYF	0,713
B07	HLA-B*53:08	84	LPFNDGVYF	0,9579
B07	HLA-B*53:08	229	LPIGINITRF	0,8211

B07	HLA-B*53:08	250	TPGDSSSGW	0,701
B07	HLA-B*53:08	321	QPTESIVRF	0,8829
B07	HLA-B*53:08	625	HADQLTPTW	0,8712
B07	HLA-B*53:08	687	VASQSIIAY	0,7141
B07	HLA-B*53:08	712	IAIPTNFTI	0,5344
B07	HLA-B*53:08	714	IPTNFTISV	0,5417
B07	HLA-B*53:08	878	LAGTITSGW	0,576
B07	HLA-B*53:08	896	IPFAMQMAY	0,8758
B07	HLA-B*53:08	898	FAMQMAYRF	0,6831
B07	HLA-B*53:08	1052	FPQSAPHGVVF	0,6472
B07	HLA-B*53:08	1095	FVSNGTHWF	0,6097
B07	HLA-B*53:10	24	LPPAYTNSF	0,7097
B07	HLA-B*53:10	55	FLPFFSNVTW	0,7688
B07	HLA-B*53:10	56	LPFFSNVTW	0,9556
B07	HLA-B*53:10	56	LPFFSNVTWF	0,502
B07	HLA-B*53:10	83	VLPFNDGVYF	0,7116
B07	HLA-B*53:10	84	LPFNDGVYF	0,967
B07	HLA-B*53:10	229	LPIGINITRF	0,8328
B07	HLA-B*53:10	250	TPGDSSSGW	0,8069
B07	HLA-B*53:10	320	VQPTESIVRF	0,5847
B07	HLA-B*53:10	321	QPTESIVRF	0,9157
B07	HLA-B*53:10	625	HADQLTPTW	0,9493
B07	HLA-B*53:10	714	IPTNFTISV	0,5509
B07	HLA-B*53:10	878	LAGTITSGW	0,6015
B07	HLA-B*53:10	896	IPFAMQMAY	0,8495
B07	HLA-B*53:10	898	FAMQMAYRF	0,7049
B07	HLA-B*53:10	1052	FPQSAPHGV	0,6048
B07	HLA-B*53:10	1052	FPQSAPHGVVF	0,6896
B07	HLA-B*54:01	38	YPDKVFRSS	0,5631
B07	HLA-B*54:01	38	YPDKVFRSSV	0,551
B07	HLA-B*54:01	84	LPFNDGVYFA	0,8147
B07	HLA-B*54:01	336	CPFGEVFNA	0,675
B07	HLA-B*54:01	506	QPYRVVVLS	0,517
B07	HLA-B*54:01	664	IPIGAGICA	0,7761
B07	HLA-B*54:01	714	IPTNFTISV	0,8556
B07	HLA-B*54:01	1052	FPQSAPHGV	0,7788
B07	HLA-B*54:01	1052	FPQSAPHGVV	0,5637
B07	HLA-B*54:01	1089	FPREGVFV	0,581
B07	HLA-B*54:01	1089	FPREGVFVS	0,826
B07	HLA-B*54:03	38	YPDKVFRSS	0,5065
B07	HLA-B*54:03	84	LPFNDGVYFA	0,7103
B07	HLA-B*54:03	336	CPFGEVFNA	0,5864
B07	HLA-B*54:03	664	IPIGAGICA	0,6982
B07	HLA-B*54:03	714	IPTNFTISV	0,759
B07	HLA-B*54:03	1052	FPQSAPHGV	0,7099
B07	HLA-B*54:03	1089	FPREGVFVS	0,7861
B07	HLA-B*54:04	38	YPDKVFRSSV	0,6067
B07	HLA-B*54:04	84	LPFNDGVYF	0,6325
B07	HLA-B*54:04	84	LPFNDGVYFA	0,7208
B07	HLA-B*54:04	336	CPFGEVFNA	0,5184

B07	HLA-B*54:04	664	IPIGAGICA	0,7162
B07	HLA-B*54:04	714	IPTNFTISV	0,8759
B07	HLA-B*54:04	1052	FPQSAPHGV	0,8116
B07	HLA-B*54:04	1052	FPQSAPHGVV	0,6221
B07	HLA-B*54:04	1089	FPREGVFV	0,626
B07	HLA-B*54:04	1089	FPREGVFVS	0,7032
B07	HLA-B*54:06	24	LPPAYTNSF	0,5438
B07	HLA-B*54:06	84	LPFNDGVYF	0,7179
B07	HLA-B*54:06	271	QPRTFLLKY	0,5428
B07	HLA-B*54:06	687	VASQSIIAY	0,5887
B07	HLA-B*54:06	896	IPFAMQMAY	0,8139
B07	HLA-B*54:07	38	YPDKVFRSS	0,5631
B07	HLA-B*54:07	38	YPDKVFRSSV	0,551
B07	HLA-B*54:07	84	LPFNDGVYFA	0,8147
B07	HLA-B*54:07	336	CPFGEVFNA	0,675
B07	HLA-B*54:07	506	QPYRVVVLS	0,517
B07	HLA-B*54:07	664	IPIGAGICA	0,7761
B07	HLA-B*54:07	714	IPTNFTISV	0,8556
B07	HLA-B*54:07	1052	FPQSAPHGV	0,7788
B07	HLA-B*54:07	1052	FPQSAPHGVV	0,5637
B07	HLA-B*54:07	1089	FPREGVFV	0,581
B07	HLA-B*54:07	1089	FPREGVFVS	0,826
B07	HLA-B*55:01	664	IPIGAGICA	0,566
B07	HLA-B*55:01	680	SPRRARSVA	0,6445
B07	HLA-B*55:01	714	IPTNFTISV	0,699
B07	HLA-B*55:01	1052	FPQSAPHGV	0,551
B07	HLA-B*55:01	1089	FPREGVFVS	0,6525
B07	HLA-B*55:02	38	YPDKVFRSS	0,6589
B07	HLA-B*55:02	38	YPDKVFRSSV	0,5249
B07	HLA-B*55:02	84	LPFNDGVYF	0,6389
B07	HLA-B*55:02	84	LPFNDGVYFA	0,6565
B07	HLA-B*55:02	336	CPFGEVFNA	0,5229
B07	HLA-B*55:02	462	KPFERDIST	0,6378
B07	HLA-B*55:02	506	QPYRVVVLS	0,527
B07	HLA-B*55:02	664	IPIGAGICA	0,7531
B07	HLA-B*55:02	714	IPTNFTISV	0,8516
B07	HLA-B*55:02	896	IPFAMQMAY	0,5572
B07	HLA-B*55:02	1052	FPQSAPHGV	0,758
B07	HLA-B*55:02	1052	FPQSAPHGVV	0,5174
B07	HLA-B*55:02	1089	FPREGVFV	0,5413
B07	HLA-B*55:02	1089	FPREGVFVS	0,8144
B07	HLA-B*55:03	714	IPTNFTISV	0,6188
B07	HLA-B*55:03	1052	FPQSAPHGV	0,5151
B07	HLA-B*55:03	1089	FPREGVFVS	0,531
B07	HLA-B*55:04	24	LPPAYTNSF	0,7178
B07	HLA-B*55:04	38	YPDKVFRSSV	0,6017
B07	HLA-B*55:04	56	LPFFSNVTW	0,5466
B07	HLA-B*55:04	83	VLPFNDGVYF	0,5131
B07	HLA-B*55:04	84	LPFNDGVYF	0,8753
B07	HLA-B*55:04	208	TPINLVRDL	0,8684

B07	HLA-B*55:04	216	LPQGFSAL	0,6991
B07	HLA-B*55:04	229	LPIGINITRF	0,7322
B07	HLA-B*55:04	321	QPTESIVRF	0,7159
B07	HLA-B*55:04	462	KPFERDIST	0,5424
B07	HLA-B*55:04	506	QPYRVVVL	0,7157
B07	HLA-B*55:04	620	VPVAIHADQL	0,6147
B07	HLA-B*55:04	714	IPTNFTISV	0,896
B07	HLA-B*55:04	869	MIAQYTSAL	0,5791
B07	HLA-B*55:04	892	AALQIPFAM	0,5124
B07	HLA-B*55:04	896	IPFAMQMAY	0,6175
B07	HLA-B*55:04	1052	FPQSAPHGV	0,8055
B07	HLA-B*55:04	1052	FPQSAPHGVVF	0,6991
B07	HLA-B*55:04	1056	APHGVVFL	0,6967
B07	HLA-B*55:04	1089	FPREGVFVS	0,5475
B07	HLA-B*55:04	1261	SEPVLKGVKL	0,5737
B07	HLA-B*55:04	1262	EPVLKGVKL	0,7445
B07	HLA-B*55:05	664	IPIGAGICA	0,566
B07	HLA-B*55:05	680	SPRRARSVA	0,6445
B07	HLA-B*55:05	714	IPTNFTISV	0,699
B07	HLA-B*55:05	1052	FPQSAPHGV	0,551
B07	HLA-B*55:05	1089	FPREGVFVS	0,6525
B07	HLA-B*55:07	38	YPDKVFRSS	0,5631
B07	HLA-B*55:07	38	YPDKVFRSSV	0,551
B07	HLA-B*55:07	84	LPFNDGVYFA	0,8147
B07	HLA-B*55:07	336	CPFGEVFNA	0,675
B07	HLA-B*55:07	506	QPYRVVVLS	0,517
B07	HLA-B*55:07	664	IPIGAGICA	0,7761
B07	HLA-B*55:07	714	IPTNFTISV	0,8556
B07	HLA-B*55:07	1052	FPQSAPHGV	0,7788
B07	HLA-B*55:07	1052	FPQSAPHGVV	0,5637
B07	HLA-B*55:07	1089	FPREGVFV	0,581
B07	HLA-B*55:07	1089	FPREGVFVS	0,826
B07	HLA-B*55:09	38	YPDKVFRSSV	0,6045
B07	HLA-B*55:09	664	IPIGAGICA	0,5924
B07	HLA-B*55:09	680	SPRRARSVA	0,7242
B07	HLA-B*55:09	714	IPTNFTISV	0,7624
B07	HLA-B*55:09	1052	FPQSAPHGV	0,5713
B07	HLA-B*55:09	1089	FPREGVFVS	0,6196
B07	HLA-B*55:10	38	YPDKVFRSS	0,6429
B07	HLA-B*55:10	84	LPFNDGVYF	0,6586
B07	HLA-B*55:10	84	LPFNDGVYFA	0,567
B07	HLA-B*55:10	462	KPFERDIST	0,64
B07	HLA-B*55:10	664	IPIGAGICA	0,7168
B07	HLA-B*55:10	714	IPTNFTISV	0,8293
B07	HLA-B*55:10	896	IPFAMQMAY	0,5528
B07	HLA-B*55:10	1052	FPQSAPHGV	0,723
B07	HLA-B*55:10	1089	FPREGVFV	0,5148
B07	HLA-B*55:10	1089	FPREGVFVS	0,776
B07	HLA-B*55:15	664	IPIGAGICA	0,566
B07	HLA-B*55:15	680	SPRRARSVA	0,6445

B07	HLA-B*55:15	714	IPTNFTISV	0,699
B07	HLA-B*55:15	1052	FPQSAPHGV	0,551
B07	HLA-B*55:15	1089	FPREGVFVS	0,6525
B07	HLA-B*55:17	462	KPFERDIST	0,5535
B07	HLA-B*55:17	664	IPIGAGICA	0,5355
B07	HLA-B*55:17	680	SPRRARSVA	0,6844
B07	HLA-B*55:17	714	IPTNFTISV	0,7936
B07	HLA-B*55:17	1052	FPQSAPHGV	0,6104
B07	HLA-B*55:17	1089	FPREGVFVS	0,5754
B07	HLA-B*55:19	38	YPDKVFRSS	0,6589
B07	HLA-B*55:19	38	YPDKVFRSSV	0,5249
B07	HLA-B*55:19	84	LPFNDGVYF	0,6389
B07	HLA-B*55:19	84	LPFNDGVYFA	0,6565
B07	HLA-B*55:19	336	CPFGEVFNA	0,5229
B07	HLA-B*55:19	462	KPFERDIST	0,6378
B07	HLA-B*55:19	506	QPYRVVVLS	0,527
B07	HLA-B*55:19	664	IPIGAGICA	0,7531
B07	HLA-B*55:19	714	IPTNFTISV	0,8516
B07	HLA-B*55:19	896	IPFAMQMAY	0,5572
B07	HLA-B*55:19	1052	FPQSAPHGV	0,758
B07	HLA-B*55:19	1052	FPQSAPHGVV	0,5174
B07	HLA-B*55:19	1089	FPREGVFV	0,5413
B07	HLA-B*55:19	1089	FPREGVFVS	0,8144
B07	HLA-B*56:01	38	YPDKVFRSS	0,5594
B07	HLA-B*56:01	84	LPFNDGVYF	0,6481
B07	HLA-B*56:01	84	LPFNDGVYFA	0,5533
B07	HLA-B*56:01	664	IPIGAGICA	0,6738
B07	HLA-B*56:01	714	IPTNFTISV	0,7754
B07	HLA-B*56:01	896	IPFAMQMAY	0,5446
B07	HLA-B*56:01	1052	FPQSAPHGV	0,6791
B07	HLA-B*56:01	1089	FPREGVFVS	0,7781
B07	HLA-B*56:03	84	LPFNDGVYF	0,65
B07	HLA-B*56:03	271	QPRTFLLKY	0,5652
B07	HLA-B*56:03	687	VASQSIIAY	0,5692
B07	HLA-B*56:03	896	IPFAMQMAY	0,7714
B07	HLA-B*56:05	664	IPIGAGICA	0,503
B07	HLA-B*56:05	680	SPRRARSVA	0,5529
B07	HLA-B*56:05	714	IPTNFTISV	0,7632
B07	HLA-B*56:05	896	IPFAMQMAY	0,5665
B07	HLA-B*56:05	1052	FPQSAPHGV	0,6122
B07	HLA-B*56:05	1089	FPREGVFV	0,5865
B07	HLA-B*56:05	1089	FPREGVFVS	0,6335
B07	HLA-B*56:13	38	YPDKVFRSS	0,5603
B07	HLA-B*56:13	38	YPDKVFRSSV	0,5158
B07	HLA-B*56:13	664	IPIGAGICA	0,6238
B07	HLA-B*56:13	680	SPRRARSVA	0,513
B07	HLA-B*56:13	714	IPTNFTISV	0,769
B07	HLA-B*56:13	1052	FPQSAPHGV	0,731
B07	HLA-B*56:13	1052	FPQSAPHGVV	0,5165
B07	HLA-B*56:13	1089	FPREGVFV	0,5099

B07	HLA-B*56:13	1089	FPREGVFVS	0,715
B07	HLA-B*56:15	84	LPFNDGVYF	0,7262
B07	HLA-B*56:15	664	IPIGAGICA	0,6282
B07	HLA-B*56:15	714	IPTNFTISV	0,8617
B07	HLA-B*56:15	1052	FPQSAPHGV	0,7753
B07	HLA-B*56:15	1052	FPQSAPHGVV	0,5072
B07	HLA-B*56:15	1089	FPREGVFVS	0,685
B07	HLA-B*56:16	38	YPDKVFRSS	0,5423
B07	HLA-B*56:16	84	LPFNDGVYF	0,6574
B07	HLA-B*56:16	664	IPIGAGICA	0,6319
B07	HLA-B*56:16	714	IPTNFTISV	0,7528
B07	HLA-B*56:16	896	IPFAMQMAY	0,5261
B07	HLA-B*56:16	1052	FPQSAPHGV	0,6463
B07	HLA-B*56:16	1089	FPREGVFVS	0,7472
B07	HLA-B*67:01	24	LPPAYTNSF	0,6526
B07	HLA-B*67:01	38	YPDKVFRSSV	0,5867
B07	HLA-B*67:01	38	YPDKVFRSSVL	0,5124
B07	HLA-B*67:01	84	LPFNDGVYF	0,8292
B07	HLA-B*67:01	208	TPINLVRDL	0,7949
B07	HLA-B*67:01	216	LPQGFSAL	0,5784
B07	HLA-B*67:01	229	LPIGINITRF	0,5824
B07	HLA-B*67:01	321	QPTESIVRF	0,6782
B07	HLA-B*67:01	506	QPYRVVVL	0,5066
B07	HLA-B*67:01	714	IPTNFTISV	0,8295
B07	HLA-B*67:01	861	LPPLLTDEM	0,5088
B07	HLA-B*67:01	896	IPFAMQMAY	0,5126
B07	HLA-B*67:01	1052	FPQSAPHGV	0,7937
B07	HLA-B*67:01	1052	FPQSAPHGVVF	0,6798
B07	HLA-B*67:01	1262	EPVLKGVKL	0,7213
B07	HLA-B*78:01	714	IPTNFTISV	0,7595
B07	HLA-B*78:01	1052	FPQSAPHGV	0,6988
B07	HLA-B*78:01	1089	FPREGVFV	0,6628
B07	HLA-B*78:01	1089	FPREGVFVS	0,5573
B07	HLA-B*78:02	714	IPTNFTISV	0,7684
B07	HLA-B*78:04	714	IPTNFTISV	0,7551
B07	HLA-B*78:04	1089	FPREGVFVS	0,5305
B08	HLA-B*08:01	17	NLTTRTQL	0,5899
B08	HLA-B*08:01	233	INITRFQTL	0,7017
B08	HLA-B*08:01	234	NITRFQTL	0,7511
B08	HLA-B*08:01	241	LLALHRSYL	0,5553
B08	HLA-B*08:01	269	YLQPRTFL	0,7406
B08	HLA-B*08:01	269	YLQPRTFLL	0,7867
B08	HLA-B*08:01	506	QPYRVVVL	0,766
B08	HLA-B*08:01	821	LLFNKVTL	0,6195
B08	HLA-B*08:01	996	LITGRLQSL	0,5644
B08	HLA-B*08:07	233	INITRFQTL	0,6349
B08	HLA-B*08:07	234	NITRFQTL	0,6414
B08	HLA-B*08:07	269	YLQPRTFL	0,6444
B08	HLA-B*08:07	269	YLQPRTFLL	0,7258
B08	HLA-B*08:07	506	QPYRVVVL	0,7156

B08	HLA-B*08:07	821	LLFNKVTL	0,5358
B08	HLA-B*08:07	996	LITGRLQSL	0,5316
B08	HLA-B*08:09	269	YLQPRTFLL	0,5745
B08	HLA-B*08:11	17	NLTTRTQL	0,5719
B08	HLA-B*08:11	233	INITRFQTL	0,6926
B08	HLA-B*08:11	234	NITRFQTL	0,7381
B08	HLA-B*08:11	241	LLALHRSYL	0,5524
B08	HLA-B*08:11	269	YLQPRTFL	0,7196
B08	HLA-B*08:11	269	YLQPRTFLL	0,7806
B08	HLA-B*08:11	506	QPYRVVVL	0,7665
B08	HLA-B*08:11	821	LLFNKVTL	0,5958
B08	HLA-B*08:11	996	LITGRLQSL	0,5708
B08	HLA-B*08:13	233	INITRFQTL	0,5821
B08	HLA-B*08:13	269	YLQPRTFL	0,5148
B08	HLA-B*08:13	269	YLQPRTFLL	0,6693
B08	HLA-B*08:13	506	QPYRVVVL	0,6559
B08	HLA-B*08:15	233	INITRFQTL	0,6284
B08	HLA-B*08:15	234	NITRFQTL	0,5714
B08	HLA-B*08:15	269	YLQPRTFL	0,6627
B08	HLA-B*08:15	269	YLQPRTFLL	0,7736
B08	HLA-B*08:15	821	LLFNKVTL	0,507
B08	HLA-B*08:18	17	NLTTRTQL	0,5899
B08	HLA-B*08:18	233	INITRFQTL	0,7017
B08	HLA-B*08:18	234	NITRFQTL	0,7511
B08	HLA-B*08:18	241	LLALHRSYL	0,5553
B08	HLA-B*08:18	269	YLQPRTFL	0,7406
B08	HLA-B*08:18	269	YLQPRTFLL	0,7867
B08	HLA-B*08:18	506	QPYRVVVL	0,766
B08	HLA-B*08:18	821	LLFNKVTL	0,6195
B08	HLA-B*08:18	996	LITGRLQSL	0,5644
B08	HLA-B*08:20	241	LLALHRSYL	0,5123
B08	HLA-B*08:20	269	YLQPRTFL	0,5485
B08	HLA-B*08:20	269	YLQPRTFLL	0,6503
B08	HLA-B*08:20	869	MIAQYTSAL	0,5115
B08	HLA-B*08:21	233	INITRFQTL	0,5468
B08	HLA-B*08:21	234	NITRFQTL	0,598
B08	HLA-B*08:21	269	YLQPRTFL	0,5663
B08	HLA-B*08:21	269	YLQPRTFLL	0,6617
B08	HLA-B*08:21	506	QPYRVVVL	0,6419
B08	HLA-B*08:22	17	NLTTRTQL	0,5899
B08	HLA-B*08:22	233	INITRFQTL	0,7017
B08	HLA-B*08:22	234	NITRFQTL	0,7511
B08	HLA-B*08:22	241	LLALHRSYL	0,5553
B08	HLA-B*08:22	269	YLQPRTFL	0,7406
B08	HLA-B*08:22	269	YLQPRTFLL	0,7867
B08	HLA-B*08:22	506	QPYRVVVL	0,766
B08	HLA-B*08:22	821	LLFNKVTL	0,6195
B08	HLA-B*08:22	996	LITGRLQSL	0,5644
B08	HLA-B*08:23	269	YLQPRTFL	0,5575
B08	HLA-B*08:23	269	YLQPRTFLL	0,6446

B08	HLA-B*08:23	821	LLFNKVTL	0,5015
B08	HLA-B*08:24	17	NLTTRTQL	0,5899
B08	HLA-B*08:24	233	INITRFQTL	0,7017
B08	HLA-B*08:24	234	NITRFQTL	0,7511
B08	HLA-B*08:24	241	LLALHRSYL	0,5553
B08	HLA-B*08:24	269	YLQPRTFL	0,7406
B08	HLA-B*08:24	269	YLQPRTFLL	0,7867
B08	HLA-B*08:24	506	QPYRVVVL	0,766
B08	HLA-B*08:24	821	LLFNKVTL	0,6195
B08	HLA-B*08:24	996	LITGRLQSL	0,5644
B08	HLA-B*08:25	269	YLQPRTFLL	0,5151
B27	HLA-B*14:01	236	TRFQTLLAL	0,8018
B27	HLA-B*14:01	453	YRLFRKSNL	0,6967
B27	HLA-B*14:02	236	TRFQTLLAL	0,8018
B27	HLA-B*14:02	453	YRLFRKSNL	0,6967
B27	HLA-B*14:03	236	TRFQTLLAL	0,6892
B27	HLA-B*14:03	453	YRLFRKSNL	0,576
B27	HLA-B*14:06	236	TRFQTLLAL	0,8675
B27	HLA-B*14:06	764	NRALTGIAV	0,7167
B27	HLA-B*15:03	20	TRTQLPPAY	0,7211
B27	HLA-B*15:03	22	TQLPPAYTNSF	0,6455
B27	HLA-B*15:03	35	GVYYPDKVF	0,6663
B27	HLA-B*15:03	47	VLHSTQDLF	0,5243
B27	HLA-B*15:03	77	KRFDNPVLPF	0,8296
B27	HLA-B*15:03	184	GNFKNLREF	0,6499
B27	HLA-B*15:03	186	FKNLREFVF	0,5195
B27	HLA-B*15:03	192	FVFKNIDGY	0,5282
B27	HLA-B*15:03	212	LVRDLPQGF	0,576
B27	HLA-B*15:03	236	TRFQTLLAL	0,608
B27	HLA-B*15:03	240	TLLALHRSY	0,6413
B27	HLA-B*15:03	261	GAAAYYVGY	0,5148
B27	HLA-B*15:03	267	VGYLQPRTF	0,643
B27	HLA-B*15:03	320	VQPTESIVRF	0,5258
B27	HLA-B*15:03	327	VRFPNITNL	0,8039
B27	HLA-B*15:03	339	GEVFNATRF	0,7209
B27	HLA-B*15:03	345	TRFASVYAW	0,6043
B27	HLA-B*15:03	366	SVLYNSASF	0,5032
B27	HLA-B*15:03	369	YNSASFSTF	0,7633
B27	HLA-B*15:03	408	RQIAPGQTG	0,5804
B27	HLA-B*15:03	413	GQTGKIADY	0,8037
B27	HLA-B*15:03	443	SKVGGNYNY	0,9112
B27	HLA-B*15:03	456	FRKSNLKPF	0,5235
B27	HLA-B*15:03	464	FERDISTEIY	0,635
B27	HLA-B*15:03	497	FQPTNGVGY	0,8242
B27	HLA-B*15:03	557	KKFLPFQQF	0,9568
B27	HLA-B*15:03	628	QLTPTWRVY	0,5065
B27	HLA-B*15:03	634	RVYSTGSNVF	0,7212
B27	HLA-B*15:03	687	VASQSIIAY	0,7907
B27	HLA-B*15:03	689	SQSIIAYTM	0,7655
B27	HLA-B*15:03	699	LGAENSVAY	0,5647

B27	HLA-B*15:03	710	NSIAIPTNF	0,5451
B27	HLA-B*15:03	789	YKTPPIKDF	0,8036
B27	HLA-B*15:03	794	IKDFGGFNF	0,7241
B27	HLA-B*15:03	815	RSFIEDLLF	0,624
B27	HLA-B*15:03	852	AQKFNGLTV	0,6121
B27	HLA-B*15:03	852	AQKFNGLTVL	0,5743
B27	HLA-B*15:03	853	QKFNGLTVL	0,7866
B27	HLA-B*15:03	880	GTITSGWTF	0,5063
B27	HLA-B*15:03	893	ALQIPFAMQM	0,5298
B27	HLA-B*15:03	894	LQIPFAMQM	0,9192
B27	HLA-B*15:03	919	NQKLIANQF	0,8596
B27	HLA-B*15:03	999	GRLQSLQTY	0,8486
B27	HLA-B*15:03	1044	GKGYHLMSF	0,7673
B27	HLA-B*15:03	1054	QSAPHGVVF	0,8355
B27	HLA-B*15:03	1087	AHFPREGVF	0,7921
B27	HLA-B*15:03	1113	QIITTDNTF	0,5046
B27	HLA-B*15:03	1130	IGIVNNTVY	0,5224
B27	HLA-B*15:03	1264	VLKGVKLHY	0,6224
B27	HLA-B*15:18	20	TRTQLPPAY	0,7269
B27	HLA-B*15:18	236	TRFQTLLAL	0,6174
B27	HLA-B*15:18	1087	AHFPREGVF	0,6545
B27	HLA-B*15:47	77	KRFDNPVLPF	0,5237
B27	HLA-B*15:47	339	GEVFNATRF	0,6636
B27	HLA-B*15:47	369	YNSASFSTF	0,5152
B27	HLA-B*15:47	413	GQTGKIADY	0,5603
B27	HLA-B*15:47	464	FERDISTEIY	0,5227
B27	HLA-B*15:47	557	KKFLPFQQF	0,7088
B27	HLA-B*15:47	687	VASQSIIAY	0,5883
B27	HLA-B*15:47	689	SQSIIAYTM	0,6903
B27	HLA-B*15:47	852	AQKFNGLTVL	0,503
B27	HLA-B*15:47	894	LQIPFAMQM	0,6781
B27	HLA-B*15:47	919	NQKLIANQF	0,6441
B27	HLA-B*15:47	1054	QSAPHGVVF	0,5973
B27	HLA-B*15:47	1087	AHFPREGVF	0,5178
B27	HLA-B*15:49	77	KRFDNPVLPF	0,5237
B27	HLA-B*15:49	339	GEVFNATRF	0,6636
B27	HLA-B*15:49	369	YNSASFSTF	0,5152
B27	HLA-B*15:49	413	GQTGKIADY	0,5603
B27	HLA-B*15:49	464	FERDISTEIY	0,5227
B27	HLA-B*15:49	557	KKFLPFQQF	0,7088
B27	HLA-B*15:49	687	VASQSIIAY	0,5883
B27	HLA-B*15:49	689	SQSIIAYTM	0,6903
B27	HLA-B*15:49	852	AQKFNGLTVL	0,503
B27	HLA-B*15:49	894	LQIPFAMQM	0,6781
B27	HLA-B*15:49	919	NQKLIANQF	0,6441
B27	HLA-B*15:49	1054	QSAPHGVVF	0,5973
B27	HLA-B*15:49	1087	AHFPREGVF	0,5178
B27	HLA-B*15:52	236	TRFQTLLAL	0,6881
B27	HLA-B*15:54	20	TRTQLPPAY	0,6536
B27	HLA-B*15:54	22	TQLPPAYTNSF	0,5407

B27	HLA-B*15:54	35	GVYYPDKVF	0,5415
B27	HLA-B*15:54	77	KRFDNPVLPF	0,707
B27	HLA-B*15:54	212	LVRDLPQGF	0,5164
B27	HLA-B*15:54	240	TLLALHRSY	0,5397
B27	HLA-B*15:54	320	VQPTESIVRF	0,5256
B27	HLA-B*15:54	339	GEVFNATRF	0,706
B27	HLA-B*15:54	369	YNSASFSTF	0,659
B27	HLA-B*15:54	408	RQIAPGQTG	0,5392
B27	HLA-B*15:54	413	GQTGKIADY	0,8083
B27	HLA-B*15:54	443	SKVGGNYNY	0,849
B27	HLA-B*15:54	456	FRKSNLKPF	0,5182
B27	HLA-B*15:54	464	FERDISTEIY	0,7035
B27	HLA-B*15:54	497	FQPTNGVGY	0,7908
B27	HLA-B*15:54	557	KKFLPFQQF	0,8533
B27	HLA-B*15:54	634	RVYSTGSNVF	0,6621
B27	HLA-B*15:54	687	VASQSIIAY	0,6625
B27	HLA-B*15:54	689	SQSIIAYTM	0,6728
B27	HLA-B*15:54	789	YKTPPIKDF	0,6416
B27	HLA-B*15:54	852	AQKFNGLTV	0,5786
B27	HLA-B*15:54	852	AQKFNGLTVL	0,5715
B27	HLA-B*15:54	853	QKFNGLTVL	0,5953
B27	HLA-B*15:54	894	LQIPFAMQM	0,7958
B27	HLA-B*15:54	919	NQKLIANQF	0,8585
B27	HLA-B*15:54	999	GRLQSLQTY	0,757
B27	HLA-B*15:54	1044	GKGYHLMSF	0,6503
B27	HLA-B*15:54	1054	QSAPHGVVF	0,7441
B27	HLA-B*15:54	1087	AHFPREGVF	0,6276
B27	HLA-B*15:54	1264	VLKGVKLHY	0,6417
B27	HLA-B*15:61	20	TRTQLPPAY	0,771
B27	HLA-B*15:61	35	GVYYPDKVF	0,5503
B27	HLA-B*15:61	77	KRFDNPVLPF	0,8652
B27	HLA-B*15:61	236	TRFQTLLAL	0,7603
B27	HLA-B*15:61	318	FRVQPTESI	0,5563
B27	HLA-B*15:61	327	VRFPNITNL	0,9057
B27	HLA-B*15:61	345	TRFASVYAW	0,6322
B27	HLA-B*15:61	369	YNSASFSTF	0,5292
B27	HLA-B*15:61	413	GQTGKIADY	0,6318
B27	HLA-B*15:61	443	SKVGGNYNY	0,8055
B27	HLA-B*15:61	456	FRKSNLKPF	0,5874
B27	HLA-B*15:61	497	FQPTNGVGY	0,6258
B27	HLA-B*15:61	557	KKFLPFQQF	0,895
B27	HLA-B*15:61	634	RVYSTGSNVF	0,5973
B27	HLA-B*15:61	687	VASQSIIAY	0,5972
B27	HLA-B*15:61	689	SQSIIAYTM	0,6401
B27	HLA-B*15:61	789	YKTPPIKDF	0,7085
B27	HLA-B*15:61	794	IKDFGGFNF	0,5487
B27	HLA-B*15:61	852	AQKFNGLTV	0,6105
B27	HLA-B*15:61	852	AQKFNGLTVL	0,5775
B27	HLA-B*15:61	853	QKFNGLTVL	0,7774
B27	HLA-B*15:61	894	LQIPFAMQM	0,7963

B27	HLA-B*15:61	919	NQKLIANQF	0,6902
B27	HLA-B*15:61	999	GRLQSLQTY	0,8572
B27	HLA-B*15:61	1038	KRVDFCGKGY	0,5634
B27	HLA-B*15:61	1044	GKGYHLMSF	0,636
B27	HLA-B*15:61	1054	QSAPHGVVF	0,6853
B27	HLA-B*15:61	1087	AHFPREGVF	0,6534
B27	HLA-B*15:61	1264	VLKGVKLHY	0,5156
B27	HLA-B*15:62	20	TRTQLPPAY	0,7053
B27	HLA-B*15:62	22	TQLPPAYTNSF	0,6112
B27	HLA-B*15:62	30	NSFTRGVYY	0,5218
B27	HLA-B*15:62	35	GVYYPDKVF	0,6492
B27	HLA-B*15:62	47	VLHSTQDLF	0,5047
B27	HLA-B*15:62	77	KRFDNPVLPF	0,7733
B27	HLA-B*15:62	184	GNFKNLREF	0,6307
B27	HLA-B*15:62	192	FVFKNIDGY	0,5448
B27	HLA-B*15:62	212	LVRDLPQGF	0,5648
B27	HLA-B*15:62	236	TRFQTLLAL	0,573
B27	HLA-B*15:62	240	TLLALHRSY	0,6832
B27	HLA-B*15:62	261	GAAAYYVGY	0,5324
B27	HLA-B*15:62	267	VGYLQPRTF	0,6274
B27	HLA-B*15:62	327	VRFPNITNL	0,7421
B27	HLA-B*15:62	339	GEVFNATRF	0,7077
B27	HLA-B*15:62	345	TRFASVYAW	0,577
B27	HLA-B*15:62	366	SVLYNSASF	0,5129
B27	HLA-B*15:62	369	YNSASFSTF	0,746
B27	HLA-B*15:62	408	RQIAPGQTG	0,5385
B27	HLA-B*15:62	413	GQTGKIADY	0,813
B27	HLA-B*15:62	443	SKVGGNYNY	0,9183
B27	HLA-B*15:62	464	FERDISTEIY	0,6286
B27	HLA-B*15:62	497	FQPTNGVGY	0,8232
B27	HLA-B*15:62	557	KKFLPFQQF	0,9461
B27	HLA-B*15:62	628	QLTPTWRVY	0,5393
B27	HLA-B*15:62	634	RVYSTGSNVF	0,7129
B27	HLA-B*15:62	687	VASQSIIAY	0,7985
B27	HLA-B*15:62	689	SQSIIAYTM	0,7527
B27	HLA-B*15:62	699	LGAENSVAY	0,5751
B27	HLA-B*15:62	710	NSIAIPTNF	0,5361
B27	HLA-B*15:62	789	YKTPPIKDF	0,7655
B27	HLA-B*15:62	794	IKDFGGFNF	0,6781
B27	HLA-B*15:62	815	RSFIEDLLF	0,5936
B27	HLA-B*15:62	852	AQKFNGLTV	0,582
B27	HLA-B*15:62	852	AQKFNGLTVL	0,5232
B27	HLA-B*15:62	853	QKFNGLTVL	0,7613
B27	HLA-B*15:62	893	ALQIPFAMQM	0,5135
B27	HLA-B*15:62	894	LQIPFAMQM	0,911
B27	HLA-B*15:62	896	IPFAMQMAY	0,5327
B27	HLA-B*15:62	919	NQKLIANQF	0,8564
B27	HLA-B*15:62	999	GRLQSLQTY	0,8305
B27	HLA-B*15:62	1044	GKGYHLMSF	0,7316
B27	HLA-B*15:62	1054	QSAPHGVVF	0,8256

B27	HLA-B*15:62	1087	AHFPREGVF	0,7587
B27	HLA-B*15:62	1113	QIITTDNTF	0,5006
B27	HLA-B*15:62	1130	IGIVNNTVY	0,5213
B27	HLA-B*15:62	1264	VLKGVKLHY	0,645
B27	HLA-B*15:69	20	TRTQLPPAY	0,7227
B27	HLA-B*15:69	22	TQLPPAYTNSF	0,5591
B27	HLA-B*15:69	35	GVYYPDKVF	0,5772
B27	HLA-B*15:69	77	KRFDNPVLPF	0,851
B27	HLA-B*15:69	184	GNFKNLREF	0,546
B27	HLA-B*15:69	236	TRFQTLLAL	0,6858
B27	HLA-B*15:69	240	TLLALHRSY	0,525
B27	HLA-B*15:69	267	VGYLQPRTF	0,5372
B27	HLA-B*15:69	327	VRFPNITNL	0,8345
B27	HLA-B*15:69	339	GEVFNATRF	0,7224
B27	HLA-B*15:69	345	TRFASVYAW	0,5818
B27	HLA-B*15:69	369	YNSASFSTF	0,6438
B27	HLA-B*15:69	408	RQIAPGQTG	0,5308
B27	HLA-B*15:69	413	GQTGKIADY	0,7354
B27	HLA-B*15:69	443	SKVGGNYNY	0,8229
B27	HLA-B*15:69	464	FERDISTEIY	0,6128
B27	HLA-B*15:69	497	FQPTNGVGY	0,674
B27	HLA-B*15:69	505	YQPYRVVVL	0,5216
B27	HLA-B*15:69	557	KKFLPFQQF	0,9341
B27	HLA-B*15:69	634	RVYSTGSNVF	0,6281
B27	HLA-B*15:69	687	VASQSIIAY	0,6873
B27	HLA-B*15:69	689	SQSIIAYTM	0,7524
B27	HLA-B*15:69	789	YKTPPIKDF	0,6877
B27	HLA-B*15:69	794	IKDFGGFNF	0,691
B27	HLA-B*15:69	815	RSFIEDLLF	0,5525
B27	HLA-B*15:69	852	AQKFNGLTV	0,584
B27	HLA-B*15:69	852	AQKFNGLTVL	0,6049
B27	HLA-B*15:69	853	QKFNGLTVL	0,7612
B27	HLA-B*15:69	893	ALQIPFAMQM	0,5311
B27	HLA-B*15:69	894	LQIPFAMQM	0,8912
B27	HLA-B*15:69	919	NQKLIANQF	0,8102
B27	HLA-B*15:69	999	GRLQSLQTY	0,8376
B27	HLA-B*15:69	1016	AEIRASANL	0,5219
B27	HLA-B*15:69	1038	KRVDFCGKGY	0,5143
B27	HLA-B*15:69	1044	GKGYHLMSF	0,7181
B27	HLA-B*15:69	1054	QSAPHGVVF	0,7141
B27	HLA-B*15:69	1087	AHFPREGVF	0,769
B27	HLA-B*15:69	1264	VLKGVKLHY	0,5553
B27	HLA-B*15:72	20	TRTQLPPAY	0,7269
B27	HLA-B*15:72	236	TRFQTLLAL	0,6174
B27	HLA-B*15:72	1087	AHFPREGVF	0,6545
B27	HLA-B*15:74	20	TRTQLPPAY	0,712
B27	HLA-B*15:74	22	TQLPPAYTNSF	0,6447
B27	HLA-B*15:74	30	NSFTRGVYY	0,5264
B27	HLA-B*15:74	35	GVYYPDKVF	0,6849
B27	HLA-B*15:74	47	VLHSTQDLF	0,519

B27	HLA-B*15:74	77	KRFDNPVLPF	0,8142
B27	HLA-B*15:74	84	LPFNDGVYF	0,5129
B27	HLA-B*15:74	184	GNFKNLREF	0,6596
B27	HLA-B*15:74	186	FKNLREFVF	0,5086
B27	HLA-B*15:74	192	FVFKNIDGY	0,5716
B27	HLA-B*15:74	212	LVRDLPQGF	0,5986
B27	HLA-B*15:74	236	TRFQTLLAL	0,6029
B27	HLA-B*15:74	240	TLLALHRSY	0,6632
B27	HLA-B*15:74	261	GAAAYYVGY	0,5496
B27	HLA-B*15:74	267	VGYLQPRTF	0,6398
B27	HLA-B*15:74	320	VQPTESIVRF	0,5264
B27	HLA-B*15:74	327	VRFPNITNL	0,7868
B27	HLA-B*15:74	339	GEVFNATRF	0,7102
B27	HLA-B*15:74	345	TRFASVYAW	0,583
B27	HLA-B*15:74	366	SVLYNSASF	0,5392
B27	HLA-B*15:74	369	YNSASFSTF	0,7528
B27	HLA-B*15:74	408	RQIAPGQTG	0,5683
B27	HLA-B*15:74	413	GQTGKIADY	0,7923
B27	HLA-B*15:74	443	SKVGGNYNY	0,9075
B27	HLA-B*15:74	456	FRKSNLKPF	0,5033
B27	HLA-B*15:74	464	FERDISTEIY	0,6276
B27	HLA-B*15:74	497	FQPTNGVGY	0,8159
B27	HLA-B*15:74	557	KKFLPFQQF	0,96
B27	HLA-B*15:74	628	QLTPTWRVY	0,5215
B27	HLA-B*15:74	634	RVYSTGSNVF	0,7445
B27	HLA-B*15:74	687	VASQSIIAY	0,7995
B27	HLA-B*15:74	689	SQSIIAYTM	0,7813
B27	HLA-B*15:74	699	LGAENSVAY	0,5653
B27	HLA-B*15:74	710	NSIAIPTNF	0,5635
B27	HLA-B*15:74	789	YKTPPIKDF	0,7759
B27	HLA-B*15:74	794	IKDFGGFNF	0,6901
B27	HLA-B*15:74	815	RSFIEDLLF	0,6421
B27	HLA-B*15:74	852	AQKFNGLTV	0,6162
B27	HLA-B*15:74	852	AQKFNGLTVL	0,5897
B27	HLA-B*15:74	853	QKFNGLTVL	0,772
B27	HLA-B*15:74	880	GTITSGWTF	0,5407
B27	HLA-B*15:74	893	ALQIPFAMQM	0,57
B27	HLA-B*15:74	894	LQIPFAMQM	0,9324
B27	HLA-B*15:74	896	IPFAMQMAY	0,5172
B27	HLA-B*15:74	919	NQKLIANQF	0,843
B27	HLA-B*15:74	999	GRLQSLQTY	0,8373
B27	HLA-B*15:74	1044	GKGYHLMSF	0,7534
B27	HLA-B*15:74	1054	QSAPHGVVF	0,8478
B27	HLA-B*15:74	1087	AHFPREGVF	0,7577
B27	HLA-B*15:74	1113	QIITTDNTF	0,5173
B27	HLA-B*15:74	1130	IGIVNNTVY	0,5267
B27	HLA-B*15:74	1264	VLKGVKLHY	0,6447
B27	HLA-B*15:80	20	TRTQLPPAY	0,7191
B27	HLA-B*15:80	236	TRFQTLLAL	0,5772
B27	HLA-B*15:80	1087	AHFPREGVF	0,5968

B27	HLA-B*15:91	153	MESEFRVY	0,6327
B27	HLA-B*15:91	297	SETKCTLKSF	0,5716
B27	HLA-B*15:91	339	GEVFNATRF	0,8899
B27	HLA-B*15:91	464	FERDISTEIY	0,7127
B27	HLA-B*15:91	653	AEHVNNSY	0,6902
B27	HLA-B*15:91	689	SQSIIAYTM	0,5235
B27	HLA-B*15:91	919	NQKLIANQF	0,6049
B27	HLA-B*15:91	1016	AEIRASANL	0,7495
B27	HLA-B*15:91	1201	QELGKYEQY	0,8926
B27	HLA-B*15:93	236	TRFQTLLAL	0,7254
B27	HLA-B*15:93	1087	AHFPREGVF	0,7077
B27	HLA-B*15:98	20	TRTQLPPAY	0,7211
B27	HLA-B*15:98	22	TQLPPAYTNSF	0,6455
B27	HLA-B*15:98	35	GVYYPDKVF	0,6663
B27	HLA-B*15:98	47	VLHSTQDLF	0,5243
B27	HLA-B*15:98	77	KRFDNPVLPF	0,8296
B27	HLA-B*15:98	184	GNFKNLREF	0,6499
B27	HLA-B*15:98	186	FKNLREFVF	0,5195
B27	HLA-B*15:98	192	FVFKNIDGY	0,5282
B27	HLA-B*15:98	212	LVRDLPQGF	0,576
B27	HLA-B*15:98	236	TRFQTLLAL	0,608
B27	HLA-B*15:98	240	TLLALHRSY	0,6413
B27	HLA-B*15:98	261	GAAAYYVGY	0,5148
B27	HLA-B*15:98	267	VGYLQPRTF	0,643
B27	HLA-B*15:98	320	VQPTESIVRF	0,5258
B27	HLA-B*15:98	327	VRFPNITNL	0,8039
B27	HLA-B*15:98	339	GEVFNATRF	0,7209
B27	HLA-B*15:98	345	TRFASVYAW	0,6043
B27	HLA-B*15:98	366	SVLYNSASF	0,5032
B27	HLA-B*15:98	369	YNSASFSTF	0,7633
B27	HLA-B*15:98	408	RQIAPGQTG	0,5804
B27	HLA-B*15:98	413	GQTGKIADY	0,8037
B27	HLA-B*15:98	443	SKVGGNYNY	0,9112
B27	HLA-B*15:98	456	FRKSNLKPF	0,5235
B27	HLA-B*15:98	464	FERDISTEIY	0,635
B27	HLA-B*15:98	497	FQPTNGVGY	0,8242
B27	HLA-B*15:98	557	KKFLPFQQF	0,9568
B27	HLA-B*15:98	628	QLTPTWRVY	0,5065
B27	HLA-B*15:98	634	RVYSTGSNVF	0,7212
B27	HLA-B*15:98	687	VASQSIIAY	0,7907
B27	HLA-B*15:98	689	SQSIIAYTM	0,7655
B27	HLA-B*15:98	699	LGAENSVAY	0,5647
B27	HLA-B*15:98	710	NSIAIPTNF	0,5451
B27	HLA-B*15:98	789	YKTPPIKDF	0,8036
B27	HLA-B*15:98	794	IKDFGGFNF	0,7241
B27	HLA-B*15:98	815	RSFIEDLLF	0,624
B27	HLA-B*15:98	852	AQKFNGLTV	0,6121
B27	HLA-B*15:98	852	AQKFNGLTVL	0,5743
B27	HLA-B*15:98	853	QKFNGLTVL	0,7866
B27	HLA-B*15:98	880	GTITSGWTF	0,5063

B27	HLA-B*15:98	893	ALQIPFAMQM	0,5298
B27	HLA-B*15:98	894	LQIPFAMQM	0,9192
B27	HLA-B*15:98	919	NQKLIANQF	0,8596
B27	HLA-B*15:98	999	GRLQSLQTY	0,8486
B27	HLA-B*15:98	1044	GKGYHLMSF	0,7673
B27	HLA-B*15:98	1054	QSAPHGVVF	0,8355
B27	HLA-B*15:98	1087	AHFPREGVF	0,7921
B27	HLA-B*15:98	1113	QIITTDNTF	0,5046
B27	HLA-B*15:98	1130	IGIVNNTVY	0,5224
B27	HLA-B*15:98	1264	VLKGVKLHY	0,6224
B27	HLA-B*27:02	77	KRFDNPVLPF	0,9936
B27	HLA-B*27:02	236	TRFQTLLAL	0,9672
B27	HLA-B*27:02	342	FNATRFASVYAW	0,5105
B27	HLA-B*27:02	343	NATRFASVYAW	0,7021
B27	HLA-B*27:02	344	ATRFASVYAW	0,9567
B27	HLA-B*27:02	345	TRFASVYAW	0,9908
B27	HLA-B*27:02	356	KRISNCVADY	0,7362
B27	HLA-B*27:02	814	KRSFIEDLLF	0,9772
B27	HLA-B*27:02	846	ARDLICAQKF	0,8438
B27	HLA-B*27:02	999	GRLQSLQTY	0,9922
B27	HLA-B*27:03	77	KRFDNPVLPF	0,9834
B27	HLA-B*27:03	814	KRSFIEDLLF	0,8897
B27	HLA-B*27:04	77	KRFDNPVLPF	0,986
B27	HLA-B*27:04	235	ITRFQTLLAL	0,5856
B27	HLA-B*27:04	236	TRFQTLLAL	0,9822
B27	HLA-B*27:04	327	VRFPNITNL	0,9799
B27	HLA-B*27:04	453	YRLFRKSNL	0,6317
B27	HLA-B*27:04	814	KRSFIEDLLF	0,8383
B27	HLA-B*27:04	904	YRFNGIGVT	0,6001
B27	HLA-B*27:04	999	GRLQSLQTY	0,967
B27	HLA-B*27:05	77	KRFDNPVLPF	0,9904
B27	HLA-B*27:05	101	IRGWIFGTTL	0,6849
B27	HLA-B*27:05	235	ITRFQTLLAL	0,5227
B27	HLA-B*27:05	236	TRFQTLLAL	0,9782
B27	HLA-B*27:05	318	FRVQPTESI	0,6722
B27	HLA-B*27:05	327	VRFPNITNL	0,9818
B27	HLA-B*27:05	344	ATRFASVYAW	0,7783
B27	HLA-B*27:05	345	TRFASVYAW	0,9414
B27	HLA-B*27:05	356	KRISNCVADY	0,6725
B27	HLA-B*27:05	453	YRLFRKSNL	0,6377
B27	HLA-B*27:05	814	KRSFIEDLL	0,8024
B27	HLA-B*27:05	814	KRSFIEDLLF	0,9257
B27	HLA-B*27:05	846	ARDLICAQKF	0,5398
B27	HLA-B*27:05	904	YRFNGIGVTQ	0,7685
B27	HLA-B*27:05	999	GRLQSLQTY	0,9825
B27	HLA-B*27:05	1038	KRVDFCGKGY	0,8924
B27	HLA-B*27:05	1038	KRVDFCGKGYHL	0,663
B27	HLA-B*27:06	77	KRFDNPVL	0,7543
B27	HLA-B*27:06	77	KRFDNPVLPF	0,9867
B27	HLA-B*27:06	101	IRGWIFGTTL	0,7577

B27	HLA-B*27:06	235	ITRFQTLLAL	0,6798
B27	HLA-B*27:06	236	TRFQTLLAL	0,9905
B27	HLA-B*27:06	318	FRVQPTESI	0,887
B27	HLA-B*27:06	325	SIVRFPNITNL	0,8466
B27	HLA-B*27:06	326	IVRFPNITNL	0,8762
B27	HLA-B*27:06	327	VRFPNITNL	0,9922
B27	HLA-B*27:06	453	YRLFRKSNL	0,7499
B27	HLA-B*27:06	684	ARSVASQSI	0,6368
B27	HLA-B*27:06	764	NRALTGIAV	0,588
B27	HLA-B*27:06	814	KRSFIEDLL	0,749
B27	HLA-B*27:06	814	KRSFIEDLLF	0,6968
B27	HLA-B*27:06	853	QKFNGLTVL	0,5745
B27	HLA-B*27:06	904	YRFNGIGV	0,617
B27	HLA-B*27:06	904	YRFNGIGVTQNV	0,5027
B27	HLA-B*27:06	1038	KRVDFCGKGYHL	0,6164
B27	HLA-B*27:06	1106	QRNFYEPQI	0,601
B27	HLA-B*27:07	77	KRFDNPVL	0,7135
B27	HLA-B*27:07	77	KRFDNPVLPF	0,9902
B27	HLA-B*27:07	101	IRGWIFGTTL	0,8223
B27	HLA-B*27:07	235	ITRFQTLLAL	0,6211
B27	HLA-B*27:07	236	TRFQTLLAL	0,9877
B27	HLA-B*27:07	318	FRVQPTESI	0,8291
B27	HLA-B*27:07	325	SIVRFPNITNL	0,8921
B27	HLA-B*27:07	326	IVRFPNITNL	0,8934
B27	HLA-B*27:07	327	VRFPNITNL	0,9922
B27	HLA-B*27:07	327	VRFPNITNLCPF	0,7232
B27	HLA-B*27:07	344	ATRFASVYAW	0,5458
B27	HLA-B*27:07	345	TRFASVYAW	0,8735
B27	HLA-B*27:07	453	YRLFRKSNL	0,6719
B27	HLA-B*27:07	557	KKFLPFQQF	0,6105
B27	HLA-B*27:07	684	ARSVASQSI	0,577
B27	HLA-B*27:07	764	NRALTGIAV	0,5708
B27	HLA-B*27:07	814	KRSFIEDLL	0,9203
B27	HLA-B*27:07	814	KRSFIEDLLF	0,8818
B27	HLA-B*27:07	904	YRFNGIGV	0,614
B27	HLA-B*27:07	904	YRFNGIGVTQNV	0,5884
B27	HLA-B*27:07	999	GRLQSLQTYV	0,526
B27	HLA-B*27:07	1038	KRVDFCGKGYHL	0,8228
B27	HLA-B*27:07	1106	QRNFYEPQI	0,7313
B27	HLA-B*27:09	77	KRFDNPVL	0,7057
B27	HLA-B*27:09	77	KRFDNPVLPF	0,9931
B27	HLA-B*27:09	101	IRGWIFGTTL	0,8159
B27	HLA-B*27:09	235	ITRFQTLLAL	0,6732
B27	HLA-B*27:09	236	TRFQTLLAL	0,9861
B27	HLA-B*27:09	318	FRVQPTESI	0,8298
B27	HLA-B*27:09	325	SIVRFPNITNL	0,8994
B27	HLA-B*27:09	326	IVRFPNITNL	0,9018
B27	HLA-B*27:09	327	VRFPNITNL	0,9903
B27	HLA-B*27:09	327	VRFPNITNLCPF	0,7706
B27	HLA-B*27:09	344	ATRFASVYAW	0,6784

B27	HLA-B*27:09	345	TRFASVYAW	0,904
B27	HLA-B*27:09	453	YRLFRKSNL	0,6207
B27	HLA-B*27:09	764	NRALTGIAV	0,51
B27	HLA-B*27:09	814	KRSFIEDLL	0,9093
B27	HLA-B*27:09	814	KRSFIEDLLF	0,925
B27	HLA-B*27:09	846	ARDLICAQKF	0,5004
B27	HLA-B*27:09	904	YRFNGIGV	0,6307
B27	HLA-B*27:09	904	YRFNGIGVT	0,5198
B27	HLA-B*27:09	904	YRFNGIGVTQNV	0,6738
B27	HLA-B*27:09	999	GRLQSLQTYV	0,5888
B27	HLA-B*27:09	1038	KRVDFCGKGYHL	0,8281
B27	HLA-B*27:09	1106	QRNFYEPQI	0,7026
B27	HLA-B*27:10	77	KRFDNPVLPF	0,9542
B27	HLA-B*27:10	236	TRFQTLLAL	0,9226
B27	HLA-B*27:10	453	YRLFRKSNL	0,5229
B27	HLA-B*27:13	77	KRFDNPVLPF	0,9904
B27	HLA-B*27:13	101	IRGWIFGTTL	0,6849
B27	HLA-B*27:13	235	ITRFQTLLAL	0,5227
B27	HLA-B*27:13	236	TRFQTLLAL	0,9782
B27	HLA-B*27:13	318	FRVQPTESI	0,6722
B27	HLA-B*27:13	327	VRFPNITNL	0,9818
B27	HLA-B*27:13	344	ATRFASVYAW	0,7783
B27	HLA-B*27:13	345	TRFASVYAW	0,9414
B27	HLA-B*27:13	356	KRISNCVADY	0,6725
B27	HLA-B*27:13	453	YRLFRKSNL	0,6377
B27	HLA-B*27:13	814	KRSFIEDLL	0,8024
B27	HLA-B*27:13	814	KRSFIEDLLF	0,9257
B27	HLA-B*27:13	846	ARDLICAQKF	0,5398
B27	HLA-B*27:13	904	YRFNGIGVTQ	0,7685
B27	HLA-B*27:13	999	GRLQSLQTY	0,9825
B27	HLA-B*27:13	1038	KRVDFCGKGY	0,8924
B27	HLA-B*27:13	1038	KRVDFCGKGYHL	0,663
B27	HLA-B*27:15	77	KRFDNPVLPF	0,9827
B27	HLA-B*27:15	235	ITRFQTLLAL	0,5206
B27	HLA-B*27:15	236	TRFQTLLAL	0,9763
B27	HLA-B*27:15	327	VRFPNITNL	0,9756
B27	HLA-B*27:15	453	YRLFRKSNL	0,6738
B27	HLA-B*27:15	814	KRSFIEDLLF	0,8178
B27	HLA-B*27:15	904	YRFNGIGVT	0,6068
B27	HLA-B*27:15	999	GRLQSLQTY	0,9613
B27	HLA-B*27:17	20	TRTQLPPAY	0,9
B27	HLA-B*27:17	77	KRFDNPVL	0,65
B27	HLA-B*27:17	77	KRFDNPVLPF	0,9915
B27	HLA-B*27:17	101	IRGWIFGTTL	0,7045
B27	HLA-B*27:17	235	ITRFQTLLAL	0,545
B27	HLA-B*27:17	236	TRFQTLLAL	0,9819
B27	HLA-B*27:17	318	FRVQPTESI	0,7209
B27	HLA-B*27:17	325	SIVRFPNITNL	0,8395
B27	HLA-B*27:17	326	IVRFPNITNL	0,8595
B27	HLA-B*27:17	327	VRFPNITNL	0,9862

B27	HLA-B*27:17	327	VRFPNITNLCPF	0,7682
B27	HLA-B*27:17	344	ATRFASVYAW	0,782
B27	HLA-B*27:17	345	TRFASVYAW	0,9514
B27	HLA-B*27:17	356	KRISNCVADY	0,6994
B27	HLA-B*27:17	453	YRLFRKSNL	0,6674
B27	HLA-B*27:17	508	YRVVVLSF	0,6075
B27	HLA-B*27:17	814	KRSFIEDLL	0,8281
B27	HLA-B*27:17	814	KRSFIEDLLF	0,9299
B27	HLA-B*27:17	846	ARDLICAQKF	0,5844
B27	HLA-B*27:17	904	YRFNGIGV	0,5341
B27	HLA-B*27:17	904	YRFNGIGVT	0,5417
B27	HLA-B*27:17	904	YRFNGIGVTQ	0,802
B27	HLA-B*27:17	998	TGRLQSLQTY	0,6318
B27	HLA-B*27:17	999	GRLQSLQTY	0,9859
B27	HLA-B*27:17	1038	KRVDFCGKGY	0,9087
B27	HLA-B*27:17	1038	KRVDFCGKGYHL	0,6876
B27	HLA-B*27:17	1106	QRNFYEPQI	0,5937
B27	HLA-B*27:25	77	KRFDNPVLPF	0,8627
B27	HLA-B*27:25	236	TRFQTLLAL	0,8437
B27	HLA-B*27:28	77	KRFDNPVLPF	0,9752
B27	HLA-B*27:28	101	IRGWIFGTTL	0,6664
B27	HLA-B*27:28	236	TRFQTLLAL	0,9591
B27	HLA-B*27:28	327	VRFPNITNL	0,961
B27	HLA-B*27:28	344	ATRFASVYAW	0,754
B27	HLA-B*27:28	345	TRFASVYAW	0,9368
B27	HLA-B*27:28	356	KRISNCVADY	0,6169
B27	HLA-B*27:28	453	YRLFRKSNL	0,6569
B27	HLA-B*27:28	508	YRVVVLSF	0,6768
B27	HLA-B*27:28	814	KRSFIEDLLF	0,8481
B27	HLA-B*27:28	846	ARDLICAQKF	0,5402
B27	HLA-B*27:28	904	YRFNGIGV	0,5641
B27	HLA-B*27:28	904	YRFNGIGVTQ	0,7159
B27	HLA-B*27:28	999	GRLQSLQTY	0,9569
B27	HLA-B*27:28	1038	KRVDFCGKGY	0,8473
B27	HLA-B*38:01	236	TRFQTLLAL	0,761
B27	HLA-B*38:05	236	TRFQTLLAL	0,761
B27	HLA-B*38:09	236	TRFQTLLAL	0,761
B27	HLA-B*38:11	236	TRFQTLLAL	0,761
B27	HLA-B*39:01	236	TRFQTLLAL	0,9488
B27	HLA-B*39:01	318	FRVQPTESI	0,8358
B27	HLA-B*39:01	505	YQPYRVVVL	0,5961
B27	HLA-B*39:01	576	VRDPQTLEI	0,7426
B27	HLA-B*39:01	689	SQSIIAYTM	0,557
B27	HLA-B*39:01	764	NRALTGIAV	0,8592
B27	HLA-B*39:01	853	QKFNGLTVL	0,7764
B27	HLA-B*39:01	1158	NHTSPDVDL	0,7288
B27	HLA-B*39:02	168	FEYVSQPFL	0,6813
B27	HLA-B*39:02	236	TRFQTLLAL	0,7521
B27	HLA-B*39:02	318	FRVQPTESI	0,588
B27	HLA-B*39:02	464	FERDISTEI	0,7304

B27	HLA-B*39:02	505	YQPYRVVVL	0,7112
B27	HLA-B*39:02	689	SQSIIAYTM	0,6777
B27	HLA-B*39:02	852	AQKFNGLTV	0,5032
B27	HLA-B*39:02	852	AQKFNGLTVL	0,6857
B27	HLA-B*39:02	853	QKFNGLTVL	0,8116
B27	HLA-B*39:02	894	LQIPFAMQM	0,6107
B27	HLA-B*39:02	1016	AEIRASANL	0,7178
B27	HLA-B*39:02	1181	KEIDRLNEV	0,7871
B27	HLA-B*39:04	236	TRFQTLLAL	0,9488
B27	HLA-B*39:04	318	FRVQPTESI	0,8358
B27	HLA-B*39:04	505	YQPYRVVVL	0,5961
B27	HLA-B*39:04	576	VRDPQTLEI	0,7426
B27	HLA-B*39:04	689	SQSIIAYTM	0,557
B27	HLA-B*39:04	764	NRALTGIAV	0,8592
B27	HLA-B*39:04	853	QKFNGLTVL	0,7764
B27	HLA-B*39:04	1158	NHTSPDVDL	0,7288
B27	HLA-B*39:07	20	TRTQLPPAY	0,7614
B27	HLA-B*39:07	236	TRFQTLLAL	0,7603
B27	HLA-B*39:07	345	TRFASVYAW	0,7796
B27	HLA-B*39:07	465	ERDISTEIY	0,6857
B27	HLA-B*39:07	689	SQSIIAYTM	0,5704
B27	HLA-B*39:07	794	IKDFGGFNF	0,5866
B27	HLA-B*39:07	853	QKFNGLTVL	0,5625
B27	HLA-B*39:07	894	LQIPFAMQM	0,5211
B27	HLA-B*39:07	1087	AHFPREGVF	0,6804
B27	HLA-B*39:09	48	LHSTQDLFL	0,5309
B27	HLA-B*39:09	236	TRFQTLLAL	0,9121
B27	HLA-B*39:09	269	YLQPRTFLL	0,5194
B27	HLA-B*39:09	318	FRVQPTESI	0,7813
B27	HLA-B*39:09	505	YQPYRVVVL	0,812
B27	HLA-B*39:09	689	SQSIIAYTM	0,6287
B27	HLA-B*39:09	764	NRALTGIAV	0,7776
B27	HLA-B*39:09	853	QKFNGLTVL	0,766
B27	HLA-B*39:09	1158	NHTSPDVDL	0,7704
B27	HLA-B*39:14	236	TRFQTLLAL	0,9724
B27	HLA-B*39:14	318	FRVQPTESI	0,8722
B27	HLA-B*39:14	327	VRFPNITNL	0,9443
B27	HLA-B*39:14	453	YRLFRKSNL	0,7017
B27	HLA-B*39:14	505	YQPYRVVVL	0,7017
B27	HLA-B*39:14	576	VRDPQTLEI	0,769
B27	HLA-B*39:14	689	SQSIIAYTM	0,5743
B27	HLA-B*39:14	764	NRALTGIAV	0,865
B27	HLA-B*39:14	853	QKFNGLTVL	0,8612
B27	HLA-B*39:14	1158	NHTSPDVDL	0,6977
B27	HLA-B*39:18	236	TRFQTLLAL	0,7919
B27	HLA-B*39:18	764	NRALTGIAV	0,7027
B27	HLA-B*39:23	168	FEYVSQPFL	0,6813
B27	HLA-B*39:23	236	TRFQTLLAL	0,7521
B27	HLA-B*39:23	318	FRVQPTESI	0,588
B27	HLA-B*39:23	464	FERDISTEI	0,7304

B27	HLA-B*39:23	505	YQPYRVVVL	0,7112
B27	HLA-B*39:23	689	SQSIIAYTM	0,6777
B27	HLA-B*39:23	852	AQKFNGLTV	0,5032
B27	HLA-B*39:23	852	AQKFNGLTVL	0,6857
B27	HLA-B*39:23	853	QKFNGLTVL	0,8116
B27	HLA-B*39:23	894	LQIPFAMQM	0,6107
B27	HLA-B*39:23	1016	AEIRASANL	0,7178
B27	HLA-B*39:23	1181	KEIDRLNEV	0,7871
B27	HLA-B*39:26	236	TRFQTLLAL	0,9488
B27	HLA-B*39:26	318	FRVQPTESI	0,8358
B27	HLA-B*39:26	505	YQPYRVVVL	0,5961
B27	HLA-B*39:26	576	VRDPQTLEI	0,7426
B27	HLA-B*39:26	689	SQSIIAYTM	0,557
B27	HLA-B*39:26	764	NRALTGIAV	0,8592
B27	HLA-B*39:26	853	QKFNGLTVL	0,7764
B27	HLA-B*39:26	1158	NHTSPDVDL	0,7288
B27	HLA-B*39:27	236	TRFQTLLAL	0,9298
B27	HLA-B*39:27	318	FRVQPTESI	0,7781
B27	HLA-B*39:27	327	VRFPNITNL	0,8349
B27	HLA-B*39:27	505	YQPYRVVVL	0,5535
B27	HLA-B*39:27	576	VRDPQTLEI	0,726
B27	HLA-B*39:27	764	NRALTGIAV	0,8242
B27	HLA-B*39:27	853	QKFNGLTVL	0,6621
B27	HLA-B*39:27	1158	NHTSPDVDL	0,5984
B27	HLA-B*39:29	236	TRFQTLLAL	0,9638
B27	HLA-B*39:29	318	FRVQPTESI	0,8376
B27	HLA-B*39:29	327	VRFPNITNL	0,9218
B27	HLA-B*39:29	453	YRLFRKSNL	0,6499
B27	HLA-B*39:29	505	YQPYRVVVL	0,5823
B27	HLA-B*39:29	764	NRALTGIAV	0,8145
B27	HLA-B*39:29	853	QKFNGLTVL	0,8131
B27	HLA-B*39:29	1158	NHTSPDVDL	0,5748
B27	HLA-B*39:30	236	TRFQTLLAL	0,8953
B27	HLA-B*39:30	318	FRVQPTESI	0,6956
B27	HLA-B*39:30	764	NRALTGIAV	0,7463
B27	HLA-B*39:30	853	QKFNGLTVL	0,6386
B27	HLA-B*39:30	1158	NHTSPDVDL	0,6332
B27	HLA-B*39:32	236	TRFQTLLAL	0,8554
B27	HLA-B*39:32	505	YQPYRVVVL	0,5179
B27	HLA-B*39:32	764	NRALTGIAV	0,7295
B27	HLA-B*39:32	1158	NHTSPDVDL	0,5368
B27	HLA-B*40:12	168	FEYVSQPFL	0,5354
B27	HLA-B*40:12	689	SQSIIAYTM	0,5826
B27	HLA-B*40:12	852	AQKFNGLTVL	0,6787
B27	HLA-B*48:01	852	AQKFNGLTVL	0,6838
B27	HLA-B*48:02	22	TQLPPAYTNSF	0,5241
B27	HLA-B*48:02	77	KRFDNPVLPF	0,5598
B27	HLA-B*48:02	339	GEVFNATRF	0,7713
B27	HLA-B*48:02	345	TRFASVYAW	0,5657
B27	HLA-B*48:02	369	YNSASFSTF	0,5533

B27	HLA-B*48:02	413	GQTGKIADY	0,6026
B27	HLA-B*48:02	443	SKVGGNYNY	0,8498
B27	HLA-B*48:02	464	FERDISTEIY	0,6629
B27	HLA-B*48:02	497	FQPTNGVGY	0,6828
B27	HLA-B*48:02	557	KKFLPFQQF	0,7916
B27	HLA-B*48:02	687	VASQSIIAY	0,6788
B27	HLA-B*48:02	689	SQSIIAYTM	0,6856
B27	HLA-B*48:02	789	YKTPPIKDF	0,5888
B27	HLA-B*48:02	794	IKDFGGFNF	0,6372
B27	HLA-B*48:02	853	QKFNGLTVL	0,5314
B27	HLA-B*48:02	894	LQIPFAMQM	0,7465
B27	HLA-B*48:02	919	NQKLIANQF	0,7202
B27	HLA-B*48:02	999	GRLQSLQTY	0,6453
B27	HLA-B*48:02	1054	QSAPHGVVF	0,5772
B27	HLA-B*48:02	1087	AHFPREGVF	0,5026
B27	HLA-B*48:02	1201	QELGKYEQY	0,6932
B27	HLA-B*48:03	168	FEYVSQPFL	0,5354
B27	HLA-B*48:03	689	SQSIIAYTM	0,5826
B27	HLA-B*48:03	852	AQKFNGLTVL	0,6787
B27	HLA-B*48:04	852	AQKFNGLTVL	0,6838
B27	HLA-B*48:05	852	AQKFNGLTV	0,5272
B27	HLA-B*48:05	852	AQKFNGLTVL	0,5881
B27	HLA-B*48:09	852	AQKFNGLTVL	0,6838
B27	HLA-B*48:10	168	FEYVSQPFL	0,5246
B27	HLA-B*48:10	852	AQKFNGLTVL	0,6418
B27	HLA-B*48:10	853	QKFNGLTVL	0,614
B27	HLA-B*48:10	894	LQIPFAMQM	0,5173
B27	HLA-B*48:12	168	FEYVSQPFL	0,5246
B27	HLA-B*48:12	852	AQKFNGLTVL	0,6418
B27	HLA-B*48:12	853	QKFNGLTVL	0,614
B27	HLA-B*48:12	894	LQIPFAMQM	0,5173
B27	HLA-B*48:13	168	FEYVSQPFL	0,5246
B27	HLA-B*48:13	852	AQKFNGLTVL	0,6418
B27	HLA-B*48:13	853	QKFNGLTVL	0,614
B27	HLA-B*48:13	894	LQIPFAMQM	0,5173
B27	HLA-B*73:01	236	TRFQTLLAL	0,8088
B27	HLA-B*73:01	764	NRALTGIAV	0,6815
B44	HLA-B*15:53	153	MESEFRVY	0,5616
B44	HLA-B*15:53	339	GEVFNATRF	0,8089
B44	HLA-B*15:53	369	YNSASFSTF	0,5654
B44	HLA-B*15:53	413	GQTGKIADY	0,7749
B44	HLA-B*15:53	443	SKVGGNYNY	0,7828
B44	HLA-B*15:53	464	FERDISTEI	0,5031
B44	HLA-B*15:53	464	FERDISTEIY	0,8541
B44	HLA-B*15:53	497	FQPTNGVGY	0,7516
B44	HLA-B*15:53	557	KKFLPFQQF	0,6864
B44	HLA-B*15:53	634	RVYSTGSNVF	0,5726
B44	HLA-B*15:53	653	AEHVNNSY	0,6845
B44	HLA-B*15:53	687	VASQSIIAY	0,6214
B44	HLA-B*15:53	689	SQSIIAYTM	0,6354

B44	HLA-B*15:53	852	AQKFNGLTV	0,5366
B44	HLA-B*15:53	852	AQKFNGLTVL	0,5324
B44	HLA-B*15:53	894	LQIPFAMQM	0,7568
B44	HLA-B*15:53	919	NQKLIANQF	0,8277
B44	HLA-B*15:53	1016	AEIRASANL	0,5785
B44	HLA-B*15:53	1054	QSAPHGVVF	0,6409
B44	HLA-B*15:53	1264	VLKGVKLHY	0,583
B44	HLA-B*18:01	153	MESEFRVY	0,8458
B44	HLA-B*18:01	168	FEYVSQPF	0,5811
B44	HLA-B*18:01	464	FERDISTEIY	0,7821
B44	HLA-B*18:01	724	TEILPVSM	0,6734
B44	HLA-B*18:01	1201	QELGKYEQY	0,8379
B44	HLA-B*18:03	153	MESEFRVY	0,5628
B44	HLA-B*18:03	464	FERDISTEI	0,5105
B44	HLA-B*18:03	464	FERDISTEIY	0,5603
B44	HLA-B*18:05	153	MESEFRVY	0,8458
B44	HLA-B*18:05	168	FEYVSQPF	0,5811
B44	HLA-B*18:05	464	FERDISTEIY	0,7821
B44	HLA-B*18:05	724	TEILPVSM	0,6734
B44	HLA-B*18:05	1201	QELGKYEQY	0,8379
B44	HLA-B*18:06	153	MESEFRVY	0,6821
B44	HLA-B*18:06	464	FERDISTEIY	0,5613
B44	HLA-B*18:10	153	MESEFRVY	0,6833
B44	HLA-B*18:10	339	GEVFNATRF	0,6957
B44	HLA-B*18:10	464	FERDISTEIY	0,7407
B44	HLA-B*18:10	724	TEILPVSM	0,6043
B44	HLA-B*18:10	1201	QELGKYEQY	0,7825
B44	HLA-B*18:11	153	MESEFRVY	0,7741
B44	HLA-B*18:11	339	GEVFNATRF	0,7018
B44	HLA-B*18:11	464	FERDISTEI	0,5685
B44	HLA-B*18:11	464	FERDISTEIY	0,799
B44	HLA-B*18:11	724	TEILPVSM	0,6215
B44	HLA-B*18:11	1201	QELGKYEQY	0,834
B44	HLA-B*18:13	153	MESEFRVY	0,8429
B44	HLA-B*18:13	168	FEYVSQPF	0,5593
B44	HLA-B*18:13	388	NDLCFTNVY	0,5383
B44	HLA-B*18:13	464	FERDISTEIY	0,7752
B44	HLA-B*18:13	1201	QELGKYEQY	0,7889
B44	HLA-B*18:13	1206	YEQYIKWPW	0,5226
B44	HLA-B*18:15	153	MESEFRVY	0,7703
B44	HLA-B*18:15	168	FEYVSQPF	0,5051
B44	HLA-B*18:15	464	FERDISTEIY	0,7513
B44	HLA-B*18:15	896	IPFAMQMAY	0,5009
B44	HLA-B*18:19	153	MESEFRVY	0,6571
B44	HLA-B*18:19	464	FERDISTEIY	0,725
B44	HLA-B*18:20	153	MESEFRVY	0,8458
B44	HLA-B*18:20	168	FEYVSQPF	0,5811
B44	HLA-B*18:20	464	FERDISTEIY	0,7821
B44	HLA-B*18:20	724	TEILPVSM	0,6734
B44	HLA-B*18:20	1201	QELGKYEQY	0,8379

B44	HLA-B*37:01	168	FEYVSQPFL	0,6847
B44	HLA-B*37:01	1181	KEIDRLNEV	0,8589
B44	HLA-B*37:04	168	FEYVSQPFL	0,6712
B44	HLA-B*40:01	168	FEYVSQPFL	0,9485
B44	HLA-B*40:01	168	FEYVSQPFLM	0,5398
B44	HLA-B*40:01	339	GEVFNATRF	0,8888
B44	HLA-B*40:01	464	FERDISTEI	0,9094
B44	HLA-B*40:01	660	YECDIPIGAGI	0,5931
B44	HLA-B*40:01	818	IEDLLFNKV	0,639
B44	HLA-B*40:01	987	VEAEVQIDRL	0,8709
B44	HLA-B*40:01	989	AEVQIDRLI	0,9071
B44	HLA-B*40:01	1016	AEIRASANL	0,9748
B44	HLA-B*40:01	1181	KEIDRLNEV	0,906
B44	HLA-B*40:01	1194	NESLIDLQEL	0,5405
B44	HLA-B*40:01	1256	FDEDDSEPVL	0,5944
B44	HLA-B*40:01	1261	SEPVLKGVKL	0,736
B44	HLA-B*40:02	155	SEFRVYSSA	0,652
B44	HLA-B*40:02	168	FEYVSQPFL	0,8998
B44	HLA-B*40:02	190	REFVFKNI	0,6104
B44	HLA-B*40:02	339	GEVFNATRF	0,776
B44	HLA-B*40:02	464	FERDISTEI	0,8995
B44	HLA-B*40:02	660	YECDIPIGA	0,512
B44	HLA-B*40:02	724	TEILPVSM	0,8512
B44	HLA-B*40:02	818	IEDLLFNKV	0,6624
B44	HLA-B*40:02	987	VEAEVQIDRL	0,7072
B44	HLA-B*40:02	989	AEVQIDRL	0,7511
B44	HLA-B*40:02	989	AEVQIDRLI	0,8417
B44	HLA-B*40:02	1016	AEIRASANL	0,9365
B44	HLA-B*40:02	1181	KEIDRLNEV	0,9443
B44	HLA-B*40:02	1261	SEPVLKGVKL	0,654
B44	HLA-B*40:05	155	SEFRVYSSA	0,5629
B44	HLA-B*40:05	168	FEYVSQPFL	0,7104
B44	HLA-B*40:05	464	FERDISTEI	0,8023
B44	HLA-B*40:05	1016	AEIRASANL	0,8349
B44	HLA-B*40:05	1181	KEIDRLNEV	0,879
B44	HLA-B*40:06	155	SEFRVYSSA	0,7601
B44	HLA-B*40:06	168	FEYVSQPFL	0,7587
B44	HLA-B*40:06	464	FERDISTEI	0,7272
B44	HLA-B*40:06	618	TEVPVAIHA	0,7591
B44	HLA-B*40:06	660	YECDIPIGA	0,6484
B44	HLA-B*40:06	818	IEDLLFNKV	0,6059
B44	HLA-B*40:06	1016	AEIRASANL	0,6441
B44	HLA-B*40:06	1181	KEIDRLNEV	0,765
B44	HLA-B*40:11	168	FEYVSQPFL	0,905
B44	HLA-B*40:11	339	GEVFNATRF	0,8092
B44	HLA-B*40:11	464	FERDISTEI	0,8897
B44	HLA-B*40:11	618	TEVPVAIHA	0,6448
B44	HLA-B*40:11	724	TEILPVSM	0,7985
B44	HLA-B*40:11	818	IEDLLFNKV	0,6927
B44	HLA-B*40:11	987	VEAEVQIDRL	0,7437

B44	HLA-B*40:11	989	AEVQIDRL	0,7049
B44	HLA-B*40:11	989	AEVQIDRLI	0,8855
B44	HLA-B*40:11	1016	AEIRASANL	0,9391
B44	HLA-B*40:11	1181	KEIDRLNEV	0,9057
B44	HLA-B*40:11	1256	FDEDDSEPVL	0,5382
B44	HLA-B*40:11	1261	SEPVLKGVKL	0,6019
B44	HLA-B*40:14	168	FEYVSQPFL	0,905
B44	HLA-B*40:14	339	GEVFNATRF	0,8092
B44	HLA-B*40:14	464	FERDISTEI	0,8897
B44	HLA-B*40:14	618	TEVPVAIHA	0,6448
B44	HLA-B*40:14	724	TEILPVSM	0,7985
B44	HLA-B*40:14	818	IEDLLFNKV	0,6927
B44	HLA-B*40:14	987	VEAEVQIDRL	0,7437
B44	HLA-B*40:14	989	AEVQIDRL	0,7049
B44	HLA-B*40:14	989	AEVQIDRLI	0,8855
B44	HLA-B*40:14	1016	AEIRASANL	0,9391
B44	HLA-B*40:14	1181	KEIDRLNEV	0,9057
B44	HLA-B*40:14	1256	FDEDDSEPVL	0,5382
B44	HLA-B*40:14	1261	SEPVLKGVKL	0,6019
B44	HLA-B*40:15	155	SEFRVYSSA	0,5585
B44	HLA-B*40:15	168	FEYVSQPFL	0,7239
B44	HLA-B*40:15	214	RDLPQGFSAL	0,5122
B44	HLA-B*40:15	464	FERDISTEI	0,8506
B44	HLA-B*40:15	1016	AEIRASANL	0,8988
B44	HLA-B*40:15	1181	KEIDRLNEV	0,8892
B44	HLA-B*40:16	155	SEFRVYSSA	0,5585
B44	HLA-B*40:16	168	FEYVSQPFL	0,7239
B44	HLA-B*40:16	214	RDLPQGFSAL	0,5122
B44	HLA-B*40:16	464	FERDISTEI	0,8506
B44	HLA-B*40:16	1016	AEIRASANL	0,8988
B44	HLA-B*40:16	1181	KEIDRLNEV	0,8892
B44	HLA-B*40:20	153	MESEFRVY	0,5689
B44	HLA-B*40:20	168	FEYVSQPFL	0,55
B44	HLA-B*40:20	190	REFVFKNIDGY	0,5832
B44	HLA-B*40:20	339	GEVFNATRF	0,906
B44	HLA-B*40:20	464	FERDISTEI	0,6148
B44	HLA-B*40:20	464	FERDISTEIY	0,806
B44	HLA-B*40:20	653	AEHVNNSY	0,7263
B44	HLA-B*40:20	724	TEILPVSM	0,5311
B44	HLA-B*40:20	989	AEVQIDRLI	0,6133
B44	HLA-B*40:20	1016	AEIRASANL	0,7146
B44	HLA-B*40:20	1181	KEIDRLNEV	0,6384
B44	HLA-B*40:20	1201	QELGKYEQY	0,8567
B44	HLA-B*40:26	155	SEFRVYSSA	0,5119
B44	HLA-B*40:26	168	FEYVSQPFL	0,7736
B44	HLA-B*40:26	339	GEVFNATRF	0,7063
B44	HLA-B*40:26	464	FERDISTEI	0,8764
B44	HLA-B*40:26	724	TEILPVSM	0,6548
B44	HLA-B*40:26	989	AEVQIDRLI	0,7297
B44	HLA-B*40:26	1016	AEIRASANL	0,9027

B44	HLA-B*40:26	1181	KEIDRLNEV	0,9114
B44	HLA-B*40:29	155	SEFRVYSSA	0,6037
B44	HLA-B*40:29	168	FEYVSQPFL	0,838
B44	HLA-B*40:29	190	REFVFKNI	0,5309
B44	HLA-B*40:29	339	GEVFNATRF	0,7589
B44	HLA-B*40:29	464	FERDISTEI	0,8407
B44	HLA-B*40:29	724	TEILPVSM	0,764
B44	HLA-B*40:29	818	IEDLLFNKV	0,5439
B44	HLA-B*40:29	987	VEAEVQIDRL	0,6192
B44	HLA-B*40:29	989	AEVQIDRL	0,6685
B44	HLA-B*40:29	989	AEVQIDRLI	0,7966
B44	HLA-B*40:29	1016	AEIRASANL	0,9162
B44	HLA-B*40:29	1181	KEIDRLNEV	0,9308
B44	HLA-B*40:29	1261	SEPVLKGVKL	0,5302
B44	HLA-B*40:35	155	SEFRVYSSA	0,652
B44	HLA-B*40:35	168	FEYVSQPFL	0,8998
B44	HLA-B*40:35	190	REFVFKNI	0,6104
B44	HLA-B*40:35	339	GEVFNATRF	0,776
B44	HLA-B*40:35	464	FERDISTEI	0,8995
B44	HLA-B*40:35	660	YECDIPIGA	0,512
B44	HLA-B*40:35	724	TEILPVSM	0,8512
B44	HLA-B*40:35	818	IEDLLFNKV	0,6624
B44	HLA-B*40:35	987	VEAEVQIDRL	0,7072
B44	HLA-B*40:35	989	AEVQIDRL	0,7511
B44	HLA-B*40:35	989	AEVQIDRLI	0,8417
B44	HLA-B*40:35	1016	AEIRASANL	0,9365
B44	HLA-B*40:35	1181	KEIDRLNEV	0,9443
B44	HLA-B*40:35	1261	SEPVLKGVKL	0,654
B44	HLA-B*40:39	155	SEFRVYSSA	0,667
B44	HLA-B*40:39	168	FEYVSQPFL	0,8825
B44	HLA-B*40:39	190	REFVFKNI	0,6464
B44	HLA-B*40:39	339	GEVFNATRF	0,7646
B44	HLA-B*40:39	464	FERDISTEI	0,911
B44	HLA-B*40:39	660	YECDIPIGA	0,5358
B44	HLA-B*40:39	724	TEILPVSM	0,8562
B44	HLA-B*40:39	818	IEDLLFNKV	0,6636
B44	HLA-B*40:39	987	VEAEVQIDRL	0,6426
B44	HLA-B*40:39	989	AEVQIDRL	0,757
B44	HLA-B*40:39	989	AEVQIDRLI	0,8286
B44	HLA-B*40:39	1016	AEIRASANL	0,9217
B44	HLA-B*40:39	1181	KEIDRLNEV	0,944
B44	HLA-B*40:39	1261	SEPVLKGVKL	0,621
B44	HLA-B*40:40	168	FEYVSQPFL	0,7627
B44	HLA-B*40:40	464	FERDISTEI	0,7123
B44	HLA-B*40:40	852	AQKFNGLTVL	0,5879
B44	HLA-B*40:40	1016	AEIRASANL	0,7906
B44	HLA-B*40:40	1181	KEIDRLNEV	0,8178
B44	HLA-B*40:49	168	FEYVSQPFL	0,7985
B44	HLA-B*40:49	689	SQSIIAYTM	0,587
B44	HLA-B*40:49	852	AQKFNGLTVL	0,6038

B44	HLA-B*40:49	1016	AEIRASANL	0,8824
B44	HLA-B*40:50	168	FEYVSQPFL	0,7931
B44	HLA-B*40:50	464	FERDISTEI	0,8322
B44	HLA-B*40:50	724	TEILPVSM	0,5961
B44	HLA-B*40:50	989	AEVQIDRLI	0,69
B44	HLA-B*40:50	1016	AEIRASANL	0,8418
B44	HLA-B*40:50	1181	KEIDRLNEV	0,9058
B44	HLA-B*40:53	155	SEFRVYSSA	0,7601
B44	HLA-B*40:53	168	FEYVSQPFL	0,7587
B44	HLA-B*40:53	464	FERDISTEI	0,7272
B44	HLA-B*40:53	618	TEVPVAIHA	0,7591
B44	HLA-B*40:53	660	YECDIPIGA	0,6484
B44	HLA-B*40:53	818	IEDLLFNKV	0,6059
B44	HLA-B*40:53	1016	AEIRASANL	0,6441
B44	HLA-B*40:53	1181	KEIDRLNEV	0,765
B44	HLA-B*40:54	168	FEYVSQPFL	0,9485
B44	HLA-B*40:54	168	FEYVSQPFLM	0,5398
B44	HLA-B*40:54	339	GEVFNATRF	0,8888
B44	HLA-B*40:54	464	FERDISTEI	0,9094
B44	HLA-B*40:54	660	YECDIPIGAGI	0,5931
B44	HLA-B*40:54	818	IEDLLFNKV	0,639
B44	HLA-B*40:54	987	VEAEVQIDRL	0,8709
B44	HLA-B*40:54	989	AEVQIDRLI	0,9071
B44	HLA-B*40:54	1016	AEIRASANL	0,9748
B44	HLA-B*40:54	1181	KEIDRLNEV	0,906
B44	HLA-B*40:54	1194	NESLIDLQEL	0,5405
B44	HLA-B*40:54	1256	FDEDDSEPVL	0,5944
B44	HLA-B*40:54	1261	SEPVLKGVKL	0,736
B44	HLA-B*40:55	168	FEYVSQPFL	0,9485
B44	HLA-B*40:55	168	FEYVSQPFLM	0,5398
B44	HLA-B*40:55	339	GEVFNATRF	0,8888
B44	HLA-B*40:55	464	FERDISTEI	0,9094
B44	HLA-B*40:55	660	YECDIPIGAGI	0,5931
B44	HLA-B*40:55	818	IEDLLFNKV	0,639
B44	HLA-B*40:55	987	VEAEVQIDRL	0,8709
B44	HLA-B*40:55	989	AEVQIDRLI	0,9071
B44	HLA-B*40:55	1016	AEIRASANL	0,9748
B44	HLA-B*40:55	1181	KEIDRLNEV	0,906
B44	HLA-B*40:55	1194	NESLIDLQEL	0,5405
B44	HLA-B*40:55	1256	FDEDDSEPVL	0,5944
B44	HLA-B*40:55	1261	SEPVLKGVKL	0,736
B44	HLA-B*40:56	155	SEFRVYSSA	0,652
B44	HLA-B*40:56	168	FEYVSQPFL	0,8998
B44	HLA-B*40:56	190	REFVFKNI	0,6104
B44	HLA-B*40:56	339	GEVFNATRF	0,776
B44	HLA-B*40:56	464	FERDISTEI	0,8995
B44	HLA-B*40:56	660	YECDIPIGA	0,512
B44	HLA-B*40:56	724	TEILPVSM	0,8512
B44	HLA-B*40:56	818	IEDLLFNKV	0,6624
B44	HLA-B*40:56	987	VEAEVQIDRL	0,7072

B44	HLA-B*40:56	989	AEVQIDRL	0,7511
B44	HLA-B*40:56	989	AEVQIDRLI	0,8417
B44	HLA-B*40:56	1016	AEIRASANL	0,9365
B44	HLA-B*40:56	1181	KEIDRLNEV	0,9443
B44	HLA-B*40:56	1261	SEPVLKGVKL	0,654
B44	HLA-B*40:57	155	SEFRVYSSA	0,652
B44	HLA-B*40:57	168	FEYVSQPFL	0,8998
B44	HLA-B*40:57	190	REFVFKNI	0,6104
B44	HLA-B*40:57	339	GEVFNATRF	0,776
B44	HLA-B*40:57	464	FERDISTEI	0,8995
B44	HLA-B*40:57	660	YECDIPIGA	0,512
B44	HLA-B*40:57	724	TEILPVSM	0,8512
B44	HLA-B*40:57	818	IEDLLFNKV	0,6624
B44	HLA-B*40:57	987	VEAEVQIDRL	0,7072
B44	HLA-B*40:57	989	AEVQIDRL	0,7511
B44	HLA-B*40:57	989	AEVQIDRLI	0,8417
B44	HLA-B*40:57	1016	AEIRASANL	0,9365
B44	HLA-B*40:57	1181	KEIDRLNEV	0,9443
B44	HLA-B*40:57	1261	SEPVLKGVKL	0,654
B44	HLA-B*41:02	155	SEFRVYSSA	0,6407
B44	HLA-B*41:02	168	FEYVSQPFL	0,8406
B44	HLA-B*41:02	190	REFVFKNI	0,6992
B44	HLA-B*41:02	339	GEVFNATRF	0,7419
B44	HLA-B*41:02	464	FERDISTEI	0,9149
B44	HLA-B*41:02	724	TEILPVSM	0,8338
B44	HLA-B*41:02	818	IEDLLFNKV	0,7103
B44	HLA-B*41:02	987	VEAEVQIDRL	0,6717
B44	HLA-B*41:02	989	AEVQIDRL	0,7643
B44	HLA-B*41:02	989	AEVQIDRLI	0,847
B44	HLA-B*41:02	1016	AEIRASANL	0,9273
B44	HLA-B*41:02	1181	KEIDRLNEV	0,9555
B44	HLA-B*41:02	1261	SEPVLKGVKL	0,7634
B44	HLA-B*41:03	168	FEYVSQPFL	0,8113
B44	HLA-B*41:03	339	GEVFNATRF	0,7437
B44	HLA-B*41:03	464	FERDISTEI	0,8985
B44	HLA-B*41:03	724	TEILPVSM	0,7399
B44	HLA-B*41:03	818	IEDLLFNKV	0,706
B44	HLA-B*41:03	987	VEAEVQIDRL	0,6522
B44	HLA-B*41:03	989	AEVQIDRL	0,6693
B44	HLA-B*41:03	989	AEVQIDRLI	0,8767
B44	HLA-B*41:03	1016	AEIRASANL	0,9094
B44	HLA-B*41:03	1181	KEIDRLNEV	0,9207
B44	HLA-B*41:03	1261	SEPVLKGVKL	0,6936
B44	HLA-B*44:02	95	TEKSNIIRGW	0,9813
B44	HLA-B*44:02	153	MESEFRVY	0,5131
B44	HLA-B*44:02	297	SETKCTLKSF	0,7692
B44	HLA-B*44:02	339	GEVFNATRF	0,8968
B44	HLA-B*44:02	779	QEVFAQVKQIY	0,7113
B44	HLA-B*44:02	989	AEVQIDRLI	0,9059
B44	HLA-B*44:02	1016	AEIRASANL	0,8002

B44	HLA-B*44:02	1091	REGVFVSNGTHW	0,792
B44	HLA-B*44:02	1201	QELGKYEQY	0,9415
B44	HLA-B*44:02	1201	QELGKYEQYIKW	0,7097
B44	HLA-B*44:02	1206	YEQYIKWPW	0,7519
B44	HLA-B*44:03	95	TEKSNIIRGW	0,9553
B44	HLA-B*44:03	153	MESEFRVY	0,5502
B44	HLA-B*44:03	297	SETKCTLKSF	0,7221
B44	HLA-B*44:03	339	GEVFNATRF	0,9332
B44	HLA-B*44:03	747	TECSNLLLQY	0,6025
B44	HLA-B*44:03	779	QEVFAQVKQIY	0,7054
B44	HLA-B*44:03	989	AEVQIDRLI	0,922
B44	HLA-B*44:03	1016	AEIRASANL	0,8443
B44	HLA-B*44:03	1091	REGVFVSNGTHW	0,7134
B44	HLA-B*44:03	1201	QELGKYEQY	0,9687
B44	HLA-B*44:03	1201	QELGKYEQYIKW	0,6195
B44	HLA-B*44:03	1206	YEQYIKWPW	0,671
B44	HLA-B*44:04	95	TEKSNIIRGW	0,9072
B44	HLA-B*44:04	297	SETKCTLKSF	0,6094
B44	HLA-B*44:04	339	GEVFNATRF	0,8061
B44	HLA-B*44:04	747	TECSNLLLQY	0,5668
B44	HLA-B*44:04	989	AEVQIDRLI	0,7773
B44	HLA-B*44:04	1016	AEIRASANL	0,6706
B44	HLA-B*44:04	1091	REGVFVSNGTHW	0,6043
B44	HLA-B*44:04	1201	QELGKYEQY	0,8836
B44	HLA-B*44:04	1206	YEQYIKWPW	0,6802
B44	HLA-B*44:07	95	TEKSNIIRGW	0,9553
B44	HLA-B*44:07	153	MESEFRVY	0,5502
B44	HLA-B*44:07	297	SETKCTLKSF	0,7221
B44	HLA-B*44:07	339	GEVFNATRF	0,9332
B44	HLA-B*44:07	747	TECSNLLLQY	0,6025
B44	HLA-B*44:07	779	QEVFAQVKQIY	0,7054
B44	HLA-B*44:07	989	AEVQIDRLI	0,922
B44	HLA-B*44:07	1016	AEIRASANL	0,8443
B44	HLA-B*44:07	1091	REGVFVSNGTHW	0,7134
B44	HLA-B*44:07	1201	QELGKYEQY	0,9687
B44	HLA-B*44:07	1201	QELGKYEQYIKW	0,6195
B44	HLA-B*44:07	1206	YEQYIKWPW	0,671
B44	HLA-B*44:13	95	TEKSNIIRGW	0,9553
B44	HLA-B*44:13	153	MESEFRVY	0,5502
B44	HLA-B*44:13	297	SETKCTLKSF	0,7221
B44	HLA-B*44:13	339	GEVFNATRF	0,9332
B44	HLA-B*44:13	747	TECSNLLLQY	0,6025
B44	HLA-B*44:13	779	QEVFAQVKQIY	0,7054
B44	HLA-B*44:13	989	AEVQIDRLI	0,922
B44	HLA-B*44:13	1016	AEIRASANL	0,8443
B44	HLA-B*44:13	1091	REGVFVSNGTHW	0,7134
B44	HLA-B*44:13	1201	QELGKYEQY	0,9687
B44	HLA-B*44:13	1201	QELGKYEQYIKW	0,6195
B44	HLA-B*44:13	1206	YEQYIKWPW	0,671
B44	HLA-B*44:16	95	TEKSNIIRGW	0,8679

B44	HLA-B*44:16	153	MESEFRVY	0,5269
B44	HLA-B*44:16	190	REFVFKNIDGY	0,5278
B44	HLA-B*44:16	297	SETKCTLKSF	0,5822
B44	HLA-B*44:16	339	GEVFNATRF	0,8995
B44	HLA-B*44:16	464	FERDISTEIY	0,6219
B44	HLA-B*44:16	653	AEHVNNSY	0,6553
B44	HLA-B*44:16	747	TECSNLLLQY	0,5015
B44	HLA-B*44:16	779	QEVFAQVKQIY	0,5029
B44	HLA-B*44:16	829	ADAGFIKQY	0,8245
B44	HLA-B*44:16	917	YENQKLIANQF	0,5047
B44	HLA-B*44:16	989	AEVQIDRLI	0,7927
B44	HLA-B*44:16	1016	AEIRASANL	0,745
B44	HLA-B*44:16	1091	REGVFVSNGTHW	0,7416
B44	HLA-B*44:16	1181	KEIDRLNEV	0,6515
B44	HLA-B*44:16	1201	QELGKYEQY	0,8678
B44	HLA-B*44:16	1201	QELGKYEQYIKW	0,5106
B44	HLA-B*44:16	1206	YEQYIKWPW	0,6551
B44	HLA-B*44:21	95	TEKSNIIRGW	0,94
B44	HLA-B*44:21	297	SETKCTLKSF	0,6607
B44	HLA-B*44:21	339	GEVFNATRF	0,8236
B44	HLA-B*44:21	989	AEVQIDRLI	0,8099
B44	HLA-B*44:21	1016	AEIRASANL	0,6996
B44	HLA-B*44:21	1091	REGVFVSNGTHW	0,682
B44	HLA-B*44:21	1201	QELGKYEQY	0,8862
B44	HLA-B*44:21	1201	QELGKYEQYIKW	0,6123
B44	HLA-B*44:21	1206	YEQYIKWPW	0,6936
B44	HLA-B*44:22	95	TEKSNIIRGW	0,9813
B44	HLA-B*44:22	153	MESEFRVY	0,5131
B44	HLA-B*44:22	297	SETKCTLKSF	0,7692
B44	HLA-B*44:22	339	GEVFNATRF	0,8968
B44	HLA-B*44:22	779	QEVFAQVKQIY	0,7113
B44	HLA-B*44:22	989	AEVQIDRLI	0,9059
B44	HLA-B*44:22	1016	AEIRASANL	0,8002
B44	HLA-B*44:22	1091	REGVFVSNGTHW	0,792
B44	HLA-B*44:22	1201	QELGKYEQY	0,9415
B44	HLA-B*44:22	1201	QELGKYEQYIKW	0,7097
B44	HLA-B*44:22	1206	YEQYIKWPW	0,7519
B44	HLA-B*44:24	95	TEKSNIIRGW	0,9813
B44	HLA-B*44:24	153	MESEFRVY	0,5131
B44	HLA-B*44:24	297	SETKCTLKSF	0,7692
B44	HLA-B*44:24	339	GEVFNATRF	0,8968
B44	HLA-B*44:24	779	QEVFAQVKQIY	0,7113
B44	HLA-B*44:24	989	AEVQIDRLI	0,9059
B44	HLA-B*44:24	1016	AEIRASANL	0,8002
B44	HLA-B*44:24	1091	REGVFVSNGTHW	0,792
B44	HLA-B*44:24	1201	QELGKYEQY	0,9415
B44	HLA-B*44:24	1201	QELGKYEQYIKW	0,7097
B44	HLA-B*44:24	1206	YEQYIKWPW	0,7519
B44	HLA-B*44:26	95	TEKSNIIRGW	0,9553
B44	HLA-B*44:26	153	MESEFRVY	0,5502

B44	HLA-B*44:26	297	SETKCTLKSF	0,7221
B44	HLA-B*44:26	339	GEVFNATRF	0,9332
B44	HLA-B*44:26	747	TECSNLLLQY	0,6025
B44	HLA-B*44:26	779	QEVFAQVKQIY	0,7054
B44	HLA-B*44:26	989	AEVQIDRLI	0,922
B44	HLA-B*44:26	1016	AEIRASANL	0,8443
B44	HLA-B*44:26	1091	REGVFVSNGTHW	0,7134
B44	HLA-B*44:26	1201	QELGKYEQY	0,9687
B44	HLA-B*44:26	1201	QELGKYEQYIKW	0,6195
B44	HLA-B*44:26	1206	YEQYIKWPW	0,671
B44	HLA-B*44:27	95	TEKSNIIRGW	0,9813
B44	HLA-B*44:27	153	MESEFRVY	0,5131
B44	HLA-B*44:27	297	SETKCTLKSF	0,7692
B44	HLA-B*44:27	339	GEVFNATRF	0,8968
B44	HLA-B*44:27	779	QEVFAQVKQIY	0,7113
B44	HLA-B*44:27	989	AEVQIDRLI	0,9059
B44	HLA-B*44:27	1016	AEIRASANL	0,8002
B44	HLA-B*44:27	1091	REGVFVSNGTHW	0,792
B44	HLA-B*44:27	1201	QELGKYEQY	0,9415
B44	HLA-B*44:27	1201	QELGKYEQYIKW	0,7097
B44	HLA-B*44:27	1206	YEQYIKWPW	0,7519
B44	HLA-B*44:28	95	TEKSNIIRGW	0,9464
B44	HLA-B*44:28	297	SETKCTLKSF	0,6716
B44	HLA-B*44:28	339	GEVFNATRF	0,8558
B44	HLA-B*44:28	747	TECSNLLLQY	0,5764
B44	HLA-B*44:28	989	AEVQIDRLI	0,8322
B44	HLA-B*44:28	1016	AEIRASANL	0,7302
B44	HLA-B*44:28	1091	REGVFVSNGTHW	0,6683
B44	HLA-B*44:28	1201	QELGKYEQY	0,92
B44	HLA-B*44:28	1201	QELGKYEQYIKW	0,5544
B44	HLA-B*44:28	1206	YEQYIKWPW	0,7125
B44	HLA-B*44:29	95	TEKSNIIRGW	0,9629
B44	HLA-B*44:29	153	MESEFRVY	0,6747
B44	HLA-B*44:29	190	REFVFKNIDGY	0,5841
B44	HLA-B*44:29	297	SETKCTLKSF	0,8275
B44	HLA-B*44:29	339	GEVFNATRF	0,9564
B44	HLA-B*44:29	464	FERDISTEIY	0,7133
B44	HLA-B*44:29	653	AEHVNNSY	0,8118
B44	HLA-B*44:29	747	TECSNLLLQY	0,7163
B44	HLA-B*44:29	779	QEVFAQVKQIY	0,8073
B44	HLA-B*44:29	829	ADAGFIKQY	0,8988
B44	HLA-B*44:29	917	YENQKLIANQF	0,5716
B44	HLA-B*44:29	989	AEVQIDRLI	0,9447
B44	HLA-B*44:29	1016	AEIRASANL	0,8969
B44	HLA-B*44:29	1091	REGVFVSNGTHW	0,8063
B44	HLA-B*44:29	1201	QELGKYEQY	0,9796
B44	HLA-B*44:29	1201	QELGKYEQYIKW	0,6973
B44	HLA-B*44:29	1206	YEQYIKWPW	0,7407
B44	HLA-B*44:30	95	TEKSNIIRGW	0,9553
B44	HLA-B*44:30	153	MESEFRVY	0,5502

B44	HLA-B*44:30	297	SETKCTLKSF	0,7221
B44	HLA-B*44:30	339	GEVFNATRF	0,9332
B44	HLA-B*44:30	747	TECSNLLLQY	0,6025
B44	HLA-B*44:30	779	QEVFAQVKQIY	0,7054
B44	HLA-B*44:30	989	AEVQIDRLI	0,922
B44	HLA-B*44:30	1016	AEIRASANL	0,8443
B44	HLA-B*44:30	1091	REGVFVSNGTHW	0,7134
B44	HLA-B*44:30	1201	QELGKYEQY	0,9687
B44	HLA-B*44:30	1201	QELGKYEQYIKW	0,6195
B44	HLA-B*44:30	1206	YEQYIKWPW	0,671
B44	HLA-B*44:32	95	TEKSNIIRGW	0,938
B44	HLA-B*44:32	297	SETKCTLKSF	0,6498
B44	HLA-B*44:32	339	GEVFNATRF	0,8844
B44	HLA-B*44:32	747	TECSNLLLQY	0,513
B44	HLA-B*44:32	779	QEVFAQVKQIY	0,6238
B44	HLA-B*44:32	989	AEVQIDRLI	0,8801
B44	HLA-B*44:32	1016	AEIRASANL	0,796
B44	HLA-B*44:32	1091	REGVFVSNGTHW	0,7563
B44	HLA-B*44:32	1201	QELGKYEQY	0,9492
B44	HLA-B*44:32	1201	QELGKYEQYIKW	0,5458
B44	HLA-B*44:32	1206	YEQYIKWPW	0,6216
B44	HLA-B*44:33	95	TEKSNIIRGW	0,9813
B44	HLA-B*44:33	153	MESEFRVY	0,5131
B44	HLA-B*44:33	297	SETKCTLKSF	0,7692
B44	HLA-B*44:33	339	GEVFNATRF	0,8968
B44	HLA-B*44:33	779	QEVFAQVKQIY	0,7113
B44	HLA-B*44:33	989	AEVQIDRLI	0,9059
B44	HLA-B*44:33	1016	AEIRASANL	0,8002
B44	HLA-B*44:33	1091	REGVFVSNGTHW	0,792
B44	HLA-B*44:33	1201	QELGKYEQY	0,9415
B44	HLA-B*44:33	1201	QELGKYEQYIKW	0,7097
B44	HLA-B*44:33	1206	YEQYIKWPW	0,7519
B44	HLA-B*44:35	95	TEKSNIIRGW	0,975
B44	HLA-B*44:35	153	MESEFRVY	0,5617
B44	HLA-B*44:35	297	SETKCTLKSF	0,7874
B44	HLA-B*44:35	339	GEVFNATRF	0,9155
B44	HLA-B*44:35	747	TECSNLLLQY	0,5932
B44	HLA-B*44:35	779	QEVFAQVKQIY	0,7582
B44	HLA-B*44:35	989	AEVQIDRLI	0,9053
B44	HLA-B*44:35	1016	AEIRASANL	0,8196
B44	HLA-B*44:35	1091	REGVFVSNGTHW	0,782
B44	HLA-B*44:35	1201	QELGKYEQY	0,9534
B44	HLA-B*44:35	1201	QELGKYEQYIKW	0,6824
B44	HLA-B*44:35	1206	YEQYIKWPW	0,7492
B44	HLA-B*44:36	95	TEKSNIIRGW	0,9553
B44	HLA-B*44:36	153	MESEFRVY	0,5502
B44	HLA-B*44:36	297	SETKCTLKSF	0,7221
B44	HLA-B*44:36	339	GEVFNATRF	0,9332
B44	HLA-B*44:36	747	TECSNLLLQY	0,6025
B44	HLA-B*44:36	779	QEVFAQVKQIY	0,7054

B44	HLA-B*44:36	989	AEVQIDRLI	0,922
B44	HLA-B*44:36	1016	AEIRASANL	0,8443
B44	HLA-B*44:36	1091	REGVFVSNGTHW	0,7134
B44	HLA-B*44:36	1201	QELGKYEQY	0,9687
B44	HLA-B*44:36	1201	QELGKYEQYIKW	0,6195
B44	HLA-B*44:36	1206	YEQYIKWPW	0,671
B44	HLA-B*44:37	95	TEKSNIIRGW	0,826
B44	HLA-B*44:37	153	MESEFRVY	0,5576
B44	HLA-B*44:37	190	REFVFKNIDGY	0,5135
B44	HLA-B*44:37	339	GEVFNATRF	0,9143
B44	HLA-B*44:37	464	FERDISTEIY	0,7083
B44	HLA-B*44:37	829	ADAGFIKQY	0,8697
B44	HLA-B*44:37	989	AEVQIDRLI	0,7629
B44	HLA-B*44:37	1016	AEIRASANL	0,7138
B44	HLA-B*44:37	1091	REGVFVSNGTHW	0,6777
B44	HLA-B*44:37	1201	QELGKYEQY	0,902
B44	HLA-B*44:37	1206	YEQYIKWPW	0,5676
B44	HLA-B*44:38	95	TEKSNIIRGW	0,9553
B44	HLA-B*44:38	153	MESEFRVY	0,5502
B44	HLA-B*44:38	297	SETKCTLKSF	0,7221
B44	HLA-B*44:38	339	GEVFNATRF	0,9332
B44	HLA-B*44:38	747	TECSNLLLQY	0,6025
B44	HLA-B*44:38	779	QEVFAQVKQIY	0,7054
B44	HLA-B*44:38	989	AEVQIDRLI	0,922
B44	HLA-B*44:38	1016	AEIRASANL	0,8443
B44	HLA-B*44:38	1091	REGVFVSNGTHW	0,7134
B44	HLA-B*44:38	1201	QELGKYEQY	0,9687
B44	HLA-B*44:38	1201	QELGKYEQYIKW	0,6195
B44	HLA-B*44:38	1206	YEQYIKWPW	0,671
B44	HLA-B*45:01	153	MESEFRVYS	0,6419
B44	HLA-B*45:01	155	SEFRVYSSA	0,8907
B44	HLA-B*45:01	280	NENGTITDA	0,7132
B44	HLA-B*45:01	618	TEVPVAIHA	0,9036
B44	HLA-B*45:01	867	DEMIAQYTSA	0,5083
B44	HLA-B*45:01	1016	AEIRASANL	0,678
B44	HLA-B*45:01	1016	AEIRASANLA	0,8233
B44	HLA-B*45:01	1016	AEIRASANLAA	0,6215
B44	HLA-B*45:01	1071	QEKNFTTAP	0,5423
B44	HLA-B*45:01	1071	QEKNFTTAPA	0,5531
B44	HLA-B*45:01	1181	KEIDRLNEV	0,7707
B44	HLA-B*45:01	1181	KEIDRLNEVA	0,6837
B44	HLA-B*45:03	153	MESEFRVYS	0,6419
B44	HLA-B*45:03	155	SEFRVYSSA	0,8907
B44	HLA-B*45:03	280	NENGTITDA	0,7132
B44	HLA-B*45:03	618	TEVPVAIHA	0,9036
B44	HLA-B*45:03	867	DEMIAQYTSA	0,5083
B44	HLA-B*45:03	1016	AEIRASANL	0,678
B44	HLA-B*45:03	1016	AEIRASANLA	0,8233
B44	HLA-B*45:03	1016	AEIRASANLAA	0,6215
B44	HLA-B*45:03	1071	QEKNFTTAP	0,5423

B44	HLA-B*45:03	1071	QEKNFTTAPA	0,5531
B44	HLA-B*45:03	1181	KEIDRLNEV	0,7707
B44	HLA-B*45:03	1181	KEIDRLNEVA	0,6837
B44	HLA-B*45:04	153	MESEFRVYS	0,6668
B44	HLA-B*45:04	155	SEFRVYSSA	0,8728
B44	HLA-B*45:04	168	FEYVSQPFL	0,6477
B44	HLA-B*45:04	280	NENGTITDA	0,6169
B44	HLA-B*45:04	339	GEVFNATRFA	0,5181
B44	HLA-B*45:04	464	FERDISTEI	0,7909
B44	HLA-B*45:04	515	FELLHAPAT	0,6115
B44	HLA-B*45:04	618	TEVPVAIHA	0,9037
B44	HLA-B*45:04	660	YECDIPIGA	0,7754
B44	HLA-B*45:04	724	TEILPVSMT	0,6427
B44	HLA-B*45:04	818	IEDLLFNKV	0,696
B44	HLA-B*45:04	989	AEVQIDRLI	0,7778
B44	HLA-B*45:04	1016	AEIRASANL	0,7431
B44	HLA-B*45:04	1016	AEIRASANLA	0,7358
B44	HLA-B*45:04	1016	AEIRASANLAA	0,6111
B44	HLA-B*45:04	1181	KEIDRLNEV	0,8925
B44	HLA-B*45:04	1181	KEIDRLNEVA	0,783
B44	HLA-B*45:05	153	MESEFRVYS	0,7073
B44	HLA-B*45:05	155	SEFRVYSSA	0,887
B44	HLA-B*45:05	280	NENGTITDA	0,7827
B44	HLA-B*45:05	280	NENGTITDAV	0,5501
B44	HLA-B*45:05	339	GEVFNATRFA	0,5214
B44	HLA-B*45:05	618	TEVPVAIHA	0,9095
B44	HLA-B*45:05	660	YECDIPIGA	0,6478
B44	HLA-B*45:05	867	DEMIAQYTS	0,5654
B44	HLA-B*45:05	867	DEMIAQYTSA	0,6146
B44	HLA-B*45:05	989	AEVQIDRLI	0,7684
B44	HLA-B*45:05	1016	AEIRASANL	0,6972
B44	HLA-B*45:05	1016	AEIRASANLA	0,8381
B44	HLA-B*45:05	1016	AEIRASANLAA	0,6471
B44	HLA-B*45:05	1071	QEKNFTTAP	0,638
B44	HLA-B*45:05	1071	QEKNFTTAPA	0,6366
B44	HLA-B*45:05	1181	KEIDRLNEV	0,7759
B44	HLA-B*45:05	1181	KEIDRLNEVA	0,7116
B44	HLA-B*45:07	153	MESEFRVYS	0,6419
B44	HLA-B*45:07	155	SEFRVYSSA	0,8907
B44	HLA-B*45:07	280	NENGTITDA	0,7132
B44	HLA-B*45:07	618	TEVPVAIHA	0,9036
B44	HLA-B*45:07	867	DEMIAQYTSA	0,5083
B44	HLA-B*45:07	1016	AEIRASANL	0,678
B44	HLA-B*45:07	1016	AEIRASANLA	0,8233
B44	HLA-B*45:07	1016	AEIRASANLAA	0,6215
B44	HLA-B*45:07	1071	QEKNFTTAP	0,5423
B44	HLA-B*45:07	1071	QEKNFTTAPA	0,5531
B44	HLA-B*45:07	1181	KEIDRLNEV	0,7707
B44	HLA-B*45:07	1181	KEIDRLNEVA	0,6837
B44	HLA-B*49:04	339	GEVFNATRF	0,7684

B44	HLA-B*49:04	464	FERDISTEI	0,6468
B44	HLA-B*49:04	989	AEVQIDRLI	0,7729
B44	HLA-B*50:01	153	MESEFRVYS	0,5279
B44	HLA-B*50:01	155	SEFRVYSSA	0,9313
B44	HLA-B*50:01	168	FEYVSQPFL	0,5682
B44	HLA-B*50:01	464	FERDISTEI	0,8164
B44	HLA-B*50:01	515	FELLHAPAT	0,5692
B44	HLA-B*50:01	618	TEVPVAIHA	0,8958
B44	HLA-B*50:01	660	YECDIPIGA	0,7228
B44	HLA-B*50:01	1016	AEIRASANL	0,635
B44	HLA-B*50:01	1016	AEIRASANLA	0,6321
B44	HLA-B*50:01	1181	KEIDRLNEV	0,8625
B44	HLA-B*50:01	1181	KEIDRLNEVA	0,7661
B44	HLA-B*50:02	155	SEFRVYSSA	0,8534
B44	HLA-B*50:02	280	NENGTITDA	0,5939
B44	HLA-B*50:02	618	TEVPVAIHA	0,8364
B44	HLA-B*50:02	1016	AEIRASANLA	0,6537
B44	HLA-B*50:02	1181	KEIDRLNEVA	0,5821
B44	HLA-B*50:04	153	MESEFRVYS	0,5279
B44	HLA-B*50:04	155	SEFRVYSSA	0,9313
B44	HLA-B*50:04	168	FEYVSQPFL	0,5682
B44	HLA-B*50:04	464	FERDISTEI	0,8164
B44	HLA-B*50:04	515	FELLHAPAT	0,5692
B44	HLA-B*50:04	618	TEVPVAIHA	0,8958
B44	HLA-B*50:04	660	YECDIPIGA	0,7228
B44	HLA-B*50:04	1016	AEIRASANL	0,635
B44	HLA-B*50:04	1016	AEIRASANLA	0,6321
B44	HLA-B*50:04	1181	KEIDRLNEV	0,8625
B44	HLA-B*50:04	1181	KEIDRLNEVA	0,7661
B58	HLA-B*15:16	34	RGVYYPDKVF	0,6031
B58	HLA-B*15:16	50	STQDLFLPF	0,5575
B58	HLA-B*15:16	160	YSSANNCTF	0,7436
B58	HLA-B*15:16	258	WTAGAAAYY	0,6769
B58	HLA-B*15:16	267	VGYLQPRTF	0,6255
B58	HLA-B*15:16	304	KSFTVEKGI	0,6627
B58	HLA-B*15:16	344	ATRFASVYAW	0,5424
B58	HLA-B*15:16	372	ASFSTFKCY	0,5797
B58	HLA-B*15:16	392	FTNVYADSF	0,6268
B58	HLA-B*15:16	590	CSFGGVSVI	0,5898
B58	HLA-B*15:16	604	TSNQVAVLY	0,7291
B58	HLA-B*15:16	634	RVYSTGSNV	0,5284
B58	HLA-B*15:16	634	RVYSTGSNVF	0,7452
B58	HLA-B*15:16	685	RSVASQSII	0,7268
B58	HLA-B*15:16	687	VASQSIIAY	0,7282
B58	HLA-B*15:16	710	NSIAIPTNF	0,7782
B58	HLA-B*15:16	712	IAIPTNFTI	0,8768
B58	HLA-B*15:16	718	FTISVTTEI	0,7002
B58	HLA-B*15:16	733	KTSVDCTMY	0,5204
B58	HLA-B*15:16	814	KRSFIEDLLF	0,6401
B58	HLA-B*15:16	815	RSFIEDLLF	0,9347

B58	HLA-B*15:16	825	KVTLADAGF	0,5684
B58	HLA-B*15:16	865	LTDEMIAQY	0,7522
B58	HLA-B*15:16	878	LAGTITSGW	0,5789
B58	HLA-B*15:16	879	AGTITSGWTF	0,542
B58	HLA-B*15:16	880	GTITSGWTF	0,8294
B58	HLA-B*15:16	886	WTFGAGAAL	0,5705
B58	HLA-B*15:16	898	FAMQMAYRF	0,655
B58	HLA-B*15:16	923	IANQFNSAI	0,5744
B58	HLA-B*15:16	1005	QTYVTQQLI	0,6943
B58	HLA-B*15:16	1054	QSAPHGVVF	0,8856
B58	HLA-B*15:16	1086	KAHFPREGVF	0,5268
B58	HLA-B*15:16	1093	GVFVSNGTHW	0,5731
B58	HLA-B*15:17	21	RTQLPPAY	0,729
B58	HLA-B*15:17	28	YTNSFTRGVY	0,712
B58	HLA-B*15:17	29	TNSFTRGVYY	0,6428
B58	HLA-B*15:17	30	NSFTRGVYY	0,8886
B58	HLA-B*15:17	34	RGVYYPDKVF	0,7986
B58	HLA-B*15:17	35	GVYYPDKVF	0,8911
B58	HLA-B*15:17	50	STQDLFLPF	0,7926
B58	HLA-B*15:17	158	RVYSSANNCTF	0,6804
B58	HLA-B*15:17	160	YSSANNCTF	0,8168
B58	HLA-B*15:17	192	FVFKNIDGY	0,8376
B58	HLA-B*15:17	204	YSKHTPINL	0,7655
B58	HLA-B*15:17	212	LVRDLPQGF	0,867
B58	HLA-B*15:17	257	GWTAGAAAYY	0,6825
B58	HLA-B*15:17	258	WTAGAAAYY	0,9236
B58	HLA-B*15:17	261	GAAAYYVGY	0,8951
B58	HLA-B*15:17	267	VGYLQPRTF	0,7451
B58	HLA-B*15:17	304	KSFTVEKGI	0,6634
B58	HLA-B*15:17	304	KSFTVEKGIY	0,7603
B58	HLA-B*15:17	310	KGIYQTSNF	0,719
B58	HLA-B*15:17	344	ATRFASVYAW	0,7702
B58	HLA-B*15:17	361	CVADYSVLY	0,6496
B58	HLA-B*15:17	366	SVLYNSASF	0,8204
B58	HLA-B*15:17	372	ASFSTFKCY	0,828
B58	HLA-B*15:17	392	FTNVYADSF	0,7806
B58	HLA-B*15:17	604	TSNQVAVLY	0,9455
B58	HLA-B*15:17	625	HADQLTPTW	0,846
B58	HLA-B*15:17	634	RVYSTGSNV	0,6546
B58	HLA-B*15:17	634	RVYSTGSNVF	0,9115
B58	HLA-B*15:17	685	RSVASQSII	0,7537
B58	HLA-B*15:17	687	VASQSIIAY	0,9477
B58	HLA-B*15:17	710	NSIAIPTNF	0,9195
B58	HLA-B*15:17	712	IAIPTNFTI	0,867
B58	HLA-B*15:17	718	FTISVTTEI	0,7184
B58	HLA-B*15:17	733	KTSVDCTMY	0,8517
B58	HLA-B*15:17	814	KRSFIEDLLF	0,7282
B58	HLA-B*15:17	815	RSFIEDLLF	0,9846
B58	HLA-B*15:17	825	KVTLADAGF	0,8775
B58	HLA-B*15:17	865	LTDEMIAQY	0,9166

B58	HLA-B*15:17	878	LAGTITSGW	0,7922
B58	HLA-B*15:17	879	AGTITSGWTF	0,7247
B58	HLA-B*15:17	880	GTITSGWTF	0,9585
B58	HLA-B*15:17	886	WTFGAGAAL	0,7745
B58	HLA-B*15:17	892	AALQIPFAM	0,6696
B58	HLA-B*15:17	898	FAMQMAYRF	0,7486
B58	HLA-B*15:17	940	STASALGKL	0,7384
B58	HLA-B*15:17	962	LVKQLSSNF	0,7867
B58	HLA-B*15:17	1005	QTYVTQQLI	0,7371
B58	HLA-B*15:17	1021	SANLAATKM	0,6738
B58	HLA-B*15:17	1054	QSAPHGVVF	0,9815
B58	HLA-B*15:17	1086	KAHFPREGVF	0,7038
B58	HLA-B*15:17	1093	GVFVSNGTHW	0,7853
B58	HLA-B*15:17	1095	FVSNGTHWF	0,6814
B58	HLA-B*15:67	34	RGVYYPDKVF	0,6031
B58	HLA-B*15:67	50	STQDLFLPF	0,5575
B58	HLA-B*15:67	160	YSSANNCTF	0,7436
B58	HLA-B*15:67	258	WTAGAAAYY	0,6769
B58	HLA-B*15:67	267	VGYLQPRTF	0,6255
B58	HLA-B*15:67	304	KSFTVEKGI	0,6627
B58	HLA-B*15:67	344	ATRFASVYAW	0,5424
B58	HLA-B*15:67	372	ASFSTFKCY	0,5797
B58	HLA-B*15:67	392	FTNVYADSF	0,6268
B58	HLA-B*15:67	590	CSFGGVSVI	0,5898
B58	HLA-B*15:67	604	TSNQVAVLY	0,7291
B58	HLA-B*15:67	634	RVYSTGSNV	0,5284
B58	HLA-B*15:67	634	RVYSTGSNVF	0,7452
B58	HLA-B*15:67	685	RSVASQSII	0,7268
B58	HLA-B*15:67	687	VASQSIIAY	0,7282
B58	HLA-B*15:67	710	NSIAIPTNF	0,7782
B58	HLA-B*15:67	712	IAIPTNFTI	0,8768
B58	HLA-B*15:67	718	FTISVTTEI	0,7002
B58	HLA-B*15:67	733	KTSVDCTMY	0,5204
B58	HLA-B*15:67	814	KRSFIEDLLF	0,6401
B58	HLA-B*15:67	815	RSFIEDLLF	0,9347
B58	HLA-B*15:67	825	KVTLADAGF	0,5684
B58	HLA-B*15:67	865	LTDEMIAQY	0,7522
B58	HLA-B*15:67	878	LAGTITSGW	0,5789
B58	HLA-B*15:67	879	AGTITSGWTF	0,542
B58	HLA-B*15:67	880	GTITSGWTF	0,8294
B58	HLA-B*15:67	886	WTFGAGAAL	0,5705
B58	HLA-B*15:67	898	FAMQMAYRF	0,655
B58	HLA-B*15:67	923	IANQFNSAI	0,5744
B58	HLA-B*15:67	1005	QTYVTQQLI	0,6943
B58	HLA-B*15:67	1054	QSAPHGVVF	0,8856
B58	HLA-B*15:67	1086	KAHFPREGVF	0,5268
B58	HLA-B*15:67	1093	GVFVSNGTHW	0,5731
B58	HLA-B*15:95	34	RGVYYPDKVF	0,5969
B58	HLA-B*15:95	50	STQDLFLPF	0,5282
B58	HLA-B*15:95	158	RVYSSANNCTF	0,5058

B58	HLA-B*15:95	160	YSSANNCTF	0,7058
B58	HLA-B*15:95	258	WTAGAAAYY	0,6382
B58	HLA-B*15:95	267	VGYLQPRTF	0,6579
B58	HLA-B*15:95	304	KSFTVEKGI	0,6907
B58	HLA-B*15:95	304	KSFTVEKGIY	0,5666
B58	HLA-B*15:95	310	KGIYQTSNF	0,5569
B58	HLA-B*15:95	344	ATRFASVYAW	0,5798
B58	HLA-B*15:95	372	ASFSTFKCY	0,6011
B58	HLA-B*15:95	392	FTNVYADSF	0,5426
B58	HLA-B*15:95	590	CSFGGVSVI	0,5003
B58	HLA-B*15:95	604	TSNQVAVLY	0,7366
B58	HLA-B*15:95	625	HADQLTPTW	0,7356
B58	HLA-B*15:95	634	RVYSTGSNVF	0,7402
B58	HLA-B*15:95	685	RSVASQSII	0,7531
B58	HLA-B*15:95	687	VASQSIIAY	0,7264
B58	HLA-B*15:95	710	NSIAIPTNF	0,7623
B58	HLA-B*15:95	712	IAIPTNFTI	0,8481
B58	HLA-B*15:95	718	FTISVTTEI	0,5787
B58	HLA-B*15:95	733	KTSVDCTMY	0,5963
B58	HLA-B*15:95	814	KRSFIEDLLF	0,6782
B58	HLA-B*15:95	815	RSFIEDLLF	0,9594
B58	HLA-B*15:95	825	KVTLADAGF	0,5677
B58	HLA-B*15:95	878	LAGTITSGW	0,5751
B58	HLA-B*15:95	879	AGTITSGWTF	0,5289
B58	HLA-B*15:95	880	GTITSGWTF	0,8368
B58	HLA-B*15:95	898	FAMQMAYRF	0,5752
B58	HLA-B*15:95	923	IANQFNSAI	0,5088
B58	HLA-B*15:95	1005	QTYVTQQLI	0,6101
B58	HLA-B*15:95	1054	QSAPHGVVF	0,8744
B58	HLA-B*15:95	1086	KAHFPREGVF	0,5522
B58	HLA-B*15:95	1093	GVFVSNGTHW	0,6012
B58	HLA-B*57:01	97	KSNIIRGW	0,8688
B58	HLA-B*57:01	304	KSFTVEKGI	0,5385
B58	HLA-B*57:01	344	ATRFASVYAW	0,7634
B58	HLA-B*57:01	622	VAIHADQLTPTW	0,5506
B58	HLA-B*57:01	625	HADQLTPTW	0,8783
B58	HLA-B*57:01	712	IAIPTNFTI	0,5812
B58	HLA-B*57:01	815	RSFIEDLLF	0,868
B58	HLA-B*57:01	878	LAGTITSGW	0,7806
B58	HLA-B*57:01	880	GTITSGWTF	0,7834
B58	HLA-B*57:01	1093	GVFVSNGTHW	0,7168
B58	HLA-B*57:02	160	YSSANNCTF	0,5613
B58	HLA-B*57:02	249	LTPGDSSSGW	0,5051
B58	HLA-B*57:02	344	ATRFASVYAW	0,6112
B58	HLA-B*57:02	622	VAIHADQLTPTW	0,6009
B58	HLA-B*57:02	624	IHADQLTPTW	0,6898
B58	HLA-B*57:02	625	HADQLTPTW	0,9339
B58	HLA-B*57:02	712	IAIPTNFTI	0,6703
B58	HLA-B*57:02	815	RSFIEDLLF	0,8365
B58	HLA-B*57:02	878	LAGTITSGW	0,7731

B58	HLA-B*57:02	880	GTITSGWTF	0,7332
B58	HLA-B*57:02	1054	QSAPHGVVF	0,8599
B58	HLA-B*57:03	97	KSNIIRGW	0,5712
B58	HLA-B*57:03	160	YSSANNCTF	0,52
B58	HLA-B*57:03	304	KSFTVEKGI	0,5693
B58	HLA-B*57:03	344	ATRFASVYAW	0,6947
B58	HLA-B*57:03	622	VAIHADQLTPTW	0,6476
B58	HLA-B*57:03	624	IHADQLTPTW	0,6328
B58	HLA-B*57:03	625	HADQLTPTW	0,9284
B58	HLA-B*57:03	634	RVYSTGSNVF	0,5104
B58	HLA-B*57:03	685	RSVASQSII	0,56
B58	HLA-B*57:03	710	NSIAIPTNF	0,7756
B58	HLA-B*57:03	712	IAIPTNFTI	0,8166
B58	HLA-B*57:03	814	KRSFIEDLLF	0,5259
B58	HLA-B*57:03	815	RSFIEDLLF	0,9134
B58	HLA-B*57:03	825	KVTLADAGF	0,5236
B58	HLA-B*57:03	878	LAGTITSGW	0,816
B58	HLA-B*57:03	880	GTITSGWTF	0,8295
B58	HLA-B*57:03	898	FAMQMAYRF	0,5657
B58	HLA-B*57:03	1054	QSAPHGVVF	0,8674
B58	HLA-B*57:03	1093	GVFVSNGTHW	0,6139
B58	HLA-B*57:07	344	ATRFASVYAW	0,5647
B58	HLA-B*57:07	344	ATRFASVYAW	0,5647
B58	HLA-B*57:07	344	ATRFASVYAW	0,5647
B58	HLA-B*57:07	344	ATRFASVYAW	0,5647
B58	HLA-B*57:07	625	HADQLTPTW	0,8776
B58	HLA-B*57:07	625	HADQLTPTW	0,8776
B58	HLA-B*57:07	625	HADQLTPTW	0,8776
B58	HLA-B*57:07	625	HADQLTPTW	0,8776
B58	HLA-B*57:07	712	IAIPTNFTI	0,6961
B58	HLA-B*57:07	712	IAIPTNFTI	0,6961
B58	HLA-B*57:07	712	IAIPTNFTI	0,6961
B58	HLA-B*57:07	712	IAIPTNFTI	0,6961
B58	HLA-B*57:07	815	RSFIEDLLF	0,7639
B58	HLA-B*57:07	815	RSFIEDLLF	0,7639
B58	HLA-B*57:07	815	RSFIEDLLF	0,7639
B58	HLA-B*57:07	815	RSFIEDLLF	0,7639
B58	HLA-B*57:07	878	LAGTITSGW	0,6899
B58	HLA-B*57:07	878	LAGTITSGW	0,6899
B58	HLA-B*57:07	878	LAGTITSGW	0,6899
B58	HLA-B*57:07	878	LAGTITSGW	0,6899
B58	HLA-B*57:07	880	GTITSGWTF	0,6913
B58	HLA-B*57:07	880	GTITSGWTF	0,6913
B58	HLA-B*57:07	880	GTITSGWTF	0,6913
B58	HLA-B*57:07	880	GTITSGWTF	0,6913
B58	HLA-B*57:07	898	FAMQMAYRF	0,5331
B58	HLA-B*57:07	898	FAMQMAYRF	0,5331
B58	HLA-B*57:07	898	FAMQMAYRF	0,5331
B58	HLA-B*57:07	898	FAMQMAYRF	0,5331
B58	HLA-B*57:08	97	KSNIIRGW	0,8688

B58	HLA-B*57:08	304	KSFTVEKGI	0,5385
B58	HLA-B*57:08	344	ATRFASVYAW	0,7634
B58	HLA-B*57:08	622	VAIHADQLTPTW	0,5506
B58	HLA-B*57:08	625	HADQLTPTW	0,8783
B58	HLA-B*57:08	712	IAIPTNFTI	0,5812
B58	HLA-B*57:08	815	RSFIEDLLF	0,868
B58	HLA-B*57:08	878	LAGTITSGW	0,7806
B58	HLA-B*57:08	880	GTITSGWTF	0,7834
B58	HLA-B*57:08	1093	GVFVSNGTHW	0,7168
B58	HLA-B*57:09	344	ATRFASVYAW	0,5174
B58	HLA-B*57:09	634	RVYSTGSNVF	0,591
B58	HLA-B*57:09	712	IAIPTNFTI	0,5816
B58	HLA-B*57:09	815	RSFIEDLLF	0,7555
B58	HLA-B*57:09	878	LAGTITSGW	0,5423
B58	HLA-B*57:09	880	GTITSGWTF	0,7045
B58	HLA-B*57:09	1054	QSAPHGVVF	0,7826
B58	HLA-B*57:09	1086	KAHFPREGVF	0,5053
B58	HLA-B*58:01	97	KSNIIRGW	0,6994
B58	HLA-B*58:01	160	YSSANNCTF	0,5597
B58	HLA-B*58:01	344	ATRFASVYAW	0,7778
B58	HLA-B*58:01	604	TSNQVAVLY	0,7554
B58	HLA-B*58:01	622	VAIHADQLTPTW	0,7607
B58	HLA-B*58:01	624	IHADQLTPTW	0,7759
B58	HLA-B*58:01	625	HADQLTPTW	0,9737
B58	HLA-B*58:01	687	VASQSIIAY	0,6802
B58	HLA-B*58:01	710	NSIAIPTNF	0,8133
B58	HLA-B*58:01	712	IAIPTNFTI	0,8079
B58	HLA-B*58:01	814	KRSFIEDLLF	0,5656
B58	HLA-B*58:01	815	RSFIEDLLF	0,9373
B58	HLA-B*58:01	878	LAGTITSGW	0,8666
B58	HLA-B*58:01	880	GTITSGWTF	0,879
B58	HLA-B*58:01	898	FAMQMAYRF	0,6403
B58	HLA-B*58:01	1054	QSAPHGVVF	0,8091
B58	HLA-B*58:01	1093	GVFVSNGTHW	0,7252
B58	HLA-B*58:02	815	RSFIEDLLF	0,6532
B58	HLA-B*58:04	56	LPFFSNVTW	0,5448
B58	HLA-B*58:04	97	KSNIIRGW	0,6084
B58	HLA-B*58:04	160	YSSANNCTF	0,5817
B58	HLA-B*58:04	344	ATRFASVYAW	0,7355
B58	HLA-B*58:04	604	TSNQVAVLY	0,7164
B58	HLA-B*58:04	622	VAIHADQLTPTW	0,7105
B58	HLA-B*58:04	624	IHADQLTPTW	0,7479
B58	HLA-B*58:04	625	HADQLTPTW	0,9681
B58	HLA-B*58:04	687	VASQSIIAY	0,648
B58	HLA-B*58:04	710	NSIAIPTNF	0,7658
B58	HLA-B*58:04	712	IAIPTNFTI	0,7552
B58	HLA-B*58:04	814	KRSFIEDLLF	0,5733
B58	HLA-B*58:04	815	RSFIEDLLF	0,9452
B58	HLA-B*58:04	878	LAGTITSGW	0,801
B58	HLA-B*58:04	880	GTITSGWTF	0,8598

B58	HLA-B*58:04	892	AALQIPFAM	0,5093
B58	HLA-B*58:04	898	FAMQMAYRF	0,6552
B58	HLA-B*58:04	1054	QSAPHGVVF	0,808
B58	HLA-B*58:04	1093	GVFVSNGTHW	0,6243
B58	HLA-B*58:06	815	RSFIEDLLF	0,7373
B58	HLA-B*58:08	62	VTWFHAIHV	0,5966
B58	HLA-B*58:08	304	KSFTVEKGI	0,7929
B58	HLA-B*58:08	590	CSFGGVSVI	0,664
B58	HLA-B*58:08	685	RSVASQSI	0,5963
B58	HLA-B*58:08	685	RSVASQSII	0,7492
B58	HLA-B*58:08	697	MSLGAENSV	0,5739
B58	HLA-B*58:08	711	SIAIPTNFTI	0,6417
B58	HLA-B*58:08	712	IAIPTNFTI	0,9331
B58	HLA-B*58:08	718	FTISVTTEI	0,7015
B58	HLA-B*58:08	815	RSFIEDLLF	0,7859
B58	HLA-B*58:08	923	IANQFNSAI	0,6908
B58	HLA-B*58:08	1005	QTYVTQQLI	0,8005
B58	HLA-B*58:09	56	LPFFSNVTW	0,6098
B58	HLA-B*58:09	97	KSNIIRGW	0,7131
B58	HLA-B*58:09	160	YSSANNCTF	0,5758
B58	HLA-B*58:09	258	WTAGAAAYY	0,5718
B58	HLA-B*58:09	304	KSFTVEKGIY	0,5095
B58	HLA-B*58:09	344	ATRFASVYAW	0,7902
B58	HLA-B*58:09	604	TSNQVAVLY	0,8181
B58	HLA-B*58:09	622	VAIHADQLTPTW	0,8017
B58	HLA-B*58:09	623	AIHADQLTPTW	0,5232
B58	HLA-B*58:09	624	IHADQLTPTW	0,8068
B58	HLA-B*58:09	625	HADQLTPTW	0,969
B58	HLA-B*58:09	687	VASQSIIAY	0,7266
B58	HLA-B*58:09	710	NSIAIPTNF	0,8466
B58	HLA-B*58:09	712	IAIPTNFTI	0,8112
B58	HLA-B*58:09	733	KTSVDCTMY	0,5183
B58	HLA-B*58:09	814	KRSFIEDLLF	0,5561
B58	HLA-B*58:09	815	RSFIEDLLF	0,8962
B58	HLA-B*58:09	878	LAGTITSGW	0,8826
B58	HLA-B*58:09	879	AGTITSGWTF	0,5228
B58	HLA-B*58:09	880	GTITSGWTF	0,86
B58	HLA-B*58:09	892	AALQIPFAM	0,5678
B58	HLA-B*58:09	898	FAMQMAYRF	0,7372
B58	HLA-B*58:09	1054	QSAPHGVVF	0,8091
B58	HLA-B*58:09	1093	GVFVSNGTHW	0,7325
B58	HLA-B*58:11	97	KSNIIRGW	0,6994
B58	HLA-B*58:11	160	YSSANNCTF	0,5597
B58	HLA-B*58:11	344	ATRFASVYAW	0,7778
B58	HLA-B*58:11	604	TSNQVAVLY	0,7554
B58	HLA-B*58:11	622	VAIHADQLTPTW	0,7607
B58	HLA-B*58:11	624	IHADQLTPTW	0,7759
B58	HLA-B*58:11	625	HADQLTPTW	0,9737
B58	HLA-B*58:11	687	VASQSIIAY	0,6802
B58	HLA-B*58:11	710	NSIAIPTNF	0,8133

B58	HLA-B*58:11	712	IAIPTNFTI	0,8079
B58	HLA-B*58:11	814	KRSFIEDLLF	0,5656
B58	HLA-B*58:11	815	RSFIEDLLF	0,9373
B58	HLA-B*58:11	878	LAGTITSGW	0,8666
B58	HLA-B*58:11	880	GTITSGWTF	0,879
B58	HLA-B*58:11	898	FAMQMAYRF	0,6403
B58	HLA-B*58:11	1054	QSAPHGVVF	0,8091
B58	HLA-B*58:11	1093	GVFVSNGTHW	0,7252
B62	HLA-B*15:01	35	GVYYPDKVF	0,677
B62	HLA-B*15:01	47	VLHSTQDLF	0,5635
B62	HLA-B*15:01	192	FVFKNIDGY	0,678
B62	HLA-B*15:01	212	LVRDLPQGF	0,7136
B62	HLA-B*15:01	240	TLLALHRSY	0,6805
B62	HLA-B*15:01	258	WTAGAAAYY	0,5072
B62	HLA-B*15:01	261	GAAAYYVGY	0,52
B62	HLA-B*15:01	366	SVLYNSASF	0,5995
B62	HLA-B*15:01	413	GQTGKIADY	0,764
B62	HLA-B*15:01	497	FQPTNGVGY	0,7039
B62	HLA-B*15:01	628	QLTPTWRVY	0,6565
B62	HLA-B*15:01	634	RVYSTGSNVF	0,8491
B62	HLA-B*15:01	686	SVASQSIIAY	0,5924
B62	HLA-B*15:01	687	VASQSIIAY	0,7822
B62	HLA-B*15:01	698	SLGAENSVAY	0,6839
B62	HLA-B*15:01	880	GTITSGWTF	0,6048
B62	HLA-B*15:01	894	LQIPFAMQM	0,6403
B62	HLA-B*15:01	919	NQKLIANQF	0,7035
B62	HLA-B*15:01	962	LVKQLSSNF	0,7084
B62	HLA-B*15:01	1000	RLQSLQTY	0,5767
B62	HLA-B*15:01	1054	QSAPHGVVF	0,7211
B62	HLA-B*15:01	1113	QIITTDNTF	0,6229
B62	HLA-B*15:01	1264	VLKGVKLHY	0,8811
B62	HLA-B*15:02	84	LPFNDGVYF	0,5012
B62	HLA-B*15:02	192	FVFKNIDGY	0,6067
B62	HLA-B*15:02	212	LVRDLPQGF	0,5599
B62	HLA-B*15:02	240	TLLALHRSY	0,6225
B62	HLA-B*15:02	366	SVLYNSASF	0,593
B62	HLA-B*15:02	628	QLTPTWRVY	0,6554
B62	HLA-B*15:02	687	VASQSIIAY	0,8247
B62	HLA-B*15:02	698	SLGAENSVAY	0,593
B62	HLA-B*15:02	699	LGAENSVAY	0,5304
B62	HLA-B*15:02	896	IPFAMQMAY	0,6693
B62	HLA-B*15:02	1054	QSAPHGVVF	0,678
B62	HLA-B*15:02	1113	QIITTDNTF	0,6078
B62	HLA-B*15:05	192	FVFKNIDGY	0,568
B62	HLA-B*15:05	240	TLLALHRSY	0,5065
B62	HLA-B*15:05	413	GQTGKIADY	0,5265
B62	HLA-B*15:05	497	FQPTNGVGY	0,5981
B62	HLA-B*15:05	634	RVYSTGSNVF	0,5053
B62	HLA-B*15:05	687	VASQSIIAY	0,7516
B62	HLA-B*15:05	689	SQSIIAYTM	0,5439

B62	HLA-B*15:05	880	GTITSGWTF	0,603
B62	HLA-B*15:05	894	LQIPFAMQM	0,5925
B62	HLA-B*15:05	1054	QSAPHGVVF	0,588
B62	HLA-B*15:05	1113	QIITTDNTF	0,5456
B62	HLA-B*15:12	628	QLTPTWRVY	0,5076
B62	HLA-B*15:12	634	RVYSTGSNVF	0,5866
B62	HLA-B*15:12	687	VASQSIIAY	0,588
B62	HLA-B*15:12	1054	QSAPHGVVF	0,5578
B62	HLA-B*15:14	634	RVYSTGSNVF	0,5315
B62	HLA-B*15:15	192	FVFKNIDGY	0,6152
B62	HLA-B*15:15	212	LVRDLPQGF	0,6015
B62	HLA-B*15:15	240	TLLALHRSY	0,5454
B62	HLA-B*15:15	366	SVLYNSASF	0,5295
B62	HLA-B*15:15	628	QLTPTWRVY	0,5711
B62	HLA-B*15:15	634	RVYSTGSNVF	0,5219
B62	HLA-B*15:15	686	SVASQSIIAY	0,5315
B62	HLA-B*15:15	687	VASQSIIAY	0,7424
B62	HLA-B*15:15	698	SLGAENSVAY	0,6339
B62	HLA-B*15:15	699	LGAENSVAY	0,5063
B62	HLA-B*15:15	919	NQKLIANQF	0,6734
B62	HLA-B*15:15	962	LVKQLSSNF	0,5773
B62	HLA-B*15:15	1054	QSAPHGVVF	0,6835
B62	HLA-B*15:15	1113	QIITTDNTF	0,6196
B62	HLA-B*15:19	628	QLTPTWRVY	0,5076
B62	HLA-B*15:19	634	RVYSTGSNVF	0,5866
B62	HLA-B*15:19	687	VASQSIIAY	0,588
B62	HLA-B*15:19	1054	QSAPHGVVF	0,5578
B62	HLA-B*15:20	192	FVFKNIDGY	0,6015
B62	HLA-B*15:20	240	TLLALHRSY	0,5374
B62	HLA-B*15:20	497	FQPTNGVGY	0,5717
B62	HLA-B*15:20	628	QLTPTWRVY	0,5008
B62	HLA-B*15:20	687	VASQSIIAY	0,764
B62	HLA-B*15:20	689	SQSIIAYTM	0,525
B62	HLA-B*15:20	698	SLGAENSVAY	0,5119
B62	HLA-B*15:20	880	GTITSGWTF	0,5777
B62	HLA-B*15:20	894	LQIPFAMQM	0,5729
B62	HLA-B*15:20	1054	QSAPHGVVF	0,5705
B62	HLA-B*15:20	1113	QIITTDNTF	0,55
B62	HLA-B*15:25	30	NSFTRGVYY	0,5102
B62	HLA-B*15:25	35	GVYYPDKVF	0,7166
B62	HLA-B*15:25	47	VLHSTQDLF	0,5497
B62	HLA-B*15:25	152	WMESEFRVY	0,5406
B62	HLA-B*15:25	192	FVFKNIDGY	0,6774
B62	HLA-B*15:25	212	LVRDLPQGF	0,6749
B62	HLA-B*15:25	240	TLLALHRSY	0,7448
B62	HLA-B*15:25	261	GAAAYYVGY	0,5614
B62	HLA-B*15:25	366	SVLYNSASF	0,6588
B62	HLA-B*15:25	413	GQTGKIADY	0,6454
B62	HLA-B*15:25	497	FQPTNGVGY	0,6173
B62	HLA-B*15:25	628	QLTPTWRVY	0,7063

B62	HLA-B*15:25	634	RVYSTGSNVF	0,8174
B62	HLA-B*15:25	686	SVASQSIIAY	0,5343
B62	HLA-B*15:25	687	VASQSIIAY	0,8425
B62	HLA-B*15:25	698	SLGAENSVAY	0,6499
B62	HLA-B*15:25	699	LGAENSVAY	0,5125
B62	HLA-B*15:25	880	GTITSGWTF	0,6109
B62	HLA-B*15:25	894	LQIPFAMQM	0,6635
B62	HLA-B*15:25	962	LVKQLSSNF	0,5897
B62	HLA-B*15:25	1000	RLQSLQTY	0,5751
B62	HLA-B*15:25	1054	QSAPHGVVF	0,6973
B62	HLA-B*15:25	1113	QIITTDNTF	0,6015
B62	HLA-B*15:25	1264	VLKGVKLHY	0,8551
B62	HLA-B*15:28	35	GVYYPDKVF	0,677
B62	HLA-B*15:28	47	VLHSTQDLF	0,5635
B62	HLA-B*15:28	192	FVFKNIDGY	0,678
B62	HLA-B*15:28	212	LVRDLPQGF	0,7136
B62	HLA-B*15:28	240	TLLALHRSY	0,6805
B62	HLA-B*15:28	258	WTAGAAAYY	0,5072
B62	HLA-B*15:28	261	GAAAYYVGY	0,52
B62	HLA-B*15:28	366	SVLYNSASF	0,5995
B62	HLA-B*15:28	413	GQTGKIADY	0,764
B62	HLA-B*15:28	497	FQPTNGVGY	0,7039
B62	HLA-B*15:28	628	QLTPTWRVY	0,6565
B62	HLA-B*15:28	634	RVYSTGSNVF	0,8491
B62	HLA-B*15:28	686	SVASQSIIAY	0,5924
B62	HLA-B*15:28	687	VASQSIIAY	0,7822
B62	HLA-B*15:28	698	SLGAENSVAY	0,6839
B62	HLA-B*15:28	880	GTITSGWTF	0,6048
B62	HLA-B*15:28	894	LQIPFAMQM	0,6403
B62	HLA-B*15:28	919	NQKLIANQF	0,7035
B62	HLA-B*15:28	962	LVKQLSSNF	0,7084
B62	HLA-B*15:28	1000	RLQSLQTY	0,5767
B62	HLA-B*15:28	1054	QSAPHGVVF	0,7211
B62	HLA-B*15:28	1113	QIITTDNTF	0,6229
B62	HLA-B*15:28	1264	VLKGVKLHY	0,8811
B62	HLA-B*15:31	84	LPFNDGVYF	0,5604
B62	HLA-B*15:31	192	FVFKNIDGY	0,5375
B62	HLA-B*15:31	687	VASQSIIAY	0,7264
B62	HLA-B*15:31	896	IPFAMQMAY	0,5929
B62	HLA-B*15:31	1054	QSAPHGVVF	0,5241
B62	HLA-B*15:31	1113	QIITTDNTF	0,5135
B62	HLA-B*15:33	35	GVYYPDKVF	0,677
B62	HLA-B*15:33	47	VLHSTQDLF	0,5635
B62	HLA-B*15:33	192	FVFKNIDGY	0,678
B62	HLA-B*15:33	212	LVRDLPQGF	0,7136
B62	HLA-B*15:33	240	TLLALHRSY	0,6805
B62	HLA-B*15:33	258	WTAGAAAYY	0,5072
B62	HLA-B*15:33	261	GAAAYYVGY	0,52
B62	HLA-B*15:33	366	SVLYNSASF	0,5995
B62	HLA-B*15:33	413	GQTGKIADY	0,764

B62	HLA-B*15:33	497	FQPTNGVGY	0,7039
B62	HLA-B*15:33	628	QLTPTWRVY	0,6565
B62	HLA-B*15:33	634	RVYSTGSNVF	0,8491
B62	HLA-B*15:33	686	SVASQSIIAY	0,5924
B62	HLA-B*15:33	687	VASQSIIAY	0,7822
B62	HLA-B*15:33	698	SLGAENSVAY	0,6839
B62	HLA-B*15:33	880	GTITSGWTF	0,6048
B62	HLA-B*15:33	894	LQIPFAMQM	0,6403
B62	HLA-B*15:33	919	NQKLIANQF	0,7035
B62	HLA-B*15:33	962	LVKQLSSNF	0,7084
B62	HLA-B*15:33	1000	RLQSLQTY	0,5767
B62	HLA-B*15:33	1054	QSAPHGVVF	0,7211
B62	HLA-B*15:33	1113	QIITTDNTF	0,6229
B62	HLA-B*15:33	1264	VLKGVKLHY	0,8811
B62	HLA-B*15:34	35	GVYYPDKVF	0,677
B62	HLA-B*15:34	47	VLHSTQDLF	0,5635
B62	HLA-B*15:34	192	FVFKNIDGY	0,678
B62	HLA-B*15:34	212	LVRDLPQGF	0,7136
B62	HLA-B*15:34	240	TLLALHRSY	0,6805
B62	HLA-B*15:34	258	WTAGAAAYY	0,5072
B62	HLA-B*15:34	261	GAAAYYVGY	0,52
B62	HLA-B*15:34	366	SVLYNSASF	0,5995
B62	HLA-B*15:34	413	GQTGKIADY	0,764
B62	HLA-B*15:34	497	FQPTNGVGY	0,7039
B62	HLA-B*15:34	628	QLTPTWRVY	0,6565
B62	HLA-B*15:34	634	RVYSTGSNVF	0,8491
B62	HLA-B*15:34	686	SVASQSIIAY	0,5924
B62	HLA-B*15:34	687	VASQSIIAY	0,7822
B62	HLA-B*15:34	698	SLGAENSVAY	0,6839
B62	HLA-B*15:34	880	GTITSGWTF	0,6048
B62	HLA-B*15:34	894	LQIPFAMQM	0,6403
B62	HLA-B*15:34	919	NQKLIANQF	0,7035
B62	HLA-B*15:34	962	LVKQLSSNF	0,7084
B62	HLA-B*15:34	1000	RLQSLQTY	0,5767
B62	HLA-B*15:34	1054	QSAPHGVVF	0,7211
B62	HLA-B*15:34	1113	QIITTDNTF	0,6229
B62	HLA-B*15:34	1264	VLKGVKLHY	0,8811
B62	HLA-B*15:38	30	NSFTRGVYY	0,5586
B62	HLA-B*15:38	152	WMESEFRVY	0,5815
B62	HLA-B*15:38	192	FVFKNIDGY	0,6613
B62	HLA-B*15:38	212	LVRDLPQGF	0,6736
B62	HLA-B*15:38	240	TLLALHRSY	0,7304
B62	HLA-B*15:38	261	GAAAYYVGY	0,5782
B62	HLA-B*15:38	366	SVLYNSASF	0,5604
B62	HLA-B*15:38	413	GQTGKIADY	0,7293
B62	HLA-B*15:38	464	FERDISTEIY	0,5752
B62	HLA-B*15:38	497	FQPTNGVGY	0,705
B62	HLA-B*15:38	628	QLTPTWRVY	0,676
B62	HLA-B*15:38	634	RVYSTGSNVF	0,7511
B62	HLA-B*15:38	686	SVASQSIIAY	0,5671

B62	HLA-B*15:38	687	VASQSIIAY	0,7799
B62	HLA-B*15:38	689	SQSIIAYTM	0,5356
B62	HLA-B*15:38	698	SLGAENSVAY	0,6347
B62	HLA-B*15:38	699	LGAENSVAY	0,5322
B62	HLA-B*15:38	880	GTITSGWTF	0,5811
B62	HLA-B*15:38	894	LQIPFAMQM	0,6945
B62	HLA-B*15:38	919	NQKLIANQF	0,8136
B62	HLA-B*15:38	962	LVKQLSSNF	0,7271
B62	HLA-B*15:38	1000	RLQSLQTY	0,5657
B62	HLA-B*15:38	1054	QSAPHGVVF	0,711
B62	HLA-B*15:38	1113	QIITTDNTF	0,5803
B62	HLA-B*15:38	1264	VLKGVKLHY	0,8714
B62	HLA-B*15:39	35	GVYYPDKVF	0,7375
B62	HLA-B*15:39	47	VLHSTQDLF	0,5828
B62	HLA-B*15:39	83	VLPFNDGVYF	0,5256
B62	HLA-B*15:39	152	WMESEFRVY	0,5416
B62	HLA-B*15:39	192	FVFKNIDGY	0,6491
B62	HLA-B*15:39	212	LVRDLPQGF	0,6773
B62	HLA-B*15:39	240	TLLALHRSY	0,7211
B62	HLA-B*15:39	261	GAAAYYVGY	0,5543
B62	HLA-B*15:39	366	SVLYNSASF	0,6531
B62	HLA-B*15:39	413	GQTGKIADY	0,6727
B62	HLA-B*15:39	497	FQPTNGVGY	0,6539
B62	HLA-B*15:39	628	QLTPTWRVY	0,6869
B62	HLA-B*15:39	634	RVYSTGSNVF	0,8327
B62	HLA-B*15:39	686	SVASQSIIAY	0,5034
B62	HLA-B*15:39	687	VASQSIIAY	0,8337
B62	HLA-B*15:39	689	SQSIIAYTM	0,5045
B62	HLA-B*15:39	698	SLGAENSVAY	0,6247
B62	HLA-B*15:39	699	LGAENSVAY	0,5064
B62	HLA-B*15:39	815	RSFIEDLLF	0,5266
B62	HLA-B*15:39	880	GTITSGWTF	0,6421
B62	HLA-B*15:39	894	LQIPFAMQM	0,6884
B62	HLA-B*15:39	919	NQKLIANQF	0,5508
B62	HLA-B*15:39	962	LVKQLSSNF	0,5971
B62	HLA-B*15:39	1000	RLQSLQTY	0,582
B62	HLA-B*15:39	1054	QSAPHGVVF	0,7223
B62	HLA-B*15:39	1113	QIITTDNTF	0,6116
B62	HLA-B*15:39	1264	VLKGVKLHY	0,8466
B62	HLA-B*15:40	634	RVYSTGSNVF	0,6651
B62	HLA-B*15:40	687	VASQSIIAY	0,5923
B62	HLA-B*15:40	894	LQIPFAMQM	0,564
B62	HLA-B*15:40	1264	VLKGVKLHY	0,65
B62	HLA-B*15:50	192	FVFKNIDGY	0,5522
B62	HLA-B*15:50	212	LVRDLPQGF	0,5998
B62	HLA-B*15:50	240	TLLALHRSY	0,595
B62	HLA-B*15:50	413	GQTGKIADY	0,6557
B62	HLA-B*15:50	628	QLTPTWRVY	0,567
B62	HLA-B*15:50	634	RVYSTGSNVF	0,7616
B62	HLA-B*15:50	687	VASQSIIAY	0,7011

B62	HLA-B*15:50	698	SLGAENSVAY	0,5791
B62	HLA-B*15:50	880	GTITSGWTF	0,5108
B62	HLA-B*15:50	894	LQIPFAMQM	0,5936
B62	HLA-B*15:50	919	NQKLIANQF	0,635
B62	HLA-B*15:50	962	LVKQLSSNF	0,5684
B62	HLA-B*15:50	1000	RLQSLQTY	0,504
B62	HLA-B*15:50	1054	QSAPHGVVF	0,6031
B62	HLA-B*15:50	1264	VLKGVKLHY	0,8458
B62	HLA-B*15:60	35	GVYYPDKVF	0,677
B62	HLA-B*15:60	47	VLHSTQDLF	0,5635
B62	HLA-B*15:60	192	FVFKNIDGY	0,678
B62	HLA-B*15:60	212	LVRDLPQGF	0,7136
B62	HLA-B*15:60	240	TLLALHRSY	0,6805
B62	HLA-B*15:60	258	WTAGAAAYY	0,5072
B62	HLA-B*15:60	261	GAAAYYVGY	0,52
B62	HLA-B*15:60	366	SVLYNSASF	0,5995
B62	HLA-B*15:60	413	GQTGKIADY	0,764
B62	HLA-B*15:60	497	FQPTNGVGY	0,7039
B62	HLA-B*15:60	628	QLTPTWRVY	0,6565
B62	HLA-B*15:60	634	RVYSTGSNVF	0,8491
B62	HLA-B*15:60	686	SVASQSIIAY	0,5924
B62	HLA-B*15:60	687	VASQSIIAY	0,7822
B62	HLA-B*15:60	698	SLGAENSVAY	0,6839
B62	HLA-B*15:60	880	GTITSGWTF	0,6048
B62	HLA-B*15:60	894	LQIPFAMQM	0,6403
B62	HLA-B*15:60	919	NQKLIANQF	0,7035
B62	HLA-B*15:60	962	LVKQLSSNF	0,7084
B62	HLA-B*15:60	1000	RLQSLQTY	0,5767
B62	HLA-B*15:60	1054	QSAPHGVVF	0,7211
B62	HLA-B*15:60	1113	QIITTDNTF	0,6229
B62	HLA-B*15:60	1264	VLKGVKLHY	0,8811
B62	HLA-B*15:65	35	GVYYPDKVF	0,5822
B62	HLA-B*15:65	47	VLHSTQDLF	0,6073
B62	HLA-B*15:65	152	WMESEFRVY	0,5864
B62	HLA-B*15:65	192	FVFKNIDGY	0,5038
B62	HLA-B*15:65	212	LVRDLPQGF	0,5359
B62	HLA-B*15:65	240	TLLALHRSY	0,6159
B62	HLA-B*15:65	366	SVLYNSASF	0,6187
B62	HLA-B*15:65	584	ILDITPCSF	0,5073
B62	HLA-B*15:65	628	QLTPTWRVY	0,6602
B62	HLA-B*15:65	634	RVYSTGSNVF	0,7599
B62	HLA-B*15:65	686	SVASQSIIAY	0,5527
B62	HLA-B*15:65	687	VASQSIIAY	0,792
B62	HLA-B*15:65	698	SLGAENSVAY	0,6847
B62	HLA-B*15:65	880	GTITSGWTF	0,5709
B62	HLA-B*15:65	1000	RLQSLQTY	0,5594
B62	HLA-B*15:65	1054	QSAPHGVVF	0,7159
B62	HLA-B*15:65	1113	QIITTDNTF	0,5676
B62	HLA-B*15:65	1264	VLKGVKLHY	0,7435
B62	HLA-B*15:70	192	FVFKNIDGY	0,5037

B62	HLA-B*15:70	212	LVRDLPQGF	0,5805
B62	HLA-B*15:70	413	GQTGKIADY	0,5593
B62	HLA-B*15:70	634	RVYSTGSNVF	0,6634
B62	HLA-B*15:70	687	VASQSIIAY	0,563
B62	HLA-B*15:70	962	LVKQLSSNF	0,5026
B62	HLA-B*15:70	1054	QSAPHGVVF	0,5214
B62	HLA-B*15:70	1264	VLKGVKLHY	0,7409
B62	HLA-B*15:75	35	GVYYPDKVF	0,6355
B62	HLA-B*15:75	47	VLHSTQDLF	0,524
B62	HLA-B*15:75	192	FVFKNIDGY	0,652
B62	HLA-B*15:75	212	LVRDLPQGF	0,6707
B62	HLA-B*15:75	240	TLLALHRSY	0,6492
B62	HLA-B*15:75	366	SVLYNSASF	0,5638
B62	HLA-B*15:75	413	GQTGKIADY	0,7207
B62	HLA-B*15:75	497	FQPTNGVGY	0,656
B62	HLA-B*15:75	628	QLTPTWRVY	0,6233
B62	HLA-B*15:75	634	RVYSTGSNVF	0,8275
B62	HLA-B*15:75	686	SVASQSIIAY	0,5578
B62	HLA-B*15:75	687	VASQSIIAY	0,7575
B62	HLA-B*15:75	698	SLGAENSVAY	0,6521
B62	HLA-B*15:75	880	GTITSGWTF	0,5821
B62	HLA-B*15:75	894	LQIPFAMQM	0,6094
B62	HLA-B*15:75	919	NQKLIANQF	0,6621
B62	HLA-B*15:75	962	LVKQLSSNF	0,6585
B62	HLA-B*15:75	1000	RLQSLQTY	0,533
B62	HLA-B*15:75	1054	QSAPHGVVF	0,6833
B62	HLA-B*15:75	1113	QIITTDNTF	0,5867
B62	HLA-B*15:75	1264	VLKGVKLHY	0,8622
B62	HLA-B*15:78	35	GVYYPDKVF	0,677
B62	HLA-B*15:78	47	VLHSTQDLF	0,5635
B62	HLA-B*15:78	192	FVFKNIDGY	0,678
B62	HLA-B*15:78	212	LVRDLPQGF	0,7136
B62	HLA-B*15:78	240	TLLALHRSY	0,6805
B62	HLA-B*15:78	258	WTAGAAAYY	0,5072
B62	HLA-B*15:78	261	GAAAYYVGY	0,52
B62	HLA-B*15:78	366	SVLYNSASF	0,5995
B62	HLA-B*15:78	413	GQTGKIADY	0,764
B62	HLA-B*15:78	497	FQPTNGVGY	0,7039
B62	HLA-B*15:78	628	QLTPTWRVY	0,6565
B62	HLA-B*15:78	634	RVYSTGSNVF	0,8491
B62	HLA-B*15:78	686	SVASQSIIAY	0,5924
B62	HLA-B*15:78	687	VASQSIIAY	0,7822
B62	HLA-B*15:78	698	SLGAENSVAY	0,6839
B62	HLA-B*15:78	880	GTITSGWTF	0,6048
B62	HLA-B*15:78	894	LQIPFAMQM	0,6403
B62	HLA-B*15:78	919	NQKLIANQF	0,7035
B62	HLA-B*15:78	962	LVKQLSSNF	0,7084
B62	HLA-B*15:78	1000	RLQSLQTY	0,5767
B62	HLA-B*15:78	1054	QSAPHGVVF	0,7211
B62	HLA-B*15:78	1113	QIITTDNTF	0,6229

B62	HLA-B*15:78	1264	VLKGVKLHY	0,8811
B62	HLA-B*15:81	35	GVYYPDKVF	0,677
B62	HLA-B*15:81	47	VLHSTQDLF	0,5635
B62	HLA-B*15:81	192	FVFKNIDGY	0,678
B62	HLA-B*15:81	212	LVRDLPQGF	0,7136
B62	HLA-B*15:81	240	TLLALHRSY	0,6805
B62	HLA-B*15:81	258	WTAGAAAYY	0,5072
B62	HLA-B*15:81	261	GAAAYYVGY	0,52
B62	HLA-B*15:81	366	SVLYNSASF	0,5995
B62	HLA-B*15:81	413	GQTGKIADY	0,764
B62	HLA-B*15:81	497	FQPTNGVGY	0,7039
B62	HLA-B*15:81	628	QLTPTWRVY	0,6565
B62	HLA-B*15:81	634	RVYSTGSNVF	0,8491
B62	HLA-B*15:81	686	SVASQSIIAY	0,5924
B62	HLA-B*15:81	687	VASQSIIAY	0,7822
B62	HLA-B*15:81	698	SLGAENSVAY	0,6839
B62	HLA-B*15:81	880	GTITSGWTF	0,6048
B62	HLA-B*15:81	894	LQIPFAMQM	0,6403
B62	HLA-B*15:81	919	NQKLIANQF	0,7035
B62	HLA-B*15:81	962	LVKQLSSNF	0,7084
B62	HLA-B*15:81	1000	RLQSLQTY	0,5767
B62	HLA-B*15:81	1054	QSAPHGVVF	0,7211
B62	HLA-B*15:81	1113	QIITTDNTF	0,6229
B62	HLA-B*15:81	1264	VLKGVKLHY	0,8811
B62	HLA-B*15:82	35	GVYYPDKVF	0,677
B62	HLA-B*15:82	47	VLHSTQDLF	0,5635
B62	HLA-B*15:82	192	FVFKNIDGY	0,678
B62	HLA-B*15:82	212	LVRDLPQGF	0,7136
B62	HLA-B*15:82	240	TLLALHRSY	0,6805
B62	HLA-B*15:82	258	WTAGAAAYY	0,5072
B62	HLA-B*15:82	261	GAAAYYVGY	0,52
B62	HLA-B*15:82	366	SVLYNSASF	0,5995
B62	HLA-B*15:82	413	GQTGKIADY	0,764
B62	HLA-B*15:82	497	FQPTNGVGY	0,7039
B62	HLA-B*15:82	628	QLTPTWRVY	0,6565
B62	HLA-B*15:82	634	RVYSTGSNVF	0,8491
B62	HLA-B*15:82	686	SVASQSIIAY	0,5924
B62	HLA-B*15:82	687	VASQSIIAY	0,7822
B62	HLA-B*15:82	698	SLGAENSVAY	0,6839
B62	HLA-B*15:82	880	GTITSGWTF	0,6048
B62	HLA-B*15:82	894	LQIPFAMQM	0,6403
B62	HLA-B*15:82	919	NQKLIANQF	0,7035
B62	HLA-B*15:82	962	LVKQLSSNF	0,7084
B62	HLA-B*15:82	1000	RLQSLQTY	0,5767
B62	HLA-B*15:82	1054	QSAPHGVVF	0,7211
B62	HLA-B*15:82	1113	QIITTDNTF	0,6229
B62	HLA-B*15:82	1264	VLKGVKLHY	0,8811
B62	HLA-B*15:85	35	GVYYPDKVF	0,6525
B62	HLA-B*15:85	47	VLHSTQDLF	0,5234
B62	HLA-B*15:85	192	FVFKNIDGY	0,6835

B62	HLA-B*15:85	212	LVRDLPQGF	0,6903
B62	HLA-B*15:85	240	TLLALHRSY	0,6924
B62	HLA-B*15:85	258	WTAGAAAYY	0,5057
B62	HLA-B*15:85	261	GAAAYYVGY	0,5182
B62	HLA-B*15:85	366	SVLYNSASF	0,5905
B62	HLA-B*15:85	413	GQTGKIADY	0,7286
B62	HLA-B*15:85	497	FQPTNGVGY	0,6701
B62	HLA-B*15:85	628	QLTPTWRVY	0,6639
B62	HLA-B*15:85	634	RVYSTGSNVF	0,8172
B62	HLA-B*15:85	686	SVASQSIIAY	0,6057
B62	HLA-B*15:85	687	VASQSIIAY	0,7768
B62	HLA-B*15:85	698	SLGAENSVAY	0,6849
B62	HLA-B*15:85	880	GTITSGWTF	0,5734
B62	HLA-B*15:85	894	LQIPFAMQM	0,5976
B62	HLA-B*15:85	919	NQKLIANQF	0,6783
B62	HLA-B*15:85	962	LVKQLSSNF	0,6864
B62	HLA-B*15:85	1000	RLQSLQTY	0,5531
B62	HLA-B*15:85	1054	QSAPHGVVF	0,6958
B62	HLA-B*15:85	1113	QIITTDNTF	0,608
B62	HLA-B*15:85	1264	VLKGVKLHY	0,8756
B62	HLA-B*15:88	84	LPFNDGVYF	0,5189
B62	HLA-B*15:88	192	FVFKNIDGY	0,5876
B62	HLA-B*15:88	240	TLLALHRSY	0,5087
B62	HLA-B*15:88	628	QLTPTWRVY	0,53
B62	HLA-B*15:88	687	VASQSIIAY	0,7704
B62	HLA-B*15:88	698	SLGAENSVAY	0,5239
B62	HLA-B*15:88	699	LGAENSVAY	0,5042
B62	HLA-B*15:88	896	IPFAMQMAY	0,6381
B62	HLA-B*15:88	1054	QSAPHGVVF	0,5587
B62	HLA-B*15:88	1113	QIITTDNTF	0,5554
B62	HLA-B*15:92	35	GVYYPDKVF	0,677
B62	HLA-B*15:92	47	VLHSTQDLF	0,5635
B62	HLA-B*15:92	192	FVFKNIDGY	0,678
B62	HLA-B*15:92	212	LVRDLPQGF	0,7136
B62	HLA-B*15:92	240	TLLALHRSY	0,6805
B62	HLA-B*15:92	258	WTAGAAAYY	0,5072
B62	HLA-B*15:92	261	GAAAYYVGY	0,52
B62	HLA-B*15:92	366	SVLYNSASF	0,5995
B62	HLA-B*15:92	413	GQTGKIADY	0,764
B62	HLA-B*15:92	497	FQPTNGVGY	0,7039
B62	HLA-B*15:92	628	QLTPTWRVY	0,6565
B62	HLA-B*15:92	634	RVYSTGSNVF	0,8491
B62	HLA-B*15:92	686	SVASQSIIAY	0,5924
B62	HLA-B*15:92	687	VASQSIIAY	0,7822
B62	HLA-B*15:92	698	SLGAENSVAY	0,6839
B62	HLA-B*15:92	880	GTITSGWTF	0,6048
B62	HLA-B*15:92	894	LQIPFAMQM	0,6403
B62	HLA-B*15:92	919	NQKLIANQF	0,7035
B62	HLA-B*15:92	962	LVKQLSSNF	0,7084
B62	HLA-B*15:92	1000	RLQSLQTY	0,5767

B62	HLA-B*15:92	1054	QSAPHGVVF	0,7211
B62	HLA-B*15:92	1113	QIITTDNTF	0,6229
B62	HLA-B*15:92	1264	VLKGVKLHY	0,8811
B62	HLA-B*15:96	35	GVYYPDKVF	0,677
B62	HLA-B*15:96	47	VLHSTQDLF	0,5635
B62	HLA-B*15:96	192	FVFKNIDGY	0,678
B62	HLA-B*15:96	212	LVRDLPQGF	0,7136
B62	HLA-B*15:96	240	TLLALHRSY	0,6805
B62	HLA-B*15:96	258	WTAGAAAYY	0,5072
B62	HLA-B*15:96	261	GAAAYYVGY	0,52
B62	HLA-B*15:96	366	SVLYNSASF	0,5995
B62	HLA-B*15:96	413	GQTGKIADY	0,764
B62	HLA-B*15:96	497	FQPTNGVGY	0,7039
B62	HLA-B*15:96	628	QLTPTWRVY	0,6565
B62	HLA-B*15:96	634	RVYSTGSNVF	0,8491
B62	HLA-B*15:96	686	SVASQSIIAY	0,5924
B62	HLA-B*15:96	687	VASQSIIAY	0,7822
B62	HLA-B*15:96	698	SLGAENSVAY	0,6839
B62	HLA-B*15:96	880	GTITSGWTF	0,6048
B62	HLA-B*15:96	894	LQIPFAMQM	0,6403
B62	HLA-B*15:96	919	NQKLIANQF	0,7035
B62	HLA-B*15:96	962	LVKQLSSNF	0,7084
B62	HLA-B*15:96	1000	RLQSLQTY	0,5767
B62	HLA-B*15:96	1054	QSAPHGVVF	0,7211
B62	HLA-B*15:96	1113	QIITTDNTF	0,6229
B62	HLA-B*15:96	1264	VLKGVKLHY	0,8811
B62	HLA-B*15:97	35	GVYYPDKVF	0,677
B62	HLA-B*15:97	47	VLHSTQDLF	0,5635
B62	HLA-B*15:97	192	FVFKNIDGY	0,678
B62	HLA-B*15:97	212	LVRDLPQGF	0,7136
B62	HLA-B*15:97	240	TLLALHRSY	0,6805
B62	HLA-B*15:97	258	WTAGAAAYY	0,5072
B62	HLA-B*15:97	261	GAAAYYVGY	0,52
B62	HLA-B*15:97	366	SVLYNSASF	0,5995
B62	HLA-B*15:97	413	GQTGKIADY	0,764
B62	HLA-B*15:97	497	FQPTNGVGY	0,7039
B62	HLA-B*15:97	628	QLTPTWRVY	0,6565
B62	HLA-B*15:97	634	RVYSTGSNVF	0,8491
B62	HLA-B*15:97	686	SVASQSIIAY	0,5924
B62	HLA-B*15:97	687	VASQSIIAY	0,7822
B62	HLA-B*15:97	698	SLGAENSVAY	0,6839
B62	HLA-B*15:97	880	GTITSGWTF	0,6048
B62	HLA-B*15:97	894	LQIPFAMQM	0,6403
B62	HLA-B*15:97	919	NQKLIANQF	0,7035
B62	HLA-B*15:97	962	LVKQLSSNF	0,7084
B62	HLA-B*15:97	1000	RLQSLQTY	0,5767
B62	HLA-B*15:97	1054	QSAPHGVVF	0,7211
B62	HLA-B*15:97	1113	QIITTDNTF	0,6229
B62	HLA-B*15:97	1264	VLKGVKLHY	0,8811
B62	HLA-B*35:28	192	FVFKNIDGY	0,6199

B62	HLA-B*35:28	240	TLLALHRSY	0,5051
B62	HLA-B*35:28	261	GAAAYYVGY	0,5209
B62	HLA-B*35:28	339	GEVFNATRF	0,6317
B62	HLA-B*35:28	413	GQTGKIADY	0,5514
B62	HLA-B*35:28	443	SKVGGNYNY	0,6002
B62	HLA-B*35:28	464	FERDISTEIY	0,5471
B62	HLA-B*35:28	497	FQPTNGVGY	0,6463
B62	HLA-B*35:28	687	VASQSIIAY	0,7882
B62	HLA-B*35:28	689	SQSIIAYTM	0,6456
B62	HLA-B*35:28	699	LGAENSVAY	0,546
B62	HLA-B*35:28	815	RSFIEDLLF	0,5236
B62	HLA-B*35:28	880	GTITSGWTF	0,5709
B62	HLA-B*35:28	894	LQIPFAMQM	0,7173
B62	HLA-B*35:28	919	NQKLIANQF	0,5854
B62	HLA-B*35:28	1054	QSAPHGVVF	0,6134
B62	HLA-B*35:28	1113	QIITTDNTF	0,5096
B62	HLA-B*52:01	712	IAIPTNFTI	0,7144
B62	HLA-B*52:02	712	IAIPTNFTI	0,7268
B62	HLA-B*52:03	712	IAIPTNFTI	0,7225
B62	HLA-B*52:04	712	IAIPTNFTI	0,7144
B62	HLA-B*52:05	712	IAIPTNFTI	0,7144
B62	HLA-B*52:07	712	IAIPTNFTI	0,7144

Note: The epitopes mutated in Omicron variant versus Wuhan-1 are indicated in red.
