# Supplementary material for: Predicted HLA Class I and Class II Epitopes From Licensed Vaccines Are Largely Conserved in New SARS-CoV-2 Omicron Variant of Concern
Source: Front Immunol. 2022 Jan 28;13:832889. doi: 10.3389/fimmu.2022.832889 (PMC8831693; doi:10.3389/fimmu.2022.832889)
Supplement: Supplementary file 3 [file DataSheet_3.pdf]

epitope ID	Sequence	Begin position
1312101	AARDLICAQKFNGLT	845
1312102	AATKMSECVLGQSKR	1025
1314170	ADAGFIKQY	829
1068878	ADAGFIKQYGDCLGD	829
1310252	ADQLTPTWRVYSTGS	626
1068879	ADSFVIRGDEVRQIA	397
1314194	AEHVNNSY	653
1312107	AEHVNNSYECDIPIG	653
1312108	AEIRASANL	1016
1074838	AEIRASANLAATK	1016
1310253	AEIRASANLAATKMS	1016
1393673	AEIRASANLAATKMSEC	1016
1598225	AEIRASANLAATKMSECV	1016
1310254	AENSVAYSNNSIAIP	701
1393674	AENSVAYSNNSIAIPTN	701
1598271	AENSVAYSNNSIAIPTNF	701
1314206	AEVQIDRL	989
1220	AEVQIDRLI	989
530739	AEVQIDRLITGRLQS	989
1393677	AGAAAYYVGYLQPRTFL	260
1393678	AGAALQIPFAMQMAYRF	890
1598366	AGAALQIPFAMQMAYRFN	890
1074840	ISYECDIPIGAGICASYQTQT	647
1310259	AGFIKQYGDCLGDIA	831
1312129	AIPTNFTISVTTEIL	713
1310266	AIVMVTIMLCCMTSC	1226
1393684	AIVMVTIMLCCMTSCCS	1226
1598634	AIVMVTIMLCCMTSCCSC	1226
1314425	ALDPLSETK	292
1310271	ALLAGTITSGWTFGA	876
1393688	ALLAGTITSGWTFGAGA	876
1598808	ALLAGTITSGWTFGAGAA	876
2801	ALNTLVKQL	958
1312167	ALQIPFAMQMAYRFN	893
1410559	ALQIPFAMQMAYRFNGIGV	893
1310274	ALTGIAVEQDKNTQE	766
1397094	ANQFNSAIGKIQDSL	924
1310279	APGQTGKIADYNYKL	411
1330420	APHGVVFL	1056
3589	APHGVVFLHV	1056
1074847	APHGVVFLHVTYV	1056
1310281	APHGVVFLHVTYVPA	1056
3939	AQALNTLVK	956
1074854	AQALNTLVKQL	956
1310282	AQALNTLVKQLSSNF	956
1069137	AQYTSALLAGTITSG	871
1310284	ARDLICAQKFNGLTV	846
1074855	YTMSLGAENSVAYSNNSIAII	684
4321	ASANLAATK	1020
1393694	ASFSTFKCYGVSPTKLN	372
1599400	ASFSTFKCYGVSPTKLND	372

1312226	ASTEKSNIIRGWIFG	93
1544519	ASVYAWNRK	348
1329156	ASVYAWNRKRISN	348
1310294	ATKMSECVLGQSKRV	1026
1646541	ATRFASVYA	344
1329170	ATRFASVYAWNRKRISN	344
1599614	ATRFASVYAWNRKRISNC	344
1646687	AVDCALDPL	288
1393699	AVDCALDPLSETKCTLK	288
1599664	AVDCALDPLSETKCTLKS	288
1310295	AVEQDKNTQEVFAQV	771
1393700	AVEQDKNTQEVFAQVKQ	771
1599678	AVEQDKNTQEVFAQVKQI	771
1312238	AVLYQDVNCTEVPVA	609
1393701	AVRDPQTLEILDITPCS	575
1599765	AVRDPQTLEILDITPCSF	575
1393702	AWNSNNLDSKVGGNYNY	435
1599836	AWNSNNLDSKVGGNYNYL	435
1647179	AYRFNGIGV	903
1315180	AYSNNSIAI	706
1310300	AYSNNSIAIPTNFTI	706
1074865	AYSNNSIAIPTNFTISV	706
1393705	AYTMSLGAENSVAYSNN	694
1599861	AYTMSLGAENSVAYSNNS	694
1074866	CALDPLSETK	291
1310302	CALDPLSETKCTLKS	291
1310303	CAQKFNGLTVLPPLL	851
1310304	CASYQTQTNSPRRAR	671
1312257	CCKFDEDDSEPVLKG	1253
1393707	CCSCGSCCKFDEDDSEP	1247
1599871	CCSCGSCCKFDEDDSEPV	1247
1393708	CCSCLKGCCSCGSCCKF	1240
1599872	CCSCLKGCCSCGSCCKFD	1240
1310305	CDVVIGIVNNTVYDP	1126
1310306	CEFQFCNDPFLGVYY	131
1647797	CFTNVYADSF	391
1069287	CFTNVYADSFVIRGD	391
1393709	CGDSTECSNLLLQYGSF	743
1599891	CGDSTECSNLLLQYGSFC	743
1312260	CGPKKSTNLVKNKCV	525
1393711	CKFDEDDSEPVLKGVKL	1254
1599907	CKFDEDDSEPVLKGVKLH	1254
1312262	CLIGAEHVNNSYECD	649
6668	CMTSCCSCLK	1236
1310309	CMTSCCSCLKGCCSC	1236
1331139	CNDPFLGVY	136
1310311	CNDPFLGVYYHKNNK	136
1310312	CPFGEVFNATRFASV	336
1539742	[illegible]PFGEVFNATRFASVYAWN[illegible]	336
1310316	CSCLKGCCSCGSCCK	1241
1312272	CSNLLLQYGSFCTQL	749
1312273	CTEVPVAIHADQLTP	617

1393715	CTEVPVAIHADQLTPTW	617
1087469	CTEVPVAIHADQLTPTWR	617
1074869	CTFEYVSQPFLM	166
1309110	CTFEYVSQPFLMDLE	166
1649022	CTLKSFTVEK	301
1069290	CTLKSFTVEKGIYQT	301
7247	CVADYSVLY	361
1069291	CVADYSVLYNSASFS	361
1393716	CVNLTTRTQLPPAYTNS	15
1599956	CVNLTTRTQLPPAYTNSF	15
1649404	CYFPLQSYGF	488
1393717	CYGVSPTKLNDLCFTNV	379
1599960	CYGVSPTKLNDLCFTNVY	379
1315271	DAVRDPQTL	574
1312282	DCTMYICGDSTECSN	737
1312283	DEDDSEPVLKGVKLH	1257
1312289	DEVRQIAPGQTGKIA	405
1310326	DFCGKGYHLMSFPQS	1041
1310327	DFGGFNFSQILPDPS	796
1329262	DFTGCVIAWNSNNLDSK	428
1600380	DFTGCVIAWNSNNLDSKV	428
1315357	DGVYFASTEK	88
1330432	DGYFKIYSKHTPINL	198
1393720	DIADTTDAVRDPQTLEI	568
1600534	DIADTTDAVRDPQTLEIL	568
1310330	DITPCSFGGVSVITP	586
1312310	DKVEAEVQIDRLITG	985
9006	DKYFKNHTSPDVDLG	1153
1312311	DLCFTNVYADSFVIR	389
1312313	DLFLPFFSNVTWFHA	53
1312314	DLGDISGINASVVNI	1165
1074871	/VNIQKEIDRLNEVAKNLNES	1165
1393724	DLICAQKFNGLTVLPPL	848
1600798	DLICAQKFNGLTVLPPLL	848
1393725	DLLFNKVTLADAGFIKQ	820
1600818	DLLFNKVTLADAGFIKQY	820
1074872	DLPIGINITRFQTL	228
1393733	DRLNEVAKNLNESLIDL	1184
1601214	DRLNEVAKNLNESLIDLQ	1184
10112	DSFKEELDKY	1146
1310335	DSFKEELDKYFKNHT	1146
1310336	DSKTQSLLIVNNATN	111
1315542	DSKVGGNYNY	442
1393735	DSKVGGNYNYLYRLFRK	442
1601336	DSKVGGNYNYLYRLFRKS	442
1310337	DSLSSTASALGKLQD	936
1312333	DSSSGWTAGAAAYYV	253
1393736	DSSSGWTAGAAAYYVGY	253
1601381	DSSSGWTAGAAAYYVGYL	253
1069347	DSTECSNLLLQYGSF	745
1069350	DTTDAVRDPQTLEIL	571
1393740	DVDLGDISGINASVVNI	1163

1601527	DVDLGDISGINASVVNIQ	1163
11038	EAEVQIDRLITGRLQSL	988
1601746	EAEVQIDRLITGRLQSLQ	988
1069378	ECDIPIGAGICASYQ	661
1310345	ECVLGQSKRVDFCGK	1031
1310352	EFRVYSSANNCTFEY	156
1309113	EFVFKNIDGYFKIYS	191
1393743	EGFNCYFPLQSYGFQPT	484
1602300	EGFNCYFPLQSYGFQPTN	484
1074876	EILDITPCSF	583
1315818	EILPVSMTK	725
1312357	EILPVSMTKTSVDCT	725
1312358	EIRASANLAATKMSE	1017
1310360	EIYQAGSTPCNGVEG	471
1312359	EKGIYQTSNFRVQPT	309
1393747	EKGIYQTSNFRVQPTES	309
1602600	EKGIYQTSNFRVQPTESI	309
1393749	EKNFTTAPAICHDGKAH	1072
1602664	EKNFTTAPAICHDGKAHF	1072
1310361	EKSNIIRGWIFGTTL	96
1310362	ELDKYFKNHTSPDVD	1151
1074877	PWYIWLGFIAGLIAIVMVTIM	1202
1331642	ELLHAPATV	516
1310365	ELLHAPATVCGPKKS	516
1540107	ELLHAPATVCGPKKSTNLVK	516
1310367	ENGTITDAVDCALDP	281
1393752	ENGTITDAVDCALDPLS	281
1087498	ENGTITDAVDCALDPLSE	281
1329348	ENQKLIANQFNSAIGKI	918
1329349	ENQKLIANQFNSAIGKIQ	918
1315940	EPLVDLPI	224
1069445	EPQIITTDNTFVSGN	1111
1310371	EPVLKGVKL	1262
1312376	EQDKNTQEVFAQVKQ	773
1312380	ERDISTEIYQAGSTP	465
1397111	ESEFRVYSSANNCTFEYV	154
1329361	ESNKKFLPFQQFGRDIA	554
1329362	ESNKKFLPFQQFGRDIAD	554
1316068	ETKCTLKSF	298
1316107	EVFAQVKQI	780
1316108	EVFAQVKQIY	780
1316109	EVFNATRFASVY	340
1310377	EVRQIAPGQTGKIAD	406
1540176	EVRQIAPGQTGKIADYNYKL	406
1316287	EYVSQPFLM	169
1312390	EYVSQPFLMDLEGKQ	169
1393760	EYVSQPFLMDLEGKQGN	169
1087507	EYVSQPFLMDLEGKQGNF	169
1316310	FAMQMAYRF	898
1546300	FAQVKQIYK	782
1393761	FASTEKSNIIRGWIFGT	92
1603889	FASTEKSNIIRGWIFGTT	92

1074883	;WIFGTTLDSKTQSLLIVNNA	92
1316312	FASVYAWNR	347
1397116	FASVYAWNRKRISNCVAD	347
1074884	FCNDPFLGVYY	135
1316323	FDEDDSEPVL	1256
1310384	FDEDDSEPVLKGVKL	1256
1316333	FDNPVLPFNDGVYF	79
1331943	FELLHAPATV	515
1316352	FERDISTEI	464
1316353	FERDISTEIY	464
1316355	FEYVSQPFLM	168
1074885	FGEVFNATRFASVY	338
1392147	FGEVFNATRFASVYA	338
1312421	FGGFNFSQILPDPSK	797
1432856	FGGFNFSQILPDPSKPSKR	797
1397120	FGGFNFSQILPDPSKPSKRS	797
1069519	FGRDIADTTDAVRDP	565
1310392	FGTTLDSKTQSLLIV	106
1393764	FGTTLDSKTQSLLIVNN	106
1604068	FGTTLDSKTQSLLIVNNA	106
1312426	FHAIHVSGTNGTKRF	65
16156	FIAGLIAIV	1220
1546420	FIEDLLFNK	817
1069521	FIEDLLFNKVTLADA	817
1312434	FIKQYGDCLGDIAAR	833
1312438	FKCYGVSPTKLNDLC	377
1657458	FKIYSKHTPI	201
1310401	FKIYSKHTPINLVRD	201
1310402	FKNHTSPDVDLGDIS	1156
1393767	FKNHTSPDVDLGDISGI	1156
1604142	FKNHTSPDVDLGDISGIN	1156
1331958	FKNLREFVF	186
1310403	FKNLREFVFKNIDGY	186
1397124	FLGVYYHKNNKSWMESE	140
1597686	FLHVTYVPA	1062
1329407	FLPFFSNV	55
1316697	FLPFFSNVTW	55
1074888	FLPFFSNVTWFHAI	55
1310412	FNCYFPLQSYGFQPT	486
1540297	FNCYFPLQSYGFQPTNGVG'	486
1310413	FNDGVYFASTEKSNI	86
1069550	FNFNGLTGTGVLTES	541
1310414	FNGIGVTQNVLYENQ	906
1074890	YENQKLIANQFNSAIGKIQDS	906
1310415	FNGLTVLPPLLTDEM	855
1393768	FNGLTVLPPLLTDEMIA	855
1604218	FNGLTVLPPLLTDEMIAQ	855
531734	FPNITNLCPFGEVFN	329
1316853	FPQSAPHGV	1052
1074891	FPQSAPHGVVF	1052
1316854	FPREGVFV	1089
1312481	FPREGVFVSNGTHWF	1089

1392157	FQFCNDPFL	133
1312484	FQFCNDPFLGVYYHK	133
1435610	FQFCNDPFLGVYYHKNNK	133
1087346	FQPTNGVGY	497
1312486	FQPTNGVGYQPYRVV	497
1310418	FRKSNLKPFERDIST	456
1393770	FRKSNLKPFERDISTEI	456
1604361	FRKSNLKPFERDISTEIY	456
1540323	FRKSNLKPFERDISTEIYQA	456
1331988	FRSSVLHST	43
1393772	FRSSVLHSTQDLFLPFF	43
1604374	FRSSVLHSTQDLFLPFFS	43
1312488	FRVYSSANNCTFEYV	157
1312501	FTGCVIAWNSNNLDS	429
1436695	FTGCVIAWNSNNLDSKVGG	429
1317060	FTISVTTEI	718
1074898	FTISVTTEIL	718
1310423	FTVEKGIYQTSNFRV	306
1074900	FVFKNIDGY	192
1332003	FVFLVLLPL	2
1659240	FVFLVLLPLV	2
1317124	FVIRGDEVR	400
1393775	FVIRGDEVRQIAPGQTG	400
1604500	FVIRGDEVRQIAPGQTGK	400
1310426	FVSGNCDVVIGIVNN	1121
1393777	FVSGNCDVVIGIVNNTV	1121
1087522	FVSGNCDVVIGIVNNTVY	1121
1317137	FVSNGTHWF	1095
1659456	FVSNGTHWFV	1095
1312522	FYEPQIITTDNTFVS	1109
1437846	FYEPQIITTDNTFVSGNCD	1109
1317282	GAAAYYVGY	261
1310431	GAAAYYVGYLQPRTF	261
18514	GAALQIPFAMQMAYR	891
1393782	GAEHVNNSYECDIPIGA	652
1604573	GAEHVNNSYECDIPIGAG	652
531783	GAGAALQIPFAMQMA	889
1310434	GAISSVLNDILSRLD	971
1310436	GCCSCGSCCKFDEDD	1246
1310437	GCVIAWNSNNLDSKV	431
1317333	GEVFNATRF	339
1393786	GFIAGLIAIVMVTIMLC	1219
1605078	GFIAGLIAIVMVTIMLCC	1219
1074907	DIAARDLICAQKFNGLTVLPI	832
1312542	GFNCYFPLQSYGFQP	485
1330463	GFNFSQILPDPSKPSKR	799
1605081	GFNFSQILPDPSKPSKRS	799
1310441	GFQPTNGVGYQPYRV	496
1540449	3FQPTNGVGYQPYRVVVLSI	496
1309117	GGNYNYLYRLFRKSN	446
1540464	3GNYNYLYRLFRKSNLKPFE	446
1397134	3NYNYLYRLFRKSNLKPFER	446

1312547	GGVSVITPGTNTSNQ	593
1312549	GIAVEQDKNTQEVFA	769
1312550	GICASYQTQTNSPRR	669
1069816	GINASVVNIQKEIDR	1171
1354273	GINITRFQTLLALHRSY	232
1605379	GINITRFQTLLALHRSYL	232
1310444	GIVNNTVYDPLQPEL	1131
1547148	GIYQTSNFR	311
1310445	GIYQTSNFRVQPTES	311
1312555	GKAHFPREGVFVSNG	1085
1397135	GKGYHLMSFPQSAPH	1044
1393800	GKGYHLMSFPQSAPHGV	1044
1605487	GKGYHLMSFPQSAPHGVV	1044
1310447	GKIADYNYKLPDDFT	416
1540496	GKIADYNYKLPDDFTGCVIA	416
1393801	GKIQDSLSSTASALGKL	932
1605510	GKIQDSLSSTASALGKLQ	932
1310448	GKLQDVVNQNAQALN	946
1393802	GKLQDVVNQNAQALNTL	946
1605536	GKLQDVVNQNAQALNTLV	946
1069822	GKQGNFKNLREFVFK	181
20907	GLIAIVMVTI	1223
1312569	GLTGTGVLTESNKKF	545
1125063	GLTVLPPLL	857
1312570	GLTVLPPLLTDEMIA	857
1329486	GNYNYLYRLF	447
1087354	GPKKSTNLV	526
1309118	GPKKSTNLVKNKCVN	526
1393805	GPKKSTNLVKNKCVNFN	526
1605951	GPKKSTNLVKNKCVNFNF	526
1540546	GPKKSTNLVKNKCVNFNFN[illegible]	526
1312600	GQTGKIADYNYKLPD	413
1310457	GRDIADTTDAVRDPQ	566
1310459	GSCCKFDEDDSEPVL	1251
22322	GSFCTQLNR	757
1070003	GSFCTQLNRALTGIA	757
1393808	GSFCTQLNRALTGIAVE	757
1606354	GSFCTQLNRALTGIAVEQ	757
1310461	GSTPCNGVEGFNCYF	476
1540593	GSTPCNGVEGFNCYFPLQS[illegible]	476
1074915	GTHWFVTQR	1099
1074916	GTITSGWTF	880
1663099	GTKRFDNPV	75
1070035	GTNTSNQVAVLYQDV	601
1317875	GVFVSNGTHW	1093
1312621	GVFVSNGTHWFVTQR	1093
1393817	GVFVSNGTHWFVTQRNF	1093
1606672	GVFVSNGTHWFVTQRNFY	1093
1547617	GVLTESNKK	550
1309120	GVSPTKLNDLCFTNV	381
23200	GVVFLHVTY	1059
1312627	GVYFASTEK	89

1312628	GVYFASTEKSNIIRG	89
1597047	GVYHKNNK	140
1312630	GVYYHKNNK	142
1317887	GVYYPDKVFR	35
1312633	GWTAGAAAYYVGYLQ	257
23293	GWTFGAGAALQIPFA	885
1310470	GYHLMSFPQSAPHGV	1046
1547648	GYLQPRTFL	268
1317916	GYLQPRTFLL	268
1547652	GYQPYRVVV	504
1074918	GYQPYRVVVLSF	504
1070176	HADQLTPTWRVYSTG	625
1310473	HAIHVSGTNGTKRFD	66
1393824	HAPATVCGPKKSTNLVK	519
1606882	HAPATVCGPKKSTNLVKN	519
1393825	HGVVFLHVTYVPAQEKN	1058
1607025	HGVVFLHVTYVPAQEKNF	1058
1310474	HKNNKSWMESEFRVY	146
1318059	HLMSFPQSA	1048
1312663	HRSYLTPGDSSSGWT	245
1312672	HSTQDLFLPFFSNVT	49
1318206	HVSGTNGTK	69
1312678	HVSGTNGTKRFDNPV	69
1318209	HVTYVPAQEK	1064
1074925	HVTYVPAQEKNF	1064
1318219	HWFVTQRNF	1101
1310476	HWFVTQRNFYEPQII	1101
1448898	HWFVTQRNFYEPQIITTDN	1101
1312683	IADTTDAVRDPQTLE	569
1310479	IAGLIAIVMVTIMLC	1221
1318299	IAIPTNFTI	712
1070307	IAIVMVTIMLCCMTS	1225
1318302	IANQFNSAI	923
1312688	IAYTMSLGAENSVAY	693
1070308	ICHDGKAHFPREGVF	1081
1312689	IDGYFKIYSKHTPIN	197
1393831	IDGYFKIYSKHTPINLV	197
1607628	IDGYFKIYSKHTPINLVR	197
1393832	IDLQELGKYEQYIKWPW	1198
1607657	IDLQELGKYEQYIKWPWY	1198
25662	IDRLITGRLQSLQTY	993
1312693	IFGTTLDSKTQSLLI	105
1074926	IGAEHVNNSY	651
1310484	IGAEHVNNSYECDIP	651
1310485	IGAGICASYQTQTNS	666
1393837	IGAGICASYQTQTNSPR	666
1607869	IGAGICASYQTQTNSPRR	666
1310487	IGINITRFQTLLALH	231
1070353	IGKIQDSLSSTASAL	931
1312699	IGVTQNVLYENQKLI	909
1318459	IHADQLTPTW	624
1393841	IHADQLTPTWRVYSTGS	624

1451328	IHADQLTPTWRVYSTGSN	624
26710	IITTDNTFV	1114
1393845	IITTDNTFVSGNCDVVI	1114
1608085	IITTDNTFVSGNCDVVIG	1114
1393846	IKQYGDCLGDIAARDLI	834
1608169	IKQYGDCLGDIAARDLIC	834
1074928	ILPDPSKPSK	805
1312733	ILPDPSKPSKRSFIE	805
1310497	ILPVSMTKTSVDCTM	726
1074929	VQIDRLITGRLQSLQTYVTQ(	980
1318821	INITRFQTL	233
1312746	INITRFQTLLALHRS	233
1318829	IPFAMQMAY	896
1310503	IPFAMQMAYRFNGIG	896
1087359	IPIGAGICASY	664
1318845	IPTNFTISV	714
1312773	IRAAEIRASANLAAT	1013
1310506	IRGWIFGTTLDSKTQ	101
28511	ISGINASVVNIQKEI	1169
1393855	ISNCVADYSVLYNSASF	358
1608726	ISNCVADYSVLYNSASFS	358
1312775	ISSVLNDILSRLDKV	973
1312780	ITDAVDCALDPLSET	285
1332424	ITGRLQSLQTY	997
1070482	ITGRLQSLQTYVTQQ	997
1668465	ITRFQTLLA	235
1310513	ITRFQTLLALHRSYL	235
1310515	IVRFPNITNLCPFGE	326
1310516	IWLGFIAGLIAIVMV	1216
1330491	IYKTPPIKDF	788
1319328	IYQTSNFRV	312
1074945	IYSKHTPINL	203
1310519	KAHFPREGVFVSNGT	1086
1393860	KAHFPREGVFVSNGTHW	1086
1609103	KAHFPREGVFVSNGTHWF	1086
1312803	KCVNFNFNGLTGTGV	537
1311170	KCYGVSPTK	378
1074948	LPDPSKPSKRSFIEDLLFNK\	795
532384	KEELDKYFKNHTSPD	1149
1393862	KEELDKYFKNHTSPDVD	1149
1609487	KEELDKYFKNHTSPDVDL	1149
1074949	KEIDRLNEV	1181
1310528	KEIDRLNEVAKNLNE	1181
1319485	KFLPFQQFGR	558
1312819	KGCCSCGSCCKFDED	1245
1312823	KGYHLMSFPQSAPHG	1045
1309123	KHTPINLVRDLPQGF	206
1319519	KIADYNYKL	417
1312824	KIADYNYKLPDDFTG	417
1312838	KIQDSLSSTASALGK	933
1319559	KIYSKHTPI	202
1312849	KKFLPFQQFGRDIAD	557

1310542	KLIANQFNSAIGKIQ	921
1125080	KLNDLCFTNV	386
1310545	KLNDLCFTNVYADSF	386
1393870	KLNDLCFTNVYADSFVI	386
1074951	KLNDLCFTNVYADSFVIR	386
1541057	KLNDLCFTNVYADSFVIRGD	386
32069	KLPDDFMGCV	411
1074952	KLPDDFTGCV	424
1312875	KNFTTAPAICHDGKA	1073
1312876	KNHTSPDVDLGDISG	1157
1310546	KNLNESLIDLQELGK	1191
1393875	KNLNESLIDLQELGKYE	1191
1610665	KNLNESLIDLQELGKYEQ	1191
1671378	KNLREFVFK	187
1310548	KNTQEVFAQVKQIYK	776
1319894	KPFERDISTEI	462
1074954	KPFERDISTEIY	462
1070803	KPSKRSFIEDLLFNK	811
1597721	KQIYKTPPI	786
1074959	KQIYKTPPIKDF	786
1310550	KQIYKTPPIKDFGGF	786
1597722	KQLSSNFGA	964
1312897	KRFDNPVLPFNDGVY	77
1310551	KRISNCVADYSVLYN	356
1541116	KRISNCVADYSVLYNSASFS	356
1070815	KSNIIRGWIFGTTLD	97
1319957	KSNLKPFER	458
1549036	KSTNLVKNK	529
1312907	KSTNLVKNKCVNFNF	529
1074961	KSWMESEFRVY	150
1312917	KTQSLLIVNNATNVV	113
1393881	KTQSLLIVNNATNVVIK	113
1087599	KTQSLLIVNNATNVVIKV	113
1074962	KTSVDCTMYI	733
1312924	KTSVDCTMYICGDST	733
1312928	KVCEFQFCNDPFLGV	129
1462784	KVCEFQFCNDPFLGVYYHK	129
1074963	LGVYYHKNNKSWMESEFR\	129
1310554	KVEAEVQIDRLITGR	986
1320006	KVFRSSVLH	41
1310555	KVFRSSVLHSTQDLF	41
1320009	KVGGNYNYLY	444
1312933	KVTLADAGFIKQYGD	825
1311572	KWPWYIWLGF	1211
1310560	KWPWYIWLGFIAGLI	1211
1312935	KYEQYIKWPWYIWLG	1205
1393884	KYEQYIKWPWYIWLGFI	1205
1334466	KYEQYIKWPWYIWLGFIA	1205
1330515	LADAGFIKQY	828
1312956	LDITPCSFGGVSVIT	585
35205	LDKYFKNHTSPDVDL	1152
1312959	LDPLSETKCTLKSFT	293

1312961	LDSFKEELDKYFKNH	1145
1310565	LDSKVGGNYNYLYRL	441
1465145	LDSKVGGNYNYLYRLFRKS	441
1393890	LEILDITPCSFGGVSVI	582
1612142	LEILDITPCSFGGVSVIT	582
1074967	LEPLVDLPI	223
1320443	LGAENSVAY	699
1074969	LGAENSVAYSNN	699
1071133	LGDIAARDLICAQKF	841
1393892	LGDIAARDLICAQKFNG	841
1087608	LGDIAARDLICAQKFNGL	841
36075	LGDISGINASVVNIQ	1166
1312974	LGKLQDVVNQNAQAL	945
1310575	LGVYYHKNNKSWMES	141
1393897	LGVYYHKNNKSWMESEF	141
532715	LICAQKFNGLTVLPP	849
36481	LIDLQELGKY	1197
1309125	LIDLQELGKYEQYI	1206
1312982	LIDLQELGKYEQYIK	1197
36724	LITGRLQSL	996
1074971	LITGRLQSLQTYV	996
1310586	LITGRLQSLQTYVTQ	996
1310589	LKPFERDISTEIYQA	461
1071263	LKYNENGTITDAVDC	277
1071265	LLAGTITSGWTFGAG	877
1074974	LLALHRSYL	241
1310592	LLALHRSYLTPGDSS	241
37289	LLFNKVTLA	821
1310593	LLFNKVTLADAGFIK	821
1071268	LLHAPATVCGPKKST	517
1312997	LLIVNNATNVVIKVC	117
1310597	LLKYNENGTITDAVD	276
1676076	LLPLVSSQCV	7
37724	LLQYGSFCT	753
1313008	LLQYGSFCTQLNRAL	753
1332664	LLTDEMIAQY	864
1310603	LMDLEGKQGNFKNLR	176
1393903	LMDLEGKQGNFKNLREF	176
1612907	LMDLEGKQGNFKNLREFV	176
1313029	LMSFPQSAPHGVVFL	1049
1313032	LNDILSRLDKVEAEV	977
1313033	LNESLIDLQELGKYE	1193
1310606	LNEVAKNLNESLIDL	1186
1313043	LPDDFTGCVIAWNSN	425
1074979	NSNNLDSKVGGNYNYLYRL	425
1310609	LPDPSKPSKRSFIED	806
1393905	LPDPSKPSKRSFIEDLL	806
1613044	LPDPSKPSKRSFIEDLLF	806
1310610	LPFFSNVTWFHAIHV	56
1321049	LPFNDGVYF	84
1321058	LPIGINITRF	229
1313051	LPIGINITRFQTLLA	229

1321078	LPLVSSQCV	8
1393908	LPLVSSQCVNLTTRTQL	8
1613172	LPLVSSQCVNLTTRTQLP	8
1074980	LPPAYTNSF	24
1321084	LPPLLTDEM	861
1310611	LPPLLTDEMIAQYTS	861
1074981	LPQGFSAL	216
1332697	LPQGFSALEPL	216
1310612	LPQGFSALEPLVDLP	216
1332702	LQELGKYEQY	1200
38855	LQIPFAMQM	894
1074986	LQIPFAMQMAY	894
1310614	LQPELDSFKEELDKY	1141
38990	LQSLQTYVTQQLIRA	1001
1677313	LQSYGFQPT	492
1397166	LQSYGFQPTNGVGYQPY	492
1313075	LREFVFKNIDGYFKI	189
1471755	FVFKNIDGYFKIYSKHTPINL	189
1310618	LSETKCTLKSFTVEK	296
1313083	LSFELLHAPATVCGP	513
1310620	LSRLDKVEAEVQIDR	981
1393912	LSRLDKVEAEVQIDRLI	981
1613822	LSRLDKVEAEVQIDRLIT	981
1392288	LSRLDKVEAEVQIDRLITGR	981
1613823	LSRLDPPEAEVQIDRLIT	981
1310621	LSSNFGAISSVLNDI	966
1074989	LSSTASALGK	938
1310623	LTDEMIAQY	865
1332727	LTDEMIAQYT	865
1071338	LTDEMIAQYTSALLA	865
1310624	LTGTGVLTESNKKFL	546
1313106	LTPGDSSSGWTAGAA	249
1313108	LTPTWRVYSTGSNVF	629
1310625	LVDLPIGINITRFQT	226
1332741	LVKNKCVNF	533
1313114	LVKNKCVNFNFNGLT	533
1393916	LVKNKCVNFNFNGLTGT	533
1614059	LVKNKCVNFNFNGLTGTG	533
1313117	LVLLPLVSSQCVNLT	5
1310633	LYENQKLIANQFNSA	916
1330526	LYNSASFSTF	368
1310635	LYQDVNCTEVPVAIH	611
1313147	MDLEGKQGNFKNLRE	177
1313152	MESEFRVYSSANNCT	153
1332785	MFVFLVLLPLVSS	1
1071428	MFVFLVLLPLVSSQC	1
1393926	MFVFLVLLPLVSSQCVN	1
1087636	MFVFLVLLPLVSSQCVNL	1
1313153	MIAQYTSAL	869
1313154	MIAQYTSALLAGTIT	869
1393927	MIAQYTSALLAGTITSG	869
1614342	MIAQYTSALLAGTITSGW	869

1074998	TITSGWTFGAGAALQIPFAM	869
1313156	MLCCMTSCCSCLKGC	1233
1393928	MLCCMTSCCSCLKGCCS	1233
1087637	MLCCMTSCCSCLKGCCSC	1233
1680251	MQMAYRFNGI	900
532967	MSECVLGQSKRVDFC	1029
1313176	MSLGAENSVAYSNNS	697
1310649	MTKTSVDCTMYICGD	731
42873	MTSCCSCLK	1237
1071491	MTSCCSCLKGCCSCG	1237
1313186	MVTIMLCCMTSCCSC	1229
1322298	NASVVNIQK	1173
1313192	NASVVNIQKEIDRLN	1173
1322299	NATRFASVY	343
1313193	NCDVVIGIVNNTVYD	1125
1310653	NCTEVPVAIHADQLT	616
1313194	NCTFEYVSQPFLMDL	165
1397173	NDGVYFASTEKSNIIR	87
1313195	NDPFLGVYYHKNNKS	137
1310658	NESLIDLQEL	1194
533050	NFGAISSVLNDILSR	969
1313201	NFKNLREFVFKNIDG	185
1393940	NFNFNGLTGTGVLTESN	540
1614814	NFNFNGLTGTGVLTESNK	540
1313203	NFRVQPTESIVRFPN	317
1309132	NFSQILPDPSKPSKR	801
1479937	NFSQILPDPSKPSKRSFIE	801
1313204	NFTISVTTEILPVSM	717
1310659	NGLTVLPPLLTDEMI	856
1075001	NGVEGFNCY	481
1071575	NGVEGFNCYFPLQSY	481
1681938	NGVGYQPYR	501
1310660	NGVGYQPYRVVVLSF	501
1597230	NIADYNYKL	414
1310664	NIDGYFKIYSKHTPI	196
1393950	NIIRGWIFGTTLDSKTQ	99
1614938	NIIRGWIFGTTLDSKTQS	99
1310666	NITNLCPFGEVFNAT	331
1322395	NITRFQTL	234
1397175	NKCVNFNFNGLTGT	536
1310669	NKCVNFNFNGLTGTG	536
1310671	NKKFLPFQQFGRDIA	556
1313211	NKSWMESEFRVYSSA	149
1393952	NLAATKMSECVLGQSKR	1023
1615045	NLAATKMSECVLGQSKRV	1023
1075002	NLDSKVGGNY	440
1071580	NLLLQYGSFCTQLNR	751
44814	NLNESLIDL	1192
1087385	NLTTRTQL	17
1313223	NLTTRTQLPPAYTNS	17
1071585	NLVRDLPQGFSALEP	211
1393954	NLVRDLPQGFSALEPLV	211

1615090	NLVRDLPQGFSALEPLVD	211
1071586	NNATNVVIKVCEFQF	121
1393956	NNKSWMESEFRVYSSAN	148
1615115	NNKSWMESEFRVYSSANN	148
1313229	NNSIAIPTNFTISVT	709
1313230	NNSYECDIPIGAGIC	657
1482748	NNSYECDIPIGAGICASYQ	657
1310678	NPVLPFNDGVYFAST	81
1550978	NQFNSAIGK	925
1071651	NQFNSAIGKIQDSLS	925
1393958	NQFNSAIGKIQDSLSST	925
1615253	NQFNSAIGKIQDSLSSTA	925
1075005	NQKLIANQF	919
1313242	NQNAQALNTLVKQLS	953
1393960	NQNAQALNTLVKQLSSN	953
1087668	NQNAQALNTLVKQLSSNF	953
1329820	NQNAQALNTLVKQLSSNFG	953
1310681	NQVAVLYQDVNCTEV	606
1393962	NRALTGIAVEQDKNTQE	764
1615277	NRALTGIAVEQDKNTQEV	764
1541817	NRKRISNCVAD	354
1313244	NSASFSTFK	370
1322611	NSFTRGVYY	30
1322613	NSIAIPTNF	710
1313249	NSNNLDSKVGGNYNY	437
1393965	NTLVKQLSSNFGAISSV	960
1615442	NTLVKQLSSNFGAISSVL	960
1683930	NTQEVFAQV	777
1313257	NTQEVFAQVKQIYKT	777
1322706	NTSNQVAVLY	603
1393968	NTSNQVAVLYQDVNCTE	603
1615457	NTSNQVAVLYQDVNCTEV	603
1393969	NTVYDPLQPELDSFKEE	1135
1615463	NTVYDPLQPELDSFKEEL	1135
1310695	NVFQTRAGCLIGAEH	641
1484768	NVFQTRAGCLIGAEHVNNS	641
1310701	NVTWFHAIHVSGTNG	61
1313262	NVVIKVCEFQFCNDP	125
1322744	NVYADSFVIR	394
1313269	NYNYLYRLF	448
1075012	NYNYLYRLFRK	448
1597243	NYNYRYRLF	448
1393973	PAICHDGKAHFPREGVF	1079
1615570	PAICHDGKAHFPREGVFV	1079
1313273	PAQEKNFTTAPAICH	1069
1310704	PATVCGPKKSTNLVK	521
1310705	PAYTNSFTRGVYYPD	26
47041	PCSFGGVSVITPGTN	589
1393975	PCSFGGVSVITPGTNTS	589
1615603	PCSFGGVSVITPGTNTSN	589
1310707	PDDFTGCVIAWNSNN	426
1541908	[illegible]DDFTGCVIAWNSNNLDSK[illegible]	426

1685074	PFAMQMAYRF	897
1309913	PFAMQMAYRFNGIGV	897
47479	PFAMQMAYRFNGIGVTQ	897
1393978	PFERDISTEIYQAGSTP	463
1615654	PFERDISTEIYQAGSTPC	463
1330548	PFFSNVTWF	57
1313276	PFFSNVTWFHAIHVS	57
1393979	PFFSNVTWFHAIHVSGT	57
1087674	PFFSNVTWFHAIHVSGTN	57
1313277	PFGEVFNATRFASVY	337
1393980	PFGEVFNATRFASVYAW	337
1087675	PFGEVFNATRFASVYAWN	337
1313279	PFNDGVYFASTEKSN	85
1393981	PFNDGVYFASTEKSNII	85
1615663	PFNDGVYFASTEKSNIIR	85
1310712	PFQQFGRDIADTTDA	561
1393982	PFQQFGRDIADTTDAVR	561
1087676	PFQQFGRDIADTTDAVRD	561
1310714	PGDSSSGWTAGAAAY	251
1071768	PHGVVFLHVTYVPAQ	1057
1313281	PIGAGICASYQTQTN	665
1313282	PIKDFGGFNFSQILP	793
1313285	PINLVRDLPQGFSAL	209
1313286	PINLVRDLPQGFWAL	209
1310721	PLQSYGFQPTNGVGY	491
1393984	PLQSYGFQPTNGVGYQP	491
1615796	PLQSYGFQPTNGVGYQPY	491
1393985	PLSETKCTLKSFTVEKG	295
1615800	PLSETKCTLKSFTVEKGI	295
1313291	PLVDLPIGINITRFQ	225
1393986	PLVDLPIGINITRFQTL	225
1087683	PLVDLPIGINITRFQTLL	225
1313292	PLVSSQCVNLTTRTQ	9
1393988	PNITNLCPFGEVFNATR	330
1615820	PNITNLCPFGEVFNATRF	330
1397182	PPAYTNSFTRGVYY	25
1071788	PPAYTNSFTRGVYYP	25
1393989	PPIKDFGGFNFSQILPD	792
1615833	PPIKDFGGFNFSQILPDP	792
1393990	PPLLTDEMIAQYTSALL	862
1071794	PQGFSALEPLVDLPI	217
1313307	PQSAPHGVVFLHVTY	1053
1310725	PRRARSVASQSIIAY	681
1397183	PRTFLLKYNENGTITDA	272
1313311	PSKPSKRSFIEDLLF	809
1393995	PTNFTISVTTEILPVSM	715
1615945	PTNFTISVTTEILPVSMT	715
1075016	VVVLSFELLHAPATVCGPKK	499
1071818	PTWRVYSTGSNVFQT	631
1393996	PTWRVYSTGSNVFQTRA	631
1615952	PTWRVYSTGSNVFQTRAG	631
1310729	PVAIHADQLTPTWRV	621

1071825	PWYIWLGFIAGLIAI	1213
50166	PYRVVVLSF	507
1313324	QALNTLVKQLSSNFG	957
1071848	QDVNCTEVPVAIHAD	613
1491922	QDVNCTEVPVAIHADQLTP	613
533333	QDVVNQNAQALNTLV	949
1310733	QEKNFTTAPAICHDG	1071
1323200	QELGKYEQY	1201
1071850	QELGKYEQYIKWPWY	1201
1323207	QEVFAQVKQIY	779
1075019	QEVFAQVKQIYK	779
1366891	QFCNDPFLGVYHKNNK	134
1394001	QFCNDPFLGVYYHKNNK	134
1616213	QFCNDPFLGVYYHKNNKS	134
1310735	QFNSAIGKIQDSLSS	926
1394003	QGFSALEPLVDLPIGIN	218
1616228	QGFSALEPLVDLPIGINI	218
1394004	QGNFKNLREFVFKNIDG	183
1616242	QGNFKNLREFVFKNIDGY	183
1323249	QIAPGQTGK	409
1313338	QIAPGQTGKIADYNY	409
1313341	QIITTDNTFVSGNCD	1113
1323266	QIPFAMQMAY	895
1397188	QIPFAMQMAYRFNGIGV	895
1313344	QIYKTPPIK	787
1071969	QKFNGLTVLPPLLTD	853
1397189	QKLIANQFNSAIGKI	920
1310739	QLIRAAEIRASANLA	1011
1584233	QLIRAAEIRASANLAATKM	1011
1688328	QLNRALTGI	762
1313359	QLSSNFGAISSVLND	965
1323406	QLTPTWRVY	628
1071978	QMAYRFNGIGVTQNV	901
1313363	QNVLYENQKLIANQF	913
1394010	QPELDSFKEELDKYFKN	1142
1616461	QPELDSFKEELDKYFKNH	1142
1313368	QPFLMDLEGKQGNFK	173
1072010	QPRTFLLKYNENGTI	271
1323461	QPTESIVRF	321
1310745	QPTESIVRFPNITNL	321
1394011	QPTNGVGYQPYRVVVLS	498
1616502	QPTNGVGYQPYRVVVLSF	498
1323467	QPYRVVVL	506
51999	QPYRVVVLSF	506
1310747	QPYRVVVLSFELLHA	506
1542166	QPYRVVVLSFELLHAPATVC	506
1310750	QRNFYEPQIITTDNT	1106
1333222	QSAPHGVVF	1054
1075023	'TYVPAQEKNFTTAPAICHD(	1054
1310752	QSKRVDFCGKGYHLM	1036
1394014	QSLQTYVTQQLIRAAEI	1002
1616645	QSLQTYVTQQLIRAAEIR	1002

1313385	QSYGFQPTNGVGYQP	493
1394015	QTGKIADYNYKLPDDFT	414
1616678	QTGKIADYNYKLPDDFTG	414
1394016	QTLLALHRSYLTPGDSS	239
1616685	QTLLALHRSYLTPGDSSS	239
1323599	QTNSPRRAR	677
1313388	QTNSPRRARSVASQS	677
52672	QTYVTQQLIRAAEIR	1005
1310755	QYGDCLGDIAARDLI	836
52911	QYGSFCTQL	755
1310756	QYIKWPWYI	1208
1323673	QYIKWPWYIW	1208
1310759	RAGCLIGAEHVNNSY	646
1313403	RALTGIAVEQDKNTQ	765
1689634	RARSVASQSI	683
1323750	RASANLAATK	1019
1310761	RDISTEIYQAGSTPC	466
1329990	RDISTEIYQAGSTPCNGVEG	466
1072118	RDPQTLEILDITPCS	577
1329996	REFVFKNIDGYFKIYSK	190
1617118	REFVFKNIDGYFKIYSKH	190
1075025	REGVFVSNGTHW	1091
1310763	REGVFVSNGTHWFVT	1091
1323761	RFASVYAWNR	346
1542248	RFASVYAWNRKR	346
1310765	RFASVYAWNRKRISN	346
1542249	RFASVYAWNRKRISNCVADY	346
1087399	RFDNPVLPF	78
1394021	RFDNPVLPFNDGVYFAS	78
1313420	RFNGIGVTQNVLYEN	905
1323786	RFPNITNLCPF	328
1313423	RFQTLLALHRSYLTP	237
1075029	RISNCVADY	357
1313442	RISNCVADYSVLYNS	357
1498219	RISNCVADYSVLYNSASFS	357
1690556	RKRISNCVA	355
1542282	RKRISNCVAD	355
1072366	RKSNLKPFERDISTE	457
54507	RLDKVEAEV	983
1333325	RLDKVEAEVQI	983
1075031	RLFRKSNLK	454
54599	RLITGRLQSLQTYVTQQ	995
1309940	RLITGRLQSLQTYVTQQL	995
54680	RLNEVAKNL	1185
1313457	RLNEVAKNLNESLID	1185
1323919	RLQSLQTY	1000
54725	RLQSLQTYV	1000
1394027	RNFYEPQIITTDNTFVS	1107
1617939	RNFYEPQIITTDNTFVSG	1107
1330580	RQIAPGQTGK	408
1075039	RQIAPGQTGKIADYNYKL	408
1324060	RSVASQSII	685

1075041	RSVASQSIIAYTMSL	685
1310775	RSYLTPGDSSSGWTA	246
1394031	RSYLTPGDSSSGWTAGA	246
1618573	RSYLTPGDSSSGWTAGAA	246
1313495	RTFLLKYNENGTITD	273
1310777	RTQLPPAYTNSFTRG	21
56252	RVDFCGKGY	1039
1333410	RVQPTESIVRF	319
1313517	RVVVLSFELLHAPAT	509
1501129	RVVVLSFELLHAPATVCGP	509
1324116	RVYSTGSNV	634
1324117	RVYSTGSNVF	634
1313526	SAIGKIQDSLSSTAS	929
1310785	SALEPLVDLPIGINI	221
1072541	SANLAATKMSECVLG	1021
1324274	SANNCTFEY	162
1394035	SANNCTFEYVSQPFLMD	162
1618992	SANNCTFEYVSQPFLMDL	162
1333450	SASFSTFKCY	371
1310787	SASFSTFKCYGVSPT	371
533643	SCGSCCKFDEDDSEP	1249
1394041	SECVLGQSKRVDFCGKG	1030
1619203	SECVLGQSKRVDFCGKGY	1030
1394042	SEFRVYSSANNCTFEYV	155
1619222	SEFRVYSSANNCTFEYVS	155
57592	SEPVLKGVKL	1261
1072563	SEPVLKGVKLHYT	1261
1324353	SETKCTLKSF	297
1075045	SETKCTLKSFTVEK	297
1313542	SETKCTLKSFTVEKG	297
1333520	SFELLHAPATV	514
1310795	SFGGVSVITPGTNTS	591
1333523	SFIEDLLF	816
1310796	SFIEDLLFNKVTLAD	816
1324400	SFKEELDKY	1147
1324414	SFPQSAPHGVVF	1051
1072604	SFPQSAPHGVVFLHV	1051
1394045	SFPQSAPHGVVFLHVTY	1051
1619379	SFPQSAPHGVVFLHVTYV	1051
1313549	SFSTFKCYGVSPTKL	373
1072624	SFTRGVYYPDKVFRS	31
1313550	SFTVEKGIYQTSNFR	305
1394049	SGINASVVNIQKEIDRL	1170
1619434	SGINASVVNIQKEIDRLN	1170
1310799	SGTNGTKRFDNPVLP	71
1394052	SGTNGTKRFDNPVLPFN	71
1619518	SGTNGTKRFDNPVLPFND	71
1310800	SGWTAGAAAYYVGYL	256
1310802	SIAIPTNFTISVTTE	711
1309137	SIIAYTMSL	691
1310803	SIIAYTMSLGAENSV	691
58730	SIVAYTMSL	

1313572	SIVRFPNITNLCPFG	325
1072807	SKHTPINLVRDLPQG	205
1313576	SKRSFIEDLLFNKVT	813
1370489	SKRSFIEDLLFNKVTLA	813
1619782	SKRSFIEDLLFNKVTLAD	813
1505853	SKRSFIEDLLFNKVTLADA	813
1333568	SKRVDFCGKGY	1037
1313577	SKRVDFCGKGYHLMS	1037
1394060	SKRVDFCGKGYHLMSFP	1037
1619788	SKRVDFCGKGYHLMSFPQ	1037
1125138	SLIDLQEL	1196
59161	SLIDLQELGK	1196
1310809	SLIDLQELGKYEQYI	1196
59162	SLIDLQELGKYEQYIKW	1196
1310810	SLLIVNNATNVVIKV	116
1072822	SLSSTASALGKLQDV	937
1310814	SNFRVQPTESIVRFP	316
1394062	SNFRVQPTESIVRFPNI	316
1542532	SNFRVQPTESIVRFPNITNL	316
1313627	SNGTHWFVTQRNFYE	1097
1394065	SNLLLQYGSFCTQLNRA	750
1619999	SNLLLQYGSFCTQLNRAL	750
1394066	SNNSIAIPTNFTISVTT	708
1620005	SNNSIAIPTNFTISVTTE	708
1313628	SNQVAVLYQDVNCTE	605
60024	SPDVDLGDISGINAS	1161
1324899	SPRRARSV	680
1311590	SPRRARSVA	680
1394068	SPRRARSVASQSIIAYT	680
1620210	SPRRARSVASQSIIAYTM	680
1313652	SQCVNLTTRTQLPPA	13
1313657	SQSIIAYTMSLGAEN	689
1333782	SSANNCTFEY	161
1310820	SSANNCTFEYVSQPF	161
1508891	SSANNCTFEYVSQPFLMDL	161
1394071	SSNFGAISSVLNDILSR	967
1620549	SSNFGAISSVLNDILSRL	967
1310821	SSTASALGK	939
1394073	SSTASALGKLQDVVNQN	939
1620617	SSTASALGKLQDVVNQNA	939
1313684	SSVLHSTQDLFLPFF	45
1394074	SSVLNDILSRLDKVEAE	974
1620632	SSVLNDILSRLDKVEAEV	974
1620633	SSVLNDILSRLDPPEAEV	974
1333801	STECSNLLLQY	746
1309139	STECSNLLLQYGSFC	746
1313689	STEIYQAGSTPCNGV	469
1325105	STGSNVFQTR	637
1072943	STGSNVFQTRAGCLI	637
1313696	STPCNGVEGFNCYFP	477
1394075	STPCNGVEGFNCYFPLQ	477
1620718	STPCNGVEGFNCYFPLQS	477

1333812	STQDLFLPF	50
1075065	STQDLFLPFF	50
1394076	STQDLFLPFFSNVTWFH	50
1620734	STQDLFLPFFSNVTWFHA	50
1325128	SVASQSIIAY	686
1310825	SVASQSIIAYTMSLG	686
1310826	SVITPGTNTSNQVAV	596
1394079	SVITPGTNTSNQVAVLY	596
1620817	SVITPGTNTSNQVAVLYQ	596
1310827	SVLHSTQDLFLPFFS	46
62221	SVLNDILSR	975
1075066	SVLNDILSRL	975
1392439	SVLYNSASF	366
1310828	SVLYNSASFSTFKCY	366
1542626	SVLYNSASFSTFKCYGVSPT	366
1597815	SVTTEILPV	721
1072965	SVTTEILPVSMTKTS	721
1075068	KTSVDCTMYICGDSTECSNI	721
1325172	SVYAWNRKR	349
1313713	SVYAWNRKRISNCVA	349
1333886	SWMESEFRV	151
1325190	SWMESEFRVY	151
1310830	SWMESEFRVYSSANN	151
1394083	SYECDIPIGAGICASYQ	659
1620909	SYECDIPIGAGICASYQT	659
1313719	SYQTQTNSPRRARSV	673
1075070	SYQTQTNSPRRARSVA	673
1394085	SYQTQTNSPRRARSVAS	673
1087736	SYQTQTNSPRRARSVASQ	673
1313732	TAPAICHDGKAHFPR	1077
1310833	TASALGKLQDVVNQN	941
1310834	TDAVDCALDPLSETK	286
1313735	TDAVRDPQTLEILDI	573
1333921	TDEMIAQY	866
1330164	TDEMIAQYTSALLA	866
1309140	TDEMIAQYTSALLAG	866
1073276	TDNTFVSGNCDVVIG	1117
1325401	TECSNLLLQY	747
1087408	TEILPVSMTK	724
1394090	TEIYQAGSTPCNGVEGF	470
1621193	TEIYQAGSTPCNGVEGFN	470
1075071	TEKSNIIRGW	95
1325420	TESIVRFPNITNL	323
1394091	TESIVRFPNITNLCPFG	323
1621247	TESIVRFPNITNLCPFGE	323
1325421	TESNKKFLPFQQF	553
1073280	TESNKKFLPFQQFGR	553
1397507	TFEYVSQPF	167
1333951	TFEYVSQPFLM	167
1310841	TFKCYGVSPTKLNDL	376
1542737	TFKCYGVSPTKLNDLCFTNV	376
1394092	TFLLKYNENGTITDAVD	274

1621279	TFLLKYNENGTITDAVDC	274
63951	TGRLQSLQTYVTQQL	998
1394094	TGSNVFQTRAGCLIGAE	638
1621375	TGSNVFQTRAGCLIGAEH	638
1394095	TGTGVLTESNKKFLPFQ	547
1621380	TGTGVLTESNKKFLPFQQ	547
1313741	TGVLTESNKKFLPFQ	549
1394096	THWFVTQRNFYEPQIIT	1100
1621419	THWFVTQRNFYEPQIITT	1100
1597376	TIADYNYKL	417
1073433	TIMLCCMTSCCSCLK	1231
1310847	TITSGWTFGAGAALQ	881
1073438	TKLNDLCFTNVYADS	385
1310848	TKRFDNPVLPFNDGV	76
1325536	TLADAGFIK	827
1394099	TLADAGFIKQYGDCLGD	827
1621584	TLADAGFIKQYGDCLGDI	827
1075075	TLDSKTQSL	109
1313752	TLDSKTQSLLIVNNA	109
1310850	TLEILDITPCSFGGV	581
1313756	TLKSFTVEK	302
1330211	TLKSFTVEKGIYQTSNF	302
1621623	TLKSFTVEKGIYQTSNFR	302
1075077	GDSSSGWTAGAAAYYVGYL	240
1310852	TLVKQLSSNFGAISS	961
1310854	TMSLGAENSVAYSNN	696
1310855	TNFTISVTTEILPVS	716
1313767	TNGTKRFDNPVLPFN	73
1313768	TNLCPFGEVFNATRF	333
1310857	TNLVKNKCVNFNFNG	531
1313769	TNSFTRGVYYPDKVF	29
1394101	TNSFTRGVYYPDKVFRS	29
1621710	TNSFTRGVYYPDKVFRSS	29
1313770	TNVYADSFVIRGDEV	393
1394102	TNVYADSFVIRGDEVRQ	393
1087748	TNVYADSFVIRGDEVRQI	393
1325795	TPCSFGGVSV	588
1075079	TPINLVRDL	208
1310860	TPPIKDFGGFNFSQI	791
1330227	TQDLFLPFF	51
1310861	TQDLFLPFFSNVTWF	51
1330231	TQEVFAQVKQIYKTPPI	778
1621919	TQEVFAQVKQIYKTPPIK	778
1087411	TQLNRALTGI	761
1310863	TQLNRALTGIAVEQD	761
1518044	TQLNRALTGIAVEQDKNTQ	761
1518045	QLNRALTGIAVEQDKNTQEV	761
1394103	TQLPPAYTNSFTRGVYY	22
1621943	TQLPPAYTNSFTRGVYYP	22
65906	TQNVLYENQK	912
534141	TQQLIRAAEIRASAN	1009
1394104	TQQLIRAAEIRASANLA	1009

1087755	TQQLIRAAEIRASANLAA	1009
1073475	TQRNFYEPQIITTDN	1105
1310864	TQTNSPRRARSVASQ	676
1313795	TRAGCLIGAEHVNNS	645
1394105	TRAGCLIGAEHVNNSYE	645
1621969	TRAGCLIGAEHVNNSYEC	645
1334060	TRFASVYAW	345
1313796	TRFASVYAWNRKRIS	345
1518321	TRFASVYAWNRKRISNCVA	345
1518322	SVYAWNRKRISNCVADYSV	345
1310865	TRFQTLLALHRSYLT	236
1313797	TRGVYYPDKVFRSSV	33
1334061	TRTQLPPAY	20
1394106	TSGWTFGAGAALQIPFA	883
1622060	TSGWTFGAGAALQIPFAM	883
1397216	TSNFRVQPTESIVRF	315
1087414	TSNQVAVLY	604
1310870	TTAPAICHDGKAHFP	1076
1310871	TTDNTFVSGNCDVVI	1116
1700707	TTRTQLPPA	19
1075085	TVYDPLQPELDSFK	1136
1310874	TVYDPLQPELDSFKE	1136
1330639	TYVPAQEKNF	1066
1334122	TYVPAQEKNFT	1066
1310875	TYVPAQEKNFTTAPA	1066
1310876	TYVTQQLIRAAEIRA	1006
1392485	VADYSVLYNSASFST	362
1326246	VAIHADQLTPTW	622
534192	VAKNLNESLIDLQEL	1189
1326261	VASQSIIAY	687
1394111	VASQSIIAYTMSLGAEN	687
1622559	VASQSIIAYTMSLGAENS	687
1313836	VAYSNNSIAIPTNFT	705
1313838	VDCALDPLSETKCTL	289
1310877	VDCTMYICGDSTECS	736
1394114	VDCTMYICGDSTECSNL	736
1622602	VDCTMYICGDSTECSNLL	736
1075088	VEAEVQIDRLITGR	987
1397221	VEGFNCYFPLQSYGFQPT	483
1326322	VFAQVKQIY	781
1310884	VFAQVKQIYKTPPIK	781
1326409	VFKNIDGYF	193
1313847	VFKNIDGYFKIYSKH	193
1310885	VFLHVTYVPAQEKNF	1061
1310886	VFNATRFASVYAWNR	341
1334171	VFVSNGTHW	1094
1326455	VFVSNGTHWF	1094
1326471	VGGNYNYLY	445
1073698	VGGNYNYLYRLFRKS	445
1330284	VGGNYNYLYRLFRKSNLKP	445
1334183	VGYLQPRTFL	267
1394118	VGYLQPRTFLLKYNENG	267

1623020	VGYLQPRTFLLKYNENGT	267
1334184	VGYQPYRVV	503
1313864	VIAWNSNNLDSKVGG	433
1313865	VIGIVNNTVYDPLQP	1129
1394119	VIKVCEFQFCNDPFLGV	127
1623119	VIKVCEFQFCNDPFLGVY	127
1310891	VIRGDEVRQIAPGQT	401
1313871	VITPGTNTSNQVAVL	597
1313875	VKQIYKTPPIKDFGG	785
1394123	VKQIYKTPPIKDFGGFN	785
1087780	VKQIYKTPPIKDFGGFNF	785
1313879	VLGQSKRVDFCGKGY	1033
1310896	VLKGVKLHY	1264
1310899	VLLPLVSSQCVNLTT	6
69657	VLNDILSRL	976
1310902	VLNDILSRLDKVEAE	976
1334231	VLPFNDGVYFA	83
1075093	VLPFNDGVYFASTEK	83
1075094	VLPPLLTDEMIAQYT	860
1394127	VLSFELLHAPATVCGPK	512
1623349	VLSFELLHAPATVCGPKK	512
1334234	VLTESNKKF	551
1310904	VLTESNKKFLPFQQF	551
1555419	VLYENQKLI	915
1394130	VLYQDVNCTEVPVAIHA	610
1623361	VLYQDVNCTEVPVAIHAD	610
1075096	[illegible]VAIHADQLTPTWRVYSTGS	610
1073912	VNIQKEIDRLNEVAK	1177
1394133	VNIQKEIDRLNEVAKNL	1177
1087783	VNIQKEIDRLNEVAKNLN	1177
1310909	VNLTTRTQLPPAYTN	16
1394134	VNNATNVVIKVCEFQFC	120
1623423	VNNATNVVIKVCEFQFCN	120
1310910	VNNSYECDIPIGAGI	656
1313901	VNNTVYDPLQPELDS	1133
1073938	VQIDRLITGRLQSLQ	991
1075108	VQPTESIVRFPNITNLCPF	320
1313917	VRDLPQGFSALEPLV	213
1310916	VRDPQTLEILDITPC	576
70718	VRFPNITNL	327
1394138	VRQIAPGQTGKIADYNY	407
1623821	VRQIAPGQTGKIADYNYK	407
1313921	VSMTKTSVDCTMYIC	729
1394143	VSMTKTSVDCTMYICGD	729
1087786	VSMTKTSVDCTMYICGDS	729
1310921	VSNGTHWFVTQRNFY	1096
1310922	VSQPFLMDLEGKQGN	171
1310923	VSSQCVNLTTRTQLP	11
1706303	VTLADAGFIK	826
1310925	VTLADAGFIKQYGDC	826
1310927	VTQNVLYENQKLIAN	911
1394146	VTQNVLYENQKLIANQF	911

1330323	VTQNVLYENQKLIANQFN	911
1394147	VTTEILPVSMTKTSVDC	722
1624131	VTTEILPVSMTKTSVDCT	722
1597845	VTWFHAIHV	62
1310928	VTYVPAQEK	1065
1313930	VTYVPAQEKNFTTAP	1065
1394148	VTYVPAQEKNFTTAPAI	1065
1087787	VTYVPAQEKNFTTAPAIC	1065
71663	VVFLHVTYV	1060
1394149	VVIGIVNNTVYDPLQPE	1128
1624207	VVIGIVNNTVYDPLQPEL	1128
1310930	VVIKVCEFQFCNDPF	126
1073956	VVLSFELLHAPATVC	511
1310931	VVNIQKEIDRLNEVA	1176
1310932	VVNQNAQALNTLVKQ	951
1311525	VYAWNRKRI	350
1543315	VYAWNRKRIS	350
71996	VYDPLQPEL	1137
1327418	VYDPLQPELDSF	1137
1313938	VYDPLQPELDSFKEE	1137
1327695	VYSSANNCTF	159
1075111	VYSSANNCTFEY	159
1313944	VYSTGSNVF	635
1075112	VYSTGSNVFQTR	635
1327741	VYYPDKVF	36
1625462	VYYPDKVFR	36
1075113	VYYPDKVFRSSVLH	36
1310935	VYYPDKVFRSSVLHS	36
1394152	VYYPDKVFRSSVLHSTQ	36
1624375	VYYPDKVFRSSVLHSTQD	36
1394153	WFHAIHVSGTNGTKRFD	64
1624434	WFHAIHVSGTNGTKRFDN	64
1327761	WFVTQRNFY	1102
1075116	WIFGTTLDSK	104
72717	WLGFIAGLIAIVMVT	1217
1313960	WNRKRISNCVADYSV	353
1532692	WNRKRISNCVADYSVLYNS	353
1310945	WNSNNLDSKVGGNYN	436
1543369	VNSNNLDSKVGGNYNYLYR	436
1394157	WPWYIWLGFIAGLIAIV	1212
1624516	WPWYIWLGFIAGLIAIVM	1212
1313967	WRVYSTGSNVFQTRA	633
1327824	WTAGAAAYY	258
1075117	WTAGAAAYYVGY	258
1327836	WTFGAGAAL	886
1310947	WTFGAGAALQIPFAM	886
1310948	YADSFVIRGDEVRQI	396
1543393	YADSFVIRGDEVRQIAPGQT	396
1327895	YAWNRKRI	351
1309143	YAWNRKRISNCVADY	351
1394158	YAWNRKRISNCVADYSV	351
1624638	YAWNRKRISNCVADYSVL	351

1075118	/ADYSVLYNSASFSTFKCYG	351
1327917	YECDIPIGAGI	660
1327923	YENQKLIANQF	917
1313987	YENQKLIANQFNSAI	917
1330361	YENQKLIANQFNSAIGKIQ	917
1311601	YEQYIKWPW	1206
1075120	YEQYIKWPWYI	1206
1309144	YEQYIKWPWYIWLGF	1206
1074069	YFASTEKSNIIRGWI	91
1075121	YFPLQSYGF	489
1313990	YFPLQSYGFQPTNGV	489
1346790	YFQPRTFLL	
1313992	YGDCLGDIAARDLIC	837
1328001	YGFQPTNGV	495
1310955	YGSFCTQLNRALTGI	756
1313994	YHKNNKSWMESEFRV	145
1075122	YHLMSFPQSA	1047
1310956	YICGDSTECSNLLLQ	741
1314002	YIKWPWYIWLGFIAG	1209
1314005	YKTPPIKDFGGFNFS	789
1593848	YLQLRTFLL	
1593850	YLQPRIFLL	
1309147	YLQPRTFLL	269
1314014	YLQPRTFLLKYNENG	269
1593851	YLQPSTFLL	
1074201	YLYRLFRKSNLKPFE	451
1397512	YNSASFSTF	369
1314022	YNSASFSTFKCYGVS	369
1074214	YNYKLPDDFTGCVIA	421
1394168	YNYKLPDDFTGCVIAWN	421
1624985	YNYKLPDDFTGCVIAWNS	421
1328431	YNYLYRLFR	449
1314023	YNYLYRLFRKSNLKP	449
1330382	YNYLYRLFRKSNLKPFE	449
1087807	YNYLYRLFRKSNLKPFER	449
1314028	YQAGSTPCNGVEGFN	473
1328471	YQDVNCTEV	612
1334394	YQPYRVVVL	505
1314034	YQPYRVVVLSFELLH	505
1394171	YQPYRVVVLSFELLHAP	505
1087809	YQPYRVVVLSFELLHAPA	505
1314036	YQTSNFRVQPTESIV	313
1537200	YQTSNFRVQPTESIVRFPN	313
1394173	YRFNGIGVTQNVLYENQ	904
1625097	YRFNGIGVTQNVLYENQK	904
1314038	YRLFRKSNLKPFERD	453
1394175	YSKHTPINLVRDLPQGF	204
1625152	YSKHTPINLVRDLPQGFS	204
1310978	YSTGSNVFQTRAGCL	636
1075125	YSVLYNSASFSTFK	365
1314047	YSVLYNSASFSTFKC	365
1394178	YSVLYNSASFSTFKCYG	365

1625177	YSVLYNSASFSTFKCYGV	365
1328800	YTMSLGAENSVAY	695
1328804	YTNSFTRGVY	28
1334416	YTNSFTRGVYY	28
1314060	YTSALLAGTITSGWT	873
1310979	YVGYLQPRTFLLKYN	266
1328953	YYHKNNKSW	144
1074296	YYPDKVFRSSVLHST	37
1711597	YYVGYLQPR	265
1329031	YYVGYLQPRTF	265
1075131	YYVGYLQPRTFLL	265
1314078	YYVGYLQPRTFLLKY	265

Note: The epitopes mutated in Omicron variant versus Wuhan-1 are indicated in red.
